# Synthesis of Cyclobutane‐Containing Tricyclic β‐Lactams Based on a Saturated Scaffold Enabled by Iron‐catalysed [2 + 2]‐Cycloaddition

**DOI:** 10.1002/chem.202502476

**Published:** 2025-09-26

**Authors:** Lea Freitag, Johannes Zeh, Levi A. Ziegenhagen, Felix J. Becker, Dragoș‐Adrian Roșca

**Affiliations:** ^1^ Anorganisch‐Chemisches Institut Universität Heidelberg Im Neuenheimer Feld 270 69120 Heidelberg Germany; ^2^ Institut des Sciences Chimiques de Rennes Université de Rennes Rennes F‐35000 France

**Keywords:** bioactive compounds, carbene C─H insertion, cycloaddition, iron catalysis, β‐lactams

## Abstract

Highly saturated, three‐dimensional β‐lactams are valuable motifs in medicinal chemistry, yet general routes to cyclobutane‐fused analogues remain scarce. Here we disclose a concise strategy that delivers pyrrolidine‐, piperidine‐, and azepane‐based tricyclic β‐lactams. A pyrimidinediimine‐iron catalyst first constructs the cyclobutane ring and the *N*‐heterocycle in one step through an intermolecular [2 + 2]‐cycloaddition of allyl amines; a subsequent photochemical intramolecular C─H insertion then forges the β‐lactam. The major products adopt rigid, cage‐like conformations confirmed for the pyrrolidine series by single‐crystal X‐ray diffraction. Comprehensive conformer sampling (using CREST), DFT‐calculated NMR shifts, and DP4 statistical analysis establish the stereochemistry across the library. Strain‐release opening of the β‐lactam ring furnishes methylphenidate analogues in a single step, underscoring the scaffolds’ synthetic versatility. Comparative studies on the corresponding bicyclic systems highlight the unique three‐dimensionality imparted by the additional cyclobutane ring, further expanding the toolbox for lead‐oriented synthesis.

## Graphical abstract

First syntheses of pyrrolidine‐, piperidine‐, and azepane tricyclic β‐lactams based on a saturated scaffold are achieved through an Fe‐pyrimidinediimine‐catalysed [2 + 2] cycloaddition of allylamines analogues followed by photo‐induced intramolecular C─H insertion. The cage‐like β‐lactam scaffolds enable strain‐release access to methylphenidate analogues and expand the three‐dimensional chemical space.

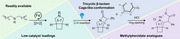

## 1 Introduction

Escalating microbial resistance has rekindled the search for unexplored chemical space that can out‐manoeuvre evolving bacterial defenses. β‐Lactam–based antibiotics remain among the safest and most clinically successful agents,^[^ ^1^ ^]^ yet nearly all approved members rely on comparatively simple bicyclic frameworks ‐ typically a β‐lactam fused to a five‐ or six‐membered heterocycle such as a pyrrolidine (penams/carbapenam) or piperidine (cephems/carbocephems):^[^ ^2^ ^]^ Their clinical success relies on the highly strained four‐membered azetidinone ring, which undergoes rapid acylation of penicillin‐binding proteins (PBPs) yet is sufficiently robust to survive distribution in vivo. Unfortunately, the same reactivity makes the scaffold vulnerable to enzymatic hydrolysis by an ever‐expanding arsenal of β‐lactamases, fueling an urgent search for new β‐lactam architectures that can evade resistance mechanisms.^[^ ^3^ ^]^

**Figure 1:**
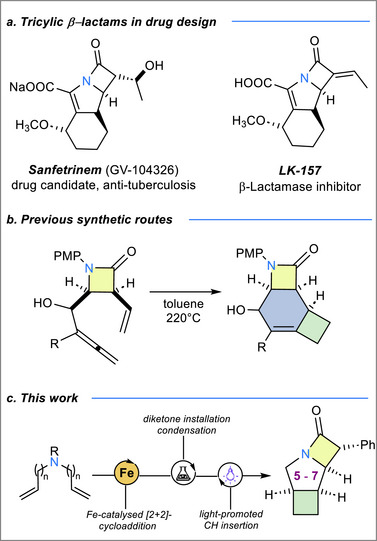
a) Drug candidates based on tricyclic β‐lactams. b) Literature precedence for the synthesis of piperidine‐based tricyclic β‐lactams. c) Current approach, based on iron‐catalysis and photoactivation.

Although decades of optimization have yielded potent derivatives, the structural repertoire around the β‐lactam nucleus is still remarkably narrow. Introducing a third ring—especially a highly strained, saturated cyclobutane— is particularly attractive: it simultaneously boosts three‐dimensionality through a higher proportion of sp^3^‐hybridized carbon atoms (“Escape from flatland”)^[^ ^4^ ^]^ and embeds latent strain that can be exploited in downstream transformations. Moreover, the enhanced conformational rigidity is expected to enable precise placement of substituents for tuning spectrum and potency, therefore allowing better control over the chemical space. Yet, for all their promise, tricyclic β‐lactam motifs are limited to a handful of examples relying on cyclohexane‐fused systems^[^ ^5^, ^6^, ^7^, ^8^, ^9^, ^10^, ^11^, ^12^ ^]^ (Figure 1a), while cyclobutane‐annulated analogues remain conspicuously absent. Their scarcity is rooted in synthetic intractability: conventional photochemical [2 + 2]‐cycloadditions demand high‐energy irradiation, offer narrow substrate scope, and often become impractical beyond milligram scale, discouraging broader exploration of this scaffold. Current workarounds typically graft allene or alkene tethers onto the β‐lactam, then drive a thermal [2 + 2]‐cycloaddition, yielding a cyclohexene ring, which would need to be further reduced. Because orbital‐symmetry rules dictate a suprafacial‐antarafacial alignment in the transition state, these reactions demand forcing temperatures, severely restricting functional‐group tolerance and scalability (Figure 1b).^[^ ^13^ ^]^

Herein we present an alternative strategy, which significantly enriches the library of tricyclic β‐lactam motifs, relying on *(i)* an iron‐based [2 + 2]‐cycloaddition protocol which simultaneously constructs the cyclobutane ring fused to the *N*‐heterocycle using readily available allylamine derivatives and *(ii)* an intramolecular C─H insertion reaction which furnishes the β‐lactam ring at the later stage of the synthesis (Figure 1c).

## 2 Results and Discussion

### 2.1 Synthesis of Bicyclic and Tricyclic β‐lactams

**Scheme 1:**
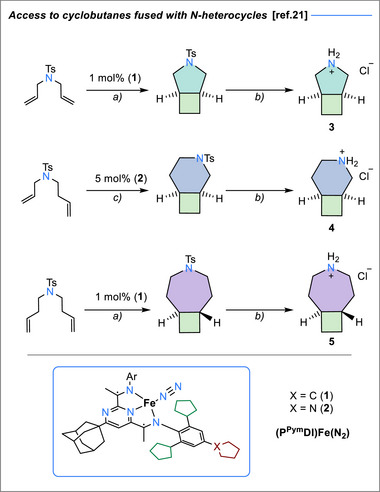
Conditions: a) 1 mol% (**1**), toluene, RT, 85%; b) Na‐naphtalenide, THF, RT, 16 h then HCl, THF, 0 °C, RT, 99%; c) 5 mol% (**2**), toluene, RT, 87%.

**Scheme 2:**
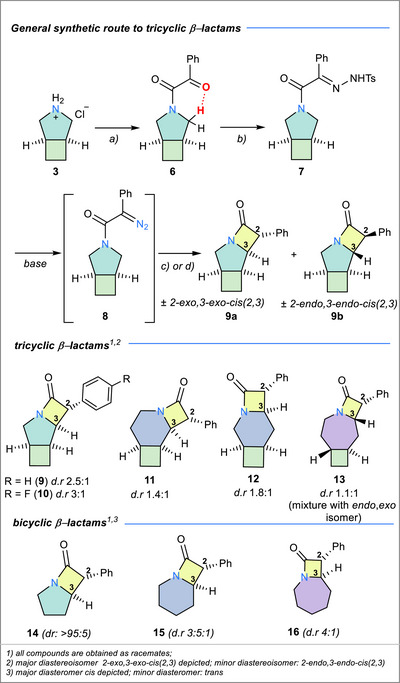
Conditions for the synthesis of **9**: a) pyridine (1.6 equiv.), phenylglyoxalic acid (0.7 equiv.), DMF (1.1 equiv.), oxalyl chloride (1.0 equiv.), CH_2_Cl_2_ 0 °C →RT, 16 h, 81%; b) tosylhydrazide, H_2_SO_4_, EtOH, 85 °C, 16 h, 49%; c) Thermal route: LiHMDS or KO*^t^* Bu, toluene, RT then 100 °C, 2 h, 69%, **9a**:**9b** 3:1; d) Photochemical route: Cs_2_CO_3_, CH_2_Cl_2_, *hν* 390 nm, 22 h, yield 47%, **9a**:**9b** ratio 3:1. For the yields of larger‐ring tricyclic analogues (**10**–**13**) as well as the synthesis of bicyclic derivatives **14**‐ **16**, see the SI.

Previous research in our group has focused on developing robust iron‐based cycloaddition protocols,^[^ ^14^, ^15^ ^]^ where diazine‐based redox‐active ligands play an essential role in catalyst stability and functional‐group tolerance.^[^ ^16^, ^17^, ^18^, ^19^ ^]^ Using this approach, we have improved and expanded the scope of an iron‐catalysed thermal [2 + 2]‐cycloaddition,^[^ ^20^ ^]^ employing suitably functionalized pyrimidineiimine‐ligands (**1** or **2**, Scheme 1). This protocol allowed the use of cheap and easily accessible allyl amine derivatives as substrates, yielding access to cyclobutane‐fused pyrroline, piperidine, and azepane frameworks.^[^ ^21^ ^]^ Subsequent tosyl group removal under radical conditions (using sodium naphthalenide), followed by an acidic work‐up, delivers the amine hydrochlorides (**3**–**5**) in near‐quantitative yield (Scheme 1). Interestingly, although more electron‐deficient aryl sulfones usually deprotect more readily,^[^ ^22^ ^]^ deprotection of tosyl groups ultimately furnished higher isolated yields than the *p*‐fluoroaryl analogues, since the removal of the by‐products from tosyl deprotection was more facile. The downstream functionalization for the *N*‐building blocks follows an analogous reaction sequence for the pyrrolidine, piperidine, and azepane derivatives, and will herein be described only for the azabicyclo[3.2.0]heptanes (**3**) (Scheme 2). The corresponding hydrochloride (**3**) was deprotonated and reacted with the in situ generated oxo‐phenylacetyl‐chloride.^[^ ^23^ ^]^ The resulting pyrrolidinyldione **6** exhibits strong hydrogen bonding between the β‐oxo group of the installed arm and the α‐CH groups of the pyrrolidine ring, which become diastereotopic and thus breaking the symmetry of the pyrrolidine ring. The locked tricylic conformation persists also in chloroform solutions, as revealed by ^1^H and ^13^C{^1^H} NMR spectroscopy. Acid‐catalysed condensation with tosylhydrazide gives rise to the corresponding hydrazone (**7**), setting the stage for the intramolecular C─H insertion reaction.

Subsequently, the α‐diazo amide (**8**) was generated in situ in the presence of a base (*vide infra*), which upon thermal or photochemical activation, forms the corresponding carbenes.^[^ ^24^, ^25^ ^]^ Intramolecular C─H insertion delivered the desired doubly annulated β‐lactams (**9**). Ultimately, the photocyclization route inspired by König et al.^[^ ^26^ ^]^ was selected for the synthesis of the *N*‐heterocyclic rings due to the ease of purification of the resulting products and avoidance of strong bases (*vide infra*). Interestingly, the intramolecular cyclization reaction yielded exclusively a *cis*‐configured β‐lactam ring (i.e., *cis*(2,3) stereoisomer). **9** was obtained as a mixture of two stereoisomers, with a 2‐*exo*,3‐*exo* (**9a**): 2‐*endo*,3‐*endo* (**9b**) ratio of 3:1 irrespective of the route chosen (Scheme 2). The presence of only two (of the possible four) diastereoisomers was further confirmed by the synthesis of the *p*‐F‐phenyl analogue of **9**, where a similar ratio of 2.5:1 between **10a** (*exo*,*exo*) and **10b** (*endo*,*endo*) was observed by ^19^F NMR spectroscopy. The identity of the *exo*,*exo* as the major isomer was confirmed by crystallographic studies. The X‐ray structure of **9a** exhibits the formation of a cage‐like structure, dictated by the all‐*cis* arrangement of the substituents. The resulting concave conformation brings the axial hydrogen atoms into proximity, also confirmed by NOESY conducted on **10b**. While the minor *endo*,*endo* isomer (**9b** or **10b**) could not be crystallized, its identity was established through quantum‐chemical modelling, calculation of the NMR chemical shifts and comparison with the experimental spectroscopic data (*vide infra*). The same synthetic route was then applied to the synthesis of the piperidine and azepane analogues of **9** (**11** – **13**, Scheme 2). Since the piperidine‐analogue lacks a *C*_2_‐rotational axis, the C─H insertion reaction yields two types of regioisomers (**11** and **12**) in a ratio of 1.6:1 which could be separated by column chromatography. Each of these regioisomers is obtained as a mixture of inseparable *exo*,*exo* and *endo*,*endo* stereoisomers (*d.r*. *ca*. 1.5:1). For the azepane‐derived tricyclic β‐lactam **13**, the intramolecular C─H insertion proved markedly less selective, furnishing all four stereoisomers. In contrast to the pyrrolidine and piperidine analogues **9**–1**2** — which selectively deliver the *cis*(2,3) series—compound **13** formed chiefly the *trans*(2,3) isomer (∼85%), isolated as an equimolar mixture of *exo,endo* and *endo,exo* diastereomers. This switch in stereochemical preference likely stems from the *trans* configuration of the cyclobutane ring embedded in the azepane precursor, whereas the five‐ and six‐membered congeners emerge from the iron‐catalyzed [2 + 2]‐cycloaddition with an inherent *cis*‐cyclobutane configuration.^[^ ^21^ ^]^ It is also interesting to note that the stereoselectivity of the insertive C─H cyclization decreases with the increase of the *N*–heterocycle ring size (3:1 for **9**, ∼1.5:1 for **11** and **12** and 1.1:1 for **13**).

**Figure 2:**
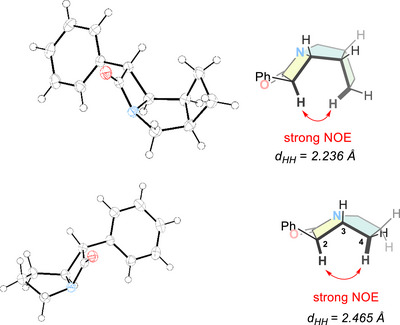
Top: (left) Solid state molecular structure of **9a** 2‐*exo*, 3‐*exo*, *cis* (2,3) (thermal ellipsoids are set at 50% probability; (right) ChemDraw depiction highlighting cage‐structure and diagnostic NOE contacts. Bottom: (left) Solid state molecular structure of **14** *cis* (2,3) (thermal ellipsoids are set at 50% probability); (right) ChemDraw depiction.

To enable a direct comparison between tricyclic β‐lactams **9**–**13** and their bicyclic counterparts, analogues **14**–**16** were synthesized following similar synthetic procedures. The synthesis of the piperidine‐derived β‐lactam (**15**) has previously been explored in several literature reports, particularly in the context of accessing bioactive methylphenidate analogues (type **22**, Scheme 4, vide infra), commercially employed as central nervous system stimulants.^[^ ^23^, ^27^, ^28^, ^29^, ^30^ ^]^ The *cis* isomer is the major one observed in all cases and is formed almost exclusively in the case of the pyrrolidine‐derivative **14**, while for the piperidine **15** and azepane **16** analogues, the selectivity is poorer (*d.r*. 3.5:1). For the pyrrolidine derivative **14**, the major isomer could also be structurally characterized (Figure 2, bottom left) and its structure in solution was corroborated by NOESY, where short contacts between H2 and H4 could be detected (Figure 2, bottom right).

### 2.2 NMR Assignment of Diastereoisomers

To further validate the assignments of the diastereomers, quantum chemical calculations were performed to determine the relative energy differences between various isomers and to calculate their corresponding NMR spectra.

**Figure 3:**
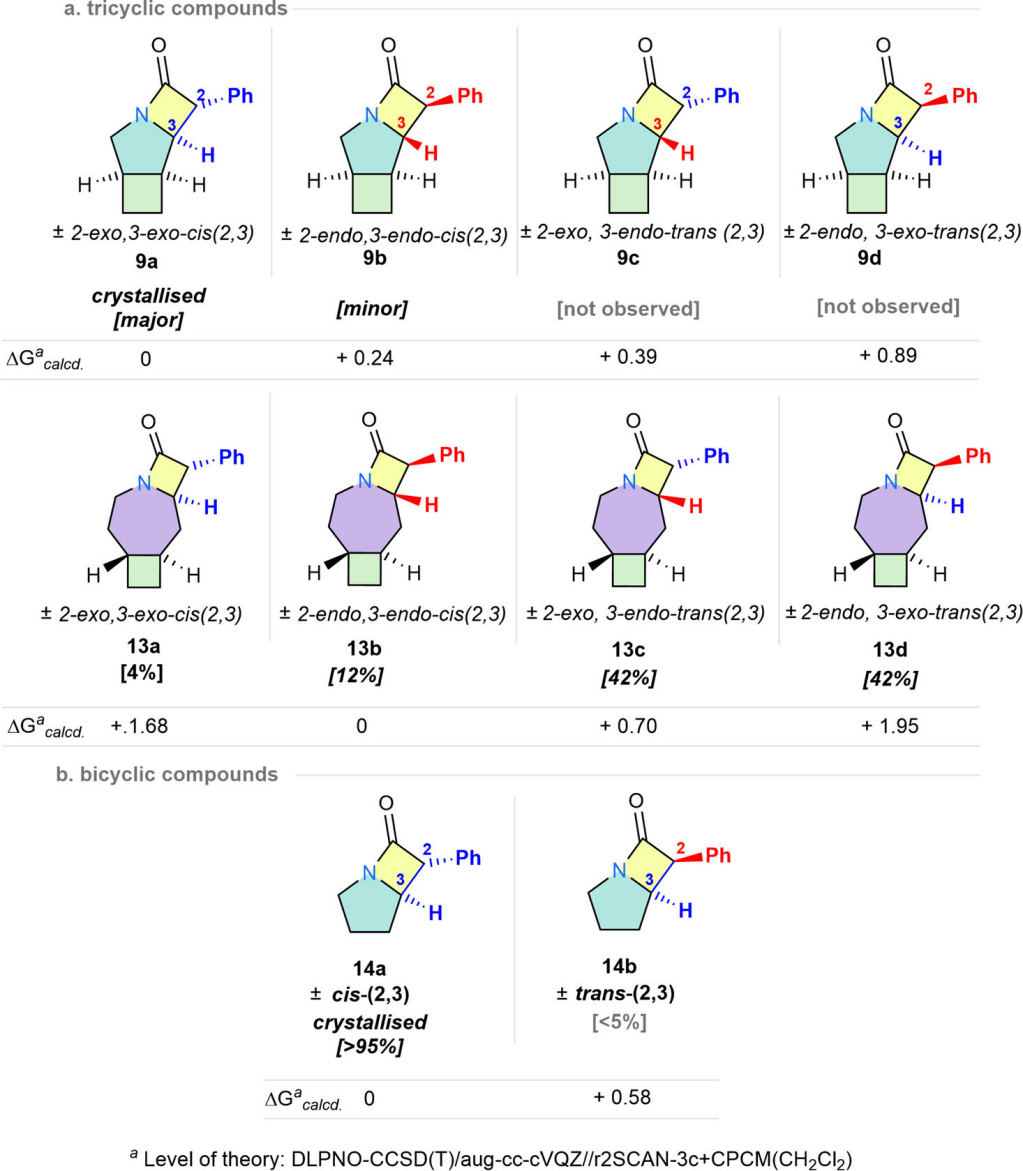
Possible diastereomeric mixtures resulting from the intramolecular C─H insertion/cyclization reaction, exemplified on the pyrrolidine‐containing compounds **9** and **14**, as well as the azepane‐derivative **13**. The observed isomers and their relative energies to the other possible isomers are indicated. For a full analysis of the piperidine‐ and azepane‐based ring systems, see the SI.

All four possible stereoisomers of compounds **9**, **11**, **12**, and **13**, as well as the two possible stereoisomers of compounds **14**–**16**, underwent conformational analysis using the Conformer‐Rotamer Sampling Tool (CREST) algorithm.^[^ ^31^, ^32^, ^33^ ^]^ The resulting ensemble was then further analyzed with Commandline Energetic Sorting (CENSO) algorithm,^[^ ^34^ ^]^ which involves an initial pre‐screening via single‐point calculations (PBE‐D3/def2‐SV(P)), followed by geometry optimization and refinement using density functional theory (DFT) at the ωB97x‐v /def2‐TZVP//r2‐SCAN‐3c level for all conformers within a 3.5 kcal mol^−1^ threshold. For a more reliable comparison of the energies, the optimized geometries of the lowest‐energy conformer were subjected to a coupled cluster single point calculation (DLPNO‐CCSD(T)/aug‐cc‐cVQZ) using Orca 6.0.1.^[^ ^35^, ^36^ ^]^ For the tricyclic compounds **9** and **11**, the 2‐*exo*,3‐*exo*‐*cis*(2,3) was found to be the lowest in energy, while the energy separation to the next isomer higher in energy was less than 1 kcal mol^−1^. In the case of the bicyclic compounds **14**–**16**, the *cis*(2,3) isomer was the lowest in energy, while the energy separation to the trans isomer was in all cases less than 0.7 kcal/mol. These calculations are also in line with the experimental observations for most investigated compounds (**9**–**12**, **14**–**16**) (see Figure 3 for an excerpt, and Table S20 for the full dataset). A notable exception is the azepane‐derived **13**, where the *cis*‐configured isomer **13b** was lowest in energy, while experimentally, the *trans*‐configured isomers **13c** and **13d** were the major compounds observed after the cyclizing C─H insertion reaction.

Subsequently, experimentally collected ^13^C{^1^H} NMR spectroscopic data for the structurally characterized compounds **9a** and **14** were used in benchmarking the functional performance of a range of functionals (B3LYP, PBE0, TPSS0, TPSSh, ωB97, ωB97m‐V, DLPNO‐MP2, and PBE), in conjunction with Jensen's polarization consistent NMR shielding optimized basis set (pcSseg‐3). Mean‐absolute error (MAE) and Root Mean Squared Error (RMSE) analysis (Tables S4‐S5) pointed toward ωB97m‐V as the most reliable functional, which was further used throughout this study.

Lastly, all conformers within a 2.5 kcal mol^−1^ threshold for compounds **9**, **11**–**16** were subsequently subjected to NMR calculations at the ωB97m‐V/pcSseg‐3 + CPCM(chloroform) level of theory. Where applicable,^[^ ^37^ ^]^ a Boltzmann‐weighted analysis was employed to improve the accuracy of the calculated ^13^C NMR chemical shifts for the respective isomers (additional details are available in the SI) using the ANMR module.^[^ ^38^ ^]^

The calculated chemical shifts for each conformer ensemble were then compared to the experimental data collected for each isomer or isomer mixture. The numerical agreement between the calculated and experimental shifts was scrutinized with the statistical DP4 method pioneered by Smith and Goodman.^[^ ^39^ ^]^ This approach, which is commonly used in the structure elucidation of natural products and drug‐like compounds,^[^ ^40^, ^41^, ^42^, ^43^ ^]^ converts the individual |δ_calc_ – δ_exp_| differences for every carbon nucleus (the residuals) of each candidate stereoisomer into Bayesian posterior probabilities (DP4%), which sum to 100% and single out the most probable structure with quantitative confidence.^[^ ^44^ ^]^

**Figure 4:**
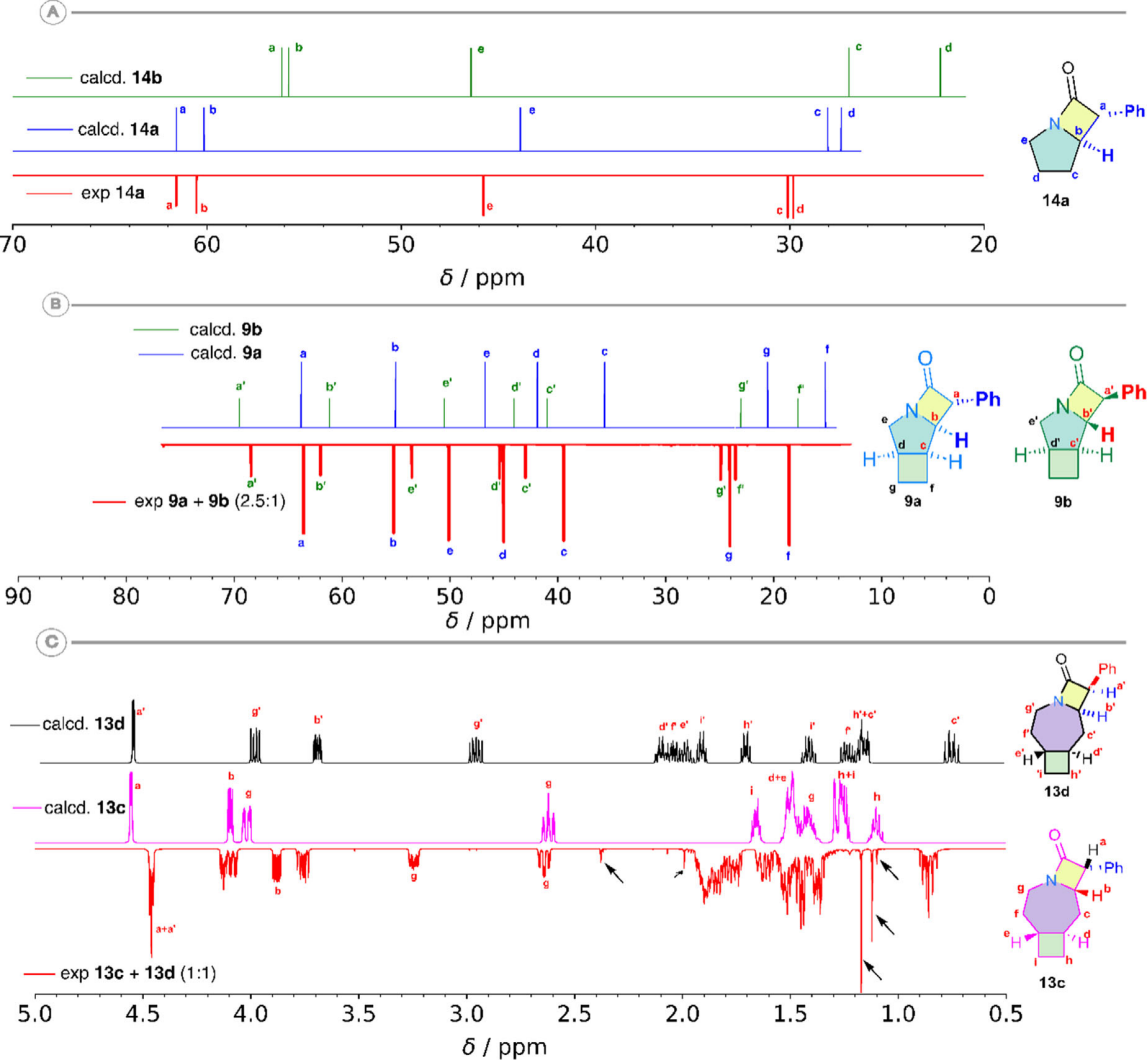
Comparison between experimental and calculated NMR spectra for synthesized bicyclic and tricyclic β‐lactams: a) Experimental ^13^C{^1^H} NMR spectrum of **14a** (400 MHz, C_6_D_6_, red, inverted) and the calculated Boltzmann‐averaged ^13^C NMR calculated of the likely isomer (blue, *cis*(2,3)) and unlikely isomer (green, *trans*(2,3)). b) Experimental ^13^C{^1^H} NMR spectrum of **9a** and **9b** (2.5:1 mixture, 600 MHz, CDCl_3_, red, inverted) and the calculated Boltzmann‐averaged ^13^C NMR spectrum of the major isomer (blue, *exo,exo*‐cis(2,3)) and minor isomer (green, *endo,endo*‐cis(2,3)). An empirically determined scaling of the chemical shifts (x0.92) was applied (see the SI). c) Experimental ^1^H NMR spectrum of **13c** and **13d** (1:1 mixture, 600 MHz, CDCl_3_, red, inverted) and the calculated ^1^H spectrum **13c** (*exo, endo‐trans*(2,3), magenta, and **13d** (*endo,exo‐trans*(2,3), black). Spin‐spin coupling on the Boltzmann‐averaged chemical shifts was calculated solving the spin Hamiltonian using the ANMR software.^[^ ^31^, ^32^ ^]^ No scaling was used for the calculated ^1^H spectra. All chemical shifts were calculated at a ωB97m‐V/pcSseg‐3 + CPCM(benzene) theory level. NMR plots were rendered using the nmrplot script.^[^ ^31^, ^32^ ^]^

For the bicyclic β‐lactams, DP4 analysis indicates that the *cis*(2,3) isomer is the major product formed, consistent with crystallographic data (for **14**), NOESY studies (for **14**–**16**), and literature precedence (compound **15**).^[^ ^23^ ^]^ In the case of the pyrrolidine‐ and piperidine‐based tricyclic β‐lactams (compounds **9**, **11**, and **12**), DP4 analysis suggests exclusive formation of the *cis*(2,3) species, with the *exo,exo* isomer (type **a**) as the dominant product and the *endo,endo* isomer (type **b**) as the minor one (Figure 4b). Where possible, this assignment was corroborated by crystallographic analysis (i.e. **9a**) and NOE studies (i.e. **10a** and **11a**). In the case of the azepane‐based **13**, which contains a *trans*‐configured cyclobutane ring, the C─H insertion reaction proceeded with a poorer selectivity, yielding all four possible diastereoisomers. Unlike the previous cases, the major species formed was the *trans*(2,3)‐isomer (85%), with the *exo,endo* (**13a**) and *endo,exo* (**13b**) species formed in equimolar amounts. The minor *cis*(2,3)‐isomer (**13a** and **13b**, *d.r*. 3:1) could be further identified and assigned by ^1^H NMR spectroscopy.

**Figure 5:**
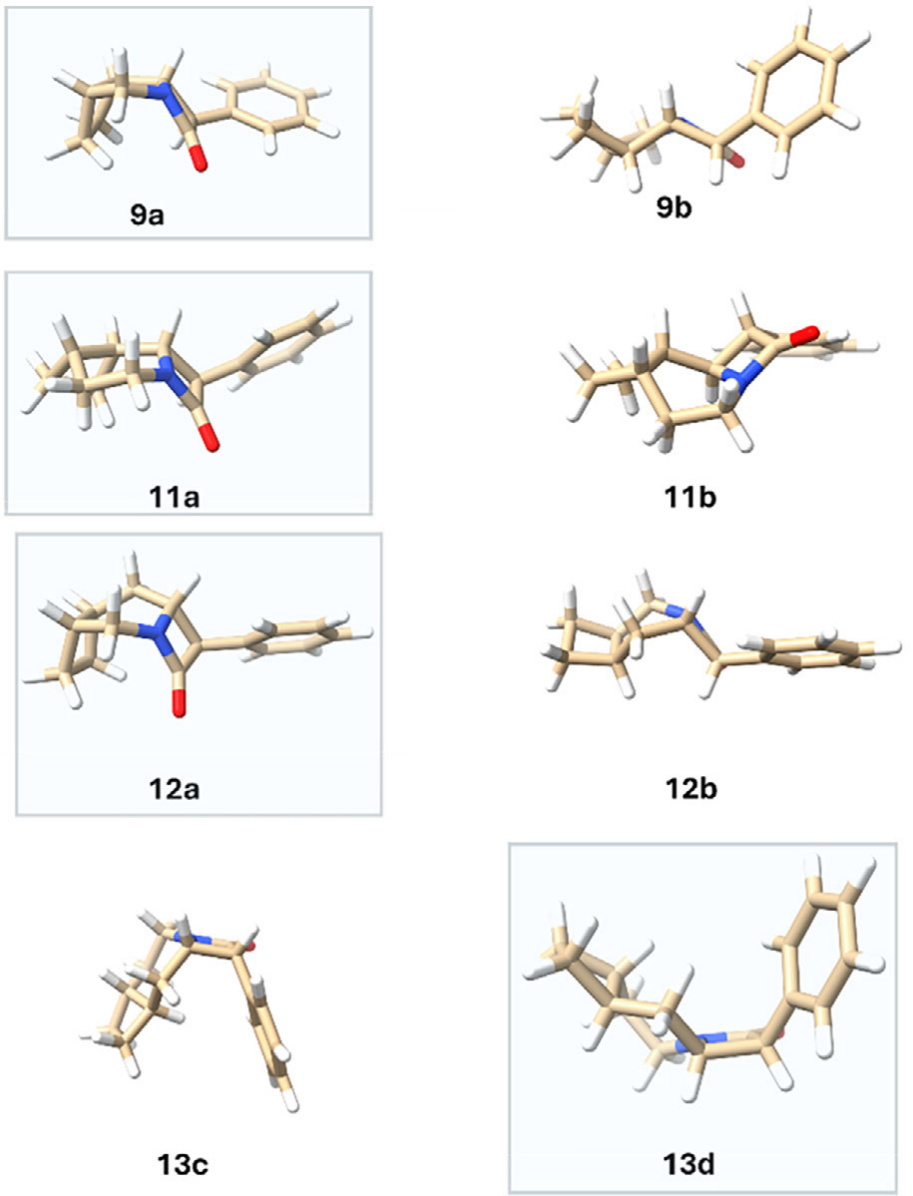
Calculated geometries of the major observed isomers for the tricylic β‐lactam species **9**–**13** (r^2^‐scan‐3c). Plots were rendered using the ChimeraX 1.10 software.^[^ ^45^ ^]^ For the structures of the bicyclic analogues, see Figure S31 (SI).

The calculated geometries of all the major species observed are given in Figure 5.

### 2.3 Attempts in Using Rh‐catalysed C─H Cyclization

**Scheme 3:**
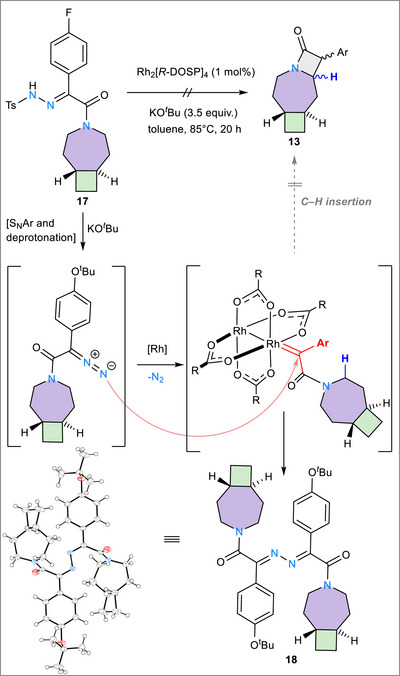
Attempts at rhodium‐mediated cyclizing C─H insertion from bicyclic azepane derivative, to yield the azo‐coupling product **18**, characterized by single crystal X‐ray diffraction. Thermal ellipsoids are set at 50% probability. Conditions: Rh_2_(*R*‐DOSP)_4_ 1 mol%, KO*^t^* Bu (3.7 equiv.), toluene, 85 °C, 18 h. NMR yield: 100%.

While attempting to improve the stereoselectivity for the synthesis of **13**, an alternative approach was tested. This relied on the in situ trapping of the generated carbene resulting from the diazo‐salt at a paddlewheel rhodium complex Rh_2_(OAc)_4_ or Rh_2_[*R*‐DOSP]_4_ [DOSP = Tetrakis[(*R*)‐(+)‐N‐(p‐dodecylphenylsulfonyl)prolinato)], where the ligand environment can exert control over the stereochemistry of the formed β‐lactam ring.^[^ ^46^, ^47^, ^48^, ^49^, ^50^, ^51^ ^]^ Our approach started from the hydrazone **17**, with the aim to use the *p*‐fluorophenyl functional group as an NMR probe for stereoselectivity (Scheme 3). Nevertheless, under these reaction conditions, the intramolecular C─H insertion/cyclization product **13** could not be detected. Instead, the azo‐coupling product **18** was exclusively detected and isolated, which could also be further confirmed by crystallographic studies (Scheme 3). The product distribution indicates that intramolecular C─H insertion is kinetically disfavored, allowing instead the *in‐situ*‐generated diazo salt to intercept the nascent Rh‐carbene. The KO*^t^* Bu used to deprotonate **17** to its diazo salt also acts as a nucleophile, triggering an S_N_Ar reaction on the *p*‐fluorophenyl ring. This side reaction is absent when Cs_2_CO_3_ is employed during the synthesis of **10** (Scheme 2), highlighting the superior functional‐group tolerance of the photochemical protocol. In addition, the electron‐donating p‐O*^t^* Bu substituent can further stabilize the Rh‐carbene complex through resonance,^[^ ^52^ ^]^ likely exacerbating the sluggishness of the desired C─H insertion step.

### 2.4 Ring Opening of β‐lactams and Synthesis of Methylphenidate Derivatives

**Scheme 4:**
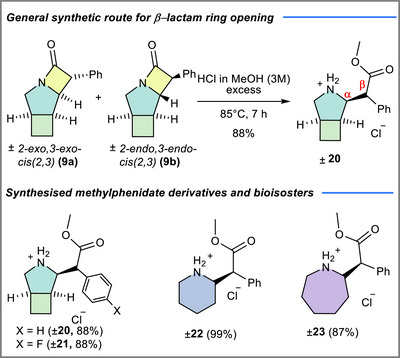
Acid mediated ring opening and synthesis of methylphenidate derivatives.

**Figure 6:**
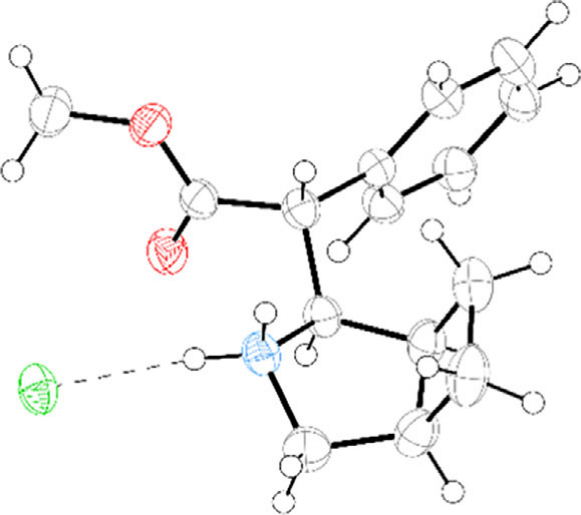
Molecular structure of **20**. Thermal Ellipsoids are displayed at 50% probability.^[^ ^61^ ^]^

Lastly, we sought to further explore the strain‐release reaction of the newly synthesized β‐lactam derivatives under acidic conditions, which gives rise to amino‐ester derivatives. Our interest was further fuelled by the well‐established bioactivity toward the central nervous system of methylphenidate derivatives such as **22**, which are currently commercialized as drugs against attention deficit hyperactivity disorder (ADHD).^[^ ^53^, ^54^, ^55^, ^56^, ^57^, ^58^, ^59^ ^]^ It was already established by Winkler et al.^[^ ^23^ ^]^ that piperazine‐based β‐lactams can be opened under acidic conditions (HCl, MeOH) to give the methylphenidate derivative **22** (Scheme 4). Applying a similar protocol,^[^ ^60^ ^]^ the pyrrolidine‐based tricyclic β‐lactams **20** and **21** yielded the corresponding methylphenidate derivatives in very good yields (88%). The structure of the bicyclic methylphenidate analogue **20** was confirmed by X‐ray diffraction (Figure 6), which showed that the compound crystallizes as a racemate. The β‐lactam ring opening generates a new stereocenter at C3 (the β‐carbon). Because the starting materials **9a** and **9b** were used as racemic mixtures, the product **20** is likewise obtained as a racemate.

The azepane derivative **23** was prepared in the same way, but the extended reaction times required for lactam acidolysis of **13** produced complex mixtures. Both unreacted starting material and the desired methylphenidate analogues were detectable; however, the target compounds could not be isolated in pure form.

## 3 Conclusion

We report the first synthesis of saturated cyclobutane‐fused tricyclic β‐lactams, achieved through an Fe‐catalysed intermolecular [2 + 2]‐cycloaddition of allyl amines followed by a mild, photochemical intramolecular C─H insertion of α‐diazo amides. The sequence delivers a family of pyrrolidine‐, piperidine‐, and azepane‐based frameworks with predictable *cis*(2,3) stereocontrol in the five‐ and six‐membered *N*‐heterocycles. Isomer assignment combined state‐of‐the‐art conformer sampling (CREST), modelling the NMR spectra using quantum‐chemical protocols and DP4‐probabilistic NMR analysis, corroborated by single‐crystal X‐ray diffraction. These studies revealed that the major products adopt conformationally locked, cage‐like architectures, which can offer enhanced control over the chemical space in drug design. Moreover, they can undergo strain‐release ring opening to furnish methylphenidate analogues in a single step.

Beyond providing practical access to scarcely explored, highly saturated polycyclic β‐lactams, this work showcases the interplay between earth‐abundant metal catalysis and photochemical carbene chemistry, and further highlights the growing power of modern computational‐spectroscopic workflows for deconvoluting isomer mixtures. We anticipate that the strategy will invigorate the search for conformationally restricted tricyclic β‐lactam scaffolds in drug discovery and broaden the application of iron‐mediated [2 + 2]‐cycloadditions in complex‐molecule synthesis.

## Supporting Information

The authors have cited additional references within the Supporting Information.^[^ ^62^, ^63^, ^64^, ^65^, ^66^, ^67^, ^68^, ^69^, ^70^, ^71^, ^72^, ^73^, ^74^, ^75^, ^76^, ^77^, ^78^, ^79^, ^80^, ^81^ ^]^ The Supporting Information includes the synthetic procedures for all new compounds, supplementary crystallographic details, computational details, and depictions of all the relevant NMR and IR spectra.

## Conflict of Interest

The authors declare no conflict of interest.

## Supporting information



Supporting Information

Supporting Information

Supporting Information

## Data Availability

The data that support the findings of this study are available in the supplementary material of this article.
