# Supplementary material for: Synthesis of Cyclobutane‐Containing Tricyclic β‐Lactams Based on a Saturated Scaffold Enabled by Iron‐catalysed [2 + 2]‐Cycloaddition
Source: Chemistry. 2025 Sep 26;31(64):e02476. doi: 10.1002/chem.202502476 (PMC12624322; doi:10.1002/chem.202502476)
Supplement: Supplementary file 1 — Supporting Information [file CHEM-31-e02476-s003.pdf]

Supporting Information

Synthesis of Cyclobutane-Containing Tricyclic β-Lactams Based on a Saturated Scaffold Enabled by Iron-catalysed [2+2]-cycloaddition

Lea Freitag[a,b], Johannes Zeh[a], Levi A. Ziegenhagen[a], Felix J. Becker[a], and Dragoș-Adrian Roșca*[,a,b]

[a]*Anorganisch-Chemisches Institut, Universität Heidelberg, Im Neuenheimer Feld 276, 69120 Heidelberg (Germany)*
E-Mail: dragos.rosca@uni-heidelberg.de
[b]*Institut des Sciences Chimiques, Université de Rennes, 263 Av. Général Leclerc, 35700 Rennes (France)*
E-Mail: dragos-adrian.rosca@univ-rennes.fr

Table of Contents

1 Synthetic Procedures **2**

2 Supporting Crystallographic Information **27**

3 Computational Details **31**

4 Spectra **61**

5 References **103**

General Remarks

^{Tric}PDI $FeCl_2$,[1] ($^{Ad}P^{CypPyr}$DI)$FeCl_2$,[2] (1R,5S)-3-azabicyclo[3.2.0]heptane ·HCl **3**,[2] (1R,6R)-3-azabicyclo[4.2.0]octane ·HCl **4**[2] and (1S,7S)-4-azabicyclo[5.2.0]nonane ·HCl **5**[2] were synthesised according to the literature procedures. Unless otherwise stated, all manipulations were performed using standard Schlenk techniques under dry argon in flame-dried glassware or in a nitrogen filled MBraun glovebox. Anhydrous solvents were freshly distilled from appropriate drying agents (CH_2Cl_2 and CH_3CN over CaH_2. THF, benzene, Et_2O, pentane over Na, MeOH over Mg) or dried over activated alumina columns (MBraun SPS 800 - Et_2O, hexane, pentane, toluene, methanol) and were transferred under argon or N_2. Unless otherwise stated, all reagents and solvents were purchased from Acros Organics, Alfa Aesar, Sigma-Aldrich, abcr GmbH, TCI Chemicals and used without further purification. Deuterated solvents were purchased from Sigma-Aldrich and Eurisotop .

^{1}H NMR/^{13}C{^{1}H}/^{19}F{^{1}H} NMR - spectra were recorded at 298 K in deuterated solvents either on BRUKER AVANCE III 600 (600.13 MHz / 150.93 MHz), BRUKER AVANCE II 400 (399.89 MHz / 101 MHz / 376 MHz) or Bruker Avance I 300 (300 MHz / 75 MHz / 282 MHz). ^{1}H NMR spectra were referenced to the residual protons of the deuterated solvent used. ^{13}C NMR spectra were referenced to the D-coupled ^{13}C resonances of the solvent. When needed, resonances were assigned using 2D NMR homo- and heterocorrelation techniques (HSQC, HMBC, COSY, NOESY). Coupling constants (J) are given in Hz, chemical shifts (δ) in ppm relative to TMS. Signal patterns are noted according to usual conventions (s = singlet, d = doublet, t = triplet, q = quartet, quin = quintet, sext = sextet, dd = doublet of doublet, dt = doublet of triplet, ddd = doublet of doublet of doublet, m = multiplet). Spectra were analysed using MestReNova 15.1.0.

High resolution mass spectra (HRMS) were performed by the mass spectroscopy facility of the Organisch-Chemisches Institut either on a Bruker Apex Qehybrid 9.4 T FT-ICR-MS or a Finnigan LCQ for positive electrospray ionisation (ESI+), or on a JEOL JMS-700 for electron ionisation (EI+). The mass is given dependent on ionic charge in m/z.

Analytical thin layer chromatography (TLC) were done on precoated TLC plates using Polygram Sil G/UV 254 plates from Macherey-Nagel Co. KG, Düren (Germany) and visualized by UV illumination (254 nm or 366 nm), staining with a Ninhydrin solution or *via* oxidation by potassium permangante solution.

Flash column chromatography was performed either on the Grace Reveleris Flash Chromatography System using pre-packed Flash Cartridges (FlashPure Büchi) or using silica gel from Sigma-Aldrich (particle size: 40-63 μm). For filtration Celite® 545, coarse, was used.

1 Synthetic Procedures

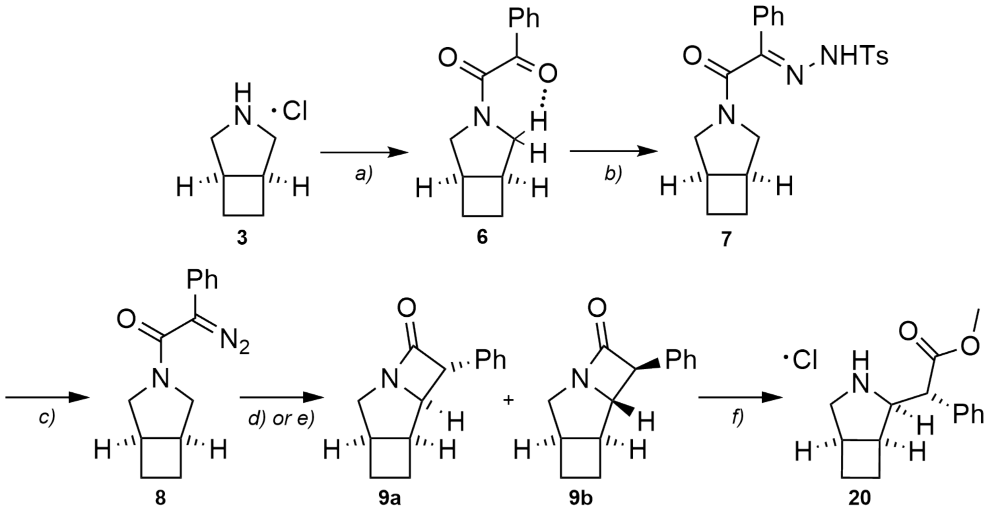

Figure S1: Conditions for the the synthesis of **9**: (a) pyridine (2.2 eq.), phenylglyoxalic acid (1.0 eq.), DMF (1.7 eq.), oxalyl chloride (1.5 eq.), CH_2Cl_2, 0 °C → RT, 16 h, 81 %; (b) tosyl hydrazide, H_2SO_4, EtOH, 85 °C, 16 h, 49 %; (c) NaH (1.0 eq.), DME, Aliquat® 336, RT → 60 °C, 4 h, 60 %, estimated purity 60 %; (d) Photochemical route: C_6H_6, hν 390 nm, 18 h, 47 %, **9a/9b** 3:1; e) Thermal route: LiHMDS or KO^{t}Bu, toluene, RT then 100 °C, 2 h, 69 %, **9a**:**9b** 3:1; f) HCl (3 M in MeOH), 85 °C, 7 h, 88 %.

1-((1*R*,5*S*)-3-azabicyclo[3.2.0]heptan-3-yl)-2-phenylethane-1,2-dione 6

Adapting a procedure by He *et al.*,[3] in a flame-dried Schlenk flask phenylglyoxylic acid (768 mg, 5.12 mmol, 1.0 eq.) was dissolved in anhydrous dichloromethane (15 mL). To this, DMF (0.86 mL, 8.7 mmol, 1.7 eq.) was added and the mixture was cooled to 0 °C. Oxalyl chloride (0.65 mL, 7.67 mmol, 1.5 eq.) was added dropwise and the reaction mixture was stirred at 0 °C for 30 min. With an external vacuum pump and cooling trap, the solvent was removed, resulting in the acid chloride as a viscous yellow liquid. In a second flame-dried Schlenk flask, (1*R*,5*S*)-3-azabicyclo[3.2.0]heptane ·HCl **3** (957 mg, 7.16 mmol, 1.4 eq.) and dry pyridine (0.86 mL, 11.25 mmol, 2.2 eq.) were dissolved in anhydrous dichloromethane (5 mL) and cooled to 0 °C. The acid chloride was redissolved in 5 mL anhydrous dichloromethane and added dropwise to the second flask under strong stirring. The mixture was warmed to room temperature and stirred overnight resulting in a dark red solution. Upon filtering the mixture through a silica plug (PE/EtOAc 3:2, 400 mL), the volatile components were removed *in vacuo*. The product **6** was used without further purification as a yellow oil (950 mg, 5.12 mmol, 81 %).

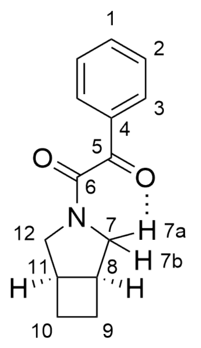

^{1}H NMR (600 MHz, $CDCl_3$) δ [ppm] = 8.03 (d, J = 8.6 Hz, 2H, H-3), 7.65 (t, J = 7.4 Hz, 1H, H-1), 7.52 (t, J = 7.7 Hz, 2H, H-2), 4.11 (d, J = 12.8 Hz, 1H, H-7a), 3.49 – 3.37 (m, 3H, H-7b, H-12), 3.05 – 3.00 (m, 1H, H-8), 3.00 – 2.94 (m, 1H, H-11), 2.34 – 2.25 (m, 1H, H-9), 2.24 – 2.14 (m, 1H, H-10), 1.81 – 1.72 (m, 1H, H-9), 1.69 – 1.58 (m, 1H, H-10).
^{13}C{^{1}H} NMR (151 MHz, $CDCl_3$) δ [ppm] = 191.8 (C_q, C-5), 165.7 (C_q, C-6), 134.8 (CH, C-1), 133.2 (C_q, C-4), 130.0 (CH, C-3), 129.1 (CH, C-2), 53.8 (CH_2, C-12), 51.8 (CH_2, C-7), 37.9 (CH, C-8), 36.5 (CH, C-8), 24.6 (CH_2, C-9), 24.0, (CH_2, C-9).
HRMS (EI+): m/z for $C_{14}H_{15}NO_2^+$ [M^+] calcd: 229.1097, found: 229.1120.

N'-((*E*)-2-((1*S*,6*S*)-3-azabicyclo[4.2.0]octan-3-yl)-2-oxo-1-phenylethylidene)-tosylhydrazide 7

Adapting aprocedure by Lapinsky *et al.*,[4] tosyl hydrazide (926 mg, 4.97 mmol, 1.2 eq.) was dissolved in EtOH (25 mL). To this suspension H_2SO_4 (1 mL) was added, until everything had dissolved. Upon addition of 1-((1*R*,5*S*)-3-azabicyclo[3.2.0]heptan-3-yl)-2-phenylethane-1,2-dione **6** (950 mg, 4.14 mmol, 1.0 eq.), the reaction mixture was stirred at 85 °C overnight. The crude product was purified *via* flash column chromatography (SiO_2, PE/EtOAc 3:2) affording the product **7** as a colourless solid (730 mg, 1.84 mmol, 49 %).

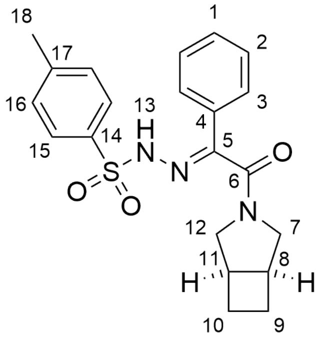

^{1}H NMR (600 MHz, $CDCl_3$) δ [ppm] = 8.45 (s, 1H, H-13), 7.87 (d, J = 8.4 Hz, 2H, H-15), 7.64 (dd, J = 7.7, 2.0 Hz, 2H, H-3), 7.42 – 7.35 (m, 3H, H-1, H-2), 7.30 (d, J = 9.4 Hz, 2H, H-16), 4.10 (d, J = 12.9 Hz, 1H, H-7/H-12), 3.46 – 3.40 (m, 1H, H-7/H-12), 3.22 – 3.19 (m, 2H, H-7/H-12), 3.05 – 2.97 (m, 1H, H-8/H-11), 2.94 – 2.86 (m, 1H, H-8/H-11), 2.40 (s, 3H, H-17/H-20), 2.33 – 2.23 (m, 1H, H-9/H-10), 2.22 – 2.12 (m, 1H, H-9/H-10), 1.79 – 1.69 (m, 1H, H-9/H-10), 1.65 – 1.56 (m, 1H, H-9/H-10).
^{13}C{^{1}H} NMR (151 MHz, $CDCl_3$) δ [ppm] = 162.6 (C_q, C-6), 151.1 (C_q, C-5), 144.5 (C_q, C-14), 135.2 (C_q, C-17), 132.1 (C_q, C-4), 130.8 (CH, C-1), 129.8 (CH, C-16), 129.1 (CH, C-2), 128.2 (CH, C-15), 126.5 (CH, C-3), 54.3 (CH_2, C-7/C-12), 51.9 (CH_2, C-7/C-12), 37.5 (CH_2, C-8/C-11), 36.6 (CH_2, C-8/C-11), 24.6 (CH_2, C-9/C-10), 24.1 (CH_2, C-9/C-10), 21.8 (CH_3, C-18).
HRMS (EI+): m/z for $C_{14}H_{16}NO^+$ [$M-TSN_2^+$] calcd: 214.1226, found: 214.1255.

1-((1*R*,5*S*)-3-azabicyclo[3.2.0]heptan-3-yl)-2-diazo-2-phenylethan-1-one 8

Adapting a procedure of Corey *et al.*,[5] a flame-dried Schlenk flask was charged with NaH (30.2 mg, 1.26 mmol, 1.0 eq.) and dry dimethoxyethane (13 mL). To this a suspension of *N*'-(2-(3-azabicyclo[3.2.0]heptan-3-yl)-2-oxo-1-phenylethylidene)-tosylhydrazide **7** (500 mg,

1.26 mmol, 1.0 eq.) and Aliquat® 336 (3 drops) was added. The reaction mixture was stirred at room temperature until formation of H_2-gas ceased (20 min), then heated to 60 °C and stirred for 4 h. The initially pale yellow and clear solution changed to a bright orange solution with a colourless precipitate. The reaction mixture was cooled to room temperature, filtered over Celite®, eluted with Et_2O and removed the solvent *in vacuo*. The product **8** is prone to decomposition and was used without further purification with an estimated purity of 60 % (216 mg (w %), 890 μmol, 71 %).

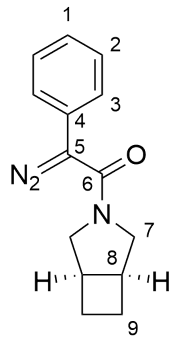

^{1}H NMR (600 MHz, $CDCl_3$) δ [ppm] = 7.40 – 7.36 (m, 2H, H-3), 7.34 – 7.30 (m, 2H, H-2), 7.18 (t, J = 7.4 Hz, 1H, H-1), 3.72 (d, J = 11.1 Hz, 2H, H-7), 3.33 – 3.27 (m, 2H, H-7), 2.96 – 2.91 (m, 2H, H-8), 2.22 – 2.17 (m, 2H, H-9), 1.73 – 1.66 (m, 2H, H-9).
^{13}C{^{1}H} NMR (151 MHz, $CDCl_3$) δ [ppm] = 174.8 (C_q, C-5), 164.6 (C_q, C-6), 129.2 (CH, C-3), 127.7 (C_q, C-4), 126.0 (CH, C-1), 125.3 (CH, C-2), 54.2 (CH_2, C-7), 37.4 (CH, C-8), 24.3 (CH_2, C-9).
HRMS (EI+): m/z for $C_7H_{10}NO^+$ [$(M-C_7H_5N_2)^+$] calcd: 124.0757, found: 124.0758.
IR (ATR): $\tilde{\nu}(N_2) = 2071\ cm^{-1}$.

(1*S*,7*R*)-3-phenyl-5-azatricyclo[5.2.0.0^{2,5}]nonan-4-one 9

Protocol A

Adapting a procedure of Corey *et al.*,[5] a vial was charged with 1-((1*R*,5*S*)-3-azabicyclo[3.2.0] heptan-3-yl)-2-diazo-2-phenylethan-1-one **8** (192 mg, 800 μmol, 1.0 eq.) and evacuated and backfilled with argon three times. Anhydrous benzene (5 mL) was added and the reaction mixture was irradiated by a 390 nm LED light for 18 h. The crude product was purified *via* flash column chromatography (SiO_2, PE/EtOAc 2:1). The reaction yields a mixture of two inseparable diastereomers in a ratio of **9a/9b** = 3:1, as a yellow oil (80 mg, 380 μmol, 47 %). The data for each isomer are given below.

Protocol B

In a flame dried ampoule hydrazone **7** (48.7 mg, 123 μmol, 1.00 eq.) was dissolved in anhydrous toluene (1 mL). A solution of LiHMDS (21.5 mg, 129 μmol, 1.05 eq.) in anhydrous toluene (1.5 mL) was added dropwise, while the solution turned deep orange. The reaction mixture was refluxed at 100 °C for 2 h, while the reaction mixture turned almost colourless and a colourless precipitate was formed. Afterwards the suspension was diluted with toluene (10 mL) and washed with water (2-5 mL). The aqueous phase was again extracted with toluene (2-5 mL). The combined organic phase was washed with brine (10 mL), dried over $MgSO_4$ and the solvent was removed under reduced pressure. For further purification the product was filtrated through a

silica plug (PE/EE 2:1). The reaction yields a mixture of two inseparable diastereomers in a ratio of **9a/9b** = 3:1, as a yellow oil (17.9 mg, 83.9 μmol, 69 %). The data for each isomer are given below.

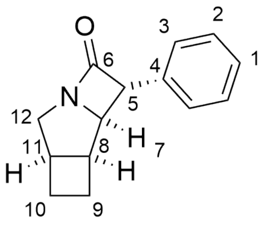

9a

^{1}H NMR (600 MHz, $CDCl_3$) δ [ppm] = 7.38 – 7.30 (m, 4H, H-2, H-3), 7.29 – 7.27 (m, 1H, H-1), 4.17 (d, J = 1.9 Hz, 1H, H-5), 3.72 (dd, J = 6.8, 1.9 Hz, 1H, H-7), 3.68 (dd, J = 12.4, 2.1 Hz, 1H, H-12), 3.30 – 3.22 (m, 1H, H-11), 3.14 (p, 1H, H-8), 2.97 (dd, J = 12.3, 7.5 Hz, 1H, H-12), 2.36 – 2.26 (m, 1H, H-10), 2.23 – 2.13 (m, 1H, H-9), 2.14 – 2.05 (m, 1H, H-9), 1.76 – 1.67 (m, 1H, H-10).

^{13}C{^{1}H} NMR (151 MHz, $CDCl_3$) δ [ppm] = 174.8 (C_q, C-6), 136.1 (C_q, C-4), 129.0 (CH, C-2), 127.7 (CH, C-3), 127.5 (CH, C-1), 63.9 (CH, C-7), 55.6 (CH, C-5), 50.5 (CH_2, C-12), 45.4 (CH, C-11), 39.8 (CH, C-8), 24.4 (CH_2, C-10), 18.9 (CH_2, C-9).

HRMS (ESI+): m/z for $C_{14}H_{22}NNaO^+$ [$(M+Na)^+$] calcd: 236.1046, found: 236.1047.

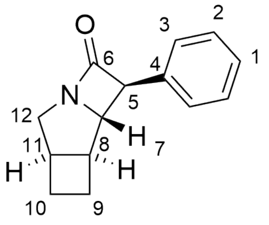

9b

^{1}H NMR (600 MHz, $CDCl_3$) δ [ppm] = 7.33 – 7.30 (m, 4H, H-2, H-3), 7.29 – 7.28 (m, 1H, H-1), 4.07 (dd, J = 12.0, 8.2 Hz, 1H, H-12), 3.95 (d, J = 2.5 Hz, 1H, H-5), 3.80 (t, J = 2.9 Hz, 1H, H-7), 3.34 – 3.29 (m, 1H, H-11), 2.94 – 2.89 (m, 1H, H-8), 2.87 (dd, J = 12.0, 4.3 Hz, 1H, H-12), 2.35 – 2.32 (m, 1H, H-9), 2.28 – 2.25 (m, 1H, H-10), 1.91 – 1.85 (m, 1H, H-9), 1.85 – 1.79 (m, 1H, H-10).

^{13}C{^{1}H} NMR (151 MHz, $CDCl_3$) δ [ppm] = 176.3 (C_q, C-6), 135.9 (C_q, C-4), 129.0 (CH, C-2), 127.6 (CH, C-3), 127.5 (CH, C-1), 68.8 (CH, C-7), 62.4 (CH, C-5), 53.9 (CH_2, C-12), 45.8 (CH, C-11), 43.4 (CH, C-8), 25.3 (CH_2, C-10), 23.9 (CH_2, C-9).

Methyl 2-((1*R*,5*S*)-3-azabicyclo[3.2.0]heptan-2-yl)-2-phenylacetate hydrochloride 20

Adapting a procedure of Lapinsky *et al.*,[4] a Schlenk flask was charged with (1*S*,7*R*)-3-phenyl-5-azatricyclo[5.2.0.0^{2,5}]nonan-4-one **9** (60 mg, 279 μmol, 1.0 eq.) and evacuated and backfilled with argon three times. To this, 3 M HCl in MeOH (12 mL) was added and the reaction mixture was stirred at 85 °C for 7 h. Upon cooling to room temperature, the volatile components were removed *in vacuo*. The crude product was washed with EtOAc (3x10mL) and pentane (5x10 mL) and dried under reduced pressure. The product **20** was obtained as a colourless crystalline solid (60 mg, 245 μmol, 88 %).

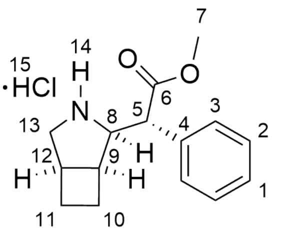

^{1}H NMR (600 MHz, $CDCl_3$) δ [ppm] = 11.0 (bs, 1H, H-14, H-15), 9.51 (s, 1H, H-14/H-15), 7.36 – 7.29 (m, 3H, H-1, H-2), 7.24 – 7.18 (m, 2H, H-3), 4.54 (d, J = 11.4 Hz, 1H, H-5), 4.16 – 4.05 (m, 1H, H-8), 3.81 (s, 3H, H-7), 3.78 – 3.74 (m, 1H, H-13), 3.57 – 3.44 (m, 1H, H-13),

2.99 – 2.92 (m, 1H, H-12), 2.74 – 2.66 (m, 1H, H-9), 2.65 – 2.58 (m, 1H, H-10), 2.28 – 2.17 (m, 1H, H-11), 2.13 – 2.05 (m, 1H, H-11), 1.89 – 1.80 (m, 1H, H-10).

^{13}C{^{1}H} NMR (151 MHz, $CDCl_3$) δ [ppm] = 172.1 (C_q, C-6), 135.4 (C_q, C-4), 129.3 (CH, C-2), 128.4 (CH, C-1), 128.1 (CH, C-3), 65.2 (CH, C-8), 53.4 (CH_3, C-7), 53.2 (CH_2, C-13), 49.7 (CH, C-5), 39.6 (CH, C-9), 35.2 (CH, C-12), 23.2 (CH_2, C-11), 18.4 (CH_2, C-10).

HRMS (EI+): m/z for $C_{15}H_{17}NO_2^+$ [$(M\text{-}H_2)^+$] calcd: 243.1254, found: 243.1253.

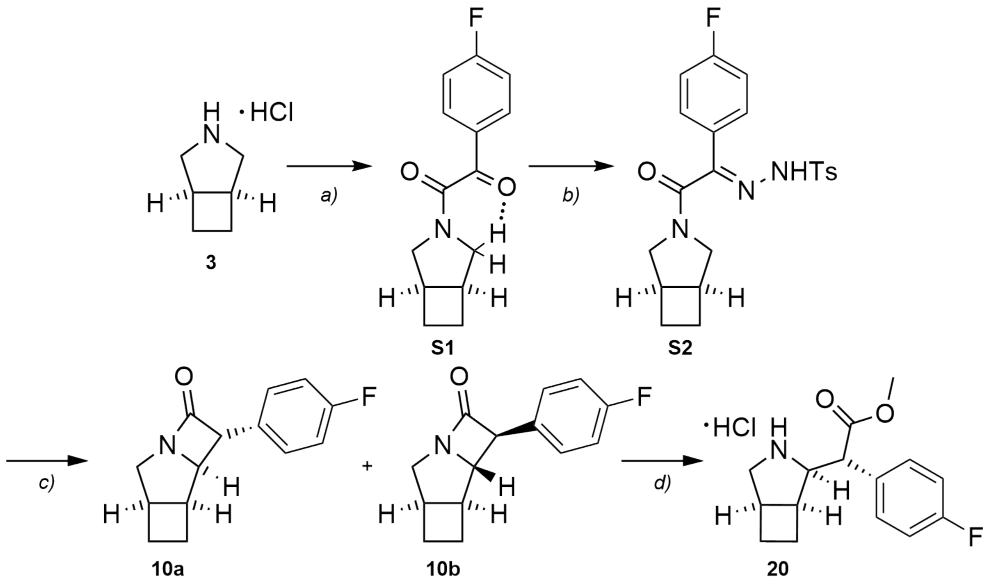

Figure S2: Conditions for the the synthesis of **10**: (a) pyridine (2.2 eq.), phenylglyoxalic acid (1.0 eq.), DMF (1.7 eq.), oxalyl chloride (1.5 eq.), CH_2Cl_2, 0 °C → RT, 16 h, 47 %; (b) tosyl hydrazide, H_2SO_4, EtOH, 85 °C, 16 h, 54 %; (c) Cs_2CO_3 (1 eq.), MeCN, hν 365 nm, 72 h, 52 %, **10a/10b** 2.5:1; d) HCl (2 M in Et_2O), MeOH, 55 °C, 7 h, 88 %.

1-((1*R*,5*S*)-3-azabicyclo[3.2.0]heptan-3-yl)-2-(4-fluorophenyl)ethane-1,2-dione S1

Adapting a procedure by He *et al.*,[3] in a flame-dried Schlenk flask phenylglyoxylic acid (512 mg, 3.05 mmol, 1.0 eq.) was dissolved in anhydrous dichloromethane (15 mL). To this, DMF (0.51 mL, 5.18 mmol, 1.7 eq.) was added and the mixture was cooled to 0 °C. Oxalyl chloride (0.39 mL, 4.57 mmol, 1.5 eq.) was added dropwise and the reaction mixture was stirred at 0 °C for 30 min. Using two additional cooling traps, the solvent was removed under inert conditions, resulting in the acid chloride as a viscous yellow liquid. In a second flame-dried Schlenk flask, (1*R*,5*S*)-3-azabicyclo[3.2.0]heptane ·HCl **3** (570 mg, 4.27 mmol, 1.4 eq.) and dry pyridine (0.51 mL, 6.70 mmol, 2.2 eq.) were dissolved in anhydrous dichloromethane (3 mL) and cooled to 0 °C. The acid chloride was redissolved in 3 mL anhydrous dichloromethane and added dropwise to the second flask under strong stirring. The mixture was warmed to room temperature and stirred overnight resulting in a dark red solution. Upon filtering the mixture through a silica plug (PE/EtOAc 3:2, 400 mL), the volatile components were removed *in vacuo*. The product **S1** was used without further purification as a yellow oil (350 mg, 1.42 mmol, 47 %).

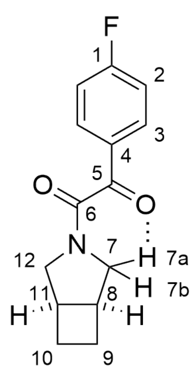

^{1}**H NMR** (400 MHz, $CDCl_3$) δ [ppm] = 8.15 – 7.89 (m, 2H, H-3), 7.19 (t, J = 8.6 Hz, 2H, H-2), 4.10 (d, J = 11.5 Hz, 1H, H-7a), 3.49 – 3.31 (m, 3H, H-7b, H-12), 3.09 – 2.93 (m, 2H, H-8/H-11), 2.37 – 2.26 (m, 1H, H-9), 2.26 – 2.15 (m, 1H, H-10), 1.81 – 1.70 (m, 1H, H-9), 1.69 – 1.57 (m, 1H, H-10).

^{13}**C{^{1}H} NMR** (101 MHz, $CDCl_3$) δ [ppm] = 190.1 (C_q, C-5), 166.8 (C_q, $^1J_{C-F}$ = 259.1 Hz, C-1) 165.3 (C_q, C-6), 132.8 (CH, $^3J_{C-F}$ = 9.7 Hz, C-3), 129.8 (C_q, $^4J_{C-F}$ = 2.7 Hz, C-4), 116.4 (CH, $^2J_{C-F}$ = 22.2 Hz, C-2), 53.9 (CH_2, C-12), 51.9 (CH_2, C-7), 37.9 (CH, C-8/C-11), 36.5 (CH, C-8/C-11), 24.7 (CH_2, C-9), 24.1 (CH_2, C-10).

^{19}**F{^{1}H}** (282 MHz, $CDCl_3$) δ [ppm] = -101.96.

HRMS (EI+): m/z for $C_{14}H_{14}NO_2F^+$ [M^+] calcd: 247.1004, found: 247.1006.

N'-(2-(3-azabicyclo[3.2.0]heptan-3-yl)-1-(4-fluorophenyl)-2-oxoethylidene)-tosylhydrazide S2

Adapting a procedure by Lapinsky *et al.*,[4] tosyl hydrazide (316 mg, 1.7 mmol, 1.2 eq.) was dissolved in EtOH (8 mL). To this suspension H_2SO_4 (1 mL) was added, until everything had dissolved. Upon addition of 1-((1*R*,5*S*)-3-azabicyclo[3.2.0]heptan-3-yl)-2-(4-fluorophenyl)ethane-1,2-dione **S1** (350 mg, 1.42 mmol, 1.0 eq.), the reaction mixture was stirred at 85 °C overnight. After cooling to room temperature, the mixture was cooled to -20 °C overnight in order to facilitate crystallisation. The resulting precipitate was filtered off and dried *in vacuo* affording the product **S2** as a colourless crystalline solid (320 mg, 770 μmol, 54 %).

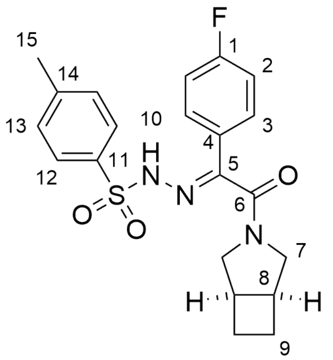

^{1}**H NMR** (300 MHz, $CDCl_3$) δ [ppm] = 8.39 (s, 1H, H-13), 7.86 (d, J = 8.4 Hz, 2H, H-15), 7.66 – 7.59 (m, 2H, H-3), 7.30 (d, J = 8.6 Hz, 2H, H-16), 7.07 (t, J = 8.7 Hz, 2H, H-2), 4.09 (d, J = 13.0 Hz, 1H, H-7/H-12), 3.42 (dd, J = 13.1, 7.4 Hz, 1H, H-7/H-12), 3.24 – 3.16 (m, 2H, H-7/H-12), 3.07 – 2.97 (m, 1H, H-8/H-11), 2.96 – 2.85 (m, 1H, H-8/H-11), 2.41 (s, 3H, H-18), 2.36 – 2.12 (m, 2H, H-9/H-10), 1.81 – 1.68 (m, 1H, H-9/H-10), 1.66 – 1.57 (m, 1H, H-9/H-10).

^{13}**C{^{1}H} NMR** (75 MHz, $CDCl_3$) δ [ppm] = 163.2 (C_q, C-1), 162.4 (C_q, C-6), 152.9 (C_q, C-5), 144.5 (C_q, C-14), 135.6 (C_q, C-17), 131.6 (C_q, C-4), 129.9 (CH, C-16), 128.7 (CH, C-3), 128.2 (CH, C-15), 116.4 (CH, C-2), 54.3 (CH_2, C-7/C-12), 52.0 (CH_2, C-7/C-12), 37.6 (CH, C-8/C-11), 36.6 (CH, C-8/C-11), 24.7 (CH_2, C-9/C-10), 24.1 (CH_2, C-9/C-10), 21.7 (CH_3, C-18). Due to low intensity of the spectrum, J_{C-F} coupling could not be accurately determined.

^{19}**F{^{1}H}** (282 MHz, $CDCl_3$) δ [ppm] = -108.87.

HRMS (EI+): m/z for $C_{14}H_{15}FNO^+$ [$(M-TsN_2)^+$] calcd: 232.1137, found: 232.1147.

(1*R*,7*S*)-3-(4-fluorophenyl)-5-azatricyclo[5.2.0.0^{2,5}]nonan-4-one 10

Adapting a procedure of König *et al.*,[6] an oven dried Schlenk tube was charged with *N*'-(2-(3-azabicyclo[3.2.0]heptan-3-yl)-1-(4-fluorophenyl)-2-oxoethylidene)-tosylhydrazide **S2** (260 mg, 625 μmol, 1.0 eq.) and Cs_2CO_3 (204mg, 625 μmol, 1.0 eq.) and evacuated and backfilled with argon three times. Anhydrous acetonitrile (8 mL) was added and the mixture was subsequently irradiated at 365 nm while stirring vigorously for 72 h until the tosylhydrazone was fully consumed. The crude product was adsorbed onto Celite®, filtered over a silica plug (SiO_2, PE/EtOAc 1:1, 200 mL) and further purified *via* flash column chromatography (SiO_2, PE/EtOAc 4:1 $\rightarrow$ 5:2 $\rightarrow$ 1:3). The reaction yields a mixture of two inseparable diastereomers in a ratio of **10a/10b** = 2.5:1 as a colourless crystalline solid (75 mg, 324 μmol, 52 %). The data for each isomer are given below.

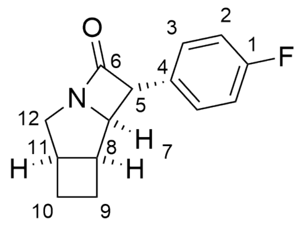

10a

^{1}**H NMR** (400 MHz, $CDCl_3$) δ [ppm] = 7.34 – 7.26 (m, 2H, H-3), 7.03 (t, J = 8.7 Hz, 2H, H-2), 4.14 (d, J = 2.1 Hz, 1H, H-5), 3.69 (t, J = 1.9 Hz, 1H, H-7), 3.66 (dd, J = 3.9, 2.1 Hz, 1H, H-12), 3.28 – 3.23 (m, 1H, H-11), 3.14 (p, 1H. H-8), 2.97 (dd, J = 12.5, 7.5 Hz, 1H, H-12), 2.35 – 2.27 (m, 1H, H-10), 2.23 – 2.14 (m, 1H, H-9), 2.12 – 2.02 (m, 1H, H-9), 1.76 – 1.63 (m, 1H, H-10).

^{13}**C{^{1}H} NMR** (101 MHz, $CDCl_3$) δ [ppm] = 174.5 (C_q, C-6), 162.3 (C_q, $^1J_{C-F}$ = 245.8 Hz, C-1), 132.0 (C_q, $^4J_{C-F}$ = 3.2 Hz, C-4), 129.3 (CH, $^3J_{C-F}$ = 8.1 Hz, C-3), 115.8 (CH, $^2J_{C-F}$ = 21.2 Hz, C-2), 64.1 (CH, C-7), 54.8 (CH, C-5), 50.5 (CH_2, C-12), 45.4 (CH, C-11), 39.8 (CH, C-8), 24.4 (CH_2, C-10), 18.9 (CH_2, C-9).

^{19}**F{^{1}H}** (376 MHz, $CDCl_3$) δ [ppm] = -115.16.

HRMS (EI+): m/z for $C_{14}H_{14}FNO^+$ [M^+] calcd: 231.1054, found: 231.1054.

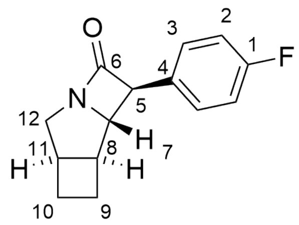

10b

^{1}**H NMR** (400 MHz, $CDCl_3$) δ [ppm] = 7.29 – 7.27 (m, 2H, H-3), 7.03 (t, J = 8.0 Hz, 2H, H-2), 4.07 (dd, J = 12.0, 8.1 Hz, 1H, H-12), 3.92 (d, J = 2.4 Hz, 1H, H-5), 3.76 (dd, J = 3.3, 2.4 Hz, 1H, H-7), 3.35 – 3.29 (m, 1H, H-11), 2.93 – 2.88 (m, 1H, H-8), 2.86 (dd, J = 12.1, 4.4 Hz, 1H, H-12), 2.38 – 2.33 (m, 1H, H-9), 2.29 – 2.26 (m, 1H, H-10), 1.93 – 1.86 (m, 1H, H-9), 1.85 – 1.77 (m, 1H, H-10).

^{13}**C{^{1}H} NMR** (101 MHz, $CDCl_3$) δ [ppm] = 176.0 (C_q, C-6), 162.3 (C_q, $^1J_{C-F}$ = 245.4 Hz, C-1), 131.4 (C_q, $^4J_{C-F}$ = 3.0 Hz, C-4), 129.1 (CH, $^3J_{C-F}$ = 8.1 Hz, C-3), 115.9 (CH, $^2J_{C-F}$ = 21.6 Hz, C-2), 68.9 (CH, C-7), 61.6 (CH, C-5), 53.9 (CH_2, C-12), 45.8 (CH, C-11), 43.4 (CH, C-8), 25.3 (CH_2, C-10), 23.9 (CH_2, C-9).

^{19}**F{^{1}H}** (376 MHz, $CDCl_3$) δ [ppm] = -115.00.

Methyl 2-((1*R*,5*S*)-3-azabicyclo[3.2.0]heptan-2-yl)-2-(4-fluorophenyl)acetate hydrochloride 21

Adapting a procedure of Lapinsky *et al.*,[4] a Schlenk flask was charged with β-lactam (1*R*,7*S*)-3-(4-fluorophenyl)-5-azatricyclo[5.2.0.0^{2,5}]nonan-4-one **10** (35 mg, 151 μmol, 1.0 eq.) and evacuated and backfilled with argon three times. To this, dry MeOH (8 mL) and 2 M HCl in Et_2O (11 mL) was added and the reaction mixture was stirred at 55 °C for 7 h. Upon cooling to room temperature, the volatile components were removed *in vacuo*. The crude product was washed with EtOAc (3x15mL) and pentane (5x20 mL) and dried under reduced pressure. The product **21** was obtained as a colourless crystalline solid (40 mg, 133 μmol, 88 %).

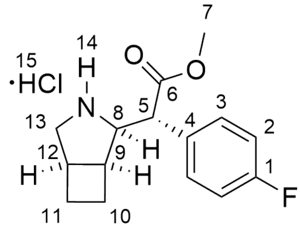

^{1}H NMR (400 MHz, $CDCl_3$) δ [ppm] = 10.89 (s, 1H, H-14/H-15), 9.78 (s, 1H, H-14/H-15), 7.24 – 7.19 (m, 2H, H-3), 7.09 – 6.98 (m, 2H, H-2), 4.55 (s, 1H, H-5), 4.11 – 4.10 (m, 1H, H-8), 3.83 (s, 3H, H-7), 3.54 – 3.53 (m, 2H, H-13), 3.00 – 2.95 (m, 1H, H-12), 2.75 – 2.70 (m, 1H, H-9), 2.59 – 2.54 (m, 1H, H-10), 2.25 – 2.21 (m, 1H, H-11), 2.10 – 2.06 (m, 1H, H-11), 1.83 – 1.81 (m, 1H, H-10).

^{13}C{^{1}H} NMR (151 MHz, $CDCl_3$) δ [ppm] = 171.9 (C_q, C-6), 161.4 (C_q, C-1), 140.5 (C_q, C-4), 129.9 (CH, C-3), 116.2 (CH, C-2), 65.3 (CH, C-8), 53.7 (CH_3, C-7), 51.1 (CH_2, C-13), 49.0 (CH, C-5), 39.7 (CH, C-9), 35.3 (CH, C-12), 23.3 (CH_2, C-11), 18.3 (CH_2, C-10). Due to low intensity of the spectrum, J_{C-F} coupling could not be accurately determined.

^{19}F{^{1}H} (376 MHz, $CDCl_3$) δ [ppm] = -133.21.

HRMS (EI+): m/z for $C_{15}H_{16}FNO_2^+$ [$(M-H_2)^+$] calcd: 261.1160, found: 261.1150.

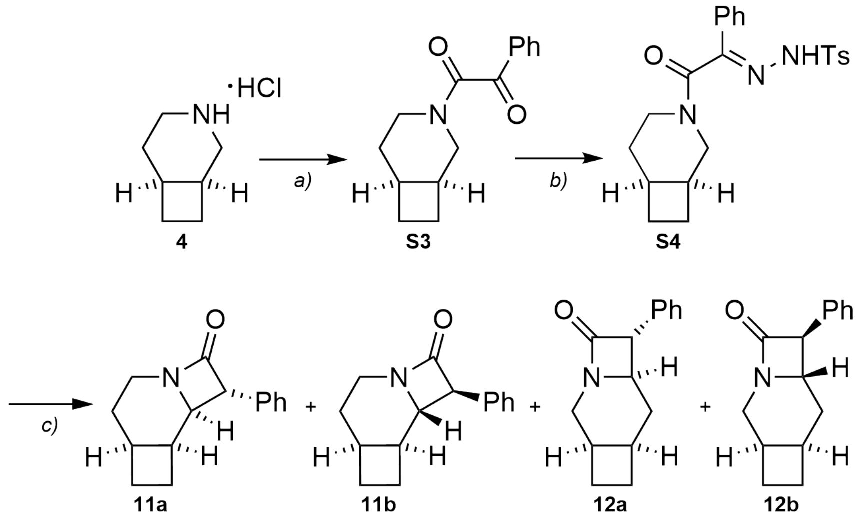

Figure S3: Conditions for the the synthesis of **11** and **12**: (a) pyridine (2.2 eq.), phenylglyoxalic acid (1.0 eq.), DMF (1.7 eq.), oxalyl chloride (1.5 eq.), CH_2Cl_2, 0 °C → RT, 16 h, 52 %; (b) tosyl hydrazide, H_2SO_4, EtOH, 85 °C, 16 h, 43 %; (c) Cs_2CO_3 (1 eq.), CH_2Cl_2, hν 365 nm, 24 h, 70 %, **11/12** 1.6:1, **11a/11b** 1.4:1, **12a/12b** 1.8:1.

1-((1*S*,6*S*)-3-azabicyclo[4.2.0]octan-3-yl)-2-phenylethane-1,2-dione S3

Adapting a procedure by He *et al.*,[3] in a flame-dried Schlenk flask phenylglyoxylic acid (323 mg, 2.15 mmol, 1.0 eq.) was dissolved in anhydrous dichloromethane (7 mL). To this, DMF (0.35 mL, 3.66 mmol, 1.7 eq.) was added and the mixture was cooled to 0 °C. Oxalyl chloride (0.27 mL, 3.23 mmol, 1.5 eq.) was added dropwise and the reaction mixture was stirred at 0 °C for 30 min. Using two additional cooling traps, the solvent was removed under inert conditions, resulting in the acid chloride as a viscous yellow liquid. In a second flame-dried Schlenk flask, (1*R*,6*R*)-3-azabicyclo[4.2.0]octane ·HCl **4** (445 mg, 3.01 mmol, 1.4 eq.) and dry pyridine (0.35 mL, 4.74 mmol, 2.2 eq.) were dissolved in anhydrous dichloromethane (2 mL) and cooled to 0 °C. The acid chloride was redissolved in 2 mL anhydrous dichloromethane and added dropwise to the second flask under strong stirring. The mixture was warmed to room temperature and stirred overnight resulting in a dark red solution. Upon filtering the mixture through a silica plug (PE/EtOAc 3:2, 400 mL), the volatile components were removed *in vacuo*. The product **S3** was used without further purification as a yellow oil (270 mg, 1.11 mmol, 52 %). The reaction yields a mixture of two inseparable hydrogen bonded isomers (**S3a/S3b** = 1:1)

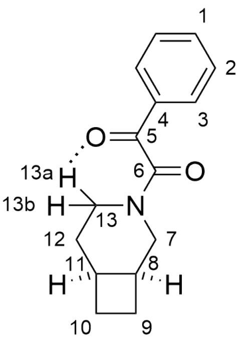

S3a

^{1}**H NMR** (300 MHz, $CDCl_3$) δ [ppm] = 8.00 – 7.94 (m, 2H, H-3), 7.66 – 7.60 (m, 1H, H-1), 7.53 – 7.47 (m, 2H, H-2), 3.92 (ddd, J = 13.3, 8.1, 4.4 Hz, 1H, H-13a), 3.54 – 3.50 (m, 1H, H-13b), 3.41 (d, J = 5.6 Hz, 1H, H-7), 3.26 (dd, J = 13.9, 5.5 Hz, 1H, H-7), 2.59 – 2.55 (m, 1H, H-11), 2.52 – 2.46 (m, 1H, H-8), 2.12 – 2.06 (m, 1H, H-9), 1.98 – 1.94 (m, 1H, H-12), 1.93 – 1.90 (m, 1H, H-10), 1.89 – 1.84 (m, 1H, H-9), 1.81 – 1.78 (m, 1H, H-10), 1.78 – 1.73 (m, 1H, H-12).

^{13}**C{^{1}H} NMR** (151 MHz, $CDCl_3$) δ [ppm] = 192.0 (C_q, C-5), 166.5 (C_q, C-6), 134.8 (CH, C-1), 133.4 (C_q, C-4), 129.8 (CH, C-3), 129.1 (CH, C-2), 47.1 (CH_2, C-7), 39.3 (CH_2, C-13), 32.5 (CH, C-8), 31.0 (CH, C-11), 26.1 (CH_2, C-12), 23.6 (CH_2. C-9), 22.4 (CH_2, C-10).

HRMS (EI+): m/z for $C_{15}H_{17}NO_2^+$ [M^+] calcd: 243.1254, found: 243.1272.

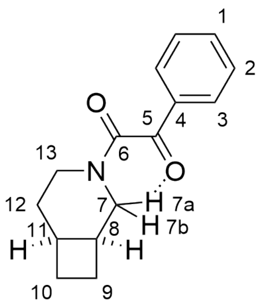

S3b

^{1}**H NMR** (300 MHz, $CDCl_3$) δ [ppm] = 8.00 – 7.94 (m, 2H, H-3), 7.66 – 7.60 (m, 1H, H-1), 7.53 – 7.47 (m, 2H, H-2), 3.84 (dd, J = 14.0, 5.1 Hz, 1H, H-7a), 3.63 (dd, J = 13.9, 5.8 Hz, 1H, H-7b), 3.49 – 3.44 (m, 1H, H-13), 3.17 (ddd, J = 13.3, 8.2, 4.3 Hz, 1H, H-13), 2.66 – 2.61 (m, 1H, H-8), 2.59 – 2.55 (m, 1H, H-11), 2.12 – 2.06 (m, 1H, H-9), 2.04 – 2.00 (m, 1H, H-10), 1.84 – 1.82 (m, 1H, H-12), 1.81 – 1.78 (m, 1H, H-9), 1.73 – 1.68 (m, 1H, H-10), 1.65 – 1.61 (m, 1H, H-12).

^{13}**C{^{1}H} NMR** (151 MHz, $CDCl_3$) δ [ppm] = 192.1 (C_q, C-5), 166.5 (C_q, C-6), 134.8 (CH, C-1), 133.3 (C_q, C-4), 129.8 (CH, C-3), 129.2 (CH, C-2), 43.3 (CH_2, C-13), 42.9 (CH_2, C-7), 32.2 (CH, C-8), 31.0 (CH, C-11), 27.5 (CH_2, C-12), 24.3 (CH_2, C-10),

23.4 (CH_2, C-9).

(*E*)-*N*'-(2-(3-azabicyclo[4.2.0]octan-3-yl)-2-oxo-1-phenylethylidene)-tosylhydrazide S4

Adapting a procedure by Lapinsky *et al.*,[4] tosyl hydrazide (239 mg, 1.28 mmol, 1.2 eq.) was dissolved in EtOH (6 mL). To this suspension H_2SO_4 (0.8 mL) was added, until everything had dissolved. Upon addition of 1-((1*S*,6*S*)-3-azabicyclo[4.2.0]octan-3-yl)-2-phenylethane-1,2-dione **S3** (260 mg, 1.07 mmol, 1.0 eq.), the reaction mixture was stirred at 85 °C overnight. The crude product was purified *via* flash column chromatography (SiO_2, PE/EtOAc 4:1 → 1:1 → 1:3) affording the product as a colourless crystalline solid (190 mg, 462 μmol, 43 %). The reaction yields a mixture of two inseparable hydrogen bonded isomers (**S4a/S4b** = 1.4:1)

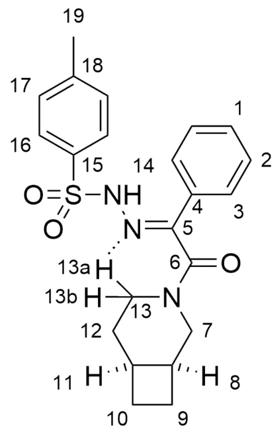

S4a

^{1}H NMR (400 MHz, $CDCl_3$) δ [ppm] = 8.24 (s, 1H, H-14), 7.86 (d, J = 8.4 Hz, 2H, H-16), 7.59 – 7.56 (m, 2H, H-3), 7.37 (t, J = 7.0 Hz, 3H, H-1, H-2), 7.29 (d, J = 8.1 Hz, 2H, H-17), 3.87 (ddd, J = 13.5 / 8.2 / 4.4 Hz, 1H, H-13a), 3.52 (ddd, J = 13.4 / 7.8 / 4.5 Hz, 1H, H-13b), 3.29 (dd, J = 13.8, 5.9 Hz, 1H, H-7), 3.11 (dd, J = 13.8, 5.9 Hz, 1H, H-7), 2.58 – 2.51 (m, 1H, H-11), 2.39 (s, 3H, H-19), 2.36 – 2.35 (m, 1H, H-8), 2.11 – 2.04 (m, 1H, H-10), 1.99 – 1.94 (m, 1H, H-12), 1.93 – 1.89 (m, 1H, H-9), 1.81 – 1.76 (m, 1H, H-10), 1.75 – 1.71 (m, 1H, H-12), 1.70 – 1.64 (m, 1H, H-9).

^{13}C{^{1}H} NMR (151 MHz, $CDCl_3$) δ [ppm] = 163.0 (C_q, C-6), 150.8 (C_q, C-5), 144.4 (C_q, C-18), 135.3 (C_q, C-15), 132.3 (C_q, C-4), 130.8 (CH, C-1), 129.8 (CH, C-17) , 129.0 (CH, C-2), 128.2 (CH, C-16), 126.4 (CH, C-3), 47.6 (CH_2, C-7), 39.5 (CH_2, C.13), 32.4 (CH, C-8), 30.7 (CH, C-11), 26.1 (CH_2, C-12), 23.5 (CH_2, C-10), 22.8 (CH_2, C-9), 21.7 (CH_3, C-19).

HRMS (EI+): m/z for $C_{22}H_{24}N_3O_3S^+$ [$(M\text{-}H)^+$] calcd: 410.1534, found: 410.0969.

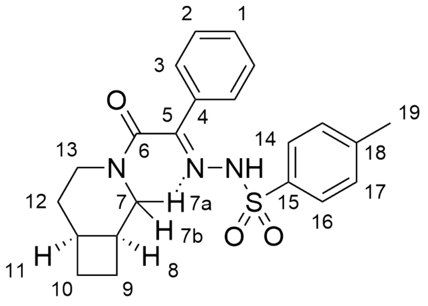

S4b

^{1}H NMR (400 MHz, $CDCl_3$) δ [ppm] = 8.17 (s, 1H, H-14), 7.86 (d, J = 8.4 Hz, 2H, H-16), 7.62 – 7.60 (m, 2H, H-3), 7.37 (t, J = 7.0 Hz, 3H, H-1, H-2), 7.29 (d, J = 8.1 Hz, 2H, H-17), 3.82 (dd, J = 12.5, 3.9 Hz, 1H, H-7a), 3.63 (dd, J = 13.9, 5.9 Hz, 1H, H-7b), 3.36 (ddd, J = 11.9, 7.6, 4.1 Hz, 1H, H-13), 3.05 (ddd, J = 13.1, 8.1, 4.3 Hz, 1H, H-13), 2.68 – 2.60 (m, 1H, H-8), 2.51 – 2.46 (m, 1H, H-11), 2.39 (s, 3H, H-19), 2.11 – 2.04 (m, 2H, H-9, H-10), 1.89 – 1.83 (m, 1H, H-9), 1.75 – 1.71 (m, 1H, H-12), 1.70 – 1.64 (m, 1H, H-10), 1.52 – 1.44 (m, 1H, H-12).

^{13}C{^{1}H} NMR (151 MHz, $CDCl_3$) δ [ppm] = 163.0 (C_q, C-6), 150.8 (C_q, C-5), 144.4 (C_q, C-18), 135.3 (C_q, C-15), 132.3 (C_q, C-4), 130.8 (CH, C-1), 129.8 (CH, C-17) , 129.0 (CH, C-2), 128.2 (CH, C-16), 126.4 (CH, C-3), 43.5 (CH_2, C-13), 42.9 (CH_2, C-7), 32.1 (CH, C-8), 30.6

(CH, C-11), 27.4 (CH_2, C-12), 24.1 (CH_2, C-10), 23.5 (CH_2, C-9), 21.7 (CH_3, C-19).

9-phenyl-1-azatricyclo[6.2.0.0^{3,6}]decan-10-one / 3-phenyl-5-azatricyclo[6.2.0.0^{2,5}]decan-4-one 11, 12

Adapting a procedure of König *et al.*,[6] an oven dried Schlenk tube was charged with (*E*)-*N*'-(2-(3-azabicyclo[4.2.0]octan-3-yl)-2-oxo-1-phenylethylidene)-tosylhydrazide (60 mg, 146 μmol, 1.0 eq.) and Cs_2CO_3 (48 mg, 146 μmol, 1.0 eq.) and evacuated and backfilled with argon three times. Anhydrous dichloromethane (5 mL) was added and the mixture was subsequently irradiated at 365 nm while stirring vigorously for 24 h until the tosylhydrazone was fully consumed. The crude product was adsorbed onto Celite®, filtered over a silica plug (SiO_2, PE/EtOAc 1:1, 200 mL) and further purified *via* flash column chromatography (SiO_2, PE/EtOAc 1:0 → 1:1 → 1:3). The reaction yields two regioisomers in a ratio of **11/12** = 1.6:1 affording the products as a mixture of two inseparable diastereomers in a ratio of **11a/11b** = 1.4:1 and **12a/12b** = 1.8:1 as colourless crystalline solids (23 mg, 101 μmol, 70 %). The data for each isomer are given below.

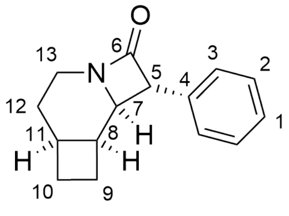

11a

^{1}**H NMR** (400 MHz, $CDCl_3$) δ [ppm] = 7.32 – 7.32 (m, 2H, H-2), 7.27 – 7.27 (m, 2H, H-3), 7.25 – 7.25 (m, 1H, H-1), 3.83 (d, J = 2.0 Hz, 1H, H-5), 3.86 – 3.79 (m, 1H, H-13), 3.58 – 3.58 (m, 1H, H-7), 3.26 – 3.19 (m, 1H, H-13), 2.99 – 2.90 (m, 1H, H-8), 2.74 – 2.63 (m, 1H, H-11), 2.27 – 2.19 (m, 1H, H-10), 2.16 – 2.06 (m, 2H, H-9), 2.01 – 1.93 (m, 1H, H-12), 1.77 – 1.67 (m, 1H, H-10), 1.65 – 1.57 (m, 1H, H-12).

^{13}**C{^{1}H} NMR** (101 MHz, $CDCl_3$) δ [ppm] = 168.9 (C_q, C-6), 135.6 (C_q, C-4), 128.9 (CH, C-2), 127.6 (CH, C-3), 127.4 (CH, C-1), 57.8 (CH, C-5), 55.3 (CH, C-7), 36.1 (CH_2, C-13), 33.5 (CH, C-8), 30.3 (CH, C-11), 24.4 (CH_2, C-10), 22.1 CH_2, C-12), 18.7 (CH_2, C-9).

HRMS (EI+): m/z for $C_{15}H_{17}NO^+$ [M^+] calcd: 227.1305, found: 227.1318.

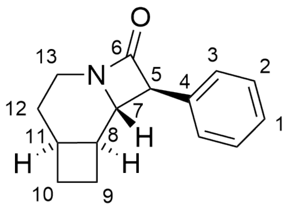

11b

^{1}**H NMR** (400 MHz, $CDCl_3$) δ [ppm] = 7.31 – 7.29 (m, 4H, H-2, H-3), 7.25 – 7.21 (m, 1H, H-1), 3.92 (s, 1H, H-5), 3.59 (d, J = 2.0 Hz, 1H, H-13), 3.19 – 3.15 (m, 1H, H-7), 3.05 (dd, J = 13.8, 7.2 Hz, 1H, H-13), 2.57 – 2.48 (m, 1H, H-11), 2.41 – 2.36 (m, 1H, H-8), 2.35 – 2.31 (m, 1H, H-9), 2.19 – 2.15 (m, 1H, H-12), 2.04 – 2.01 (m, 1H, H-10), 1.95 – 1.89 (m, 1H, H-10), 1.61 – 1.58 (m, 1H, H-9), 1.50 – 1.43 (m, 1H, H-12).

^{13}**C{^{1}H} NMR** (101 MHz, $CDCl_3$) δ [ppm] = 167.7 (C_q, C-6), 135.8 (C_q, C-4), 128.9 (Ch, C-2), 127.5 (CH, C-3), 127.5 (CH, C-1), 63.0 (CH, C-5), 53.6 (CH, C-7), 39.1 (CH_2, C-13), 32.5 (CH_2, C-9), 31.8 (CH, C-8), 29.3 (CH, C-11), 26.4 (CH_2, H-12), 24.7 (CH_2, C-10).

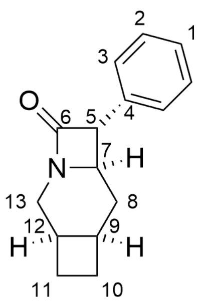

12a

^{1}H NMR (400 MHz, $CDCl_3$) δ [ppm] = 7.37 – 7.31 (m, 2H, H-2), 7.30 – 7.27 (m, 3H, H-1, H-3), 3.82 (d, J = 2.0 Hz, 1H, H-5), 3.54 – 3.49 (m, 1H, H-7), 3.49 – 3.41 (m, 1H, H-13), 3.35 – 3.27 (m, 1H, H-13), 2.75 – 2.69 (m, 1H, H-9), 2.60 – 2.51 (m, 1H, H-12), 2.30 – 2.23 (m, 1H, H-8), 2.22 – 2.16 (m, 1H, H-10), 1.89 – 1.84 (m, 1H, H-8), 1.84 – 1.79 (m, 1H, H-10), 1.73 – 1.59 (m, 2H, H-11).

^{13}C{^{1}H} NMR (101 MHz, $CDCl_3$) δ [ppm] = 168.3 (C_q, C-6), 136.0 (C_q, C-4), 128.9 (CH, C-2), 127.6 (CH, C-1), 127.5 (CH, C-3), 63.6 (CH, C-5), 58.9 (CH, C-7), 36.9 (CH, C-9), 36.8 (CH_2, C-13), 32.2 (CH, C-12), 25.5 (CH_2, C-8), 25.2 (CH_2, C-10), 24.4 (CH_2, C-11).

HRMS (EI+): m/z for $C_{15}H_{17}NO^+$ [M^+] calcd: 227.1305, found: 227.1308.

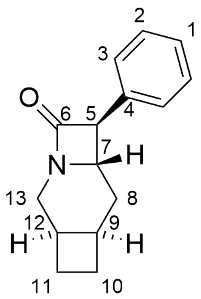

12b

^{1}H NMR (400 MHz, $CDCl_3$) δ [ppm] = 7.34 – 7.32 (m, 4H, H-2, H-3), 7.26 – 7.24 (m, 1H, H-1), 3.92 (s, 1H, H-5), 3.81 – 3.77 (m, 1H, H-7), 3.75 – 3.72 (m, 1H, H-13), 3.11 (dd, J = 13.2, 3.4 Hz, 1H, H-13), 2.78 – 2.75 (m, 1H, H-9), 2.71 – 2.65 (m, 1H, H-11), 2.32 – 2.29 (m, 1H, H-11), 2.18 – 2.16 (m, 1H, H-10), 2.07 – 2.03 (m, 1H, H-8), 1.92 – 1.89 (m, 1H, H-11), 1.84 – 1.82 (m, 1H, H-10), 1.74 – 1.70 (m, 1H, H-8).

^{13}C{^{1}H} NMR (101 MHz, $CDCl_3$) δ [ppm] = 169.2 (C_q, C-6), 135.8 (C_q, C-4), 128.9 (CH, C-2), 127.6 (CH, C-1), 127.5 (CH, C-3), 62.9 (CH, C-5), 53.4 (CH, C-7), 41.2 (CH_2, C-13), 32.2 (CH_2, C-8), 31.0 (CH, C-9), 28.4 (CH, C-12), 25.9 (CH_2, C-11), 23.0 (CH_2, C-10).

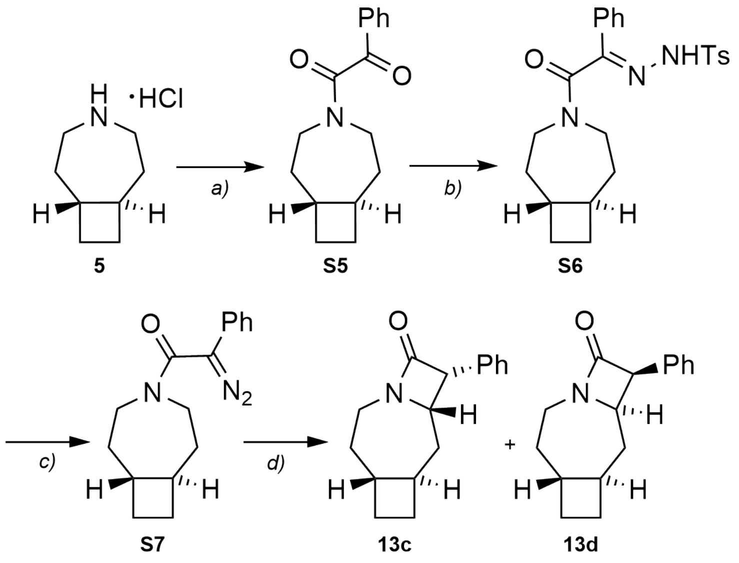

Figure S4: Conditions for the the synthesis of **13**: (a) pyridine (2.2 eq.), phenylglyoxalic acid (1.0 eq.), DMF (1.7 eq.), oxalyl chloride (1.5 eq.), CH_2Cl_2, 0 °C → RT, 16 h, 64 %; (b) tosyl hydrazide, H_2SO_4, EtOH, 85 °C, 16 h, 43 %; (c) NaH (1.0 eq.), DME, Aliquat® 336, RT → 60 °C, 4 h, 85 %, estimated purity 70 %; (d) C_6D_6, hν 390 nm, 24 h, 50 %, **13c/13d** 1.1:1.

1-((1*S*,7*S*)-4-azabicyclo[5.2.0]nonan-4-yl)-2-phenylethane-1,2-dione S5

Adapting a procedure by He *et al.*,[3] in a flame-dried Schlenk flask phenylglyoxylic acid (239 mg, 1.59 mmol, 1.0 eq.) was dissolved in anhydrous dichloromethane (5 mL). To this, DMF (0.27 mL, 2.7 mmol, 1.7 eq.) was added and the mixture was cooled to 0 °C. Oxalyl chloride (0.20 mL, 2.39 mmol, 1.5 eq.) was added dropwise and the reaction mixture was stirred at 0 °C for 30 min. With an external vacuum pump and cooling trap, the solvent was removed, resulting in the acid chloride as a viscous yellow liquid. In a second flame-dried Schlenk flask, (1*S*,7*S*)-4-azabicyclo[5.2.0]nonane ·HCl **5** (360 mg, 2.23 mmol, 1.4 eq.) and dry pyridine (0.27 mL, 3.5 mmol, 2.2 eq.) was dissolved in anhydrous dichloromethane (3 mL) and cooled to 0 °C. The acid chloride was redissolved in 5 mL anhydrous dichloromethane and added dropwise to the second flask under strong stirring. The mixture was warmed to room temperature and stirred overnight resulting in a dark red solution. Upon filtering the mixture twice through a silica plug (PE/EtOAc 3:2, 200 mL), the volatile components were removed *in vacuo*. The product **S5** was used without further purification as a yellow oil (260 mg, 1.01 mmol, 64 %).

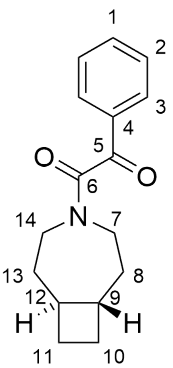

^{1}H NMR (600 MHz, $CDCl_3$) δ [ppm] = 7.96 (d, J = 7.2 Hz, 2H, H-3), 7.64 (t, J = 7.4 Hz, 1H, H-1), 7.51 (t, J = 7.8 Hz, 2H, H-2), 3.76 – 3.65 (m, 2H, H-7/H-14), 3.50 – 3.43 (m, 1H, H-7/H-14), 3.27 – 3.19 (m, 1H, H-7/H-14), 2.19 – 2.09 (m, 3H, H-8/H-13, H-9/H-12), 2.03 – 1.89 (m, 2H, H-10/H-11), 1.79 – 1.71 (m, 1H, H-8/H-13), 1.65 – 1.47 (m, 3H, H-8/H-13, H-10/H-11), 1.46 – 1.39 (m, 1H, H-8/H-13).

^{13}C{^{1}H} NMR (151 MHz, $CDCl_3$) δ [ppm] = 191.9 (C_q, C-5), 167.2 (C_q, C-6), 134.7 (CH, C-1), 133.3 (C_q, C-4), 129.8 (CH, C-3), 129.1 (CH, C-2), 51.0 (CH_2, C-7/C-14), 48.5 (CH_2, C-7/C-14), 41.8 (CH, C-9/C-12), 41.1 (CH, C-9/C-12), 34.0 (CH_2, C-8/C-13), 31.3 (CH_2, C-8/C-13), 26.7 (CH_2, C-10/C-11), 26.1 (CH_2, C-10/C-11).

HRMS (EI+): m/z for $C_{16}H_{19}NO_2^+$ [M^+] calcd: 257.1410, found: 257.1391.

***N*'-(2-((1*S*,7*S*)-4-azabicyclo[5.2.0]nonan-4-yl)-2-oxo-1-phenylethylidene)-tosyl-hydrazide S6**

Adapting a procedure by Lapinsky *et al.*,[4] tosyl hydrazide (225 mg, 1.21 mmol, 1.2 eq.) was dissolved in EtOH (5 mL). To this suspension H_2SO_4 (7 drops) was added, until everything had dissolved. Upon addition of 1-((1*S*,7*S*)-4-azabicyclo[5.2.0]nonan-4-yl)-2-phenylethane-1,2-dione **S5** (260mg, 1.01 mmol, 1.0 eq.) in 5 mL EtOH, the reaction mixture was stirred at 85 °C overnight. The crude product was purified *via* flash column chromatography (SiO_2, PE/EtOAc 3:1 → 2:1 → 1:1 → 1:1 → 0:1) affording the product **S6** as a colourless oil (186 mg, 440 μmol, 43 %).

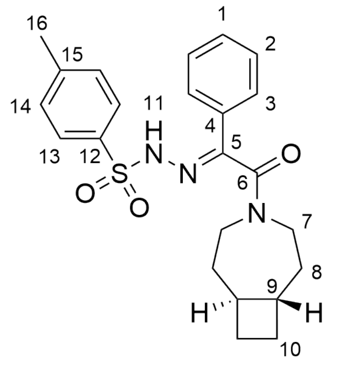

^{1}**H NMR** (600 MHz, $CDCl_3$) δ [ppm] = 8.11 (s, 1H, H-15), 7.87 (d, J = 8.4 Hz, 2H, H-17), 7.59 (dd, J = 8.1, 1.7 Hz, 2H, H-3), 7.43 – 7.34 (m, 3H, H-1, H-2), 7.29 (d, J = 8.0 Hz, 2H, H-18), 3.80 – 3.73 (m, 1H, H-7/H-14), 3.64 – 3.56 (m, 1H, H-7/H-14), 3.44 – 3.37 (m, 1H, H-7/H-14), 3.11 – 3.04 (m, 1H, H-7/H-14), 2.39 (s, 3H, H-20), 2.20 – 2.11 (m, 1H, H-8/H-13), 2.12 – 2.01 (m, 2H, H-9/H-12), 2.02 – 1.95 (m, 1H, H-10/H-11), 1.91 – 1.84 (m, 1H, H-10/H-11), 1.64 – 1.56 (m, 1H, H-10/H-11), 1.54 – 1.50 (m, 1H, H-8/H-13), 1.51 – 1.44 (m, 2H, H-8/H-13, H-10/H-11), 1.20 – 1.11 (m, 1H, H-8/H-13).
^{13}**C{^{1}H} NMR** (151 MHz, $CDCl_3$) δ [ppm] = 164.0 (C_q, C-6), 151.5 (C_q, C-5), 144.5 (C_q, C-16), 135.2 (C_q, C-19), 132.4 (C_q, C-4), 130.9 (CH, H-1), 129.8 (CH, C-18), 129.0 (CH, C-2), 128.2 (CH, C-17), 126.4 (CH, C-3), 51.8 (CH_2, C-7/C-14), 48.7 (CH_2, C-7/C-14), 41.7 (CH, C-9/C-12), 41.1 (CH, C-9/C-12), 33.9 (CH_2, C-8/C-13), 31.1 (CH_2, C-8/C-13), 26.9 (CH_2, C-10/C-11), 26.0 (CH_2, C-10/C-11), 21.8 (CH_3, C-20).
HRMS (EI+): m/z for $C_{16}H_{20}NO^+$ [$(M\text{-}TsN_2)^+$] calcd: 242.1539, found: 242.1531.

1-((1*S*,7*S*)-4-azabicyclo[5.2.0]nonan-4-yl)-2-diazo-2-phenylethan-1-one S7

Adapting a procedure of Corey *et al.*,[5] a flame-dried Schlenk flask was charged with NaH (7.3 mg, 305 μmol, 1.0 eq.) and dry dimethoxyethane (4 mL). To this a suspension of *N*'-(2-((1*S*,7*S*)-4-azabicyclo[5.2.0]nonan-4-yl)-2-oxo-1-phenylethylidene)-tosylhydrazide **S6** (130 mg, 305 μmol, 1.0 eq.) and Aliquat® 336 (1 drop) was added. The reaction mixture was stirred at room temperature until formation of H_2-gas ceased (20 min), then heated to 60 °C and stirred for 4 h. The initially pale yellow and clear solution changed to a bright orange solution with a colourless precipitate. The reaction mixture was cooled to room temperature, filtered over Celite®, eluted with Et_2O and removed the solvent *in vacuo*. The product **S7** is prone to decomposition and was used without further purification with an estimated purity of 70 % (70 mg (w %), 260 μmol, 85 %).

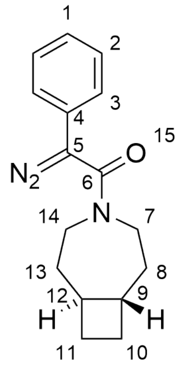

^{1}**H NMR** (600 MHz, $CDCl_3$) δ [ppm] = 7.38 (d, J = 24.3 Hz, 2H, H-3), 7.31 (d, J = 17.6 Hz, 2H, H-2), 7.21 (d, J = 19.3 Hz, 1H, H-1), 3.24 (t, J = 8.6 Hz, 1H, H-7/H-14), 2.24 – 1.70 (m, 7H, H-7/H-14, H-10, H-11), 1.60 – 1.52 (m, 4H, H-8, H-13), 1.36 – 1.29 (m, 2H, H-9, H-12).
IR (ATR): $\tilde{\nu}(N_2) = 2054\ cm^{-1}$.

(1*S*,9*S*)-4-phenyl-6-azatricyclo[7.2.0.0^{3,6}]undecan-5-one 13

Adapting a procedure of Corey *et al.*,[5] a vial was charged with 1-((1*S*,7*S*)-4-azabicyclo[5.2.0]nonan-4-yl)-2-diazo-2-phenylethan-1-one **S7** (56 mg, 208 µmol, 1.0 eq.) and evacuated and backfilled with argon three times. Anhydrous C_6D_6 (1 mL) was added and the reaction mixture was irradiated by a 390 nm LED light for 24 h. The crude product was purified *via* flash column chromatography (SiO_2, PE/EtOAc 1:0 → 4:1 → 2:1 → 0:1). The reaction yields a mixture of two inseparable diastereomers in a ratio of **13c/13d** = 1.1:1 as a yellow oil (25 mg, 104 µmol, 50 %). The data for each isomer are given below.

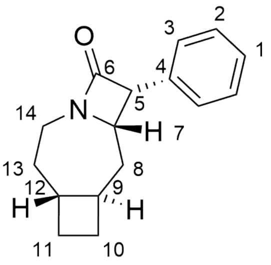

13c

^{1}H NMR (600 MHz, $CDCl_3$) δ [ppm] = 7.32 – 7.30 (m, 2H, H-2), 7.26 – 7.24 (m, 1H, H-1), 7.22 – 7.20 (m, 2H, H-3), 4.48 (d, J = 3.5 Hz, 1H, H-5), 3.90 (ddd, J = 11.6, 5.6, 3.5 Hz, 1H, H-7), 3.78 (ddd, J = 13.8, 9.3, 7.7 Hz, 1H, H-14), 3.26 (dddd, J = 13.8, 7.6, 3.5, 1.5 Hz, 1H, H-14), 1.97 – 1.93 (m, 1H, H-13), 1.92 – 1.90 (m, 1H, H-9), 1.88 – 1.86 (m, 1H, H-12), 1.78 – 1.74 (m, 1H, H-10), 1.64 – 1.59 (m, 1H, H-11), 1.55 – 1.53 (m, 1H, H-13), 1.53 – 1.50 (m, 1H, H-11), 1.48 – 1.47 (m, 1H, H-10), 1.39 (dd, J = 6.9, 3.1 Hz, 1H, H-8), 0.88 – 0.84 (m, 1H, H-8).

^{13}C{^{1}H} NMR (151 MHz, $CDCl_3$) δ [ppm] = 168.7 (C_q, C-6), 134.0 (C_q, C-4), 128.9 (CH, C-2), 128.6 (CH, C-1), 127.4 (CH, C-3), 60.1 (CH, C-7), 57.3 (CH, C-5), 43.6 (CH, C-12), 42.0 (CH, C-5), 41.2 (CH_2, C-14), 36.5 (CH_2, C-8), 31.2 (CH_2, C-13), 26.1 (CH_2, C-11), 25.3 (CH_2, C-10).

HRMS (EI+): m/z for $C_{16}H_{19}NO^+$ [M^+] calcd: 241.1461, found: 241.1472.

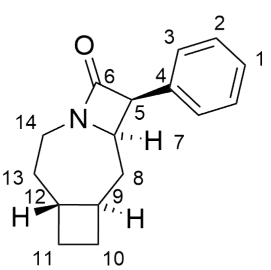

13d

^{1}H NMR (600 MHz, $CDCl_3$) δ [ppm] = 7.35 – 7.33 (m, 2H, H-2), 7.28 – 7.26 (m, 1H, H-1), 7.23 – 7.22 (m, 2H, H-3), 4.48 (d, J = 5.4 Hz, 1H, H-5), 4.15 (ddd, J = 8.0, 5.7, 3.4 Hz, 1H, H-7), 4.10 (ddd, J = 14.0, 4.6, 1.8 Hz, 1H, H-14), 2.66 (dddd, J = 13.8, 12.6, 3.4, 0.9 Hz, 1H, H-14), 1.91 – 1.90 (m, 1H, H-9), 1.88 – 1.86 (m, 1H, H-11), 1.85 – 1.83 (m, 1H, H-12), 1.83 – 1.79 (m, 1H, H-10), 1.69 – 1.64 (m, 1H, H-13), 1.64 – 1.59 (m, 1H, H-11), 1.56 – 1.55 (m, 1H, H-13), 1.47 – 1.45 (m, 2H, H-8), 1.39 – 1.36 (m, 1H, H-10).

^{13}C{^{1}H} NMR (151 MHz, $CDCl_3$) δ [ppm] = 168.3 (C_q, C-6), 133.8 (C_q, C-4), 129.0 (CH, C-2), 128.8 (CH, C-3), 127.4 (CH, C-1), 58.8 (CH, C-5), 58.2 (CH, C-7), 46.0 (CH, C-12), 45.0 (CH, C-9), 42.9 (CH_2, C-14), 34.6 (CH_2, C-13), 32.6 (CH_2, C-8), 26.2 (CH_2, C-11), 25.3 (CH_2, C-10).

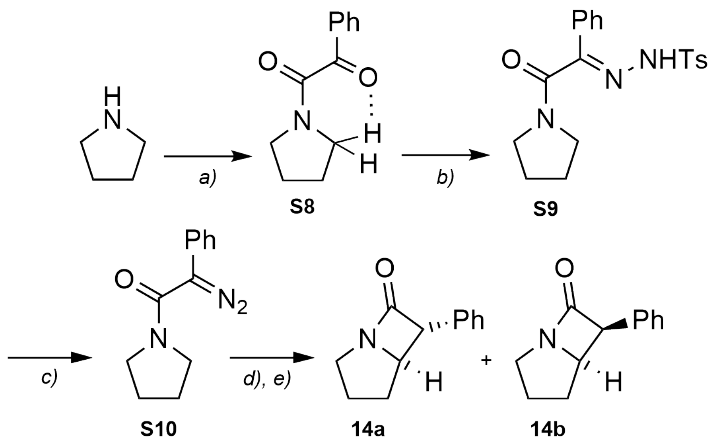

Figure S5: Conditions for the the synthesis of **14**: (a) pyridine (2.2 eq.), phenylglyoxalic acid (1.0 eq.), DMF (1.7 eq.), oxalyl chloride (1.5 eq.), CH_2Cl_2, 0 °C → RT, 16 h, 97 %; (b) tosyl hydrazide, H_2SO_4, EtOH, 85 °C, 16 h, 63 %; (c) NaH (1.0 eq.), DME, Aliquat® 336, RT → 60 °C, 3 h, 91 %, estimated purity 75 %; (d) C_6D_6, hν 390 nm, 1.5 h; e) C_6D_6, 80 °C, 16 h; 36 %, **14a/14b** 95:5.

1-Phenyl-2-(pyrrolidin-1-yl)ethane-1,2-dione S8

Following a modified procedure by He *et al.*,[3] in a flame-dried Schlenk flask phenylglyoxylic acid (3.54 g 23.6 mmol, 1.0 eq.) was dissolved in anhydrous dichloromethane (60 mL). Dry DMF (4 mL, 40 mmol, 1.7 eq.) was added, and the solution cooled to 0 °C. Oxalyl chloride (3.0 mL, 4.4 g 35.0 mmol, 1.5 eq.) was added dropwise resulting in a yellow solution. The solution was stirred for 30 minutes at 0 °C and concentrated under reduced pressure *via* an external vacuum pump equipped with a cold trap giving the acid chloride as a viscous liquid. In a second flame-dried Schlenk flask, pyrrolidine (2.8 mL, 33.5 mmol, 1.4 eq.) and pyridine (4 mL, 4.1 g 51.5 mmol, 2.2 eq.) were dissolved in anhydrous dichloromethane (20 mL) and cooled to 0 °C. The acid chloride was redissolved in anhydrous dichloromethane (20 mL) and added dropwise to the second flask under strong stirring. The mixture was warmed to room temperature and stirred overnight resulting in a dark red solution. The crude product was concentrated under reduced pressure and purified *via* flash column chromatography (silica, PE:EA 2:1 to 1:1) and dried *in vacuo*. The product **S8** was obtained as pale yellow oil in 97 % yield (4.630 g, 22.78 mmol).

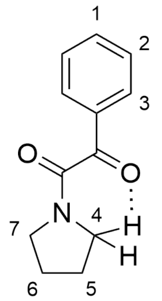

^{1}H NMR (600 MHz, $CDCl_3$) δ [ppm] = 8.01-7.93 (m, 2H, H-2/H-3), 7.60 (ddt, J = 8.7, 7.3, 1.3 Hz, 1H, H-1), 7.51-7.44 (m, 2H, H-2/H-3), 3.62 (dd, J = 7.4, 6.1 Hz, 2H, H-4/H-7), 3.39 (dd, J = 6.5, 6.5 Hz, 2H, H-4/H-7), 1.98-1.86 (m, 4H, H-5, H-6).

Spectroscopic data are in accordance with those previously reported in literature.[7]

***N*'-(2-oxo-1-phenyl-2-(pyrrolidin-1 yl)ethylidene) S9**

Adapting a modified procedure by Lapinsky et al.,[4] tosyl hydrazide (3.30 g 17.7 mmol, 1.2 eq.) was dissolved in ethanol (70 mL). To this suspension, H_2SO_4 (98 %, 0.2 mL) added resulting

in a clear solution. Upon addition of 1-Phenyl-2-(pyrrolidin-1-yl)ethane-1,2-dione **S8** (3.00 g 14.8 mmol, 1.0 eq.) the reaction mixture was stirred at 85 °C overnight. After cooling to room temperature, the product crystallized out, was filtered off, and washed with Et_2O. The remaining solution was concentrated under reduced pressure and additional product was allowed to crystallize at 5 °C overnight, followed by filtration. The product **S9** was obtained as a colourless crystalline solid (3.43 g, 9.23 mmol, 63 %).

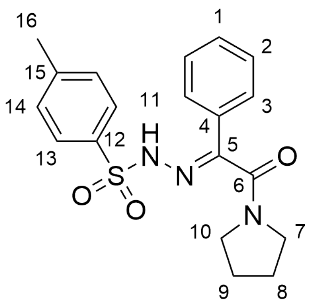

^{1}**H NMR** (600 MHz, $CDCl_3$) δ [ppm] = 8.76 (s, 1H, H-11), 7.85 (d, J = 8.4 Hz, 2H, H-14), 7.59 – 7.55 (m, 2H, H-3), 7.40 – 7.34 (m, 3H, H-1, H-2), 7.25 – 7.24 (m, 2H, H-13), 3.63 (t, J = 7.1 Hz, 2H, H-7/H-10), 3.13 (t, J = 6.8 Hz, 2H, H-7/H-10), 2.37 (s, 3H, H-14/H-16), 1.95 – 1.89 (m, 2H, H-8/H-9), 1.88 – 1.82 (m, 2H, H-8/H-9).
^{13}**C{^{1}H} NMR** (151 MHz, $CDCl_3$) δ [ppm] = 162.3 (C_q, C-6), 150.7 (C_q, C-5), 144.3 (C_q, C-15), 135.3 (C_q, C-12), 132.0 (C_q, C-4), 130.6 (CH, C-1), 129.7 (CH, C-13), 129.0 (CH, C-2), 128.1 (CH, C-14), 126.5 (CH, C-3), 47.2 (CH_2, C-7/C-14), 45.5 (CH_2, C-7/C-14), 25.8 (CH_2, C-8/C-9), 24.2 (CH_2, C-8/C-9), 21.7 (CH_3, C-16).

2-diazo-2-phenyl-1-(pyrrolidin-1-yl)ethan-1-one S10

Following a procedure of Corey *et al.*,[5] a flame-dried Schlenk flask was charged with NaH (12.9 mg, 0.53 mmol, 1.0 eq.) and dry dimethoxyethane (6.5 mL). To this a suspension of *N*'-(2-oxo-1-phenyl-2-(pyrrolidin-1-yl)ethylidene)tosylhydrazide **S9** (200 mg, 0.53 mmol, 1.0 eq.) and Aliquat® 336 (1 drop) was added. The reaction mixture was stirred at room temperature until formation of H_2-gas ceased (25 min), then heated to 60 °C and stirred for 3 h. The initially pale yellow and clear solution changed to a bright orange solution with a colourless precipitate. The reaction mixture was cooled to room temperature, filtered over Celite®, eluted with Et_2O and removed the solvent *in vacuo*. The product **S10** is prone to decomposition and was used without further purification with an estimated purity of 75 % (140 mg (w%), 490 μmol, 91 %).

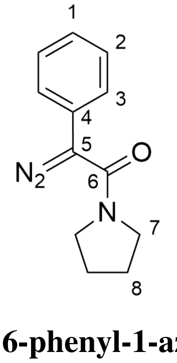

^{1}**H NMR** (400 MHz, $CDCl_3$) δ [ppm] = 7.39 – 7.34 (m, 2H, H-2), 7.33 – 7.29 (m, 2H, H-3), 7.20 – 7.14 (m, 1H, H-1), 3.43 (s, 4H, H-7), 1.96 – 1.84 (m, 4H, H-8).
^{13}**C{^{1}H} NMR** (151 MHz, $CDCl_3$) δ [ppm] = 163.8 (C_q, C-8), 129.1 (CH, C-2), 127.4 (C_q, C-4), 126.0 (CH, C-1), 125.3 (CH, C-3), 63.0 (C_q, C-5), 47.9 (CH_2, C-7), 25.5 (CH_2, C-8).
IR (ATR): $\tilde{\nu}(N_2) = 2050\ cm^{-1}$.

6-phenyl-1-azabicyclo[3.2.0]heptan-7-one 14

Adapting a procedure of Corey *et al.*,[5] two Schlenk flasks were each charged with 2-diazo-2-phenyl-1-(pyrrolidin-1-yl)ethan-1-one **S10** (25 mg, 116 μmol, 1.0 eq.), evacuated and backfilled with argon three times and brought into an Ar-filled glovebox. To both flasks, dry C_6D_6 (0.6 mL)

was added and the solutions transferred to J. Young tubes. One tube was irradiated at 390 nm for 1.5 h, while the other was heated to 80 °C overnight. The solutions were combined and purified *via* flash column chromatography (silica, PE:EtOAc 4:1). The reaction yields a mixture of two diastereomers in a ratio of **14a/14b** = 95:5 as a colourless solid (15.7 mg, 83,9 μmol, 36 %).

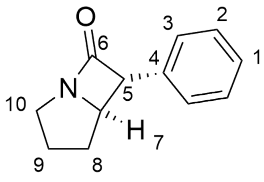

14a

^{1}**H NMR** (400 MHz, C_6D_6) δ [ppm] = 7.26 – 7.22 (m, 2H, H-2), 7.13 (ddd, J = 7.9, 6.2, 1.2 Hz, 2H, H-3), 7.08 – 7.03 (m, 1H, H-1), 3.61 (d, J = 2.2 Hz, 1H, H-5), 3.43 (dt, J = 11.0, 7.4 Hz, 1H, H-10), 3.14 (ddd, J = 7.2, 6.1, 2.1 Hz, 1H, H-7), 2.45 (ddd, J = 11.0, 8.2, 4.4 Hz, 1H, H-10), 1.49 (dtt, J = 15.9, 7.9, 4.2 Hz, 1H, H-8), 1.44 – 1.38 (m, 1H, H-9), 1.34 – 1.27 (m, 1H, H-8), 0.96 – 0.91 (m, 1H, H-9).

^{13}**C{^{1}H} NMR** (151 MHz, C_6D_6) δ [ppm] = 175.6 (C_q, C-6), 136.9 (C_q, C-4), 129.0 (CH, C-3), 127.7 (CH, C-2), 127.4 (CH, C-1), 61.6 (CH, C-5), 60.6 (CH, C-7), 45.8 (CH_2, C-10), 30.2 (CH_2, C-8), 29.9 (CH_2, C-9).

HRMS (EI+): m/z for $C_{12}H_{13}NO^+$ [M^+] calcd: 187.0992, found: 187.0962.

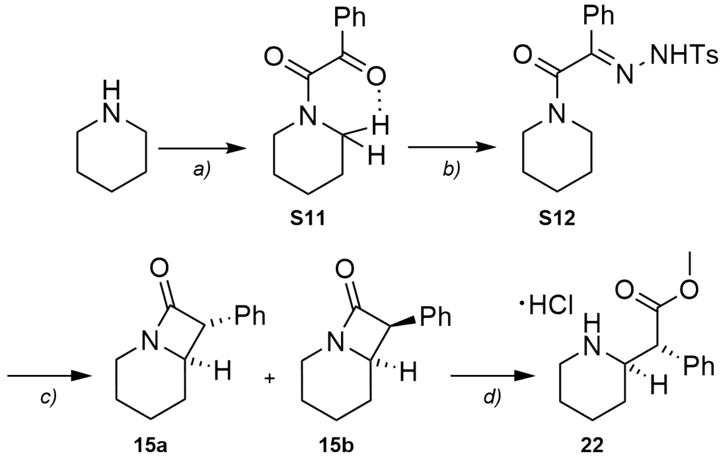

Figure S6: Conditions for the the synthesis of **15**: (a) NEt_3 (1.45 eq.), DMAP (0.01 eq.), phenylglyoxalic acid (1.0 eq.), DMF (1.7 eq.), oxalyl chloride (1.5 eq.), CH_2Cl_2, 0 °C → RT, 16 h, 59 %; (b) tosyl hydrazide, H_2SO_4, EtOH, 85 °C, 16 h, 72 %; (c) Cs_2CO_3, MeCN, hν 365 nm, 72 h, 64 %kl, **15a/15b** 3.5:1; d) HCl (2 M in Et_2O), MeOH, 55 °C, 7 h, 99 %.

1-phenyl-2-(piperidin-1-yl)ethane-1,2-dione S11

Following a modified procedure by He *et al.*,[3] in a flame-dried Schlenk flask phenylglyoxylic acid (3.67 g, 25.3 mmol, 1.0 eq.) was dissolved in anhydrous dichloromethane (30 mL). To this, DMF (4.3 mL, 42.8 mmol, 1.7 eq.) was added and the mixture was cooled to 0 °C. Oxalyl chloride (3.2 mL, 37.77 mmol, 1.5 eq.) was added dropwise and the reaction mixture was stirred at 0 °C for 30 min. With an external vacuum pump and cooling trap, the solvent was removed, resulting in the acid chloride as a viscous yellow liquid. In a second flame-dried Schlenk flask, piperidine (3.5 mL, 35.3 mmol, 1.4 eq.), DMAP (15.4 mg, 0.13 mmol, 0.01 eq.) and dry NEt_3 (5.0 mL, 36.5 mmol, 1.45 eq.) was dissolved in anhydrous dichloromethane (50 mL) and cooled

to 0 °C. The acid chloride was redissolved in 30 mL anhydrous dichloromethane and added dropwise to the second flask under strong stirring. The mixture was warmed to room temperature and stirred overnight resulting in a brown solution. The reaction mixture was washed with HCl (1 M, 4x200 mL), water (3x200 mL) and brine (3x150 mL), dried over $MgSO_4$ and removed the solvent *in vacuo*. The viscous brown crude product was redissolved in 30 mL EtOAc and the solution was concentrated under reduced pressure until the product precipitated as a colourless solid. The product was then filtered off and washed with cold EtOAc (10 mL). Subsequently, the mother liquor was concentrated *in vacuo*, overlayed with an excess amount of pentane and stored at -25 °C overnight. The solid was filtered off, washed with pentane (30 mL) and the combined fractions were dried *in vacuo* affording the product **S11** as a colourless crystalline solid (3.2 g, 14.7 mmol, 59 %).

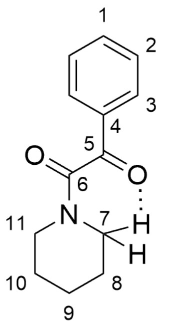

^{1}H NMR (600 MHz, $CDCl_3$) δ [ppm] = 7.95 (d, J = 7.0 Hz, 2H, H-3), 7.64 (t, J = 7.4 Hz, 1H, H-2), 7.54 – 7.49 (m, 2H, H-1), 3.73 – 3.68 (m, 2H, H-7), 3.32 – 3.27 (m, 2H, H-11), 1.74 – 1.66 (m, 4H, H-8, H-10), 1.58 – 1.52 (m, 2H, H-9).

^{13}C{^{1}H} NMR (151 MHz, $CDCl_3$) δ [ppm] = 191.7 (C_q, C-5), 165.2 (C_q, C-6), 134.4 (CH, C-1), 133.0 (C_q, C-4), 129.4 (CH, C-3), 128.8 (CH, C-2), 46.8 (CH_2, C-11), 41.9 (CH_2, C-7), 26.0 (CH_2, C-9), 25.2 (CH_2, C-8/C-10), 24.2 (CH_2, C-8/C-10).

HRMS (EI+): m/z for $C_{13}H_{15}NO_2^+$ [M^+] calcd: 217.1098, found: 217.1114.

Spectroscopic data are in accordance with those previously reported in literature.[8]

N'-(2-oxo-1-phenyl-2-(piperidin-1-yl)ethylidene)-tosylhydrazide S12

Following a modified procedure by Lapinsky *et al.*,[4] tosyl hydrazide (2.26 g, 12.2 mmol, 1.2 eq.) was dissolved in EtOH (60 mL). To this suspension H_2SO_4 (3 mL) was added, until everything had dissolved. Upon addition of 1-phenyl-2-(piperidin-1-yl)ethane-1,2-dione **S11** (2.2 g, 10.1 mmol, 1.0 eq.), the reaction mixture was stirred at 85 °C overnight. After cooling to -5 °C, the precipitate was filtered off and dried *in vacuo* affording the product **S12** as a colourless crystalline solid (2.8 g, 7.26 mmol, 72 %).

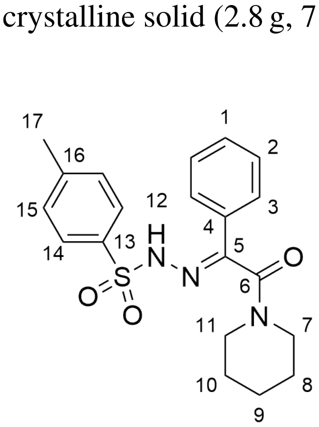

^{1}H NMR (600 MHz, $CDCl_3$) δ [ppm] = 8.14 (s, 1H, H-12), 7.86 (d, J = 8.4 Hz, 2H, H-14), 7.58 (dd, J = 8.2, 1.6 Hz, 2H, H-3), 7.42 – 7.34 (m, 3H, H-1, H-2), 7.29 (d, J = 8.6 Hz, 2H, H-15), 3.71 (t, J = 5.7 Hz, 2H, H-7/H-11), 3.19 (t, J = 5.7 Hz, 2H, H-7/H-11), 2.39 (s, 3H, H-17), 1.73 – 1.61 (m, 4H, H-8, H-10), 1.52 – 1.41 (m, 2H, H-9).

^{13}C{^{1}H} NMR (151 MHz, $CDCl_3$) δ [ppm] = 162.0 (C_q, C-6), 150.9 (C_q, C-5), 144.5 (C_q, C-13), 135.2 (C_q, C-16), 132.2 (C_q, C-4), 130.8 (CH, C-1), 129.8 (CH, C-15), 129.0 (CH, C-2), 128.2 (CH, C-14), 126.4 (CH, C-3), 47.6

(CH_2, C-7/C-11), 42.4 (CH_2, C-7/C-11), 26.5 (CH_2, C-9), 25.7 (CH_2, C-8/C-10), 24.3 (CH_2, C-8/C-10), 21.8 (CH_3, C-17).
HRMS (EI+): m/z for $C_{13}H_{16}N_3O^+$ [$(M\text{-}Ts)^+$] calcd: 230.1288, found: 230.1277.

7-phenyl-1-azabicyclo[4.2.0]octan-8-one 15

Following a procedure of König *et al.*,[6] an oven dried Schlenk tube was charged with N'-(2-oxo-1-phenyl-2-(piperidin-1-yl)ethylidene)-tosylhydrazide (300 mg, 778 μmol, 1.0 eq.) and Cs_2CO_3 (254 mg, 778 μmol, 1.0 eq.) and evacuated and backfilled with argon three times. Anhydrous acetonitrile (10 mL) was added and the mixture was subsequently irradiated at 365 nm while stirring vigorously for 72 h until the tosylhydrazone was fully consumed. The crude product was adsorbed onto Celite®, filtered over a silica plug (SiO_2, PE/EtOAc 1:1, 200 mL) and further purified *via* flash column chromatography (SiO_2, PE/EtOAc 1:0 → 1:1 → 1:3). The reaction yields a mixture of two inseparable diastereomers in a ratio of **15a/15b** = 3.5:1 as a colourless crystalline solid (100 mg, 497 μmol, 64 %). The data for each isomer are given below.

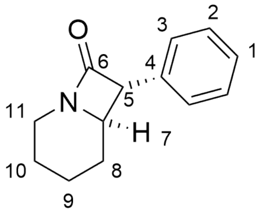

15a

^{1}**H NMR** (300 MHz, $CDCl_3$) δ [ppm] = 7.35 – 7.27 (m, 5H, H-1, H-2, H-3), 3.97 (d, J = 2.2 Hz, 1H, H-5), 3.93 – 3.90 (m, 1H, H-11), 3.41 – 3.33 (m, 1H, H-7), 2.86 – 2.75 (m, 1H, H-11), 2.22 – 2.14 (m, 1H, H-8), 1.98 – 1.88 (m, 1H, H-9), 1.74 – 1.66 (m, 1H, H-10), 1.46 – 1.44 (m, 1H, H-8), 1.43 – 1.41 (m, 1H, H-10), 1.41 – 1.34 (m, 1H, H-9).
^{13}**C{^{1}H} NMR** (75 MHz, $CDCl_3$) δ [ppm] = 166.5 (C_q, C-6), 135.9 (C_q, C-4), 128.9 (CH, C-1/C-2), 128.7 (CH, C-1/C-2), 127.5 (CH, C-3), 63.7 (CH, C-5), 57.0 (CH, C-7), 39.2 (CH_2, C-11), 30.7 (CH_2, C-8), 24.7 (CH_2, C-10), 22.4 (CH_2, C-9).
HRMS (EI+): m/z for $C_{13}H_{15}NO^+$ [M^+] calcd: 201.1149, found: 201.1160.
Spectroscopic data are in accordance with those previously reported in literature.[9]

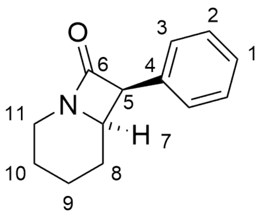

15b

^{1}**H NMR** (300 MHz, $CDCl_3$) δ [ppm] = 7.36 (t, J = 1.5 Hz, 2H, H-2/H-3), 7.32 – 7.30 (m, 2H, H-2/H-3), 7.25 – 7.22 (m, 1H, H-1), 4.61 (dd, J = 5.3, 2.1 Hz, 1H, H-5), 3.91 – 3.88 (m, 1H, H-11), 3.69 (dt, J = 11.2, 4.8 Hz, 1H, H-7), 2.77 – 2.69 (m, 1H, H-11), 1.67 – 1.61 (m, 1H, H-10), 1.53 – 1.44 (m, 4H, H-8, H-9, H-10), 1.05 – 0.72 (m, 1H, H-8).
^{13}**C{^{1}H} NMR** (75 MHz, $CDCl_3$) δ [ppm] = 128.6 (CH, C-2/C-3), 127.5 (CH, C-2/C-3), 127.3 (CH, C-1), 58.8 (CH, C-5), 52.9 (CH, C-7), 38.9 (CH_2, C-11), 27.1 (CH_2, C-8), 25.1 (CH_2, C-10), 22.0 (CH_2, C-9).

Methyl 2-phenyl-2-(piperidin-2-yl)acetate hydrochloride 22

Following a modified procedure of Lapinsky *et al.*,[4] a Schlenk flask was charged with β-lactam 7-phenyl-1-azabicyclo[4.2.0]octan-8-one **15** (60 mg, 298 μmol, 1.0 eq.) and evacuated and backfilled with argon three times. To this, dry MeOH (15 mL) and 2 M HCl in Et_2O (22 mL) was

added and the reaction mixture was stirred at 55 °C for 7 h. Upon cooling to room temperature, the volatile components were removed *in vacuo*. The crude product was washed with EtOAc (3x15 mL) and pentane (5x20 mL) and dried under reduced pressure. The product **22** was obtained as a colourless crystalline solid (80 mg, 297 μmol, 99 %).

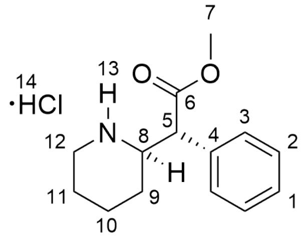

^{1}**H NMR** (400 MHz, $CDCl_3$) δ [ppm] = 10.63 (s, 1H, H-13/H-14), 8.49 (s, 1H, H-13/H-14), 7.39 – 7.39 (m, 1H, H-1), 7.34 – 7.33 (m, 2H, H-2), 7.28 – 7.27 (m, 2H, H-3), 4.35 (d, J = 8.7 Hz, 1H, H-5), 3.80 (s, 3H, H-7), 3.68 – 3.66 (m, 1H, H-12), 3.64 – 3.60 (m, 1H, H-8), 2.98 – 2.92 (m, 1H, H-12), 2.12 – 2.07 (m, 1H, H-11), 1.86 – 1.82 (m, 1H, H-11), 1.82 – 1.80 (m, 1H, H-10), 1.77 – 1.74 (m, 1H, H-9), 1.40 – 1.36 (m, 1H, H-9), 1.36 – 1.32 (m, 1H, H-10).
^{13}**C{^{1}H} NMR** (101 MHz, $CDCl_3$) δ [ppm] = 172.6 (C_q, C-6), 134.2 (C_q, C-4), 129.8 (CH, C-1), 129.4 (CH, C-2), 128.6 (CH, C-3), 59.3 (CH, C-8), 54.0 (CH, C-5), 53.7 (CH_3, C-7), 46.0 (CH_2, C-12), 26.0 (CH_2, C-9), 22.7 (CH_2, C-10), 22.1 (CH_2, C-11).
HRMS (EI+): m/z for $C_{14}H_{18}NO_2^+$ [$(M\text{-}H)^+$] calcd: 232.1330, found: 232.1390.

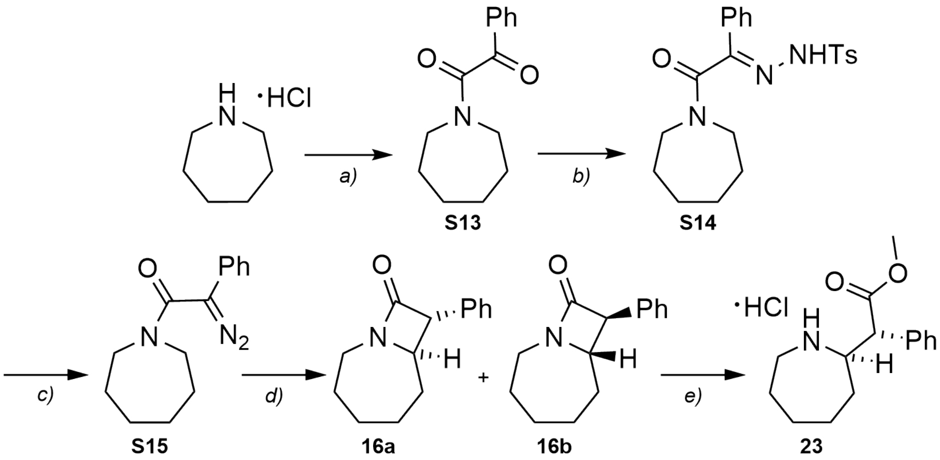

Figure S7: Conditions for the the synthesis of **16**: (a) pyridine (2.2 eq.), phenylglyoxalic acid (1.0 eq.), DMF (1.7 eq.), oxalyl chloride (1.5 eq.), CH_2Cl_2, 0 °C → RT, 16 h, 74 %; (b) tosyl hydrazide, H_2SO_4, EtOH, 85 °C, 16 h, 54 %; (c) NaH (1.0 eq.), DME, Aliquat® 336, RT → 60 °C, 4 h, 80 %; (d) C_6H_6, hν 390 nm, 1.5 h, 60 %, **16a/16b** 4:1; e) HCl (3 M in MeOH), 85 °C, 7 h, 87 %.

1-(azepan-1-yl)-2-phenylethane-1,2-dione S13

Following a modified procedure by He *et al.*,[3] in a flame-dried Schlenk flask phenylglyoxylic acid (1.77 g, 11.8 mmol, 1.0 eq.) was dissolved in anhydrous dichloromethane (30 mL). To this, DMF (2.0 mL, 20.0 mmol, 1.7 eq.) was added and the mixture was cooled to 0 °C. Oxalyl chloride (1.5 mL, 17.7 mmol, 1.5 eq.) was added dropwise and the reaction mixture was stirred at 0 °C for 30 min. With an external vacuum pump and cooling trap, the solvent was removed, resulting in the acid chloride as a viscous yellow liquid. In a second flame-dried Schlenk flask,

azepane (1.86 mL, 16.5 mmol, 1.4 eq.) and dry pyridine (2.0 mL, 25.9 mmol, 2.2 eq.) was dissolved in anhydrous dichloromethane (10 mL) and cooled to 0 °C. The acid chloride was redissolved in 10 mL anhydrous dichloromethane and added dropwise to the second flask under strong stirring. The mixture was warmed to room temperature and stirred overnight resulting in a dark red solution. Purification *via* flash column chromatography (SiO_2, PE/EtOAc 2:1 → 1:1) afforded the product **S13** as a faintly yellow oil (2.02 g, 8.73 mmol, 74 %).

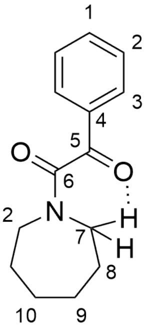

^{1}**H NMR** (600 MHz, $CDCl_3$) δ [ppm] = 7.95 (d, J = 7.1 Hz, 2H, H-3), 7.63 (t, J = 7.4 Hz, 1H, H-1), 7.51 (t, J = 7.9 Hz, 2H, H-2), 3.71 – 3.66 (m, 2H, H-7), 3.37 – 3.32 (m, 2H, H-12), 1.89 – 1.82 (m, 2H, H-8), 1.72 – 1.67 (m, 2H, H-11), 1.67 – 1.63 (m, 2H, H-9), 1.65 – 1.56 (m, 2H, H-10).

^{13}**C{^{1}H} NMR** (151 MHz, $CDCl_3$) δ [ppm] = 192.0 (C_q, C-5), 167.3 (C_q, C-6), 134.7 (CH, C-1), 133.4 (C_q, C-4), 129.8 (CH, C-3), 129.1 (CH, C-2), 48.1 (CH_2, C-12), 45.3 (CH_2, C-7), 29.2 (CH_2, C-11), 27.8 (CH_2, C-9), 27.4 (CH_2, C-8), 26.7 (CH_2, C-10).

Spectroscopic data are in accordance with those previously reported in literature.[10]

N'-(2-(azepan-1-yl)-2-oxo-1-phenylethylidene)-tosylhydrazide S14

Following a modified procedure by Lapinsky *et al.*,[4] tosyl hydrazide (1.95 g, 10.5 mmol, 1.2 eq.) was dissolved in EtOH (50 mL). To this suspension, H_2SO_4 (2 mL) was added, until everything had dissolved. Upon addition of 1-(azepan-1-yl)-2-phenylethane-1,2-dione **S13** (2.02 g, 8.73 mmol, 1.0 eq.), the reaction mixture was stirred at 85 °C overnight. After cooling to -5 °C, the precipitate was filtered off and dried *in vacuo* affording the product **S14** as a colourless crystalline solid (1.87 g, 4.68 mmol, 54 %).

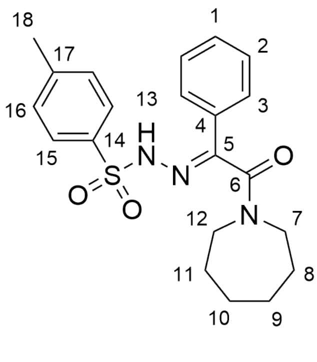

^{1}**H NMR** (600 MHz, $CDCl_3$) δ [ppm] = 8.08 (s, 1H, H-13), 7.86 (d, J = 8.5 Hz, 2H, H-15), 7.59 (dd, J = 8.2, 1.5 Hz, 2H, H-3), 7.37 – 7.37 (m, 3H, H-1, H-2), 7.32 – 7.27 (m, 2H, H-16), 3.67 (t, J = 5.9 Hz, 2H, H-7/H-12), 3.23 (t, J = 5.9 Hz, 2H, H-7/H-12), 2.39 (s, 3H, H-18), 1.87 – 1.80 (m, 2H, H-8/H-11), 1.66 – 1.61 (m, 2H, H-9/H-10), 1.56 – 1.50 (m, 4H, H-8/H-11, H-9/H-10).

^{13}**C{^{1}H} NMR** (151 MHz, $CDCl_3$) δ [ppm] = 163.9 (C_q, C-6), 151.7 (C_q, C-5), 144.5 (C_q, C-14), 135.1 (C_q, C-17), 132.2 (C_q, C-4), 130.8 (CH, C-1), 129.8 (CH, C-16), 129.0 (CH, C-2), 128.2 (CH, C-15), 126.4 (CH, C-3), 48.7 (CH_2, C-7/C-12), 45.3 (CH_2, C-7/C-12), 28.7 (CH_2, C-8/C-11), 28.0 (CH_2, C-9/C-10), 27.6 (CH_2, C-8/C-11), 26.6 (CH_2, C-9/C-10), 21.8 (CH_3, C-18).

HRMS (EI+): m/z for $C_{14}H_{18}N_3O^+$ [(M-Ts)$^+$] calcd: 244.1445, found: 244.1457.

1-(azepan-1-yl)-2-diazo-2-phenylethan-1-one S15

Adapting a procedure of Corey *et al.*,[5] a flame-dried Schlenk flask was charged with NaH (42 mg, 1.75 mmol, 1.0 eq.) and dry dimethoxyethane (18 mL). To this a suspension of *N*'-(2-(azepan-1-yl)-2-oxo-1-phenylethylidene)-tosylhydrazide **S14** (700 mg, 1.75 mmol, 1.0 eq.) and Aliquat® 336 (4 drops) was added. The reaction mixture was stirred at room temperature until formation of H_2-gas ceased (20 min), then heated to 60 °C and stirred for 4 h. The initially pale yellow and clear solution changed to a bright orange solution with a colourless precipitate. The reaction mixture was cooled to room temperature, filtered over Celite®, eluted with Et_2O and removed the solvent *in vacuo*. The product **S15** was used without further purification as it is prone to decomposition (92 mg, 430 μmol, 80 %).

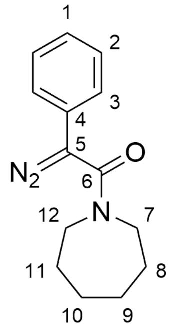

^{1}**H NMR** (600 MHz, $CDCl_3$) δ [ppm] = 7.40 – 7.29 (m, 3H, H-3, H-1), 7.25 – 7.17 (m, 2H, H-2), 3.53 – 3.41 (m, 2H, H-7/H-12), 3.21 (t, J = 8.5 Hz, 2H, H-7/H-12), 2.00 – 1.85 (m, 2H, H-8/H-11), 1.80 – 1.74 (m, 1H, H-8/H-11), 1.73 – 1.66 (m, 1H, H-8/H-11), 1.57 – 1.51 (m, 4H, H-9, H-10).

^{13}**C{^{1}H} NMR** (151 MHz, $CDCl_3$) δ [ppm] = 167.6 (C_q, C-5), 165.1 (C_q, C-6), 135.8 (C_q, C-4), 128.9 (CH, C-3), 128.2 (CH, C-2), 127.5 (CH, C-1), 61.5 (CH_2, C-7/C-12), 48.3 (CH_2, C-7/C-12), 29.4 (CH_2, C-8/C-11), 28.5 (CH_2, C-8/C-11), 28.4 (CH_2, C-9/C-10), 22.7 (CH_2, C-9/C-10). **HRMS (EI+):** m/z for $C_{14}H_{17}NO^+$ [$(M-N_2)^+$] calcd: 215.1305, found: 215.1311.

IR (ATR): $\tilde{\nu}(N_2) = 2054\ cm^{-1}$.

8-phenyl-1-azabicyclo[5.2.0]nonan-9-one 16

Adapting a procedure of Corey *et al.*,[5] a vial was charged with 1-(azepan-1-yl)-2-diazo-2-phenylethan-1-one **S5** (77 mg, 320 μmol, 1.0 eq.) and evacuated and backfilled with argon three times. Anhydrous benzene (5 mL) was added and the reaction mixture was irradiated by a 390 nm LED light for 1.5 h. The crude product was purified *via* flash column chromatography (SiO_2, PE/EtOAc 1:1). The reaction yields a mixture of two diastereomers in a ratio of **16a/16b** = 4:1, affording the product **16** as a yellow oil (41 mg, 190 μmol, 60 %).

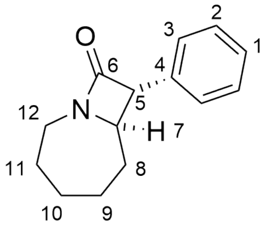

16a

^{1}**H NMR** (600 MHz, $CDCl_3$) δ [ppm] = 7.36 – 7.32 (m, 2H, H-3), 7.27 (d, J = 7.1 Hz, 3H, H-1, H-2), 3.82 (s, 1H, H-5), 3.65 (dt, J = 9.8, 2.4 Hz, 1H, H-7), 3.47 (t, J = 13.2 Hz, 1H, H-12), 3.42 – 3.37 (m, 1H, H-12), 2.23 – 2.17 (m, 1H, H-8), 1.97 – 1.84 (m, 3H, H-9, H-10, H-11), 1.65 – 1.59 (m, 1H, H-8), 1.59 – 1.54 (m, 1H, H-11), 1.46 – 1.38 (m, 2H, H-9, H-10).

^{13}**C{^{1}H} NMR** (151 MHz, $CDCl_3$) δ [ppm] = 167.6 (C_q, C-6), 135.8 (C_q, C-5), 128.9 (CH, C-3), 127.5 (CH, C-2), 127.5 (CH, C-1), 62.6 (CH, C-7), 59.7 (CH, C-5), 43.1 (CH_2, C-12), 35.2 (CH_2, C-8), 29.7 (CH_2, C-10, 28.4 (CH_2, C-9), 27.1 (CH_2, C-11).

HRMS (EI+): m/z for $C_{14}H_{17}NO^+$ [M^+] calcd: 215.1305, found: 215.1291.

Methyl-2-(azepan-2-yl)-2-phenylacetate hydrochloride 23

Following a modified procedure of Lapinsky *et al.*,[4] a Schlenk flask was charged with β-lactam 8-phenyl-1-azabicyclo[5.2.0]nonan-9-one **16** (35 mg, 160 μmol, 1.0 eq.) and evacuated and backfilled with argon three times. To this, 3 M HCl in MeOH (7 mL) was added and the reaction mixture was stirred at 85 °C for 7 h. Upon cooling to room temperature, the volatile components were removed *in vacuo*. The crude product was washed with EtOAc (3x7mL) and pentane (5x10 mL) and dried under reduced pressure. The product was obtained as a colourless crystalline solid (40 mg, 140 μmol, 87 %).

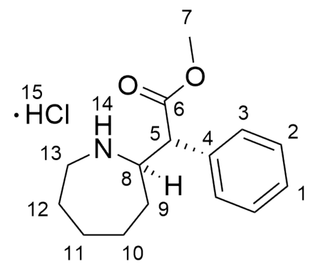

^{1}**H NMR** (600 MHz, $CDCl_3$) δ [ppm] = 10.07 (bs, 1H, H-14/H-15), 8.99 (bs, 1H, H-14/H-15), 7.39 – 7.30 (m, 3H, H-1, H-2), 7.30 – 7.26 (m, 2H, H-3), 4.29 (d, J = 9.6 Hz, 1H, H-5), 3.96 – 3.91 (m, 1H, H-8), 3.77 (s, 3H, H-7), 3.47 – 3.43 (m, 1H, H-13), 3.35 – 3.32 (m, 1H, H-13), 2.14 – 2.12 (m, 1H, H-12), 1.97 – 1.93 (m, 1H, H-12), 1.87 – 1.79 (m, 2H, H-9), 1.78 – 1.70 (m, 2H, H-11), 1.49 – 1.44 (m, 2H, H-10).

^{13}**C{^{1}H} NMR** (151 MHz, $CDCl_3$) δ [ppm] = 172.9 (C_q, C-6), 135.1 (C_q, C-4), 129.5 (CH, C-2), 128.6 (CH, C-3), 128.6 (CH, C-1), 60.5 (CH, C-8), 54.4 (CH, C-5), 53.6 (CH_3, C-7), 46.4 (CH_2, C-13), 28.3 (CH_2, C-9), 26.0 (CH_2, C-11), 25.3 (CH_2, C-12), 25.3 (CH_2, C-10).

HRMS (EI+): m/z for $C_9H_{10}O_2^+$ [$(M-C_6H_{11}N)^+$] calcd: 150.0676, found: 150.0661.

(2Z,2'Z)-2,2'-(hydrazine-1,2-diylidene)bis(2-phenyl-1-(pyrrolidin-1-yl)ethan-1-one) S16

In a N_2-filled glovebox, $Rh_2[DOSP(S)]_4$ (4.4 mg, 2.32 μmol, 0.01 eq.) was dissolved in dry CH_2Cl_2 (0.5 mL), added to a flame-dried Schlenk tube and diluted with additional dry CH_2Cl_2 (10 mL). 2-diazo-2-phenyl-1-(pyrrolidin-1-yl)ethan-1-one (50 mg, 232 µmol, 1.0 eq.) was dissolved in dry CH_2Cl_2 (1.5 mL) and added to the green solution. The solution was heated to 40 °C, stirred overnight and concentrated under reduced pressure. The crude product was purified by crystallisation from CH_2Cl_2/Et_2O and the product **S16** was afforded as a yellow crystalline solid in 19 % yield (8.9 mg, 22.4 μmol).

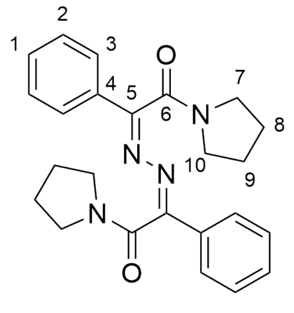

^{1}**H NMR** (600 MHz, $CDCl_3$) δ [ppm] = 7.85 – 7.82 (m, 4H, H-3), 7.50 – 7.45 (m, 2H, H-1), 7.43 – 7.38 (m, 4H, H-2), 3.77 (bs, 2H, H-8/H-11), 3.68 (bs, 2H, H-8/H-11), 3.41 (bs, 2H, H-8/H-11), 3.18 (bs, 2H, H-8/H-11), 1.98 – 1.93 (m, 4H, H-9/H-10), 1.87 – 1.86 (m, 4H, H-9/H-10).

^{13}**C{^{1}H} NMR** (151 MHz, $CDCl_3$) δ [ppm] = 165.4 (C_q, C-6), 165.0 (C_q, C-5), 132.1 (C_q, C-4), 131.8 (CH, C-1), 129.0 (CH, C-2), 128.2

(CH, C-3), 46.7 (CH_2, C-8/C-11), 45.0 (CH_2, C-8/C-11), 25.8 (CH_2, C-9/C-10), 24.7 (CH_2, C-9/C-10).

HRMS (EI+): m/z for $C_{24}H_{26}N_4O_2^+$ [$(M+H)^+$] calcd: 403.2129, found: 403.2146.

(2*E*,2'*E*)-2,2'-(hydrazine-1,2-diylidene)bis(1-(4-azabicyclo[5.2.0]nonan-4-yl)-2-(4-(tert-butoxy)phenyl)ethan-1-one) 19

In a flame-dried Schlenk flask, KO^{t}Bu (48.2 mg, 430 μmol, 3.7 eq.) and Rh_2[DOSP(*R*)]$_4$ (2.4 mg, 0.51 μmol, 0.01 eq.) were dissolved in dry toluene (12 mL). (*E*)-*N*'-(2-(4-azabicyclo[5.2.0]nonan-4-yl)-1-(4-fluorophenyl)-2-oxoethylidene)-4-methylbenzenesulfonohydrazide **17** (51.5 mg, 116 μmol, 1.0 eq.) was added under argon and the solution was stirred at 85 °C for 18 h. The crude product was purified *via* flash column chromatography (SiO_2, PE:EA, 3:1 → 2:1) giving the product **19** as a yellow crystalline solid.

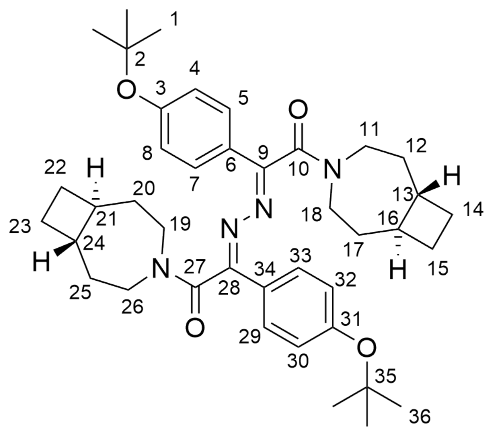

^{1}H NMR (600 MHz, C_6D_6) δ [ppm] = 8.19 – 8.06 (m, 4H, H-5, H-7, H-29, H-33), 7.04 – 6.98 (m, 4H, H-4, H-8, H-30, H-32), 4.16 – 4.06 (m, 1H, H-18/H-19), 3.97 – 3.87 (m, 1H, H-11/H-26), 3.41 (ddd, J = 14.5, 6.0, 3.4 Hz, 1H, H-18/H-19), 3.37 – 3.29 (m, 1H, H-11/H-26), 3.13 – 3.06 (m, 2H, H-11/H-26), 3.06 – 3.01 (m, 1H, H-18/H-19), 2.63 – 2.56 (m, 1H, H-18/H-19), 2.56 – 2.49 (m, 1H, H-16/H-21), 2.44 – 2.34 (m, 1H, H-17/H-20), 2.19 – 2.09 (m, 1H, H-16/H-21), 1.99 – 1.94 (m, 1H, H-12/H-25), 1.85 – 1.82 (m, 1H, H-12/H-25), 1.81 – 1.78 (m, 1H, H-12/H-25), 1.78 – 1.74 (m, 1H, H-13/H-24), 1.69 – 1.64 (m, 1H, H-13/H-24), 1.63 – 1.57 (m, 2H, H-14/H-15/H-22/H-23), 1.54 – 1.47 (m, 2H, H-17/H-20), 1.43 – 1.40 (m, 1H, H-14/H-15/H-22/H-23), 1.38 – 1.33 (m, 2H, H-14/H-15/H-22/H-23), 1.24 – 1.23 (m, 2H, H-14/H-15/H-22/H-23), 1.23 – 1.21 (m, 1H, H-14/H-15/H-22/H-23), 1.19 (d, J = 2.3 Hz, 18H, H-1, H-36), 1.19 – 1.18 (m, 1H, H-12/H-25), 1.18 – 1.17 (m, 1H, H-17/H-20).

^{13}C{^{1}H} NMR (151 MHz, C_6D_6) δ [ppm] = 166.9 (C_q, C-10/C-27), 166.7 (C_q, C-10/C-27), 165.1 (C_q, C-9/C-28), 165.0 (C_q, C-9/C-28), 164.7 (C_q, C-3/C-31), 164.6 (C_q, C-3/C-31), 159.5 (C_q, C-6, C-34), 129.8 (CH, C-5/C-7/C-29/C-33), 129.8 (CH, C-5/C-7/C-29/C-33), 129.7 (CH, C-5/C-7/C-29/C-33), 129.6 (CH, C-5/C-7/C-29/C-33), 123.5 (CH, C-4/C-8/C-30/C-32), 123.5 (CH, C-4/C-8/C-30/C-32), 123.5 (CH, C-4/C-8/C-30/C-32), 123.4 (CH, C-4/C-8/C-30/C-32), 79.0 (C_q, C-2, C-35), 51.7 (CH_2, C-18/C-19), 50.4 (CH_2, C-11/C-26), 48.1 (CH_2, C-11/C-26), 47.2 (CH_2, C-18/C-19), 42.8 (CH, C-13/C-24), 41.2 (CH, C-13/C-24), 40.9 (CH, C-16/C-21), 40.8 (CH, C-16/C-21), 34.5 (CH_2, C-12/C-25), 33.6 (CH_2, C-17/C-20), 32.7 C-12/C-25), 31.4 (CH_2, C-17/C-20), 28.9 (CH_3, C-1, C-36), 27.2 (CH_2, C-14/C-15/C-22/C-23), 26.9 (CH_2, C-14/C-15/C-22/C-23), 26.2 (CH_2, C-14/C-15/C-22/C-23), 25.7 (CH_2, C-14/C-15/C-22/C-23).

HRMS (EI+): m/z for $C_{40}H_{54}N_4O_4^+$ [$(M+H)^+$] calcd: 655.4218, found: 655.4235.

2 Supporting Crystallographic Information

Crystal data and details of the structure determinations are compiled in Tables S1 – S2. Full shells of intensity data were collected at 120(1) K with an Agilent Technologies Supernova-E CCD diffractometer (Mo-Kα or Cu-Kα radiation, microfocus X-ray tube, multilayer mirror optics). Detector frames (typically ω-, occasionally ϕ-scans, scan width 0.5 °C for Mo and 1.0 °C for Cu) were integrated by profile fitting.[11],[12] Data were corrected for air and detector absorption, Lorentz and polarization effects[12] and scaled essentially by application of appropriate spherical harmonic functions.[12],[13] Absorption by the crystal numerically (Gaussian grid, all other compounds).[12],[14] An illumination correction was performed as part of the numerical absorption correction.[13]

Using Olex2-1.5,[15] all structures were solved with SHELXT[16] (intrinsic phasing) and refined with SHELXL[17] by full-matrix least squares methods based on F^2 against all unique reflections. All non-hydrogen atoms were given anisotropic displacement parameters. Hydrogen atoms were generally input at calculated positions and refined with a riding model.[18] Split atom models were used to refine disordered groups and/or solvent molecules. When found necessary, suitable geometry and adp restraints were applied.[18],[19]

CCDC 2472879-2472882 contains the supplementary crystallographic data for this paper. These data can be obtained free of charge from the Cambridge Crystallographic Data Centre's and FIZ Karlsruhe's joint Access Service *via* https://www.ccdc.cam.ac.uk.

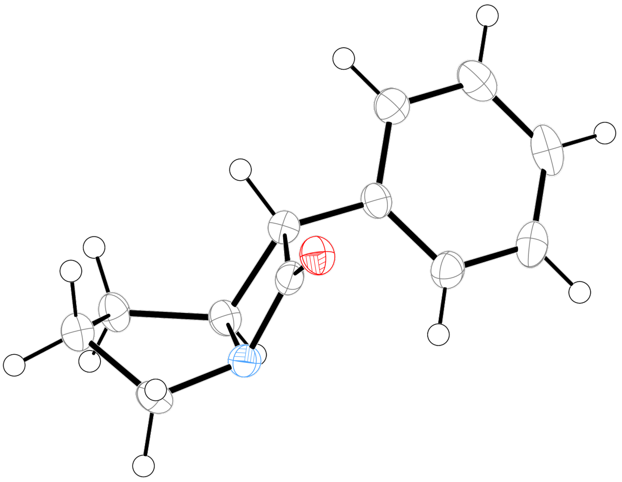

Figure S8: Molecular structure of **14a** with displacement ellipsoids drawn at 50 % probability.

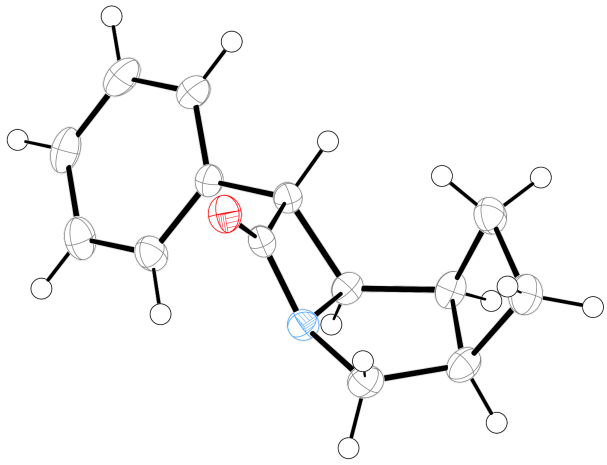

Figure S9: Molecular structure of **9a** with displacement ellipsoids drawn at 50 % probability.

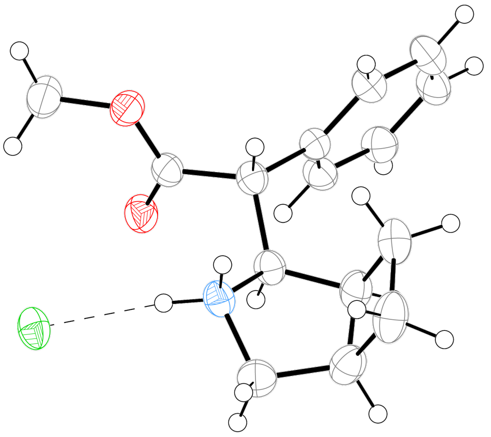

Figure S10: Molecular structure of **20** with displacement ellipsoids drawn at 50 % probability

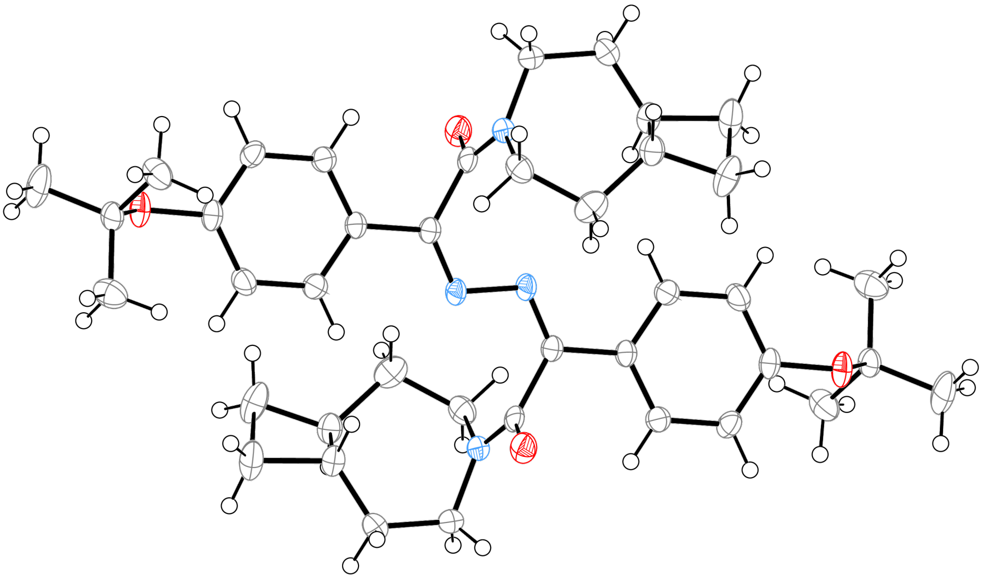

Figure S11: Molecular structure of **19** with displacement ellipsoids drawn at 50 % probability.

Table S1: Crystal data and structure refinement for **14a** and **9a**.

	**14a**	**9a**
Empirical formula	$C_{12}H_{13}NO$	$C_{14}H_{15}NO$
Formula weight	187.23	213.27
Temperature [K]	120(1)	120(1)
Crystal system	orthorombic	monoclinic
Space group	$P2_12_12_1$	$P2_1/n$
a [Å]	6.28501(19)	9.6732(2)
b [Å]	8.6397(3)	11.7720(2)
c [Å]	18.0486(5)	10.5748(3)
α [°]	90	90
β [°]	90	112.362(3)
γ [°]	90	90
Volume [Å^3]	980.05(5)	1113.63(5)
Z	4	4
ρ_{calc} [g/cm^3]	1.269	1.272
μ [mm^{-1}]	0.638	0.080
transmission factors (max, min)	1.000, 0.813	1.000, 0.475
F(000)	400.0	456.0
Radiation	Cu Kα (λ =1.54184	Mo Kα (λ = 0.71073)
2Θ range [°]	9.802 to 141.812	4.864 to 68.284
Index ranges	$-7 \leq h \leq 7$<br>$-10 \leq k \leq 10$<br>$-22 \leq l \leq 22$	$-15 \leq h \leq 15$<br>$-18 \leq k \leq 18$<br>$-16 \leq l \leq 16$
Reflections collected	13721	52148
Independent reflections	1879<br>$R_{int} = 0.0727$<br>$R_{sigma} = 0.0347$	4506<br>$R_{int} = 0.0511$<br>$R_{sigma} = 0.0238$
observed [$I \geq 2\sigma$ (I)]	12130	37823
Completeness to θ	99.5 %<br>(θ = 70.906)	98.1 %<br>(θ = 34.142)
Data/restraints/parameters	1879/0/128	4506 / 0 / 145
Goodness-of-fit on F^2	1.063	1.061
R indexes [$I \geq 2\sigma$ (I)]	$R_1 = 0.0328$<br>$wR_2 = 0.0808$	$R_1 = 0.0473$<br>$wR_2 = 0.1263$
R indexes [all data]	$R_1 = 0.0364$<br>$wR_2 = 0.0837$	$R_1 = 0.0578$<br>$wR_2 = 0.1326$
Largest diff. peak/hole [e Å^{-3}]	0.12/-0.15	0.38 / -0.20
Flack parameter	0.5(4)	-
CCDC number	2472881	2472882

Table S2: Crystal data and structure refinement for **20** and **19**.

	**20**	**19**
Empirical formula	$C_{15}H_{20}ClNO_2$	$C_{40}H_{54}N_4O_4$
Formula weight	281.78	654.87
Temperature [K]	120(1)	120(1)
Crystal system	monoclinic	monoclinic
Space group	$P2_1/n$	$P2_1/c$
a [Å]	9.0885(2)	9.3539(2)
b [Å]	10.2499(2)	18.2239(3)
c [Å]	15.6126(3)	11.1632(3)
α [°]	90	90
β [°]	92.775(2)	109.134(2)
γ [°]	90	90
Volume [$Å^3$]	1452.71(5)	1797.80(7)
Z	4	2
ρ_{calc} [g/cm^3]	1.288	1.210
μ [mm^{-1}]	2.308	0.616
transmission factors (max, min)	1.000, 0.766	1.000, 0.825
F(000)	600.0	708.0
Radiation	Cu Kα (λ =1.54184)	Cu Kα (λ =1.54184)
2Θ range [°]	10.328 to 142.976	9.69 to 142.48
Index ranges	$-10 \leq h \leq 11$<br>$-12 \leq k \leq 12$<br>$-19 \leq l \leq 19$	$-11 \leq h \leq 11$<br>$-22 \leq k \leq$<br>$-13 \leq l \leq 13$
Reflections collected	20443	40108
Independent reflections	2807<br>$R_{int} = 0.0526$<br>$R_{sigma} = 0.0257$	3477<br>$R_{int} = 0.0680$<br>$R_{sigma} = 0.0237$
observed [$I \geq 2\sigma$ (I)]	16383	31573
Completeness to θ	99.1 %<br>($\theta = 71.488$)	99.5 %<br>($\theta = 71.240$)
Data/restraints/parameters	2807/0/181	3477/93/288
Goodness-of-fit on F^2	1.042	1.045
R indexes [$I \geq 2\sigma$ (I)]	$R_1 = 0.0463$<br>$wR_2 = 0.1256$	$R_1 = 0.0396$<br>$wR_2 = 0.1008$
R indexes [all data]	$R_1 = 0.0528$<br>$wR_2 = 0.1330$	$R_1 = 0.0446$<br>$wR_2 = 0.1052$
Largest diff. peak/hole [e $Å^{-3}$]	0.72/-0.29	0.22/-0.20
Flack parameter	-	-
CCDC number	2472879	2472880

3 Computational Details

3.1 General Considerations

In order to elucidate the identity of the isomeric mixtures of the tricyclic and bicyclic β-lactam compounds, all possible stereoisomers of **9** and **11–16** were subjected to a computational chemistry investigation. For the tricyclic compounds based on pyrrolidine and piperidine rings (**9**, **11** and **12**), only compounds with a *cis*-configuration at the cyclobutane ring were considered, while for **13**, only compounds with a *cis*-configuration of the cyclobutane ring were analysed. This configuration is in line with the crystallographically confirmed stereochemistry of the cyclobutane ring obtained via the iron-based [2+2]-cycloaddition reaction.[2]

The conformer/ rotamer ensemble was generated using the CREST algorithm, using the GFN-xTB tight-binding method.[20] The ensemble was then subjected to the *Commanline Energetic Sorting*[21] algorithm (CENSO 2.1.3) with the following settings: (a) **Pre-screening**: Single point energies at a pbe/def2-sv(p) level of theory, then xtb_gsolv, threshold: 6 kcal/mol (b) **Screening**: r2scan-3c, threshold 3.5 kcal/mol, (c) **Optimisation**: r^{2}scan-3c RI level of theory, including solvation through the CPCM Model, threshold 3.0 kcal/mol (d) **Refinement**: ωB97x-v/def2-tzvp level of theory, including solvation through the CPCM model;[22] (d) **NMR Calculation** (Orca 6.0.1),[23] using pcSseg-3 basis set for both the shieldings and the spin-spin coupling (for an input model, see below).

The chemical shieldings obtained were converted to the corresponding chemical shifts by subtracting them from the shielings or $SiMe_4$ calculated at the same level of theory under the same solvation conditions (Eq. S1).

$$\delta_i = \sigma_{ref} - \sigma_i \qquad \text{(S1)}$$

The spin-spin coupling calculations and the Boltzmann averaging of the chemical shifts were performed using the ANMR module.[24] The spectra were plotted using the software nmrplot, developed by F. Bohle (Universität Bonn).[24]

3.2 Conformer Analysis

Following the CREST and CENSO algorithms, for each stereoisomer, conformers within a 2.5 kcal/mol window were kept for the NMR analysis. A summary is given in Table S3 below.

Table S3: Number of conformers obtained with the CENSO workflow for compounds **9–16** (including corresponding isomers) and Boltzmann-weighted population of the dominant conformer at 298 K

**Compound**	**Number of conformers after the CREST procedure**	**Number of conformers after the CENSO procedure**	**% dominant conformer after Boltzmann averaging (RT)**
**9a**	4	2	77.57 %
**9b**	6	3	100 %
**9c**	2	1	100 %
**9d**	1	1	100 %
**11a**	6	2	73.88 %
**11b**	4	2	75.90 %
**11c**	2	1	100 %
**11d**	2	1	100 %
**12a**	6	3	90.95 %
**12b**	8	4	75.41 %
**12c**	3	2	99.07 %
**12d**	3	1	100 %
**13a**	2	1	100 %
**13b**	3	1	100 %
**13c**	1	1	100 %
**13d**	1	1	100 %
**14a**	4	2	70.39 %
**14b**	2	1	100 %
**15a**	7	1	100 %
**15b**	3	1	100 %
**16a**	17	4	71.89 %
**16b**	7	2	97.77 %

3.3 Functional Benchmarking for NMR Calculations

For selecting the best functional, a benchmarking study was conducted on structurally characterized compounds **9a** and **14**, against experimentally collected spectroscopic data, using the pcsseg-3 basis set. A sample input for the NMR calculation of each conformer is given below.

Sample input for ORCA NMR calculations

```
! pcsseg-3 cc-pwCVQZ/C DEFGRID2 scfconv7  printgap
! CPCM(chloroform) # change accordingly
%pal
nprocs 4  # change accordingly
end
%output
printlevel normal
end

! wB97m-v # change accordingly. for dlpno-mp2, NoFrozenCore was
    used

* xyzfile 0 1 [filename].xyz

%eprnmr
origin giao
giao_2el giao_2el_same_as_scf
giao_1el giao_1el_analytic
SpinSpinRThresh 8.0000
Nuclei = all H { shift,ssfc }
Nuclei = all C { shift,ssfc }
End
```

Statistical analysis on the collected datasets for the Boltzmann averaged conformers was performed for each compound considered (**9** and **14**). Calculation of the Mean Average Error (MAE, Eq. S2) and Root-Mean Squared Error (RMSE, Eq. S3) on all data, before and after applying the regression law (scaling) was used to determine the best functional.[25] The data are summarised in Tables S4 and S5.

Table S4: Benchmark of calculated ^{13}C NMR chemical shifts for compound **14** and **9** using B3LYP, PBE0, TPSS0 and TPSSh; experimental values, absolute errors, MAE and RMSE are reported.

	**Exp.**	**Calculated**							
**C_Nr.**	**Comp. 14**	**B3LYP**	Err	**PBE0**	Err	**TPSS0**	Err	**TPSSh**	Err
**1**	127.4	139.189	0.06	131.724	0.06	136.056	0.06	134.43	0.06
**2**	127.7	151.483	0.03	142.95	0	147.241	0.03	146.315	0.02
**3**	129	139.189	0.06	131.724	0.06	136.056	0.06	134.43	0.06
**4**	126.9	140.145	0.01	132.308	0.01	136.471	0.01	134.877	0.01
**5**	61.6	70.707	1.35	64.043	1.35	68.158	1.25	69.759	1.31
**6**	175.6	192.629	1.16	182.536	1.18	190.056	1.15	187.46	1.17
**7**	60.6	72.891	0.37	66.226	0.34	70.659	0.33	72.33	0.32
**8**	30.2	36.245	0.93	31.187	0.81	35.564	0.84	36.447	0.85
**9**	29.9	36.247	0.97	31.324	0.89	35.678	0.83	36.712	0.87
**10**	45.8	52.804	1.11	47.627	1.07	52.622	1.02	53.712	1.03
	**Comp. 9**								
1	127.5	138.676	0.29	138.651	0.29	135.17	0.26	137.129	0.24
**2**	129	140.606	0.1	140.559	0.09	136.901	0.08	138.937	0.1
**3**	127.7	139.507	0.6	139.775	0.61	136.36	0.58	138.412	0.55
**4**	136.1	152.298	0.41	151.474	0.37	148.057	0.35	150.79	0.35
**5**	55.6	64.9	0.82	63.774	0.74	63.198	0.7	68.254	0.71
**6**	174.8	193.914	0.11	192.664	0.14	191.201	0.14	192.095	0.13
**7**	63.9	75.366	0.61	73.658	0.56	73.203	0.54	78.546	0.55
**8**	39.8	46.554	0.08	45.224	0.09	45.625	0.09	50.739	0.15
**9**	18.9	24.176	0.15	23.294	0.14	24.363	0.12	28.997	0.11
**10**	24.4	29.813	0	28.489	0	28.986	0.01	33.601	0.01
**11**	45.4	53.965	0.21	52.161	0.21	52.077	0.19	57.366	0.17
**12**	50.5	55.228	0.11	54.828	0.12	54.922	0.11	59.357	0.1
**MAE (all)**		10.37		7.14		8.24		10.29	
**RMSE (all)**		11.50		8.59		9.22		10.97	
**MAE (all, after scaling)**		2.13		3.45		2.18		2.96	
**RMSE (all, after scaling)**		3.41		4.43		3.39		4.13	
**MAE (aliphatic only)**		7.69		4.60		6.54		9.94	
**RMSE (aliphatic only)**		8.04		5.29		6.75		10.23	
**MAE (aliphatic only, after scaling)**		0.91		1.90		0.74		1.75	
**RMSE (aliphatic, after scaling)**		1.28		2.07		1.08		1.91	

MAE = Mean-absolute error (Eq S2)

RMSE = Root Mean Squared Error (Eq. S3)

Table S5: Benchmark of calculated ^{13}C NMR chemical shifts for compound **14** and **9** using ωB97M-V, ωB97, DLPNO-MP2 and PBE; statistical error metrics relative to experiment are listed.

	**Exp.**	**Calculated**							
**C_Nr.**	**Comp. 14**	**ωB97M-V**	Err.	**ωB97**	Err	**MP2**	Err	**PBE**	Err
**1**	127.4	138.41	0.06	140.375	0.07	139.761	0.08	137.179	0.04
**2**	127.7	149.546	0.03	151.369	0.02	149.807	0.05	149.539	0
**3**	129	138.41	0.06	140.375	0.07	139.761	0.08	137.179	0.04
**4**	126.9	139.44	0	141.364	0	140.428	0.01	138.041	0.02
**5**	61.6	66.938	1.28	65.905	1.16	60.763	1.01	71.726	1.52
**6**	175.6	189.784	1.07	193.38	1.08	197.907	1.03	186.59	1.24
**7**	60.6	68.083	0.35	67.32	0.36	62.955	0.36	74.1	0.39
**8**	30.2	33.868	0.84	33.793	0.85	31.668	0.83	36.976	1
**9**	29.9	33.394	0.94	33.111	0.83	31.162	0.72	37.214	1.03
**10**	45.8	49.766	1.04	49.622	1.03	46.406	1.01	53.935	1.05
	**Comp. 9**								
**1**	127.5	138.169	0.31	139.924	0.32	138.896	0.4	147.663	0.24
**2**	129	139.941	0.08	141.849	0.06	140.858	0.02	149.779	0.12
**3**	127.7	138.759	0.61	140.713	0.61	139.981	0.69	148.881	0.58
**4**	136.1	150.45	0.47	152.259	0.41	150.619	0.45	161.717	0.35
**5**	55.6	61.549	0.77	61.041	0.76	56.08	0.69	77.477	0.8
**6**	174.8	191.322	0.14	194.983	0.18	199.761	0.11	198.803	0.14
**7**	63.9	70.579	0.53	69.785	0.48	65.006	0.55	87.97	0.59
**8**	39.8	42.639	0.05	41.666	0.05	38.791	0.03	58.959	0.13
**9**	18.9	21.399	0.13	21.216	0.12	20.632	0.1	36.037	0.16
**10**	24.4	27.031	0.01	26.565	0	25.31	0.02	41.396	0.01
**11**	45.4	49.571	0.2	47.896	0.18	45.028	0.14	66.37	0.24
**12**	50.5	52.702	0.13	52.746	0.13	49.394	0.11	67.246	0.11
**MAE (all)**		7.88		8.59		7.24		15.75	
**RMSE (all)**		9.48		10.75		10.75		17.13	
**RMSE (all, after scaling)**		**1.61**		**1.69**		2.43		5.82	
**RMSE (all, after scaling)**		**2.97**		**2.94**		3.36		6.54	
**MAE (aliphatic only)**		4.24		3.67		1.10		15.23	
**RMSE (aliphatic only)**		4.56		3.99		1.28		16.27	
**MAE (aliphatic only, after scaling)**		0.75		**0.67**		0.85		4.38	
**RMSE (aliphatic, after scaling)**		0.97		**0.67**		1.08		4.73	

MAE = Mean-absolute error (Eq S2)

RMSE = Root Mean Squared Error (Eq. S3)

$$\text{MAE} = \frac{1}{N}\sum_{i=1}^{N} |\delta_{\text{calc},i} - \delta_{\text{exp},i}| \tag{S2}$$

$$\text{RMSE} = \sqrt{\frac{1}{N}\sum_{i=1}^{N} (\delta_{\text{calc},i} - \delta_{\text{exp},i})^2} \tag{S3}$$

Summary:
The analysis conducted above clearly indicates that the best suitable method for the calculation of chemical shifts is ωB97m-v (ωB97 comes close). Since in discerning the various isomers, the aliphatic chemical shifts have a higher relevance, the analysis was also conducted only taking carbon atoms with δ<80 ppm into consideration, where ωB97m-v and ωB97 outperformed again all the other DFT and MP2 functionals. Numerically, ωB97m-v had the lowest MAE on scaled data (1.61), while ωB97 performed slightly better than ωB97m-v when only the aliphatic carbon atoms were considered (0.67 ppm vs 0.97 ppm).

After applying all the regression laws extracted from the analysis of the data above, ωB97m-v was selected as the functional of choice for the remaining calculations, due to the following reasons:

(a) ωB97M-V is a range-separated hybrid meta-GGA with 100 % exact exchange at long range and kinetic-energy–density terms in the short range. The long-range part cures self-interaction error, preventing the chronic over-deshielding of carbonyl and quaternary σ-carbons that is observed with ωB97.

(b) ωB97M-V's prediction/experiment relationship is almost perfectly linear across the entire shift range, so one global slope/intercept performs reasonably.

(c) The intercept is close to 0, suggesting that the functional is not so prone to systemic bias compared to the other functionals benchmarked.

(d) Its relatively low computational cost (compared to MP2 and double hybrid approaches) allows rapid screening of multiple possible isomers of the compounds herein described.

Following the benchmarking presented in Tables S4 - S5 the following global linear regression law was used for scaling the ωB97m-v chemical shifts (to give the predicted chemical shift $\delta_{pred.}$) and was further used in the global DP4 analysis presented below.

$$\delta_{pred.} = 0.92 \cdot \delta_{\omega B97M-v} - 0.28 \tag{S4}$$

3.4 Statistical Analysis for Isomer Assignment

Following the benchmarking study, all Boltzmann-averaged conformers (298.15 K) of all stereoisomers considered for compounds **9**, **10–16** were calculated at the ωB97m-v/pcsseg-3 level of theory employing the CPCM (benzene or chloroform) solvation model. The resulting chemical shifts were then compared to the observed experimental isomers in order to facilitate their assignment, complementing the NOESY data where available.

For the statistical analysis, two approaches were used, following the DP4 model of Smith and Goodman.[26] The calculation of these two approaches was automatised using the script given in section **6**.

(a) **A global approach** ("_*glb*" in Tables S7, S9, S11, S13, S15, S17, S19). The calculated chemical shifts were subjected to linear scaling using the regression law obtained from the benchmarking study for ωB97m-v (Eq. S4), to compensate for systematic basic-sets errors. The MAE and RMSE calculated for scaled data were then compared to the experimental ones for the species in question. The obtained metrics are converted into probabilities (Gaussian likelihood) following a naïve-Bayes model with equal priors as follows:

(1) Scaling of each of the calculated chemical shift based on the regression law (Eq S4).

(2) Calculating the residuals (difference between the corrected calculated chemical shift and the experimental one):

$$r_i = y_{(calcd.,i)} - y_{(exp.,i)} \tag{S5}$$

(3) Assuming that each carbon atom is individual, zero-mean Gaussian with fixed standard deviation σ gives the single-point probability (p):

$$p(r_i) = \frac{1}{\sigma\sqrt{2\pi}} \exp\left[-\frac{1}{2}\cdot\left(\frac{r_i}{\sigma}\right)^2\right] \tag{S6}$$

For the current analysis, the $\sigma = 3$ was used, in accordance with previous literature on ^{13}C data.[26],[27]

The probability that all N carbon atoms in the molecule is the product (P, joint likelihood):

$$P_k = \prod_{i=1}^{N} p(r_{k,i}) = \sigma\sqrt{2\pi}^{-N} \prod_{i=1}^{N} \exp\left[-\frac{1}{2}\cdot\left(\frac{r_i}{\sigma}\right)^2\right] \tag{S7}$$

The pre-factor $\sigma\sqrt{\{2\pi\}}^{-N}$ is assumed to be identical for each isomer and therefore cancels out.

(4) The unnormalized products P_k are converted into percentages for each isomer.

$$DP4\%_{kk,global} = 100 \cdot \frac{P_k}{\sum_j P_j} \tag{S8}$$

Additionally, the MAE, RMSE and R^2 values for the global approach are given (*MAE_glb, RMSE_glb* and R^2*_glb*).

(b) **A per-isomer fit approach** ("*_fit*" in Tables S7, S9, S11, S13, S15, S17, S19)

For each calculated isomer, a new regression law is calculated based on the correlation between the calculated data and the experimentally determined chemical shift of the Species in question. Applying the ordinary least squares method, slopes (*a_fit*) and intercepts (*b_fit*) are obtained for each isomer. Following this, the residuals (r), single-point probabilities (p), joint likelihood (P) and ultimately *DP4%_fit* are calculated as described in Eq. S5-S8. Additionally, the MAE, RMSE and R^2 values for the per-isomer fit approach are given (*MAE_fit, RMSE_fit* and R^2*_fit*).

In all cases, both approaches (global and per-isomer fit) predict the same isomer.

Below, a detailed analysis of each compound investigated is given.

a. Analysis for Compound 14

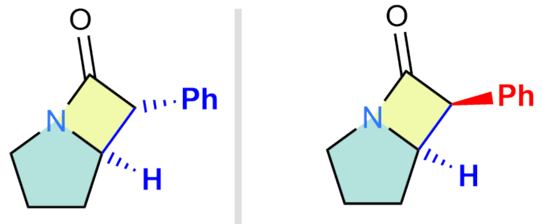

**14a** ± ***cis*-(2,3)** ***crystallised*** ***[>95%]***	**14b** ±***trans*-(2,3)** **[<5%]**

$\Delta G^{a}_{calcd.}$	0	+0.58

Figure S12: Structures of isomers A and B of compound **14** with key stereochemical differences indicated. The observed ratio and the relative energy differences between the isomers (calculated at CCSD(T)/aug-cc-cVQZ // r^2scan-3 level of theory) is indicated.

Table S6: Experimental versus calculated ^{13}C chemical shifts for compound **14** at the ωB97m-V/pcsseg-3 level of theory.

	Experimental	Calculated	
**C_Nr.**	**Species 1**	**Isomer A**	**Isomer B**
**1**	127.4	138.41	137.728
**2**	127.7	149.546	139.782
**3**	129	138.41	138.897
**4**	126.9	139.44	146.693
**5**	61.6	66.938	62.436
**6**	175.6	189.784	194.493
**7**	60.6	68.083	62.778
**8**	30.2	33.868	27.672
**9**	29.9	33.394	32.794
**10**	45.8	49.766	52.284

Table S7: Per-isomer and global regression statistics and DP4 probabilities for the candidate isomers of compound **14**.

Iso	a_fit	b_fit	MAE_fit	RMSE_fit	R^2_fit	DP4%_fit	MAE_glb	RMSE_glb	R^2_glb	DP4%_glb
**A**	**0.915**	**-0.500**	**0.651**	**0.755**	**0.996**	**98.514**	**0.695**	**0.827**	**0.996**	**99.988**
**B**	0.923	1.674	2.483	2.698	0.962	1.486	1.486	3.389	0.921	0.012

Both per-isomer fit and global scaling approaches point towards **isomer A** as the most likely species.

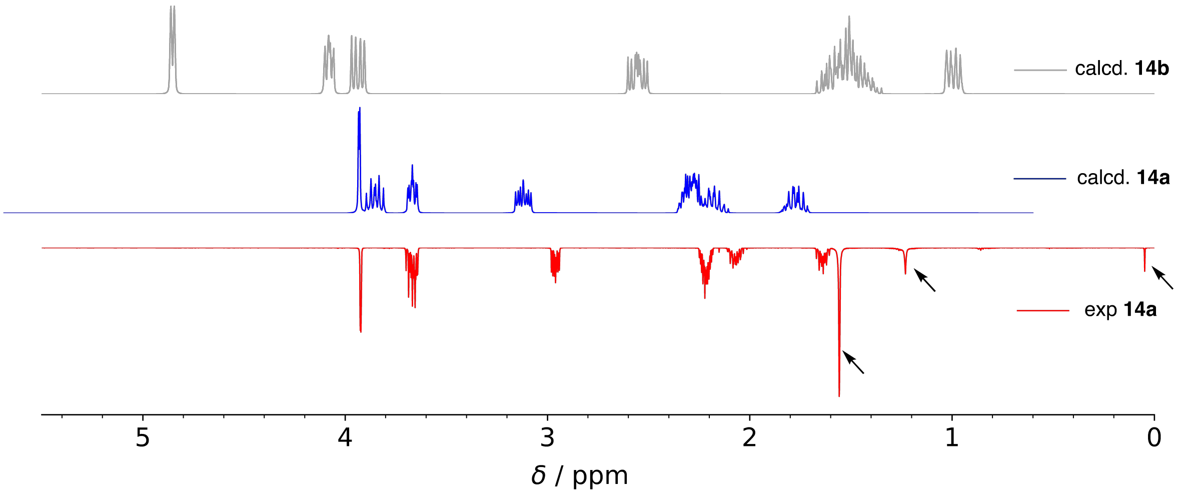

Figure S13: Experimental ^{1}H spectra of compound **14** and comparison with the calculated NMR spectra of the possible stereoisomers. Impurities in the experimental spectra are indicated with arrows.

b. Analysis for Compound 15

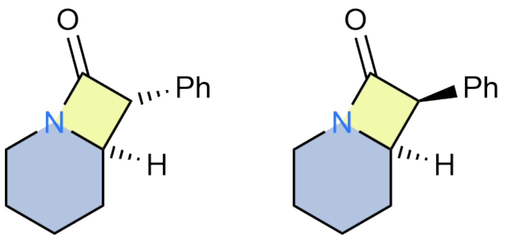

	15a	15b
d.r.	3.5	1
$\Delta G^{a}_{calcd.}$	0	+0.63

Figure S14: Structures of isomers A and B of compound **15** with key stereochemical differences indicated. The observed ratio and the relative energy differences between the isomers (calculated at CCSD(T)/aug-cc-cVQZ // r^{2}scan-3 level of theory) is indicated.

Table S8: Experimental versus calculated ^{13}C chemical shifts for compound **15** at the ωB97m-V/pcsseg-3 level of theory.

	Experimental		Calculated	
**C_Nr.**	**Species 1**	**Species 2**	**Isomer A**	**Isomer B**
**1**	128.9	127.3	138.226	138.066
**2**	128.7	128.6	139.89	139.331
**3**	127.5	127.5	138.65	140.726
**4**	135.9		150.145	147.119
**5**	63.7	58.8	69.858	64.024
**6**	166.5		180.374	180.888
**7**	57	52.9	64.071	57.409
**8**	30.7	27.1	34.22	29.944
**9**	22.4	22	25.725	25.209
**10**	24.7	25.1	28.79	29.332
**11**	39.2	38.9	42.264	42.196

Table S9: Per-isomer and global regression statistics and DP4 probabilities for the candidate isomers of compound **15**.

Iso	a_fit	b_fit	MAE_fit	RMSE_fit	R^2_fit	DP4%_fit	MAE_glb	RMSE_glb	R^2_glb	DP4%_glb
**A**	**0.929**	**-1.351**	**0.76**	**1.048**	**1**	**90.623**	**0.986**	**1.182**	**0.999**	**95.712**
**B**	0.913	1.553	1.77	2.193	0.998	9.377	2.001	2.546	0.998	4.288
**B**	0.924	-0.79	0.75	0.854	1	87.155	0.726	0.904	1	98.297
**A**	0.942	-3.763	1.84	2.135	0.998	12.845	2.073	2.988	0.995	1.703

Both per-isomer fit and global scaling approaches point towards **isomer A** as the most likely major species, and **isomer B** as the most likely minor species. This is also in full agreement with previous literature approaches where the *cis* isomer was consistently the major isomer obtained.[2],[9],[28]

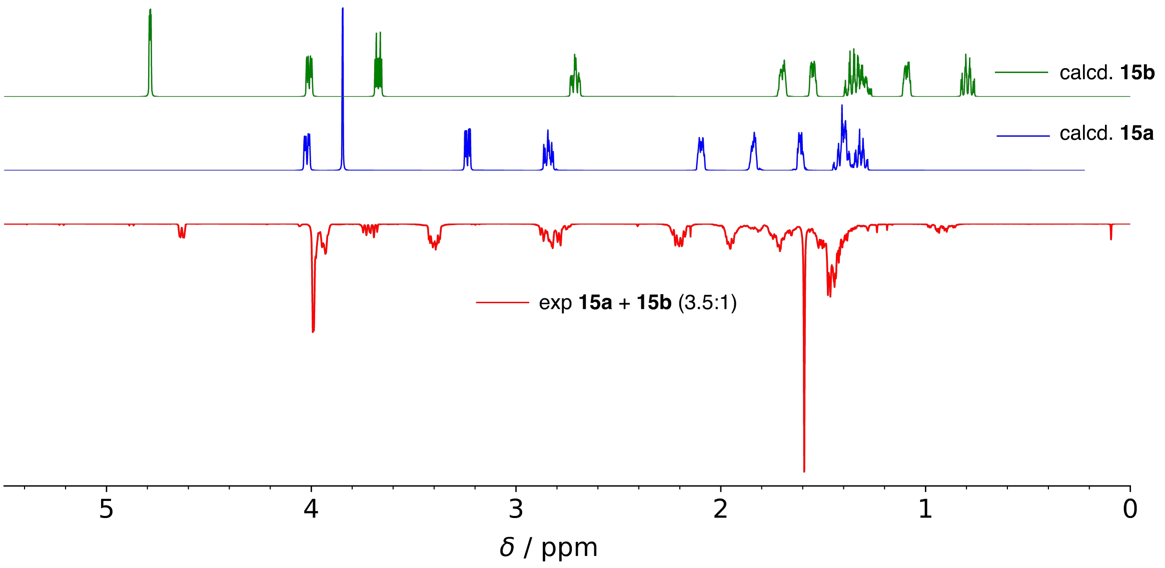

Figure S15: Experimental ^{1}H spectra of compound **15** and comparison with the calculated NMR spectra of the possible stereoisomers. Impurities in the experimental spectra are indicated with arrows.

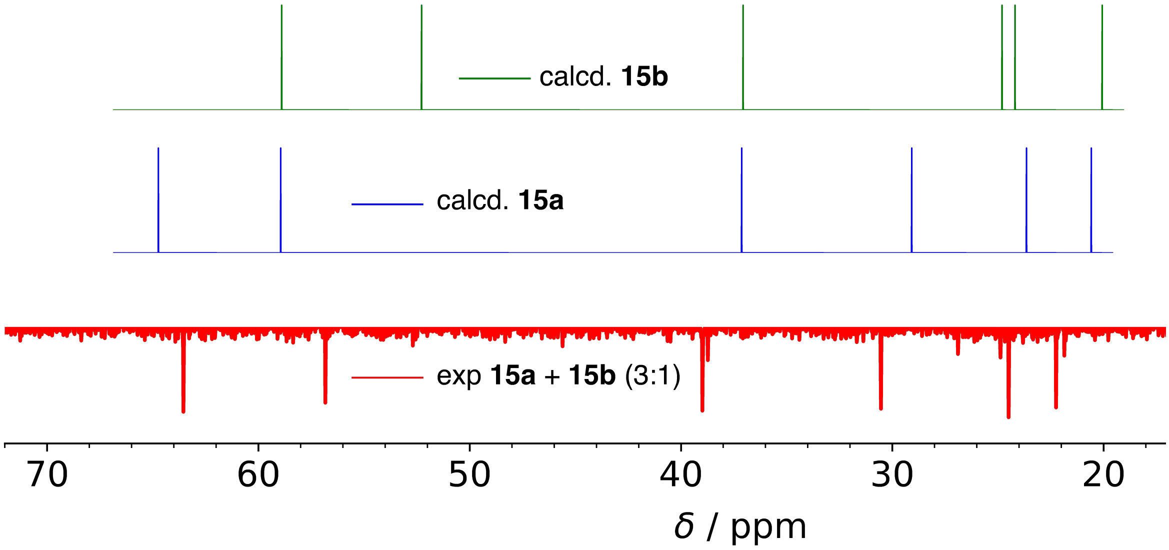

Figure S16: Experimental ^{13}C spectra of compound **15** and comparison with the calculated NMR spectra of the possible stereoisomers.

c. Analysis for Compound 16

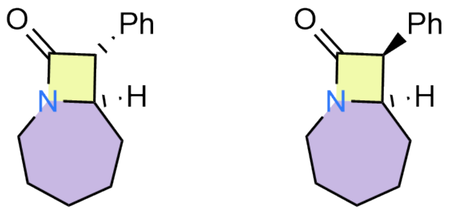

	16a	16b
d.r.	4	1
$\Delta G^a_{calcd.}$	0	+0.59

Figure S17: Structures of isomers A and B of compound **16** with key stereochemical differences indicated. The observed ratio and the relative energy differences between the isomers (calculated at CCSD(T)/aug-cc-cVQZ // r^2scan-3 level of theory) is indicated.

Table S10: Experimental versus calculated ^{13}C chemical shifts for compound **16** at the ωB97m-V/pcsseg-3 level of theory.

	Experimental	Calculated	
C_Nr.	**Species 1**	**Isomer A**	**Isomer B**
1	127.5	137.946	138.022
2	127.5	139.717	139.358
3	128.9	138.484	140.581
4		150.419	147.665
5	59.7	64.771	61.752
6	167.6	180.476	180.497
7	62.6	69.39	62.736
8	35.2	40.014	37.292
9	28.4	33.414	33.384
10	29.7	34.268	33.66
11	27.1	30.509	30.379
12	43.1	46.955	46.974

Table S11: Per-isomer and global regression statistics and DP4 probabilities for the candidate isomers of compound **16**.

**Iso**	**a_fit**	**b_fit**	**MAE_fit**	**RMSE_fit**	**R^2_fit**	**DP4%_fit**	**MAE_glb**	**RMSE_glb**	**R^2_glb**	**DP4%_glb**
**iso_B**	0.94	-2.02	0.69	0.809	1	86.77	1.127	1.274	0.999	84.044
**iso_A**	0.93	0.078	1.51	1.932	0.998	13.23	1.497	2.084	0.998	15.956

Both per-isomer fit and global scaling approaches point towards **isomer A** as the most likely major species.

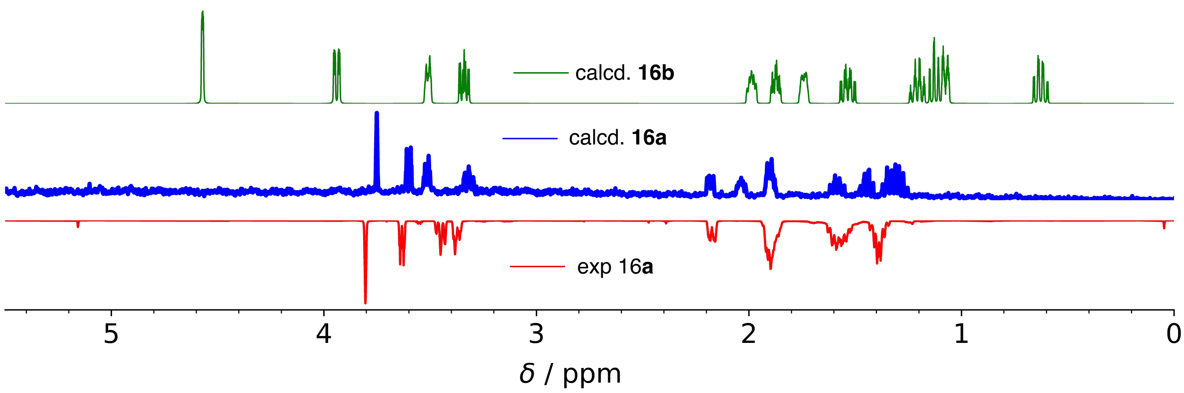

Figure S18: Experimental ^{1}H spectra of compound **16** and comparison with the calculated NMR spectra of the possible stereoisomers. Impurities in the experimental spectra are indicated with arrows.

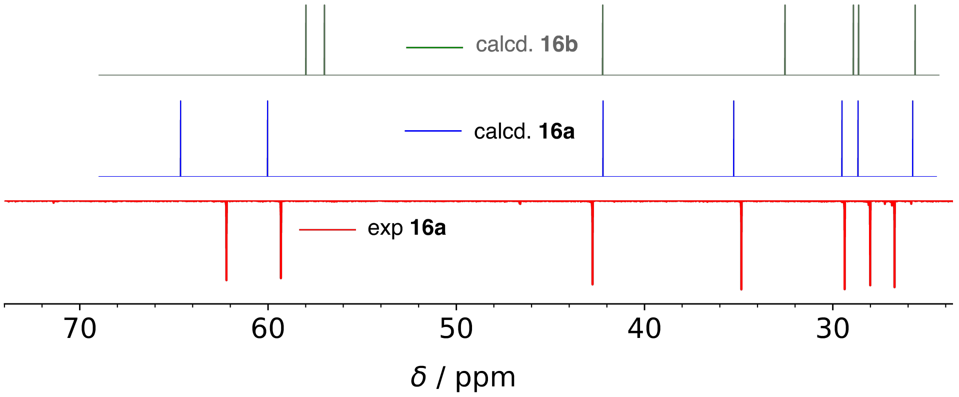

Figure S19: Experimental ^{13}C spectra of compound **16** and comparison with the calculated NMR spectra of the possible stereoisomers.

d. Analysis for Compound 9

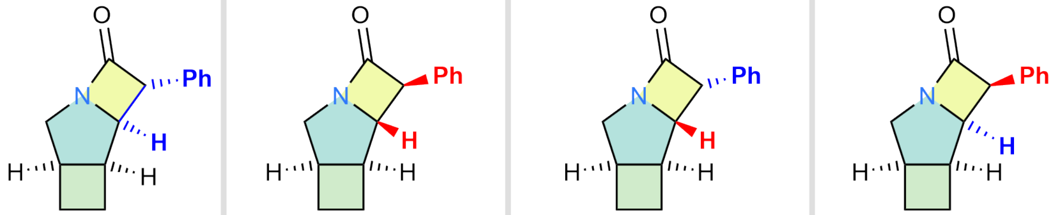

± 2-exo,3-exo-cis (2,3)	± 2-endo,3-endo-cis (2,3)	± 2-exo, 3-endo-trans (2,3)	± 2-endo, 3-exo-trans (2,3)
**9a**	**9b**	**9c**	**9d**

	9a	9b	9c	9d
d.r.	2.5	1	[not observed]	[not observed]
$\Delta G^{a}_{calcd.}$	0	+ 0.24	+ 0.39	+ 0.89

Figure S20: Structures of isomers A-D of compound **9** with key stereochemical differences indicated. The observed ratio and the relative energy differences between the isomers (calculated at CCSD(T)/aug-cc-cVQZ // r^2scan-3 level of theory) is indicated.

Table S12: Experimental versus calculated ^{13}C chemical shifts for compound **9** at the ωB97m-V/pcsseg-3 level of theory.

	**Experimental**		**Calculated**			
**C_Nr.**	**Species 1**	**Species 2**	**Isomer A**	**Isomer B**	**Isomer C**	**Isomer D**
**1**	127.5	127.5	138.169	138.424	147.087	139.564
**2**	129	129	139.941	139.98	139.792	136.946
**3**	127.7	127.6	138.759	138.92	139.649	136.772
**4**	136.1	135.9	150.45	149.869	138.341	139.564
**5**	55.6	62.4	61.549	67.973	63.14	55.439
**6**	174.8	176.3	191.322	190.142	195.241	192.29
**7**	63.9	68.8	70.579	76.163	70.558	64.719
**8**	39.8	43.4	42.639	47.835	42.142	45.457
**9**	18.9	23.9	21.399	23.803	27.419	23.072
**10**	24.4	25.3	27.031	29.623	24.071	27.453
**11**	45.4	45.8	49.571	51.469	48.806	49.773
**12**	50.5	53.9	52.702	56.543	57.808	54.859

Table S13: Per-isomer and global regression statistics and DP4 probabilities for the candidate isomers of compound **9**.

Iso	a_fit	b_fit	MAE_fit	RMSE_fit	R^2_fit	DP4%_fit	MAE_glb	RMSE_glb	R^2_glb	DP4%_glb
**iso_A**	**0.915**	**0.158**	**0.86**	**1.01**	**1**	**82.798**	**0.825**	**1.051**	**1**	**99.546**
**iso_B**	0.946	-4.758	1.54	1.847	0.999	16.812	2.284	3.1	0.996	0.344
**iso_D**	0.932	0.033	2.51	3.011	0.996	0.388	2.514	3.366	0.996	0.109
**iso_C**	0.915	-0.619	3.1	4.174	0.993	0.001	3.197	4.257	0.993	0.001
**iso_B**	**0.922**	**-0.339**	**1.07**	**1.329**	**0.999**	**80.194**	**1.101**	**1.337**	**0.999**	**99.799**
**iso_A**	0.891	4.525	1.59	1.966	0.998	19.77	2.524	3.333	0.995	0.2
**iso_C**	0.892	3.682	2.74	3.789	0.994	0.018	3.425	4.322	0.992	0.001
**iso_D**	0.907	4.452	3.11	3.792	0.994	0.018	3.704	5.243	0.989	0

Both per-isomer fit and global scaling approaches point towards **isomer A** as the most likely major species and **isomer B** as the most likely minor species.

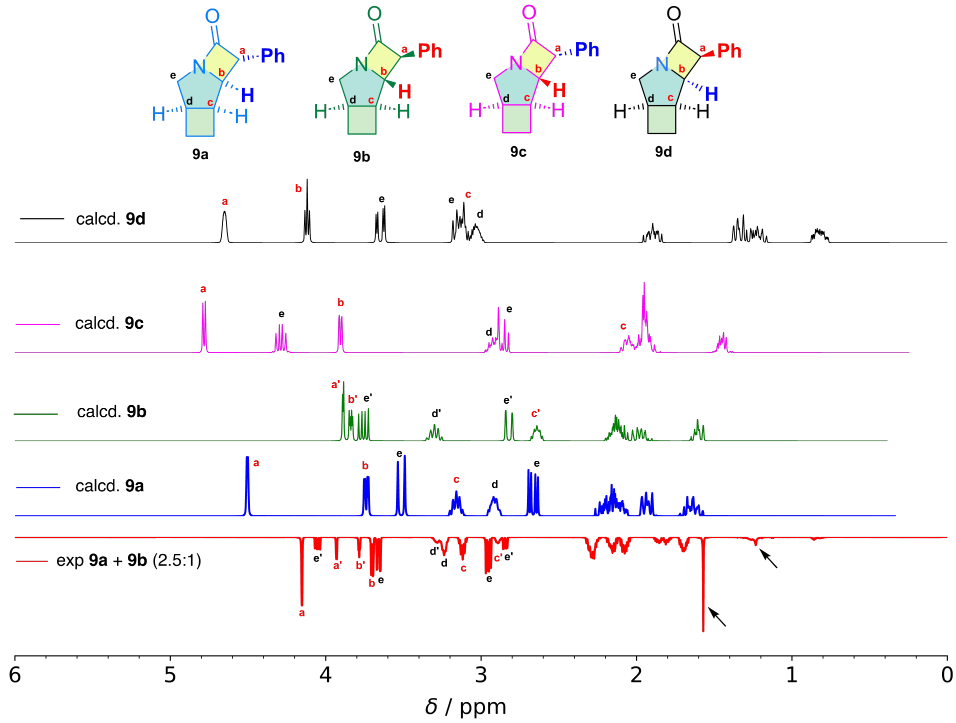

Figure S21: Experimental ^{1}H spectra of compound **9** and comparison with the calculated NMR spectra of the possible stereoisomers. Impurities in the experimental spectra are indicated with arrows.

e. Analysis for Compound 11

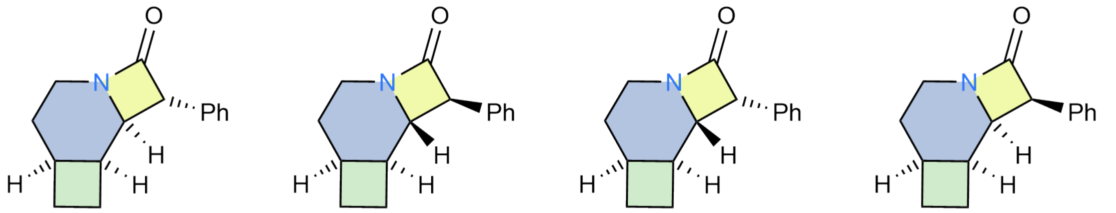

	**11a**	**11b**	**11c**	**11d**
d.r.	1.6	1	[not observed]	[not observed]
$\Delta G^{a}_{calcd.}$	0	+ 0.99	+ 1.34	+ 0,95

Figure S22: Structures of isomers A-D of compound **11** with key stereochemical differences indicated. The observed ratio and the relative energy differences between the isomers (calculated at CCSD(T)/aug-cc-cVQZ // r^{2}scan-3 level of theory) is indicated.

Table S14: Experimental versus calculated ^{13}C chemical shifts for compound **11** at the ωB97m-V/pcsseg-3 level of theory.

	Experimental		Calculated			
**C_Nr.**	**Species 1**	**Species 2**	**Isomer A**	**Isomer B**	**Isomer C**	**Isomer D**
**1**	127.4	127.5	138.224	138.013	138.169	136.902
**2**	128.9	128.9	139.864	139.827	139.873	139.253
**3**	127.6	127.5	138.9	138.365	138.562	138.165
**4**	135.6	135.8	149.745	150.615	150.427	148.353
**5**	57.8	63.0	63.568	68.838	70.559	58.392
**6**	168.9	167.7	183.798	182.631	181.348	186.143
**7**	55.3	53.6	62.453	66.126	66.748	55.647
**8**	33.5	31.8	36.83	40.419	39.589	37.385
**9**	18.7	32.5	20.561	28.634	28.357	21.834
**10**	24.4	24.7	23.937	26.261	25.151	23.525
**11**	30.3	29.3	33.53	36.106	36.598	32.949
**12**	22.1	26.4	28.214	27.138	27.303	27.644
**13**	36.1	39.1	39.621	41.387	39.239	39.251

Table S15: Per-isomer and global regression statistics and DP4 probalities for the candidate isomers of compound **11**.

Isomer	a_fit	b_fit	MAE_fit	RMSE_fit	R2_fit	DP4%_fit	MAE_glb	RMSE_glb	R2_glb	DP4%_glb
**iso_A**	**0.921**	**-0.688**	**0.986**	**1.422**	**0.999**	**76.404**	**0.931**	**1.459**	**0.999**	**90.22**
**iso_B**	0.947	-4.643	1.807	2.167	0.998	11.073	2.645	3.366	0.996	0.117
**iso_D**	0.915	0.758	1.814	2.174	0.998	10.837	1.796	2.287	0.998	9.629
**iso_C**	0.946	-4.38	2.171	2.702	0.997	1.686	2.798	3.616	0.995	0.033
**iso_B**	**0.917**	**-0.526**	**2.523**	**3.265**	**0.996**	**72.762**	**2.337**	**3.302**	**0.996**	**77.086**
**iso_C**	0.916	-0.28	2.859	3.53	0.995	19.769	2.761	3.548	0.995	22.767
**iso_D**	0.886	4.758	2.706	3.799	0.994	4.757	3.315	4.844	0.991	0.009
**iso_A**	0.891	3.405	2.612	3.9	0.994	2.711	2.758	4.434	0.992	0.138

Both per-isomer fit and global scaling approaches point towards **isomer A** as the most likely major species and **isomer B** as the most likely minor species.

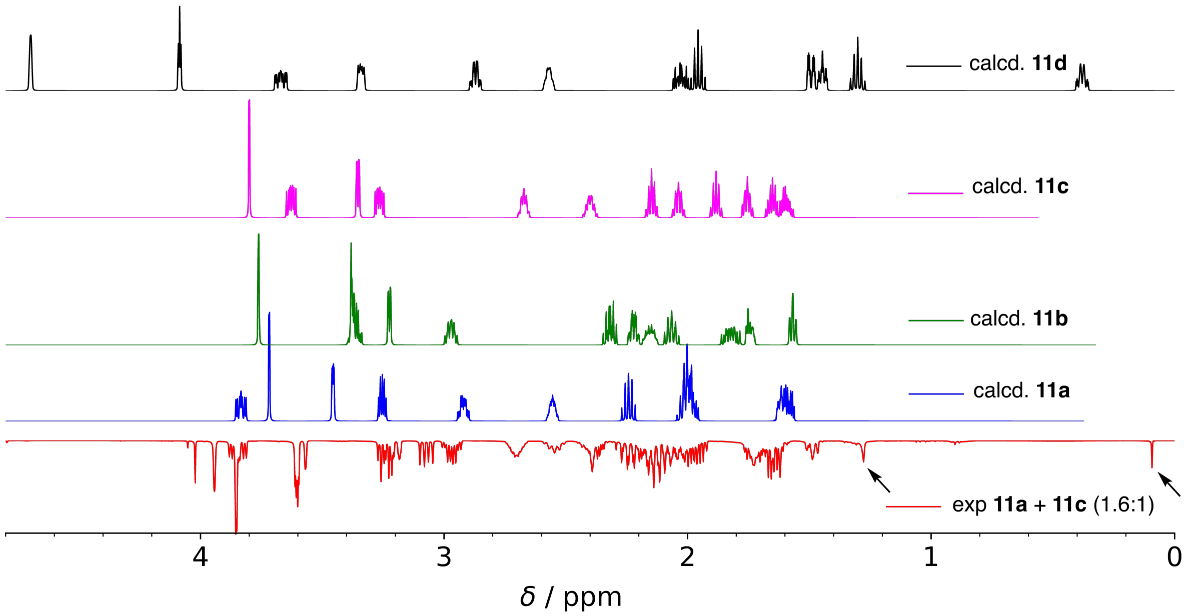

Figure S23: Experimental ^{1}H spectra of compound **11** and comparison with the calculated NMR spectra of the possible stereoisomers. Impurities in the experimental spectra are indicated with arrows.

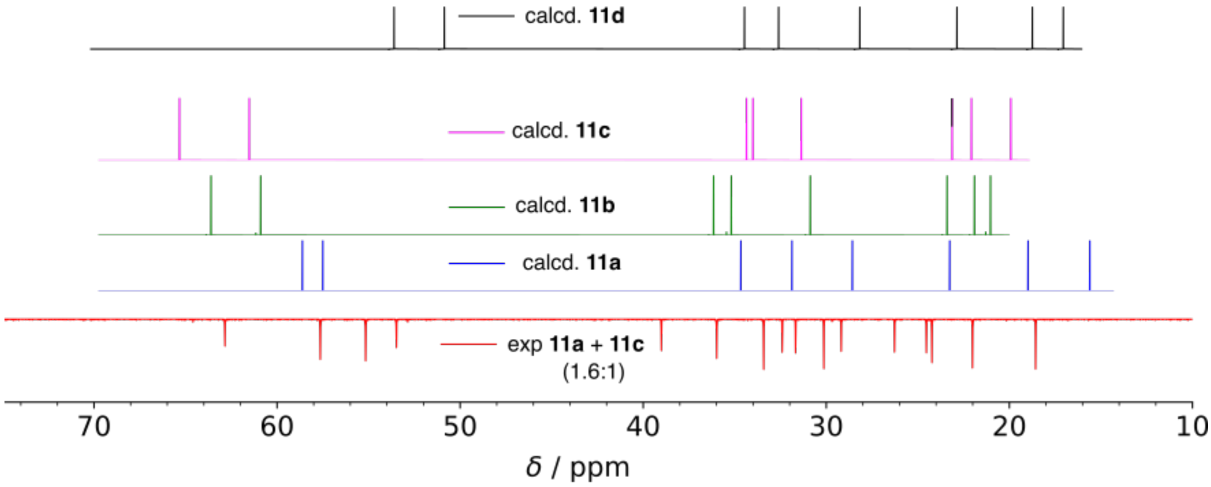

Figure S24: Experimental ^{13}C spectra of compound **11** and comparison with the calculated NMR spectra of the possible stereoisomers.

f. Analysis for Compound 12

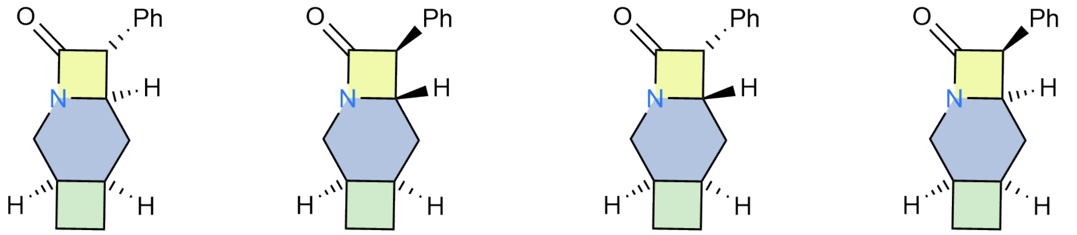

	12a	12b	12c	12d
d.r.	1.8	1	[not observed]	[not observed]
$\Delta G^{a}_{calcd.}$	0	+ 0.31	+ 0.76	+ 0.40

Figure S25: Structures of isomers A-D of compound **12** with key stereochemical differences indicated. The observed ratio and the relative energy differences between the isomers (calculated at CCSD(T)/aug-cc-cVQZ // r^2scan-3 level of theory) is indicated.

Table S16: Experimental versus calculated ^{13}C chemical shifts for compound **12** at the ωB97m-V/pcsseg-3 level of theory.

	Experimental		Calculated			
**C_Nr.**	**Species 1**	**Species 2**	**Isomer A**	**Isomer B**	**Isomer C**	**Isomer D**
**1**	127.5	127.6	138.243	138.243	137.871	138.069
**2**	128.9	128.9	139.91	139.905	139.361	139.402
**3**	127.6	127.5	138.756	138.792	140.266	140.628
**4**	136	135.8	150.173	150.111	146.807	146.905
**5**	63.6	62.9	69.211	68.444	62.288	63.493
**6**	168.3	169.2	181.225	183.993	186.617	180.806
**7**	58.9	53.4	60.355	60.335	53.964	53.778
**8**	25.5	32.2	35.816	35.548	33.503	31.739
**9**	36.9	31	34.447	34.165	32.853	33.927
**10**	25.2	23	28.309	24.411	24.426	28.077
**11**	24.4	25.9	25.883	27.251	26.992	25.928
**12**	32.2	28.4	33.366	32.138	32.358	34.304
**13**	36.8	41.2	41.704	44.297	44.212	41.196

Table S17: Per-isomer and global regression statistics and DP4 probabilities for the candidate isomers of compound **12**.

Isomer	a_fit	b_fit	MAE_fit	RMSE_fit	R^2_fit	DP4%_fit	MAE_glb	RMSE_glb	R^2_glb	DP4%_glb
**iso_A**	**0.922**	**-0.082**	**1.9**	**2.868**	**0.997**	**69.05**	**2.004**	**2.888**	**0.997**	**76.841**
**iso_B**	0.912	0.715	2.28	3.125	0.996	22.691	2.388	3.174	0.996	21.964
**iso_D**	0.915	1.835	2.57	3.349	0.996	7.984	2.619	3.76	0.995	1.168
**iso_C**	0.899	2.872	3.05	3.984	0.994	0.275	3.46	4.4	0.992	0.027
**iso_B**	**0.918**	**-0.172**	**0.77**	**0.933**	**1**	**73.286**	**0.727**	**0.941**	**1**	**82.144**
**iso_A**	0.927	-0.916	1.36	1.79	0.999	13.599	1.507	1.833	0.999	13.776
**iso_C**	0.906	1.949	1.44	1.864	0.999	11.175	1.784	2.297	0.998	3.45
**iso_D**	0.92	1.008	2.07	2.429	0.998	1.94	2.395	2.762	0.997	0.63

Both per-isomer fit and global scaling approaches point towards **isomer A** as the most likely major species and **isomer B** as the most likely minor species.

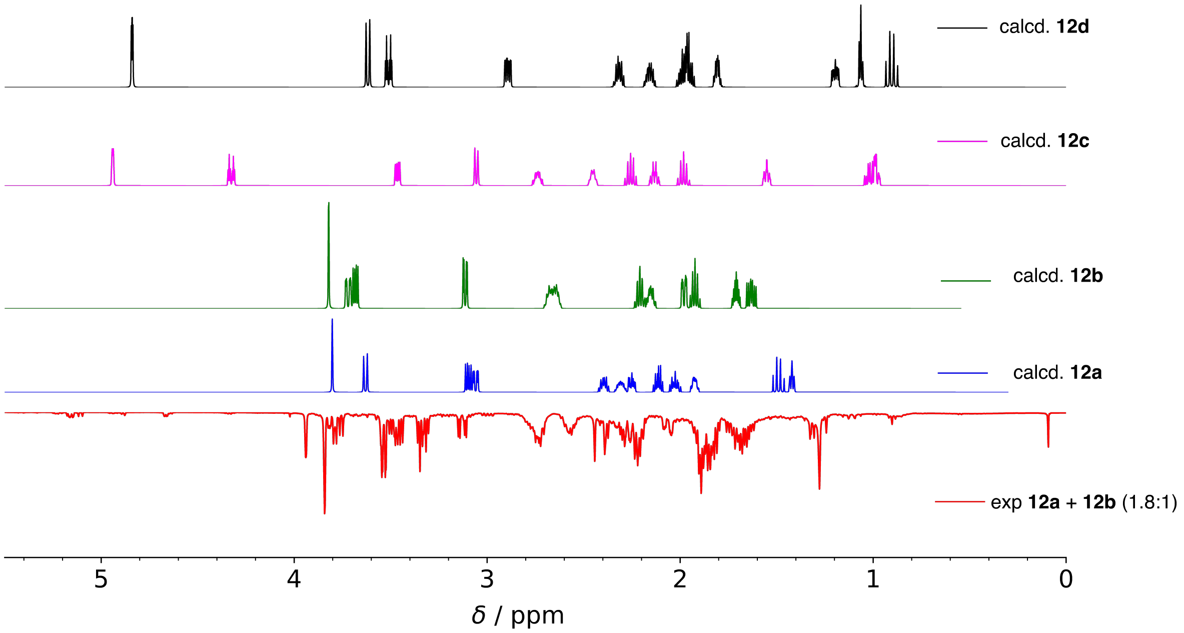

Figure S26: Experimental ^{1}H spectra of compound **12** and comparison with the calculated NMR spectra of the possible stereoisomers. Impurities in the experimental spectra are indicated with arrows.

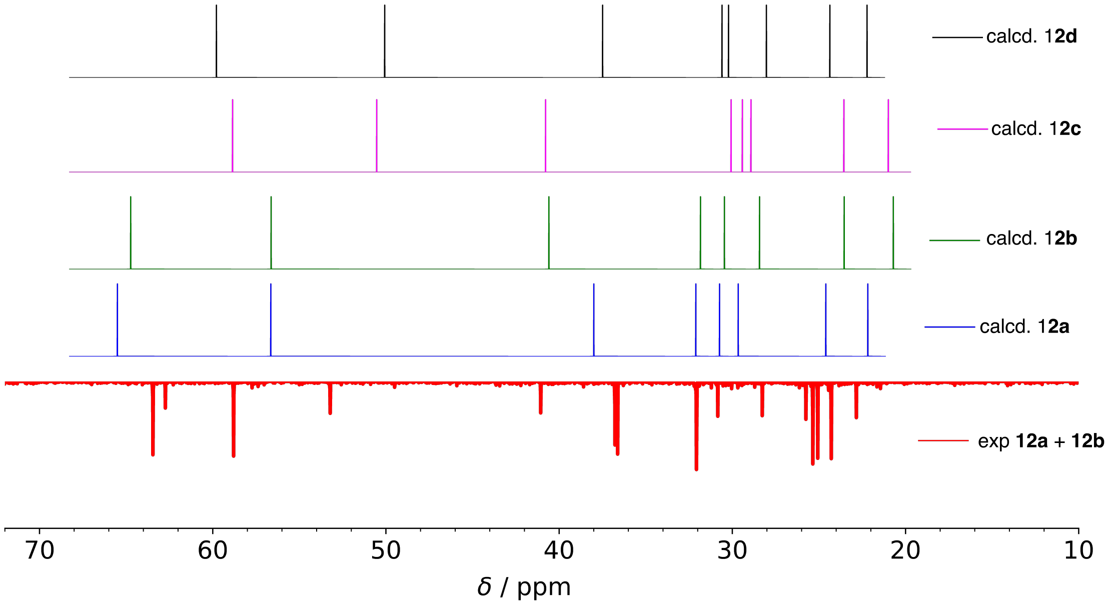

Figure S27: Experimental ^{13}C spectra of compound **12** and comparison with the calculated NMR spectra of the possible stereoisomers.

g. Analysis for Compound 13

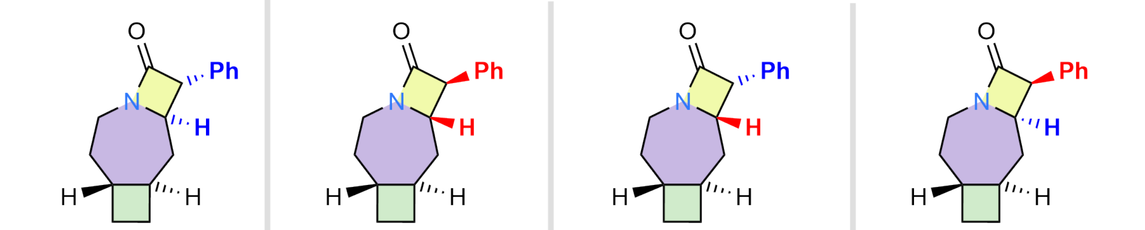

	± 2-exo,3-exo-cis (2,3) **13a**	±2-endo,3-endo-cis (2,3) **13b**	± 2-exo, 3-endo-trans (2,3) **13c**	± 2-endo, 3-exo-trans (2,3) **13d**
d.r.	0.3	1	3.5	3.5
$\Delta G^{a}_{calcd.}$	+1.68	0	+ 0.7	+ 1.95

Figure S28: Structures of isomers A-D of compound **13** with key stereochemical differences indicated. The observed ratio and the relative energy differences between the isomers (calculated at CCSD(T)/aug-cc-cVQZ // r^2scan-3 level of theory) is indicated.

Table S18: Experimental versus calculated ^{13}C chemical shifts for compound **13** at the ωB97m-V/pcsseg-3 level of theory.

	Experimental		Calculated			
**C_Nr.**	**Species 1**	**Species 2**	**Isomer A**	**Isomer B**	**Isomer C**	**Isomer D**
**1**	128.6	127.4	141.79	141.869	139.297	143.132
**2**	128.9	129	143.397	143.484	136.472	141.867
**3**	127.4	128.8	142.552	142.565	138.333	143.132
**4**	134	133.8	154.051	153.513	146.369	144.447
**5**	57.3	58.8	72.559	70.175	58.867	67.757
**6**	168.7	168.3	185.514	185.816	182.118	184.736
**7**	60.1	58.2	75.117	73.846	58.426	67.75
**8**	36.5	32.6	49.079	44.096	33.792	43.393
**9**	42	45	45.62	46.303	40.497	45.12
**10**	25.3	25.3	30.335	31.133	23.666	30.322
**11**	26.1	26.2	33.45	31.273	23.74	33.224
**12**	43.6	46	50.37	54.802	46.221	49.971
**13**	31.2	34.6	39.065	41.755	35.239	39.013
**14**	41.2	42.9	46.598	49.709	43.067	46.828

Table S19: Per-isomer and global regression statistics and DP4 probabilities for the candidate isomers of compound **13**

**Isomer**	**a_fit**	**b_fit**	**MAE_fit**	**RMSE_fit**	**R^2_fit**	**DP4%_fit**	**MAE_glb**	**RMSE_glb**	**R^2_glb**	**DP4%_glb**
**iso_D**	**0.93**	**-3.391**	**1.35**	**1.695**	**0.999**	**62.747**	**2.55**	**2.859**	**0.997**	**95.825**
**iso_C**	0.9	3.921	1.64	2.005	0.998	25.695	2.849	3.494	0.995	4.174
**iso_B**	0.926	-5.033	1.97	2.262	0.998	10.941	4.188	4.772	0.99	0.001
**iso_A**	0.923	-4.718	2.49	2.969	0.996	0.617	4.178	5.092	0.989	0
**iso_C**	**0.894**	**4.864**	**1.36**	**1.713**	**0.999**	**93.406**	**3.239**	**3.8**	**0.994**	**3.041**
**iso_D**	0.923	-2.313	2.04	2.586	0.997	5.038	2.58	3.16	0.996	96.952
**iso_B**	0.919	-3.954	2.16	2.863	0.996	1.556	4.143	4.726	0.99	0.007
**iso_A**	0.915	-3.542	3.46	4.198	0.992	0.001	4.223	5.605	0.986	0

Both per-isomer fit and global scaling approaches point towards **isomer D** and **isomer C** as the most likely major species formed.

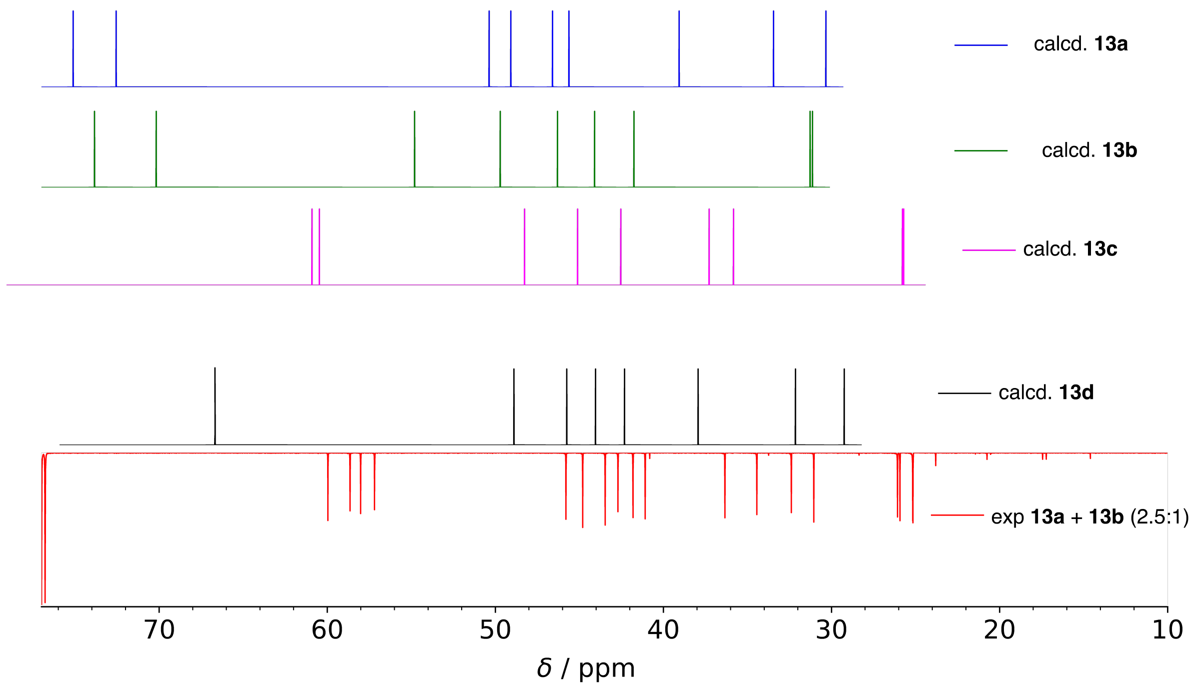

Figure S29: Experimental ^{13}C spectra of compound **13** and comparison with the calculated NMR spectra of the possible stereoisomers.

During the thermal cyclisation reaction, small amounts of **isomers A** and **B** could be observed in the crude NMR but could not be separated in large enough amounts in order to record ^{13}C NMR spectroscopy. We tentatively assume isomer B is formed in larger amounts compared to isomer A based on calculated ^{1}H NMR spectra using the same methodology as described above.

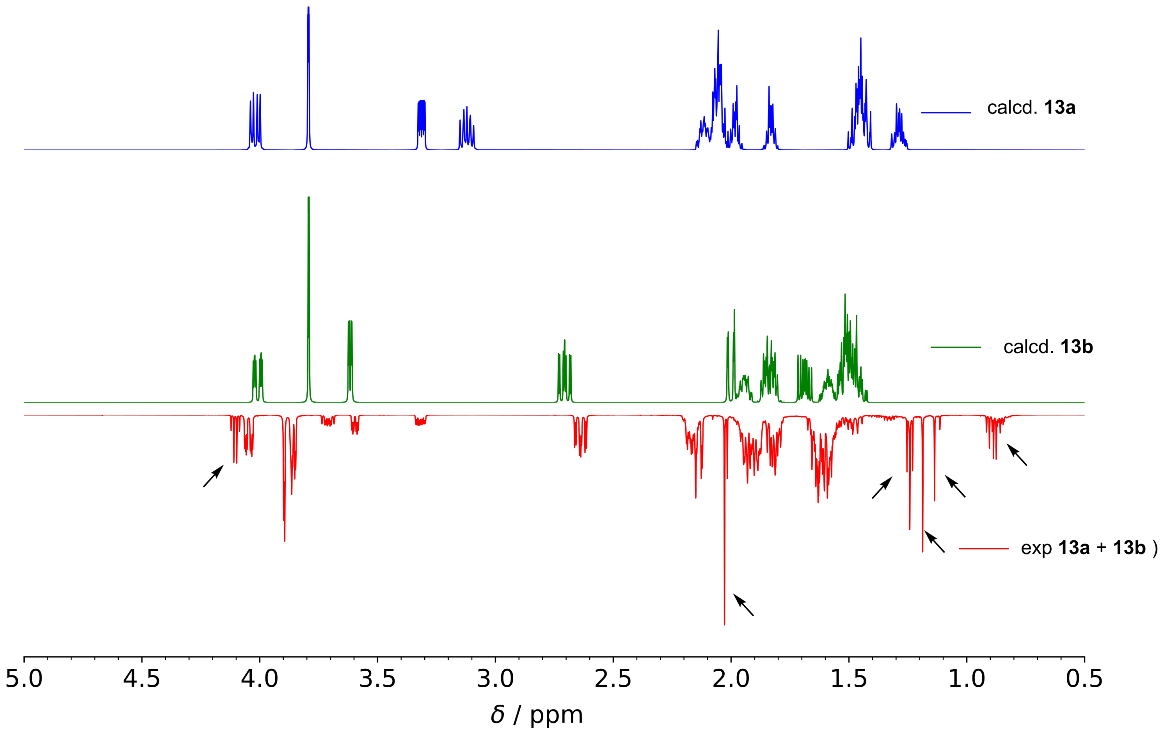

Figure S30: Experimental ^{1}H spectra of compound **13** and comparison with the calculated NMR spectra of the possible stereoisomers. Impurities in the experimental spectra are indicated with arrows.

3.5 Comparison of energies of all stereoisomers considered

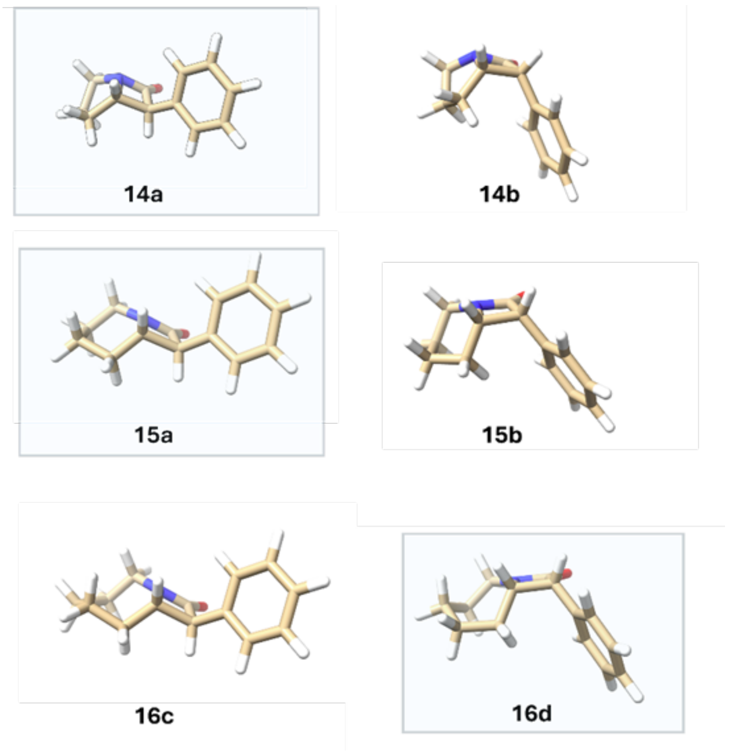

Figure S31: Calculated 3D structures of the bicyclic β-lactams investigated in this study. The main stereoisomer observed is highlighted.

Table S20: Computed Gibbs free energies of all possible isomers (ωB97X-D4/def2-TZVP//r^{2}scan-3 and CCSD(T)/aug-cc-cVQZ // r^{2}scan-3 level of theory) with relative ΔG referenced to the conformer lowest in energy of each compound.

Compound	E (Gibbs) ωB97x-D4 [Ha]	ΔG (ωB97x-D4) (Kcal/mol)	E (Gibbs) (CCSD(T) correction) [Ha]	ΔG (CCSD(T corr.) (Kcal/mol)
**9a**	-672.2987685	0	-671.0445878	0
**9b**	-672.2988986	-0.08	-671.044202	+0.24
**9c**	-672.2981831	+0.36	-671.0439724	+0.39
**9d**	-672.2974271	+0.84	-671.0431766	+0.88
**11a**	-711.3552504	0	-710.2668364	0
**11b**	-711.3542081	+0.65	-710.2652625	+0.99
**11c**	-711.3530948	+1.35	-710.2646882	+1.34
**11d**	-711.3528397	+1.51	-710.2653107	+0.95
**12a**	-711.3554851	0	-710.2669927	0
**12b**	-711.3542313	+0.31	-710.266493	+0.79
**12c**	-711.3542691	+1.12	-710.265199	+0.76
**12d**	-711.3548498	+0.33	-710.2664659	+0.40
**13a**	-750.886486	+2.06	-749.480765	+1.69
**13b**	-750.8891747	0	-749.4840431	0
**13c**	-750.8880304	+0.70	-749.4829243	+0.72
**13d**	-750.8860606	+2.17	-749.4805755	+1.95
**14a**	-594.936249	0	-593.8328184	0
**14b**	-594.935928	+0.54	-593.8319012	+0.58
**15a**	-634.0247407	0	-633.0575317	0
**15b**	-634.0237325	+0.91	-633.0563715	+0.63
**16a**	-673.2864089	0	-672.2700049	0
**16b**	-673.285467	+0.59	-672.2692784	+0.46

3.6 Script for performing the DP4 analysis

```
#!/usr/bin/env python
# isomer_decision.py
# ---------------------------------------------------------------
# Quick script for assigning structures based on experimental
   data and benchmarked computational data
# Ensure that you do a conformer sampling before and calculate
   the NMR data at a high level of theory (pcsseg-2 or pcsseg
   -3) before doing a Boltzmann avereging on the chemicals
   shifts at the desired temperature. I.e. via CREST, CENSO and
    ANMR from Grimme et al.
# ---------------------------------------------------------------
import os
import sys
import glob
import pandas as pd
```

```
import numpy as np
import re
import textwrap

# 20.07.2025
# Author: Dragos - Adrian Rosca, dragos.rosca@uni.heidelberg.de
   ; dragos-adrian.rosca@univ-rennes.fr

def main():
    # 1. Find data files in current folder
    candidates = sorted(glob.glob("*.xlsx") + glob.glob("*.xls"
       ) + glob.glob("*.csv"))
    if not candidates:
        sys.exit("Error: No Excel/CSV files found here.")

    print("\nFiles available for analysis:")
    for idx, filename in enumerate(candidates, 1):
        print(f"  [{idx}] {filename}")
    choice = input(f"Pick a file by number (1-{len(candidates)
       }): ").strip()

    try:
        chosen_file = candidates[int(choice) - 1]
    except Exception:
        sys.exit("Invalid selection -- please restart and pick
           a valid number.")

    base_name, ext = os.path.splitext(chosen_file)

    # 2. Load the data
    try:
        if ext.lower() == '.csv':
            data = pd.read_csv(chosen_file)
        else:
            data = pd.read_excel(chosen_file)
    except Exception as err:
        sys.exit(f"Could not read '{chosen_file}': {err}")

    # 3. Identify experimental shift column
    exp_col = next(
```

```
    (col for col in data.columns if 'shift' in col.lower()
       and 'exp' in col.lower()),
    None
)
if not exp_col:
    sys.exit("Experimental shift column not found. Make
       sure a column name contains 'shift' and 'exp'.")
print(f"\nUsing experimental column: '{exp_col}'")

# 4. Locate calculated isomer columns
iso_columns = [
    c for c in data.columns
    if re.match(r"shift_calc_iso_[A-Z]", c, re.IGNORECASE)
]
if not iso_columns:
    sys.exit("No calculated isomer columns found (e.g.
       shift_calc_iso_A).")
print(f"Found {len(iso_columns)} isomer candidates\n")
# 5. Filter aliphatic carbons if needed
ans = input("Filter to aliphatic carbons (<80 ppm)? [y/N]:
   ").strip().lower()
if ans == 'y':
    before_rows = data.shape[0]
    data = data[data[exp_col] < 80]
    kept = data.shape[0]
    print(f"Kept {kept}/{before_rows} rows under 80 ppm.")
    if kept == 0:
        sys.exit("No data left after filtering.")

# 6. Set up global regression (defaults provided)
default_slope, default_intercept = 0.92, -0.28
reg_input = input(
    f"Enter slope and intercept [a b] (default {
       default_slope}, {default_intercept}): "
).strip()
if reg_input:
    parts = re.split(r"[ ,;]+", reg_input)
    if len(parts) != 2:
        sys.exit("Please provide exactly two numbers for
           slope and intercept.")
```

```
    try:
        default_slope = float(parts[0])
        default_intercept = float(parts[1])
    except ValueError:
        sys.exit("Regression inputs must be numeric.")
a_global, b_global = default_slope, default_intercept
print(f"Using global regression: shift = {a_global:.3f}*
   calc + {b_global:.3f}\n")
# 7. Choose Gaussian σ for DP4
sigma_default = 3.0
sigma_input = input(f"Gaussian sigma for DP4 (ppm, default
   {sigma_default}): ").strip()
if sigma_input:
    try:
        sigma = float(sigma_input)
    except ValueError:
        sys.exit("Sigma must be a number.")
else:
    sigma = sigma_default
print(f"Sigma set to {sigma} ppm\n")

# 8. Calculate stats for each isomer
results = []
for col in iso_columns:
    mask = data[col].notna() & data[exp_col].notna()
    count = mask.sum()
    if count < 3:
        print(f"Skipping {col}: only {count} matching
           points.")
        continue

    x = data.loc[mask, col]
    y = data.loc[mask, exp_col]

    # per-isomer fit
    a_fit, b_fit = np.polyfit(x, y, 1)
    resid_fit = (a_fit * x + b_fit) - y
    mae_fit   = resid_fit.abs().mean()
    rmse_fit  = np.sqrt((resid_fit**2).mean())
```

```
    r2_fit    = 1 - (resid_fit**2).sum() / ((y - y.mean())
       **2).sum()
    dp4_fit   = np.exp(-0.5 * (resid_fit.abs() / sigma)**2)
       .prod()

    # global scaling
    resid_glb = (a_global * x + b_global) - y
    mae_glb   = resid_glb.abs().mean()
    rmse_glb  = np.sqrt((resid_glb**2).mean())
    r2_glb    = 1 - (resid_glb**2).sum() / ((y - y.mean())
       **2).sum()
    dp4_glb   = np.exp(-0.5 * (resid_glb.abs() / sigma)**2)
       .prod()

    results.append({
        'Isomer':   col.replace('shift_calc_', ''),
        'N':        count,
        'a_fit':    a_fit,  'b_fit':    b_fit,
        'MAE_fit':  mae_fit,'RMSE_fit': rmse_fit,'R2_fit':
           r2_fit,'DP4_fit': dp4_fit,
        'MAE_glb':  mae_glb,'RMSE_glb': rmse_glb,'R2_glb':
           r2_glb,'DP4_glb': dp4_glb
    })

df_res = pd.DataFrame(results)
df_res['DP4%_fit'] = 100 * df_res['DP4_fit'] / df_res['
   DP4_fit'].sum()
df_res['DP4%_glb'] = 100 * df_res['DP4_glb'] / df_res['
   DP4_glb'].sum()
df_res = df_res.sort_values('DP4%_fit', ascending=False).
   reset_index(drop=True)

# 9. Print detailed statistics in custom layout
print("=== Detailed statistics ===")
cols = [
    ("Isomer",   6), ("N",        5), ("a_fit",    6), ("
       b_fit",    6),
    ("MAE_fit",  7), ("RMSE_fit", 8), ("R2_fit",   6), ("
       DP4%_fit", 9),
```

```
    ("MAE_glb",  7), ("RMSE_glb", 8), ("R2_glb",   6), ("
       DP4%_glb", 9),
]
header = "".join(name.rjust(w) + " " for name, w in cols).
   rstrip()
print(header)
for _, row in df_res.iterrows():
    line = ""
    for name, w in cols:
        if name == "Isomer":
            s = f"{row[name]}".rjust(w)
        else:
            s = f"{row[name]:.3f}".rjust(w)
        line += s + " "
    print(line.rstrip())

# 10. Print Summary
print("\nSummary")
print("-------")
winner = df_res.iloc[0]
runner = df_res.iloc[1] if len(df_res) > 1 else None
gap    = winner['DP4%_fit'] - (runner['DP4%_fit'] if runner
    is not None else 0)
agree  = winner['DP4%_glb'] == df_res['DP4%_glb'].max()

print(f"1) Most probable isomer : {winner['Isomer']}")
print(f"   • DP4 (per-isomer fit)  : {winner['DP4%_fit']:.2
   f} %")
print(f"   • DP4 (global scaling)  : {winner['DP4%_glb']:.2
   f} %\n")

if runner is not None:
    print(f"2) Runner-up isomer     : {runner['Isomer']}")
    print(f"   • DP4 (per-isomer fit)  : {runner['DP4%_fit
       ']:.2f} %")
    print(f"   • DP4 (global scaling)  : {runner['DP4%_glb
       ']:.2f} %\n")

print(f"Confidence gap between top and runner-up: {gap:.2f}
    %.")
```

```
if agree:
    print("Global scaling agrees on the winner.")
else:
    print("Global scaling would choose a different winner;
       see DP4%_glb column.")

# 11. Optional export using UTF-8
if input("\nSave report to TXT file? [y/N]: ").strip().
   lower() == 'y':
    outname = f"{base_name}_report.txt"
    with open(outname, 'w', encoding='utf-8') as fout:
        fout.write("=== Detailed statistics ===\n")
        fout.write(header + "\n")
        for _, row in df_res.iterrows():
            line = ""
            for name, w in cols:
                if name == "Isomer":
                    s = f"{row[name]}".rjust(w)
                else:
                    s = f"{row[name]:.3f}".rjust(w)
                line += s + " "
            fout.write(line.rstrip() + "\n")
        fout.write("\nSummary\n-------\n")
        fout.write(f"1) Most probable isomer : {winner['
           Isomer']}\n")
        fout.write(f"   • DP4 (per-isomer fit)  : {winner['
           DP4%_fit']:.2f} %\n")
        fout.write(f"   • DP4 (global scaling)  : {winner['
           DP4%_glb']:.2f} %\n\n")
        if runner is not None:
            fout.write(f"2) Runner-up isomer     : {runner
               ['Isomer']}\n")
            fout.write(f"   • DP4 (per-isomer fit)  : {
               runner['DP4%_fit']:.2f} %\n")
            fout.write(f"   • DP4 (global scaling)  : {
               runner['DP4%_glb']:.2f} %\n\n")
        fout.write(f"Confidence gap between top and runner-
           up: {gap:.2f} %.\n")
        if agree:
```

```
                fout.write("Global scaling agrees on the winner
                    .\n")
            else:
                fout.write("Global scaling would choose a
                    different winner; see DP4%_glb column.\n")
        print(f"Report saved to {outname}")

if __name__ == '__main__':
    main()
```

4 Spectra

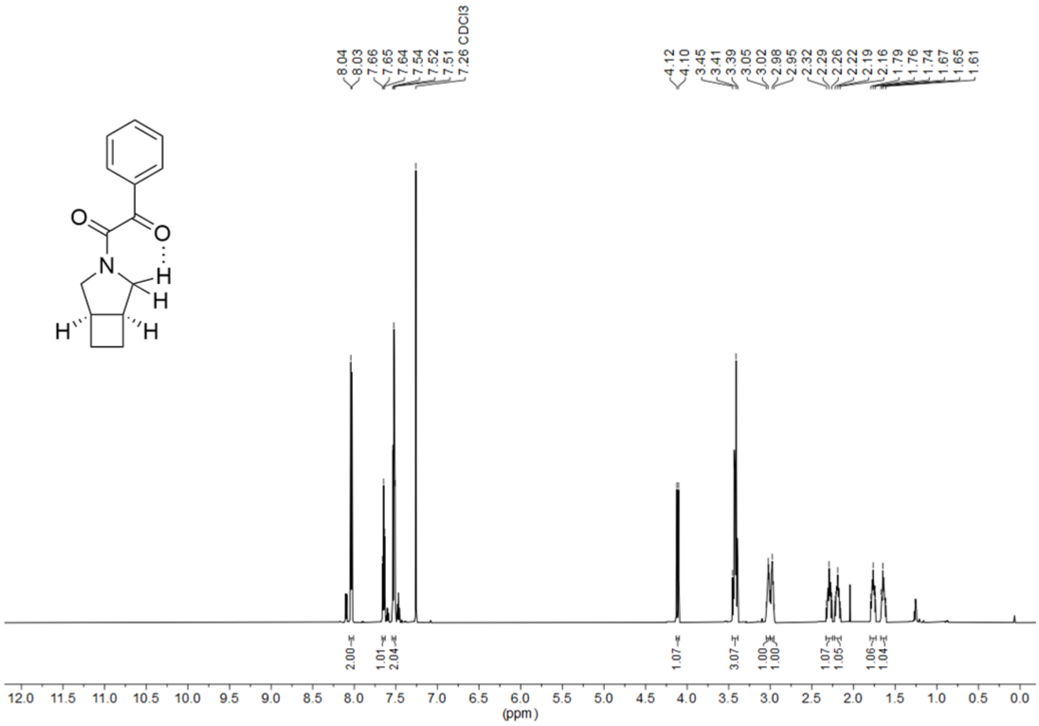

Figure S32: ^{1}H-NMR of **6** (600 MHz, $CDCl_3$).

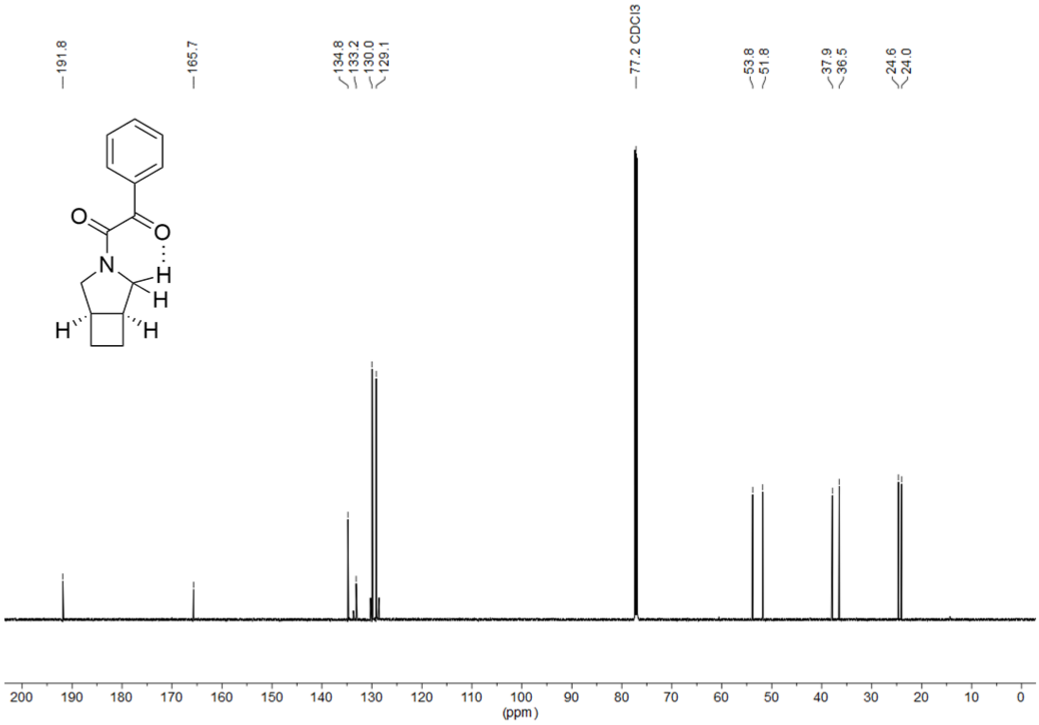

Figure S33: ^{13}C{^{1}H}-NMR of **6** (151 MHz, $CDCl_3$).

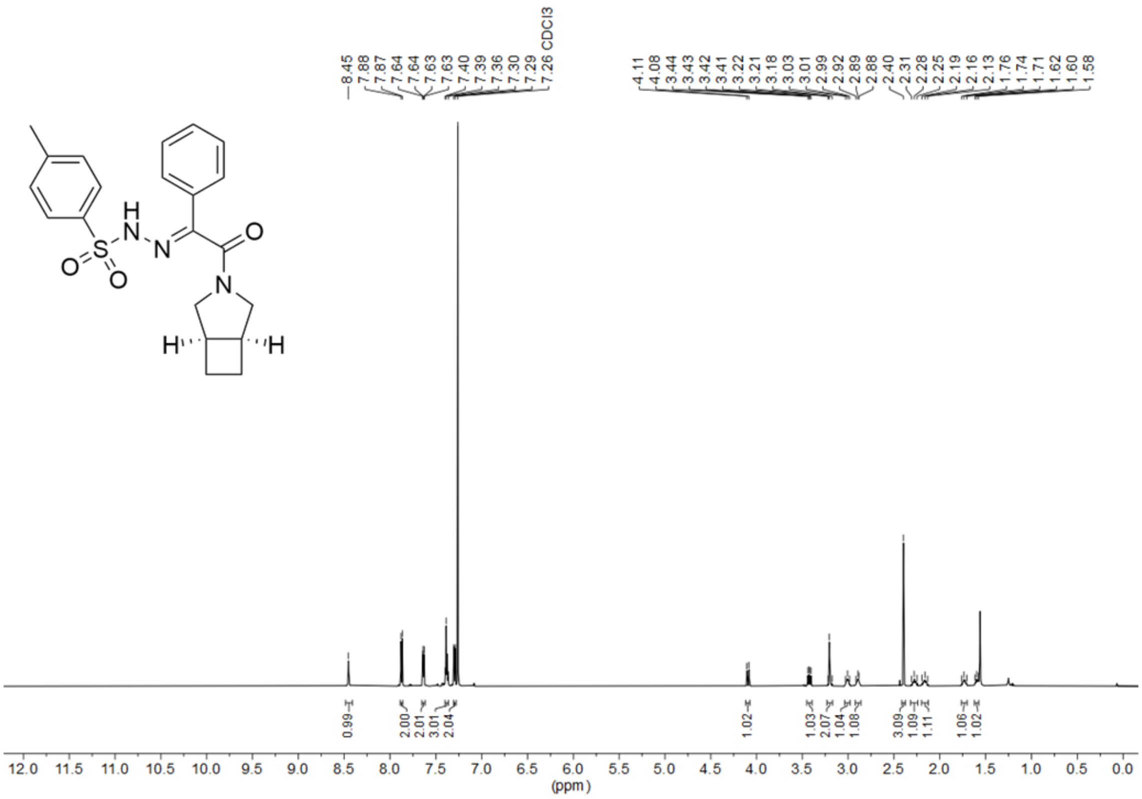

Figure S34: ^{1}H-NMR of **7** (600 MHz, $CDCl_3$).

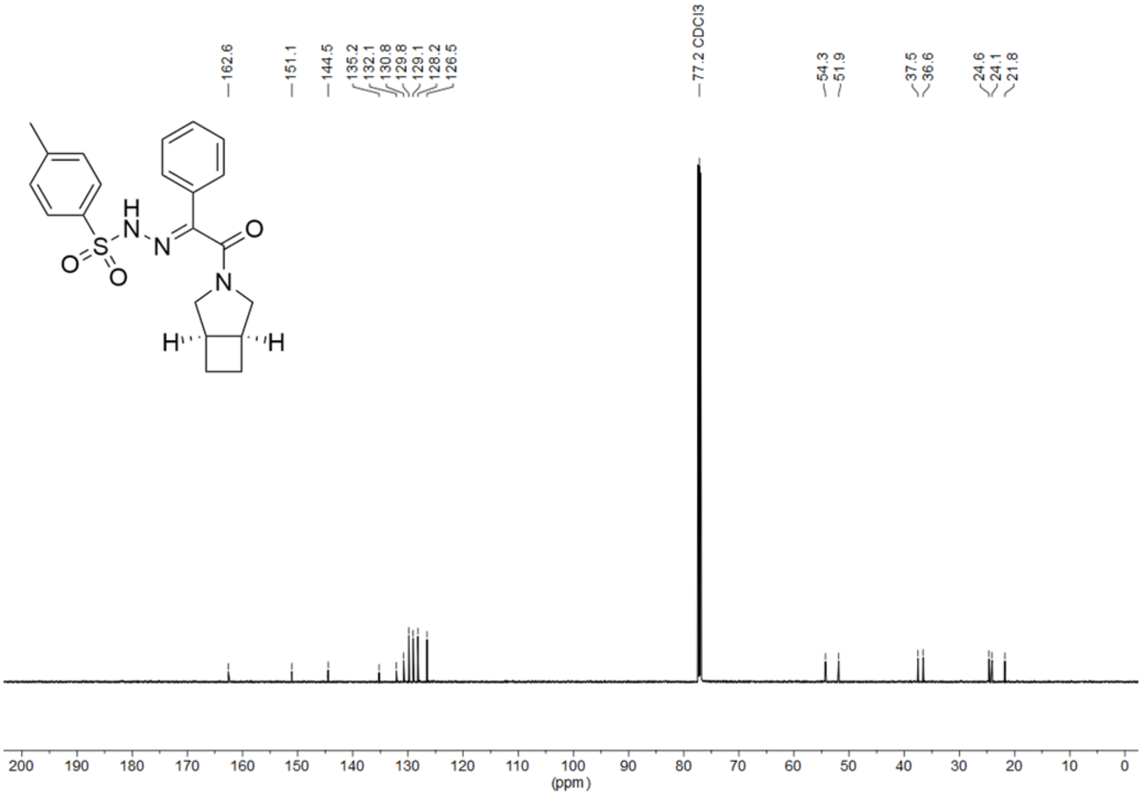

Figure S35: $^{13}C\{^{1}H\}$-NMR of **7** (151 MHz, $CDCl_3$).

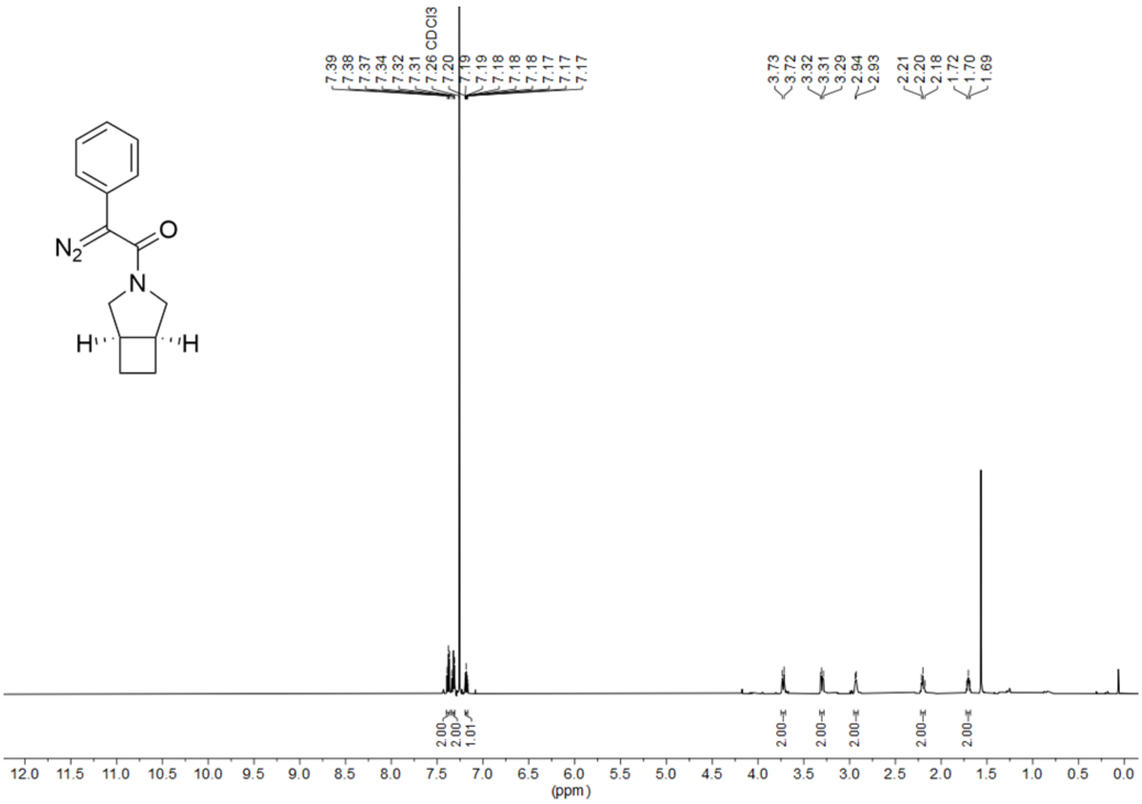

Figure S36: ^{1}H-NMR of **8** (600 MHz, $CDCl_3$).

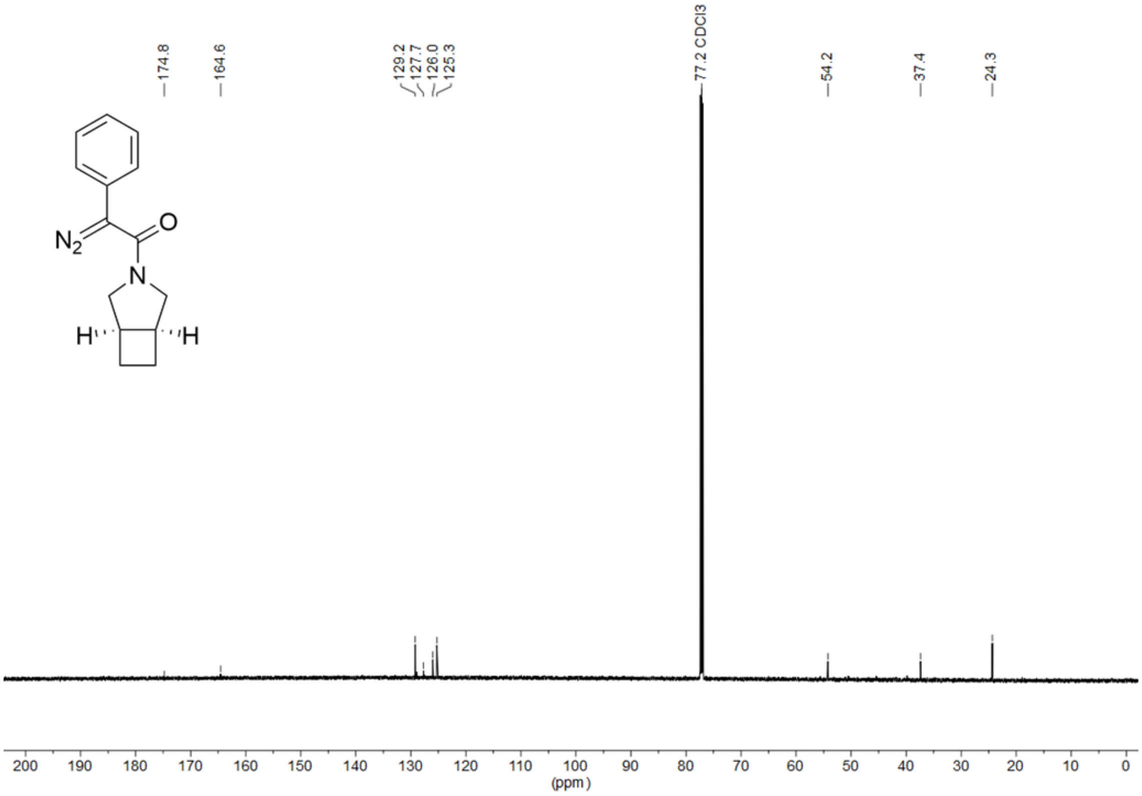

Figure S37: ^{13}C{^{1}H}-NMR of **8** (151 MHz, $CDCl_3$).

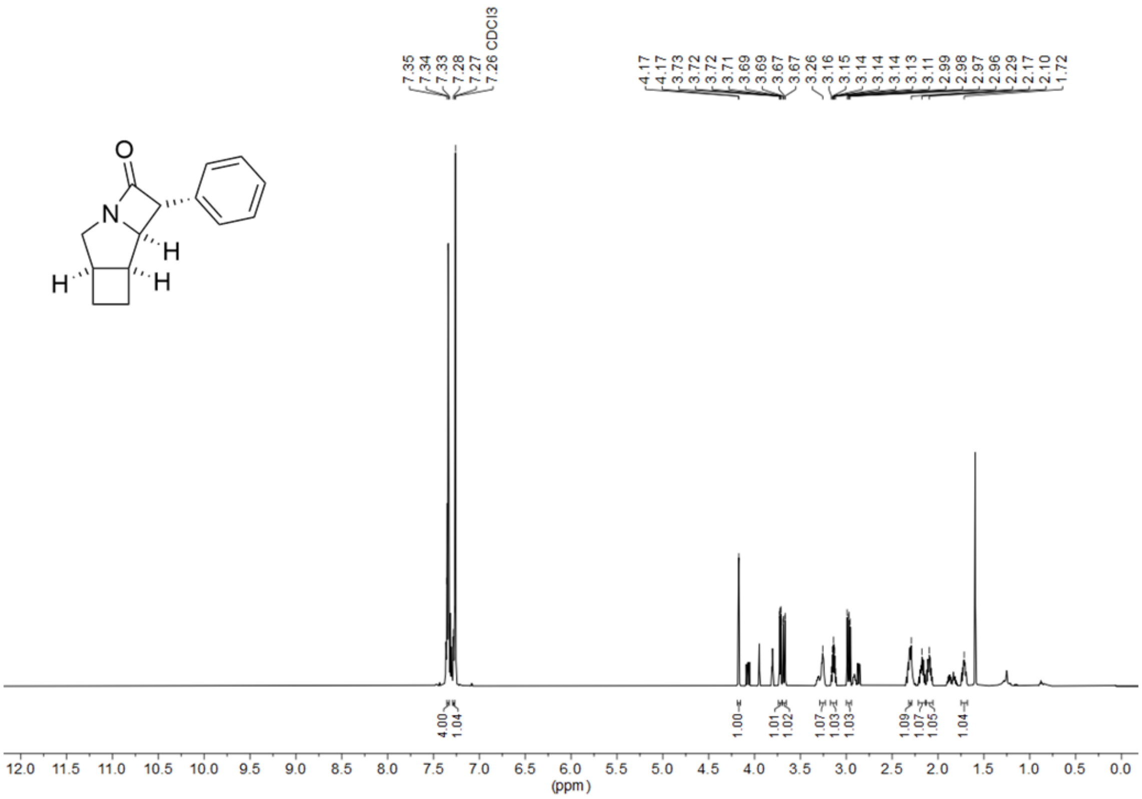

Figure S38: ^{1}H-NMR of **9a** (600 MHz, $CDCl_3$).

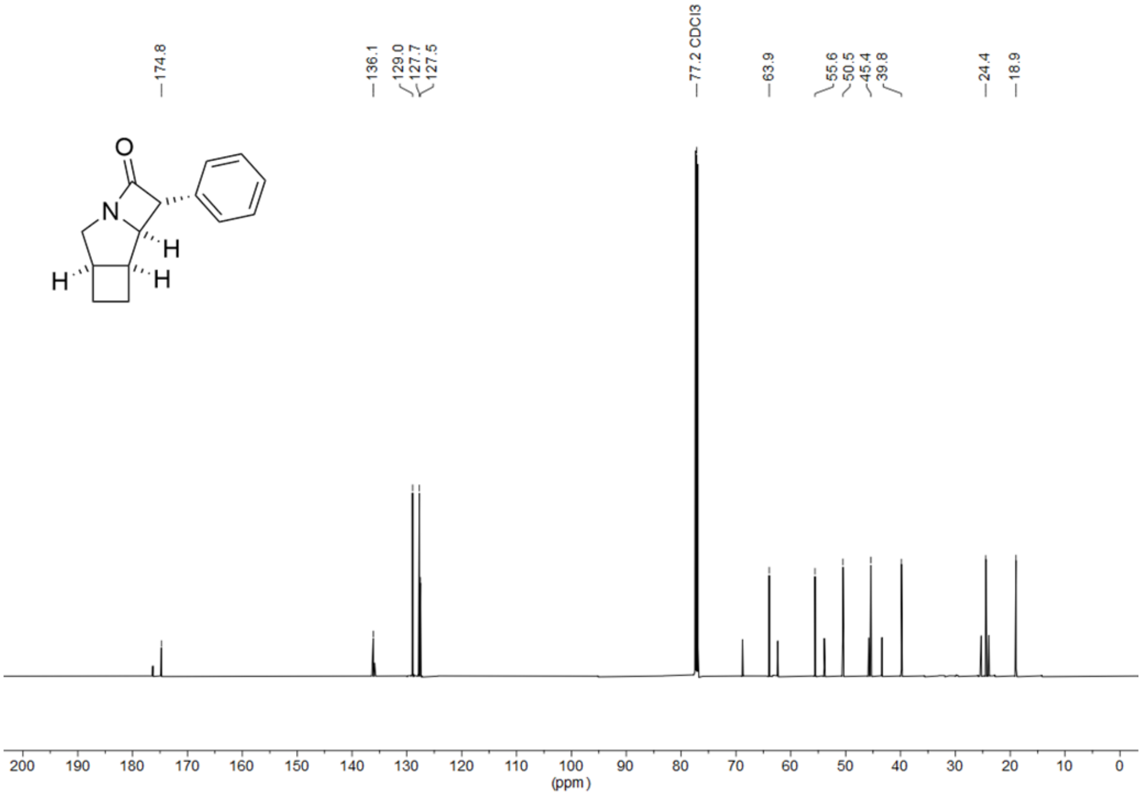

Figure S39: $^{13}C\{^1H\}$-NMR of **9a** (151 MHz, $CDCl_3$).

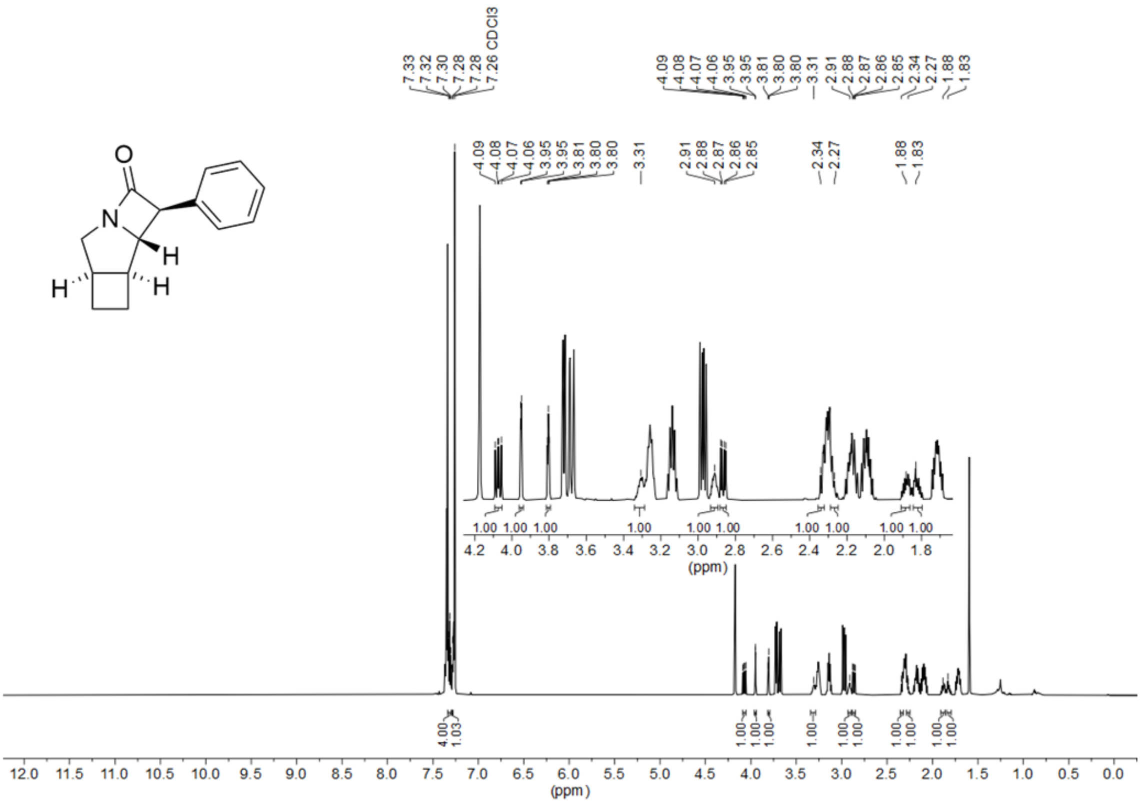

Figure S40: ^{1}H-NMR of **9b** (600 MHz, $CDCl_3$).

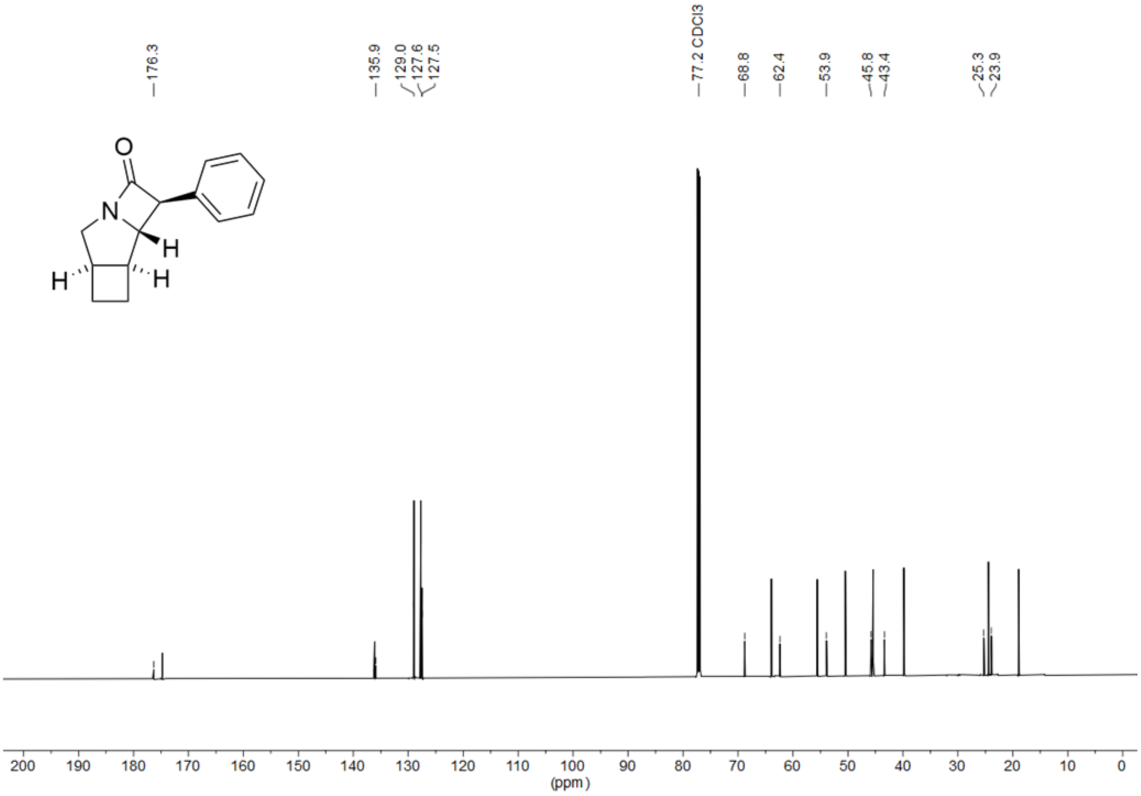

Figure S41: ^{13}C{^{1}H}-NMR of **9b** (151 MHz, $CDCl_3$).

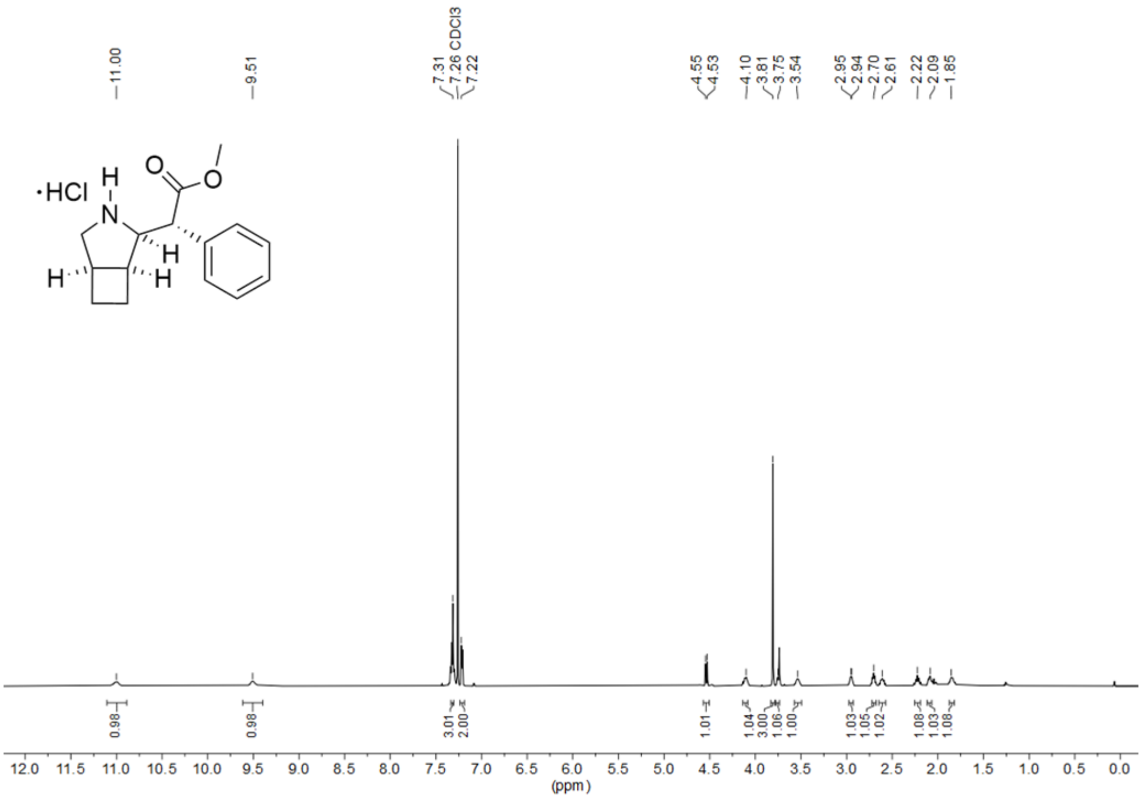

Figure S42: ^{1}H-NMR of **20** (600 MHz, $CDCl_3$).

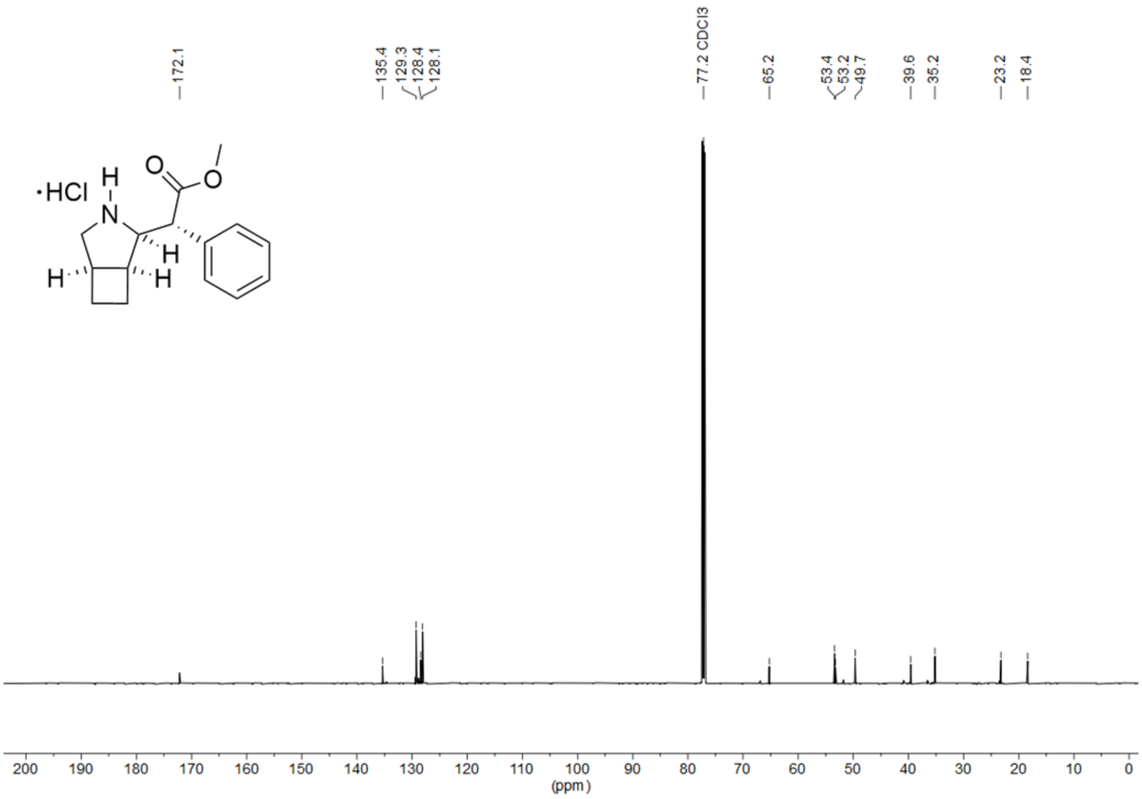

Figure S43: $^{13}C\{^{1}H\}$-NMR of **20** (151 MHz, $CDCl_3$).

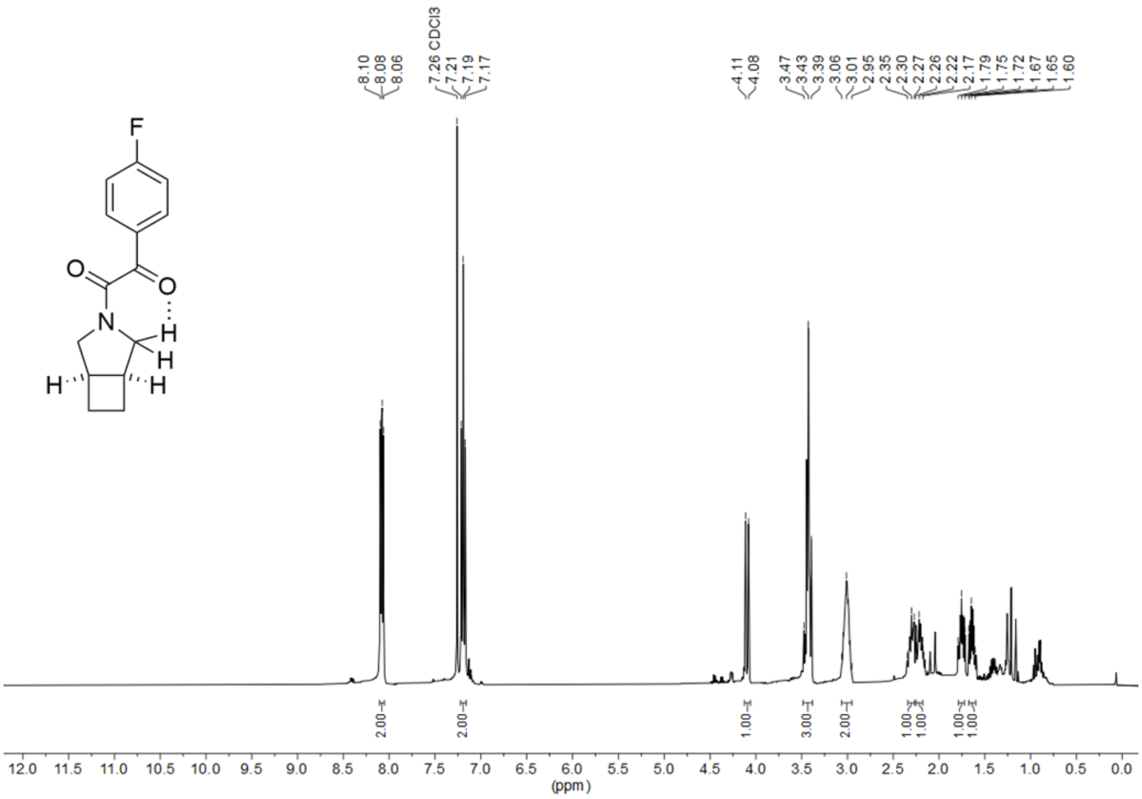

Figure S44: ^{1}H-NMR of **S1** (400 MHz, $CDCl_3$).

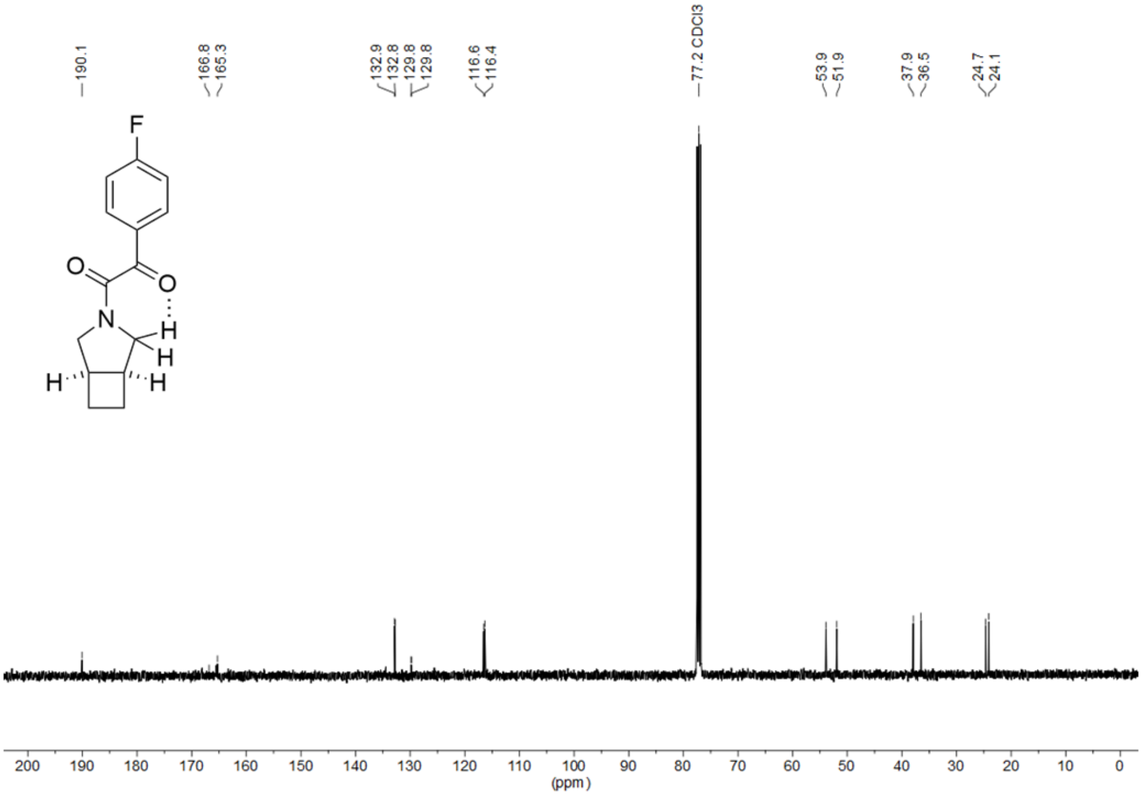

Figure S45: ^{13}C{^{1}H}-NMR of **S1** (101 MHz, $CDCl_3$).

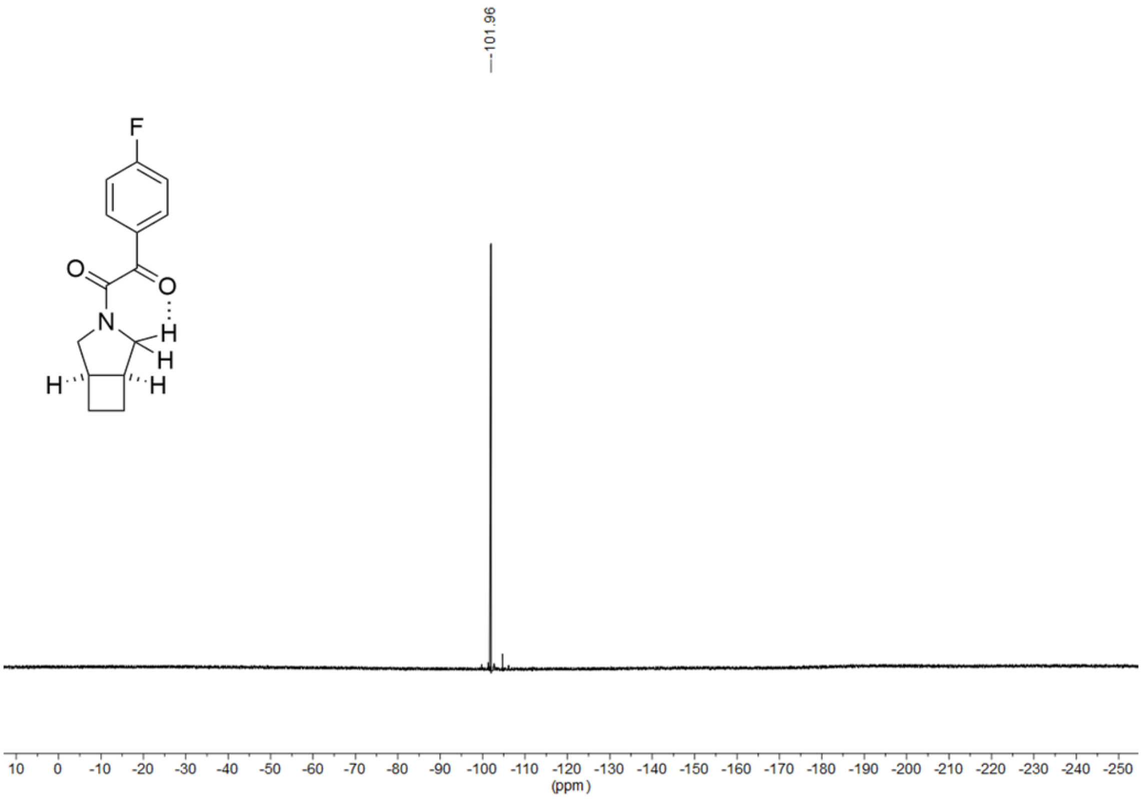

Figure S46: $^{19}F\{^{1}H\}$-NMR of **S1** (282 MHz, $CDCl_3$).

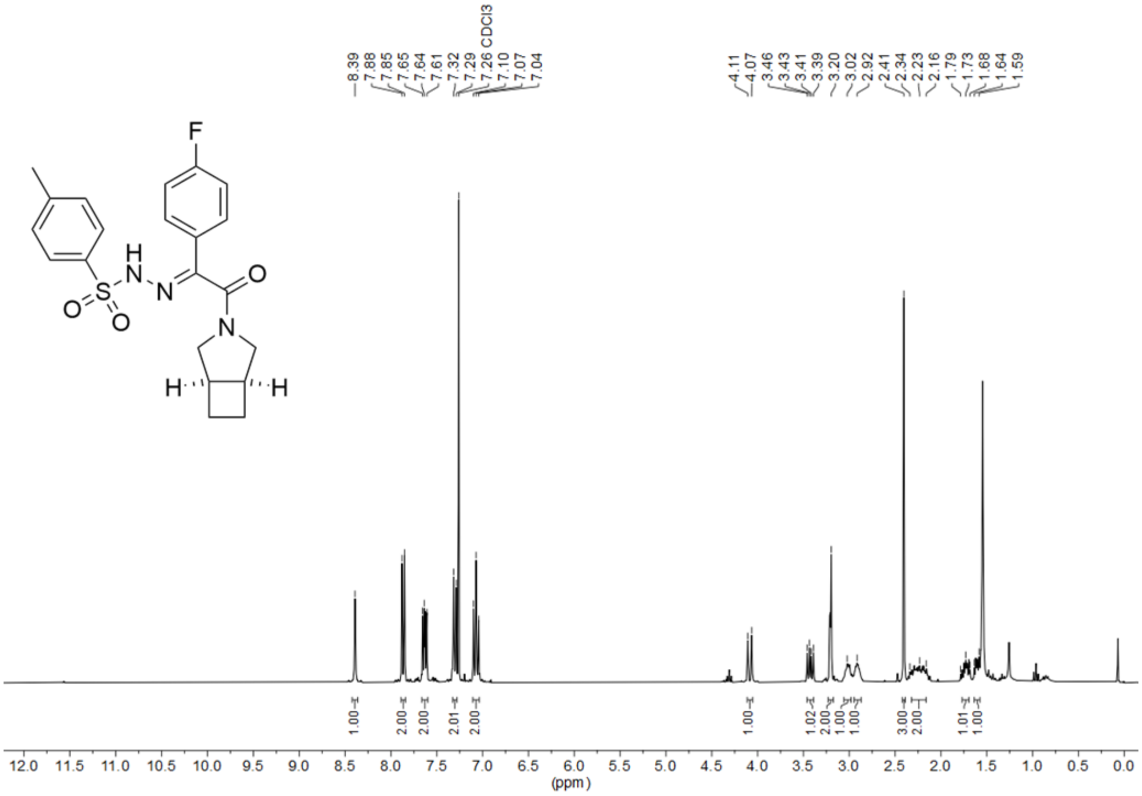

Figure S47: ^{1}H-NMR of **S2** (300 MHz, $CDCl_3$).

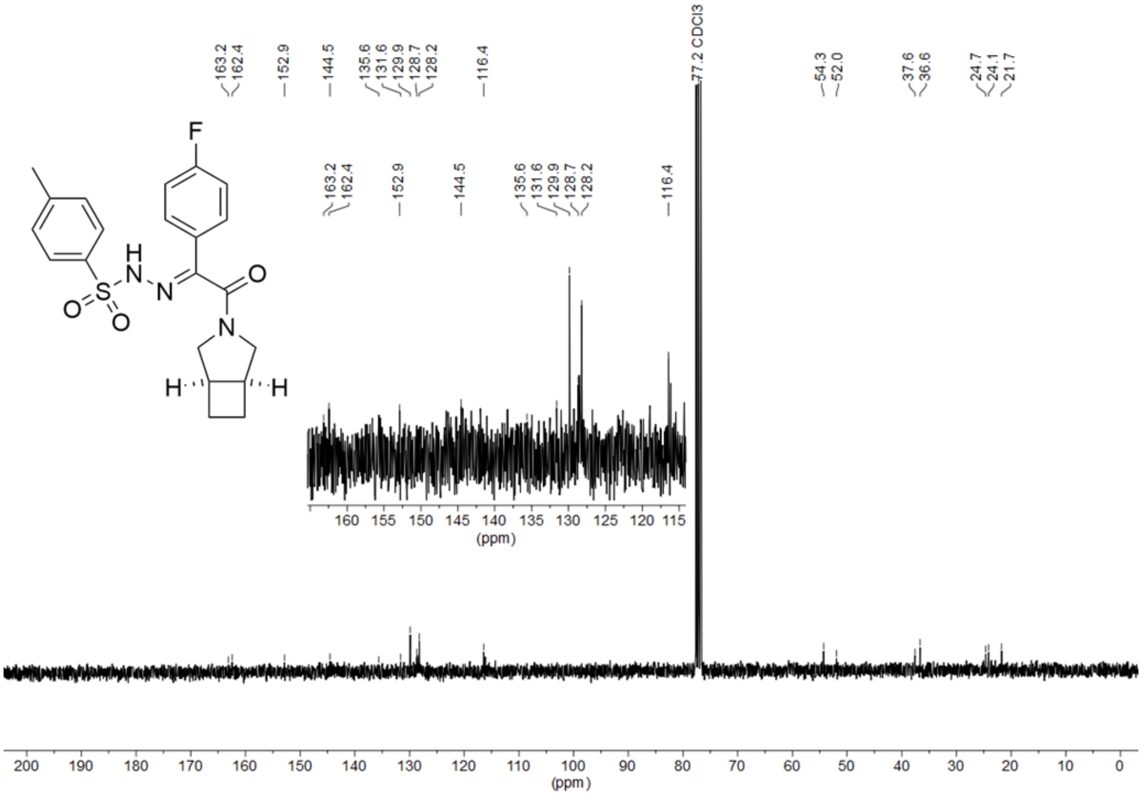

Figure S48: $^{13}C\{^{1}H\}$-NMR of **S2** (75 MHz, $CDCl_3$).

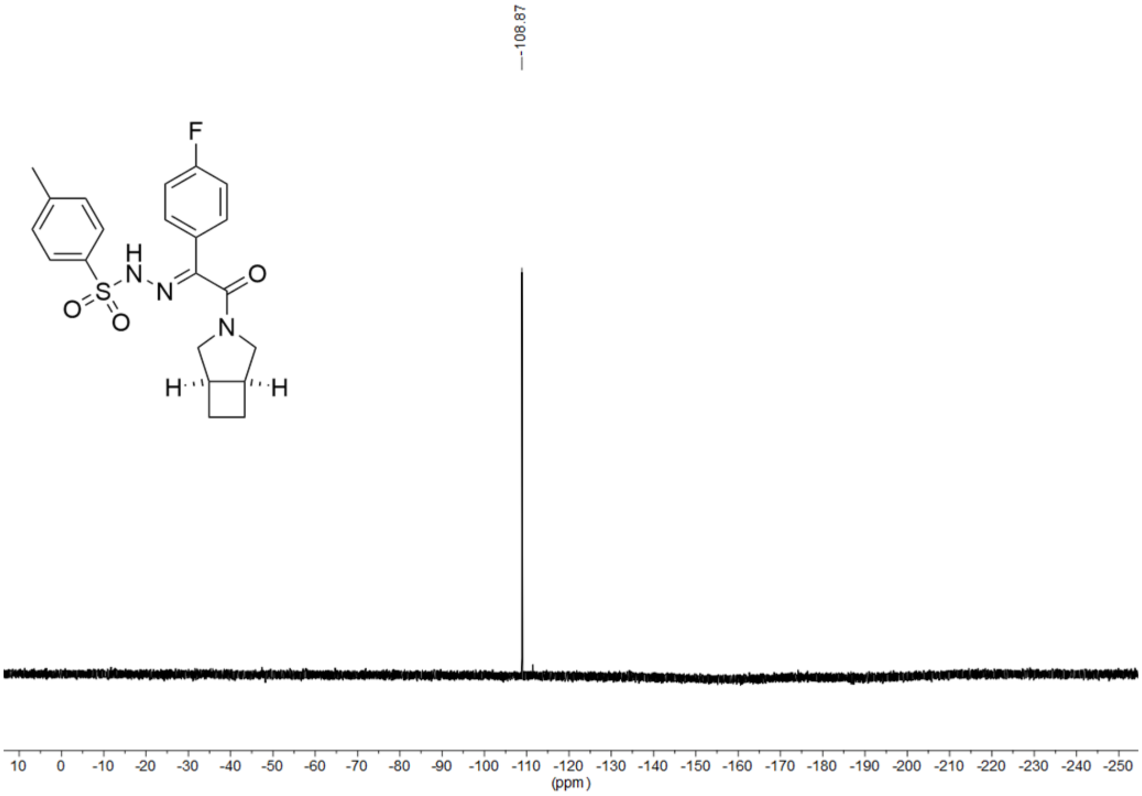

Figure S49: $^{19}F\{^{1}H\}$-NMR of **S2** (282 MHz, $CDCl_3$).

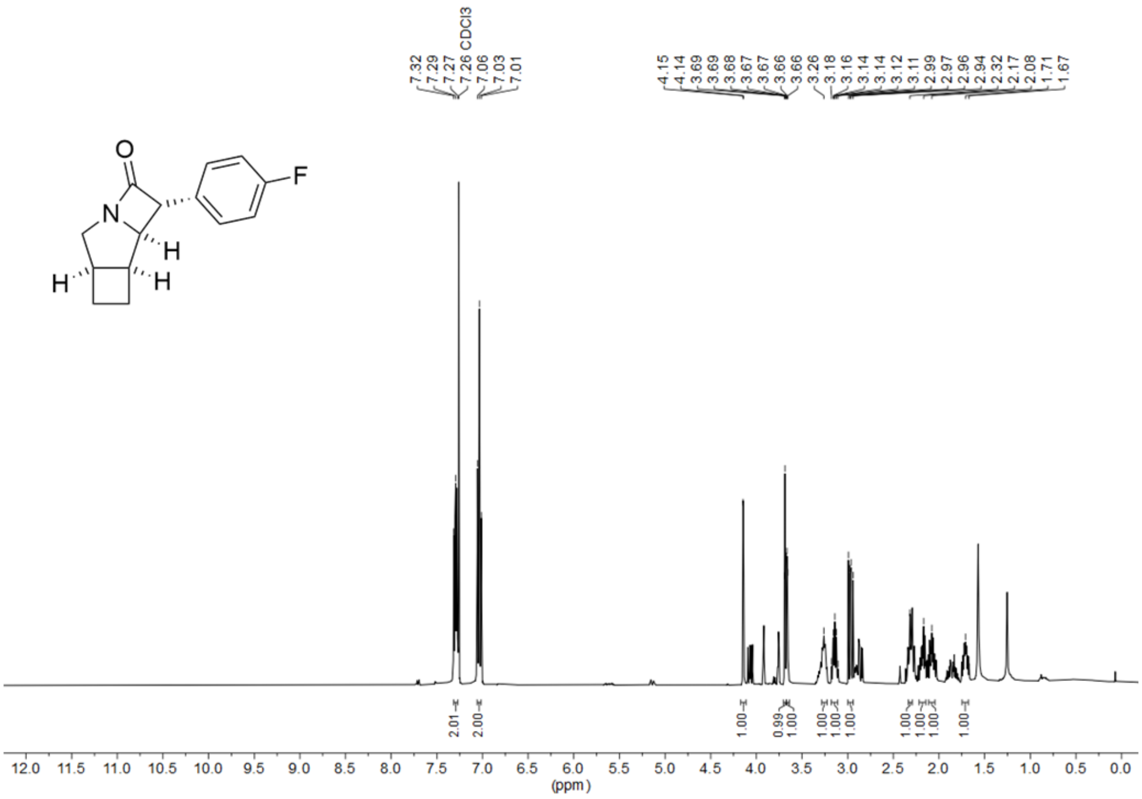

Figure S50: ^{1}H-NMR of **10a** (400 MHz, $CDCl_3$).

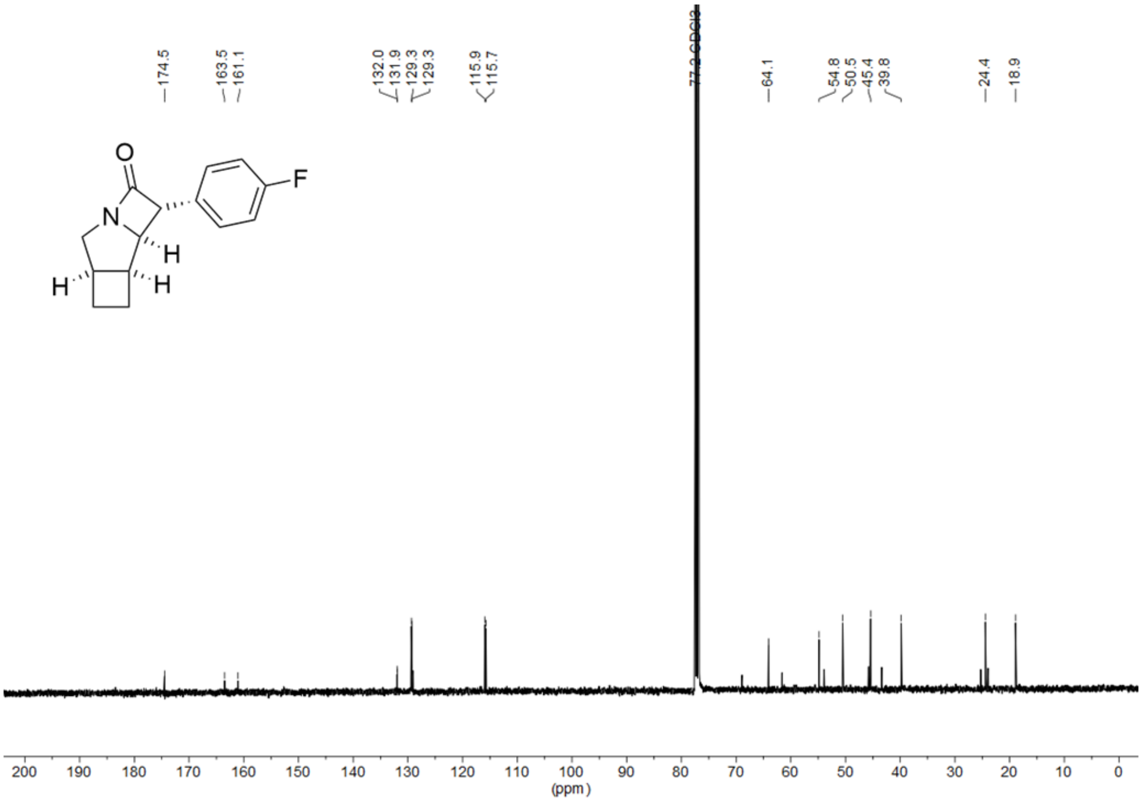

Figure S51: ^{13}C{^{1}H}-NMR of **10a** (101 MHz, $CDCl_3$).

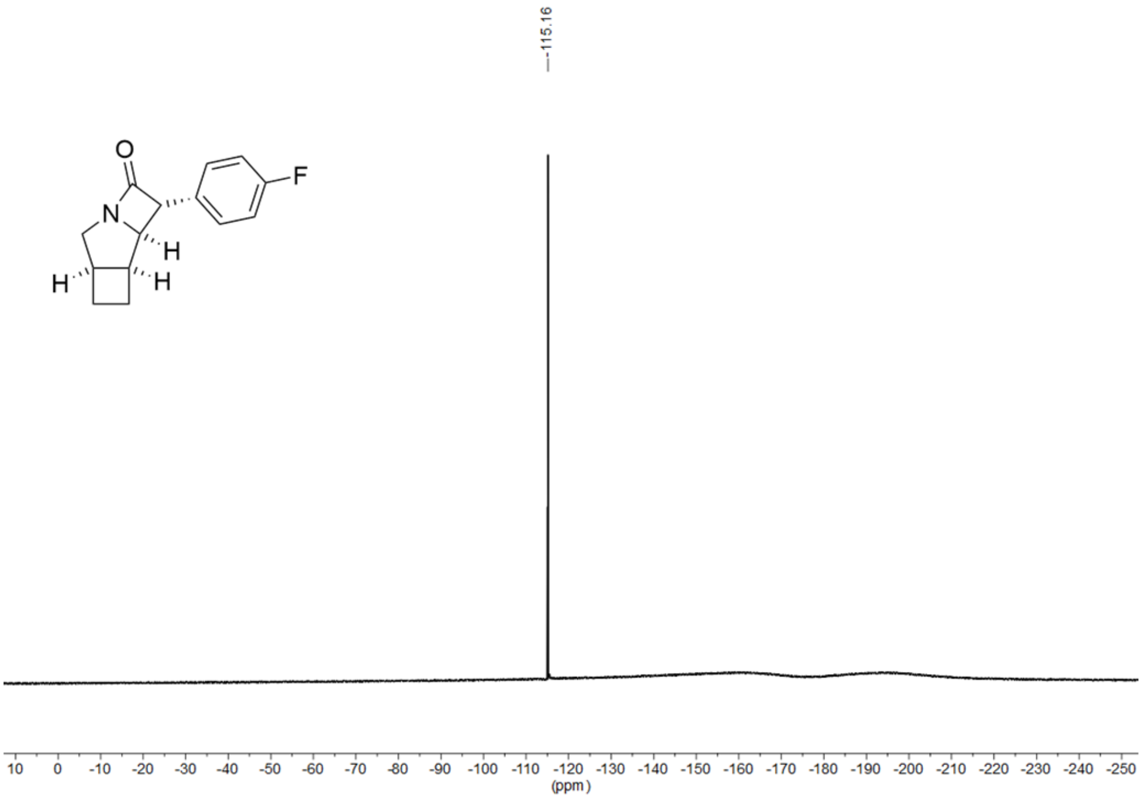

Figure S52: $^{19}F\{^{1}H\}$-NMR of **10a** (376 MHz, $CDCl_3$).

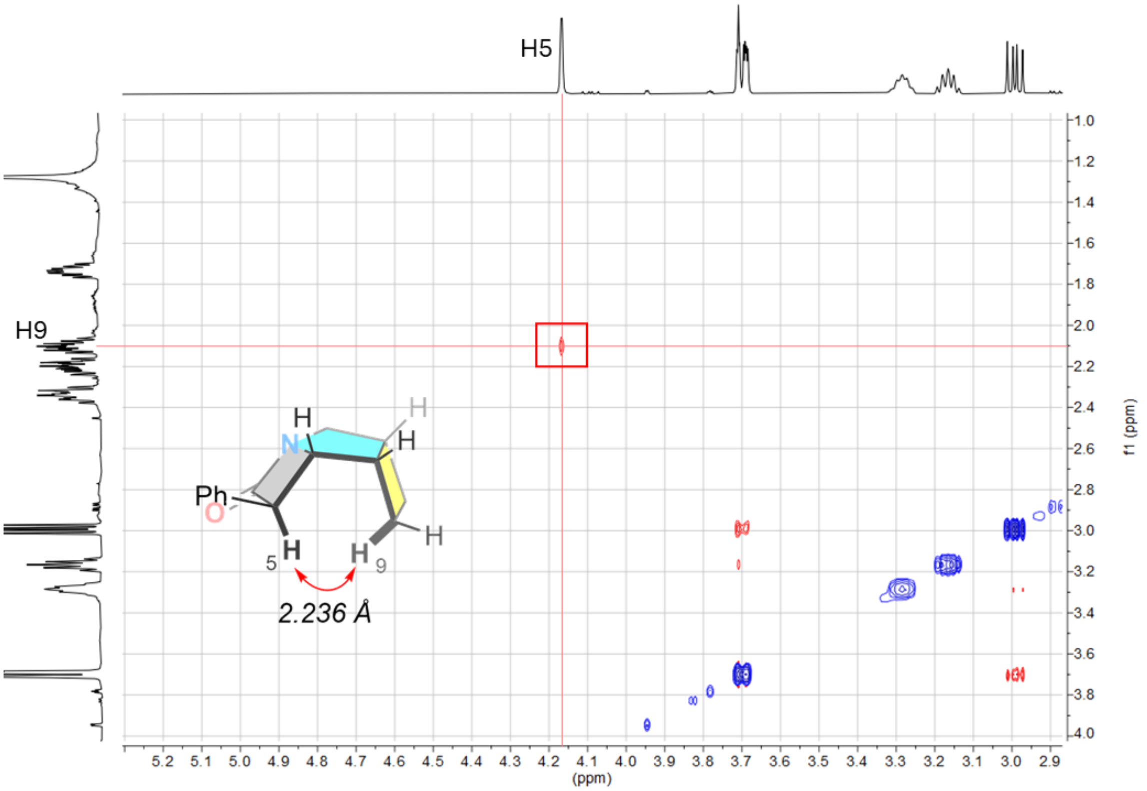

Figure S53: NOESY spectrum of **10a** (500 MHz, $CDCl_3$).

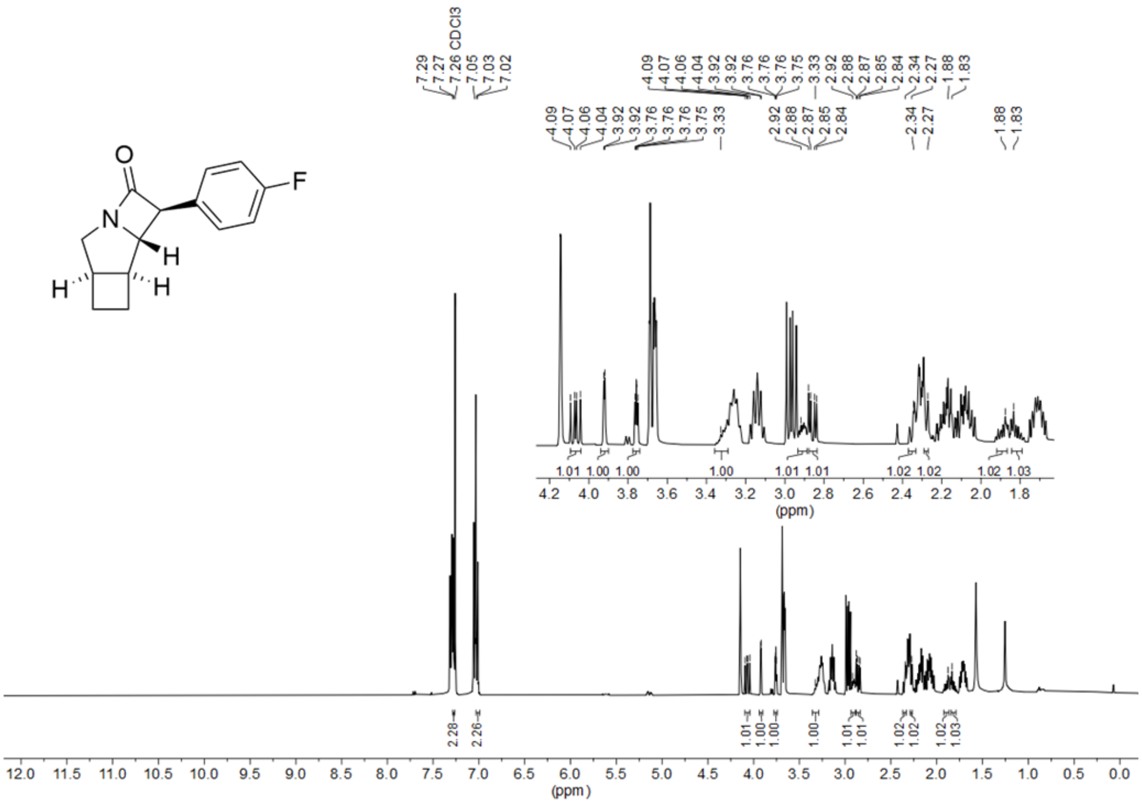

Figure S54: ^{1}H-NMR of **10b** (400 MHz, $CDCl_3$).

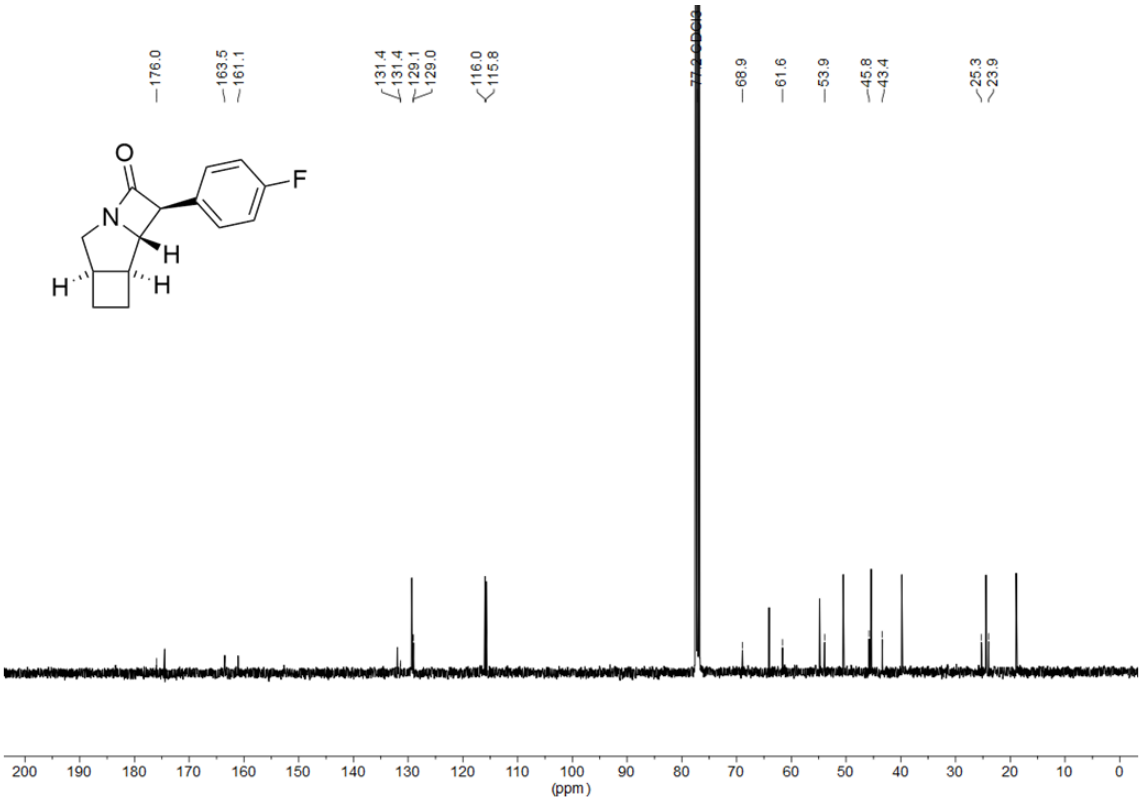

Figure S55: $^{13}C\{^{1}H\}$-NMR of **10b** (101 MHz, $CDCl_3$).

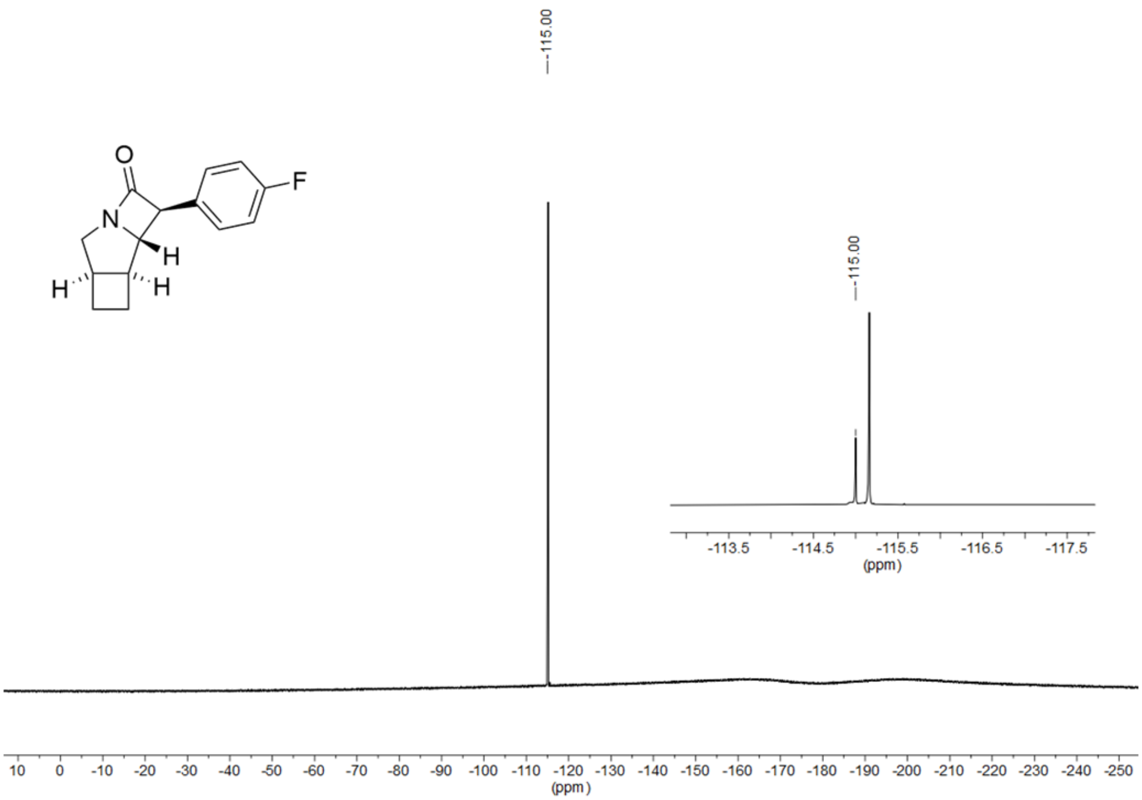

Figure S56: $^{19}F\{^{1}H\}$-NMR of **10b** (376 MHz, $CDCl_3$).

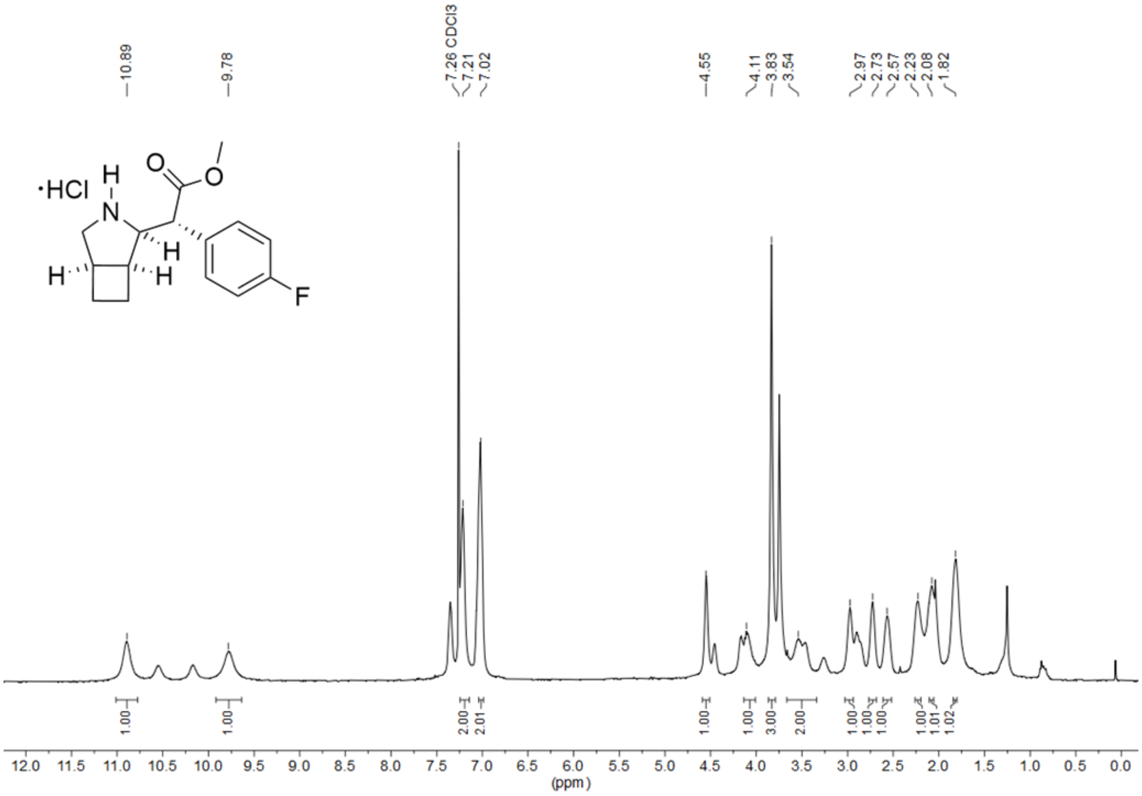

Figure S57: ^{1}H-NMR of **21** (400 MHz, $CDCl_3$).

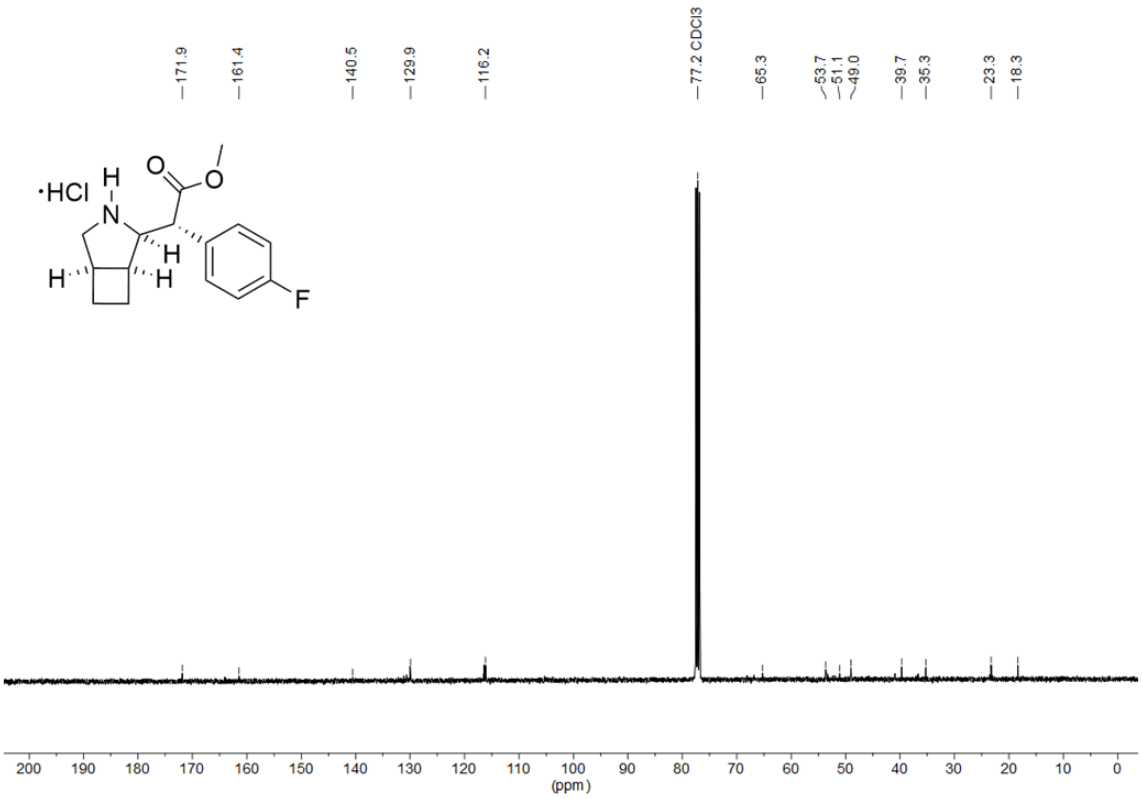

Figure S58: $^{13}C\{^{1}H\}$-NMR of **21** (101 MHz, $CDCl_3$).

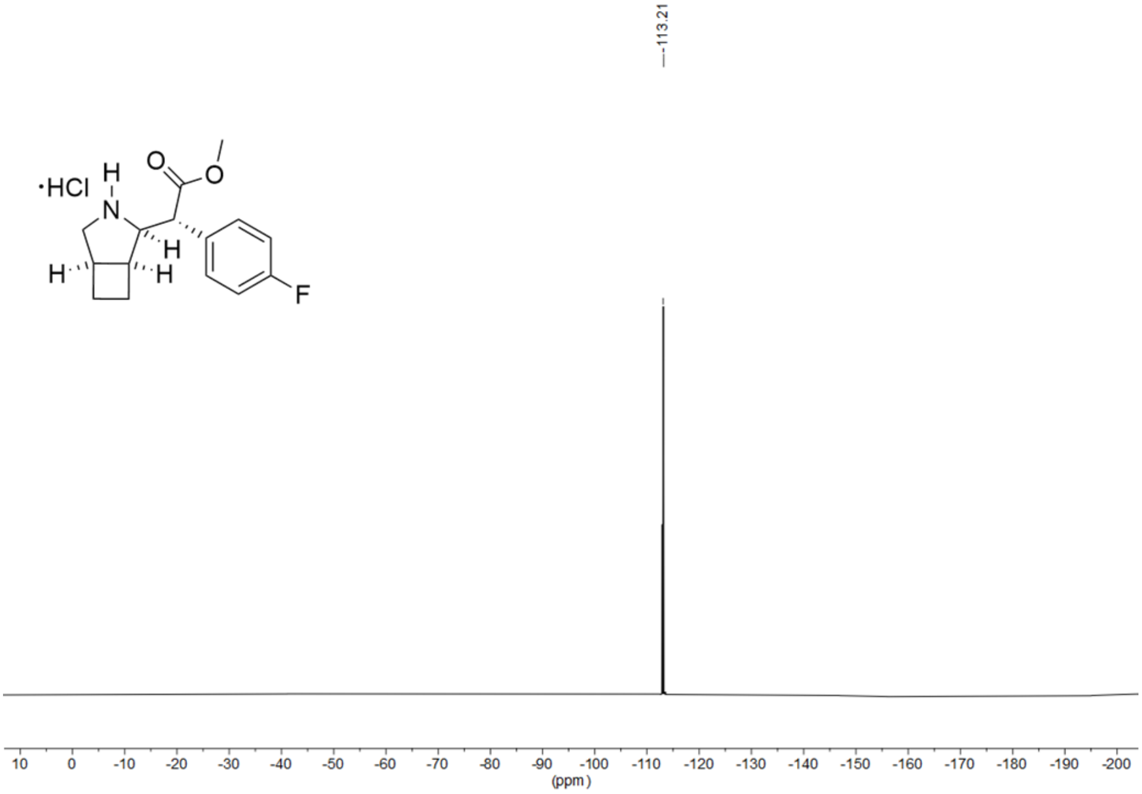

Figure S59: $^{19}F\{^{1}H\}$-NMR of **21** (376 MHz, $CDCl_3$).

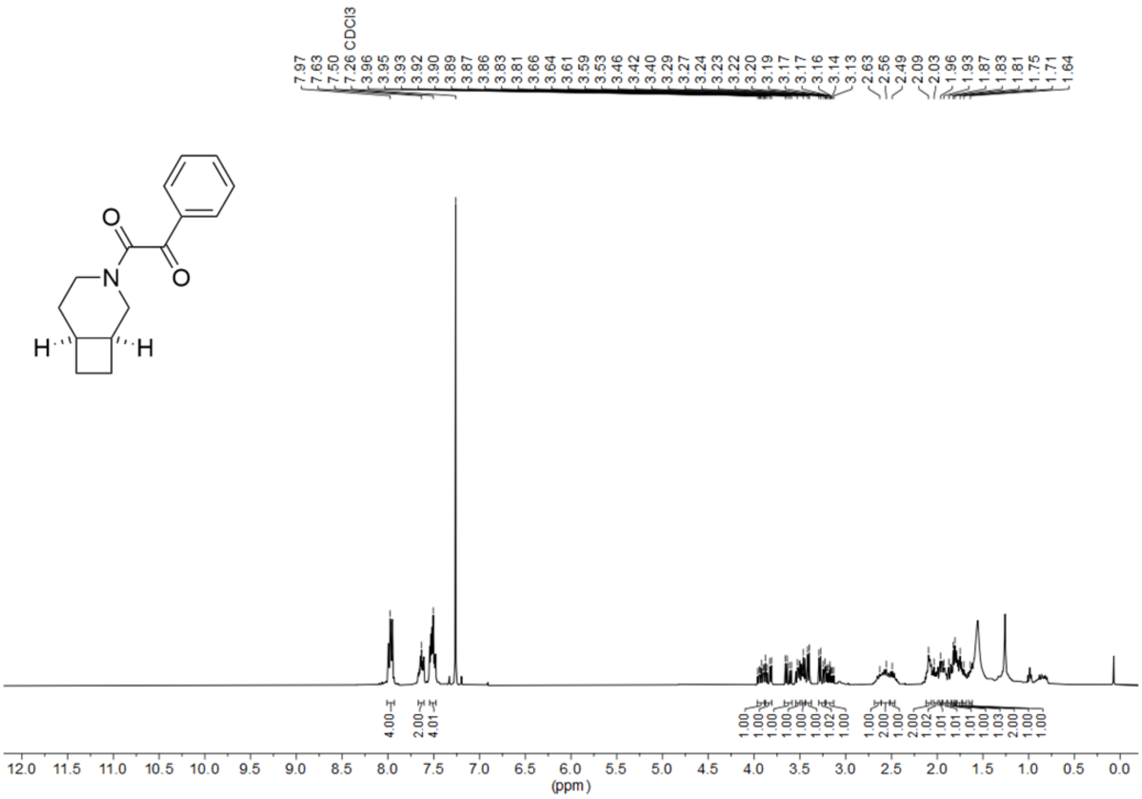

Figure S60: ^{1}H-NMR of **S3** (300 MHz, $CDCl_3$).

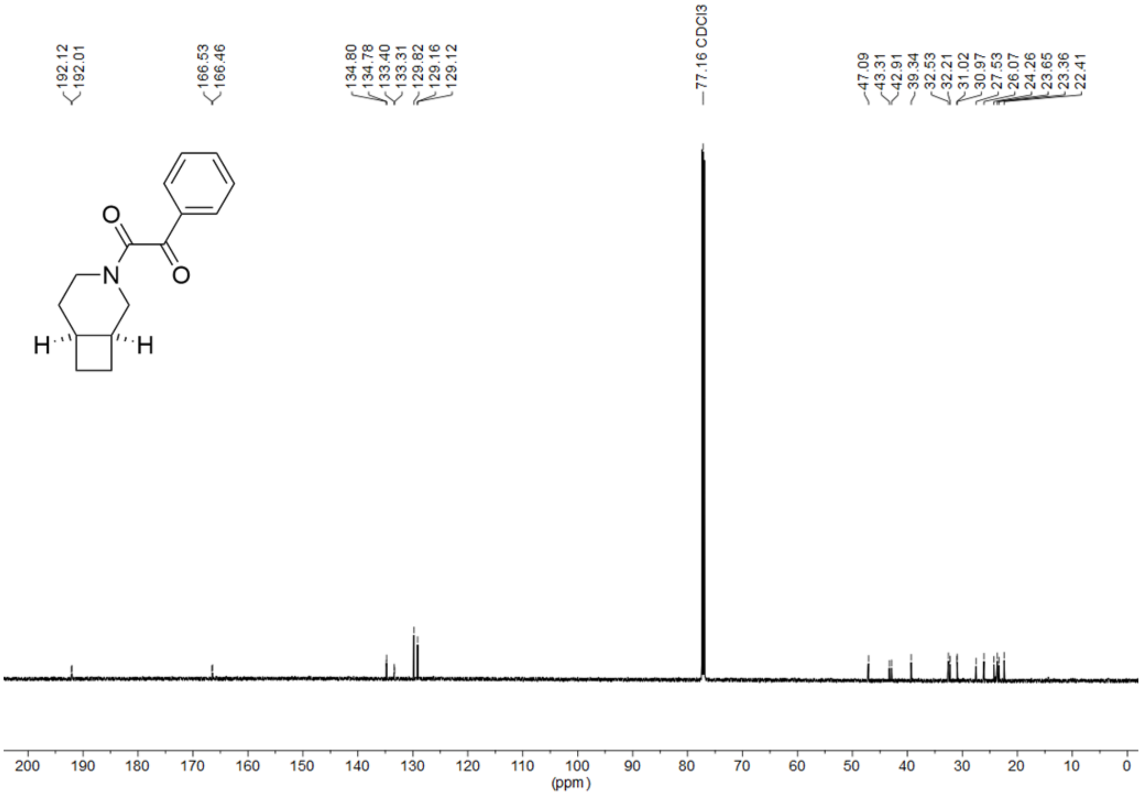

Figure S61: $^{13}C\{^{1}H\}$-NMR of **S3** (151 MHz, $CDCl_3$).

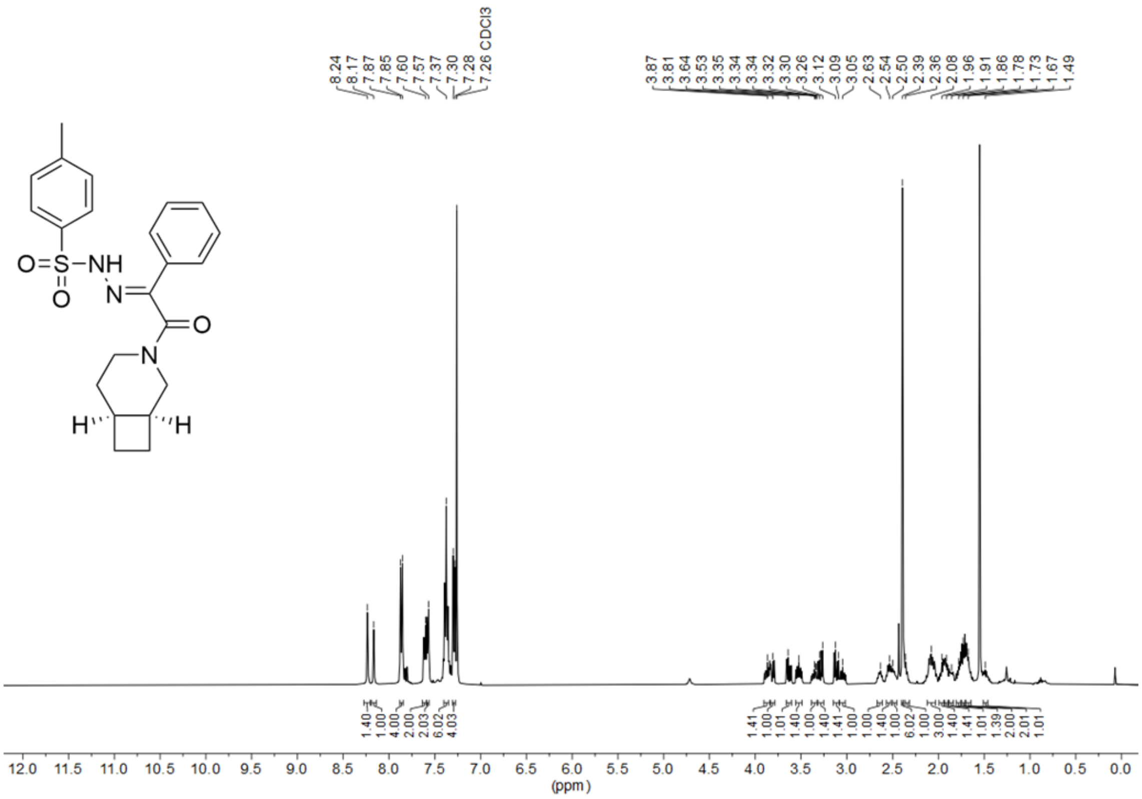

Figure S62: ^{1}H-NMR of **S4** (400 MHz, $CDCl_3$).

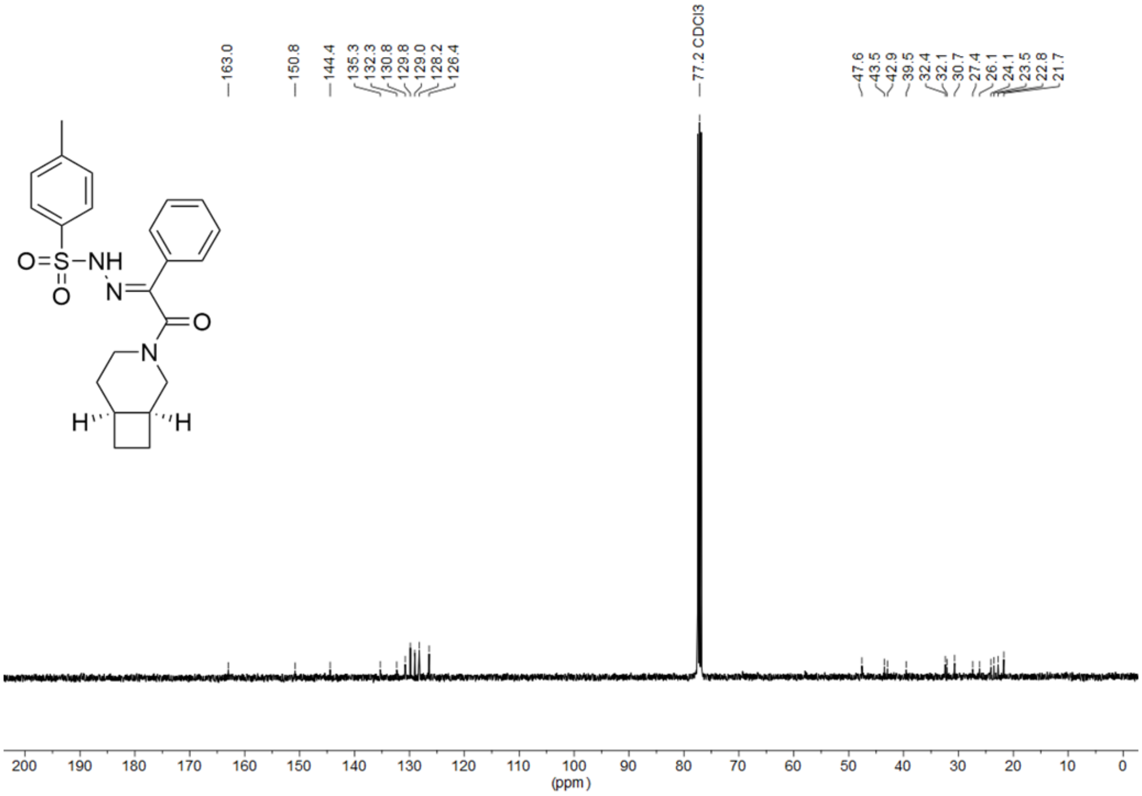

Figure S63: $^{13}C\{^{1}H\}$-NMR of **S4** (101 MHz, $CDCl_3$).

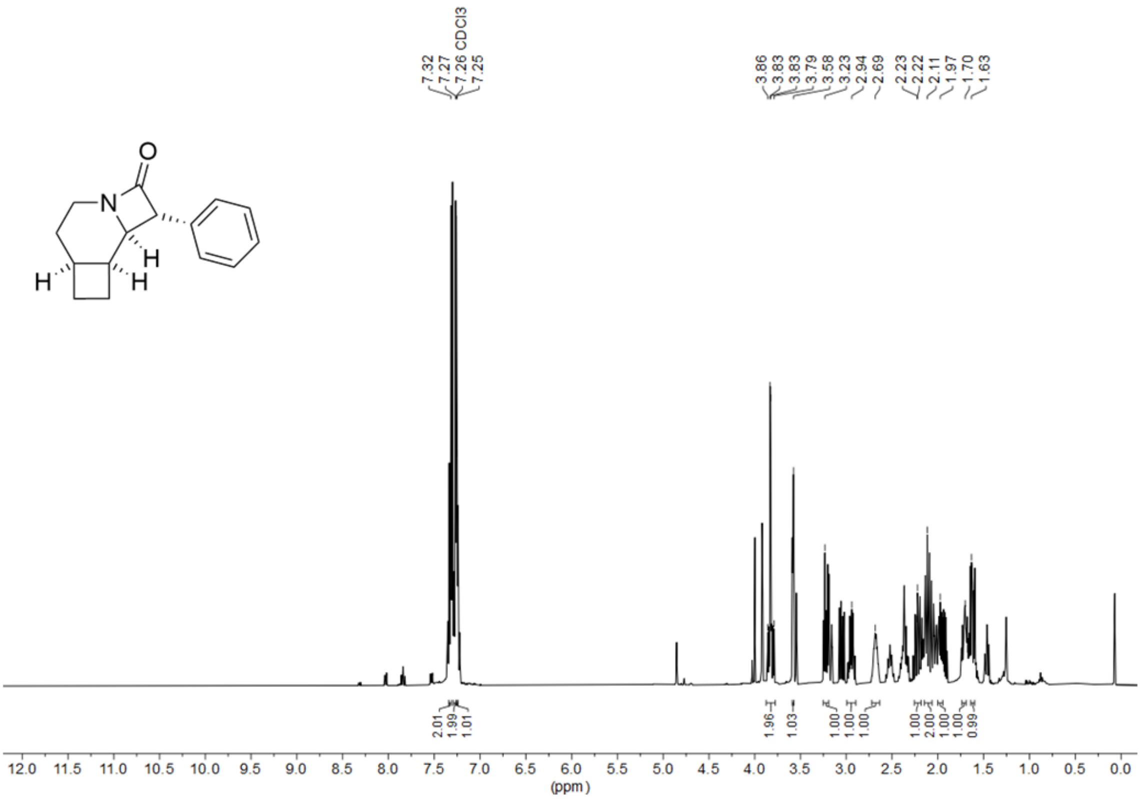

Figure S64: ^{1}H-NMR of **11a** (400 MHz, $CDCl_3$).

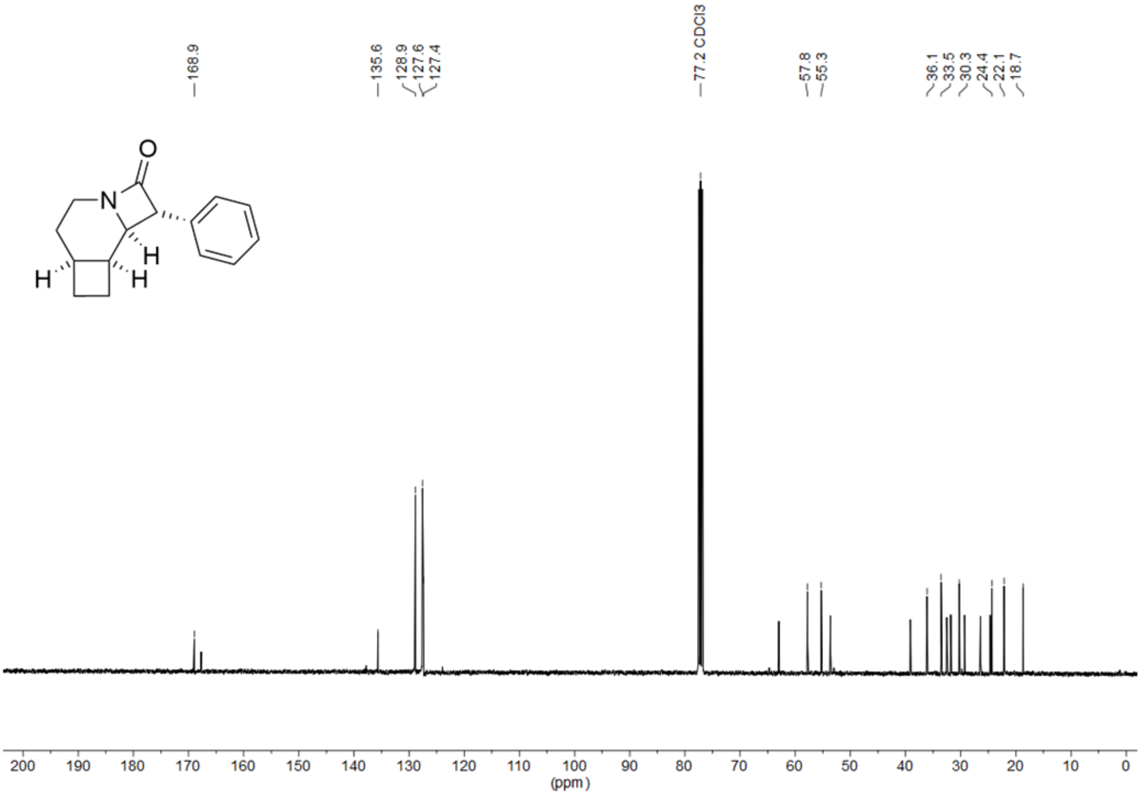

Figure S65: ^{13}C{^{1}H}-NMR of **11a** (101 MHz, $CDCl_3$).

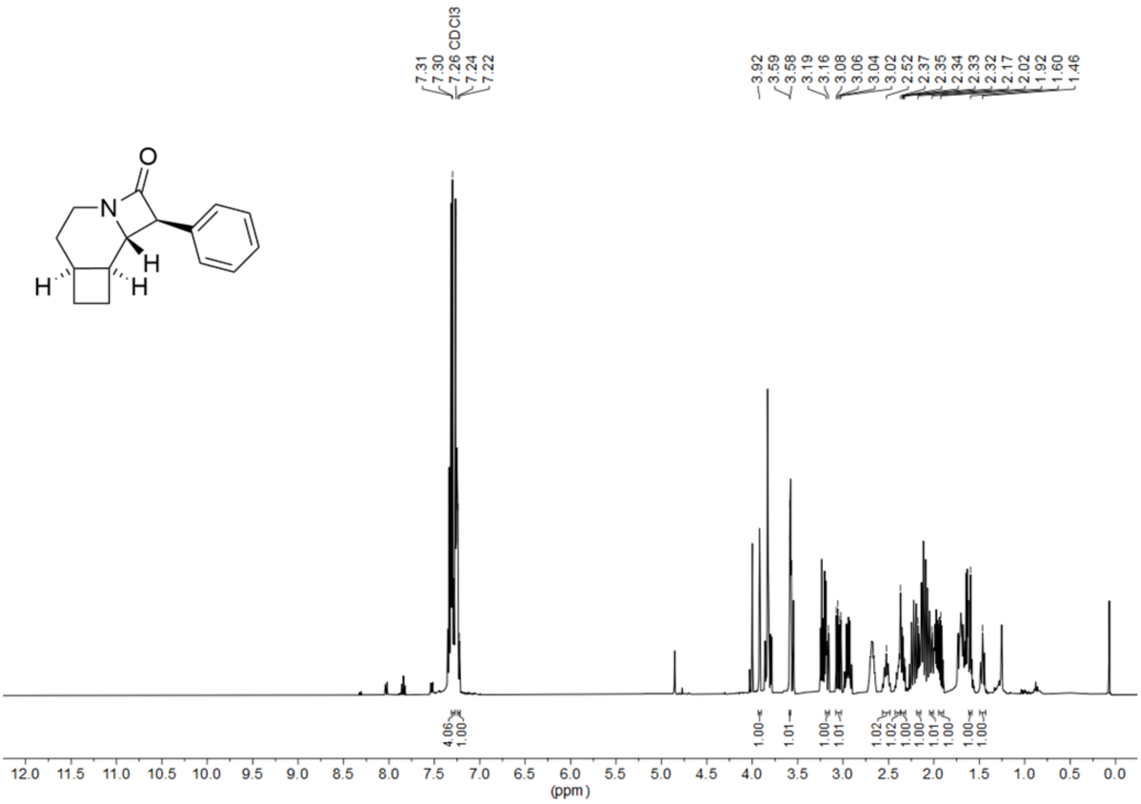

Figure S66: ^{1}H-NMR of **11b** (400 MHz, $CDCl_3$).

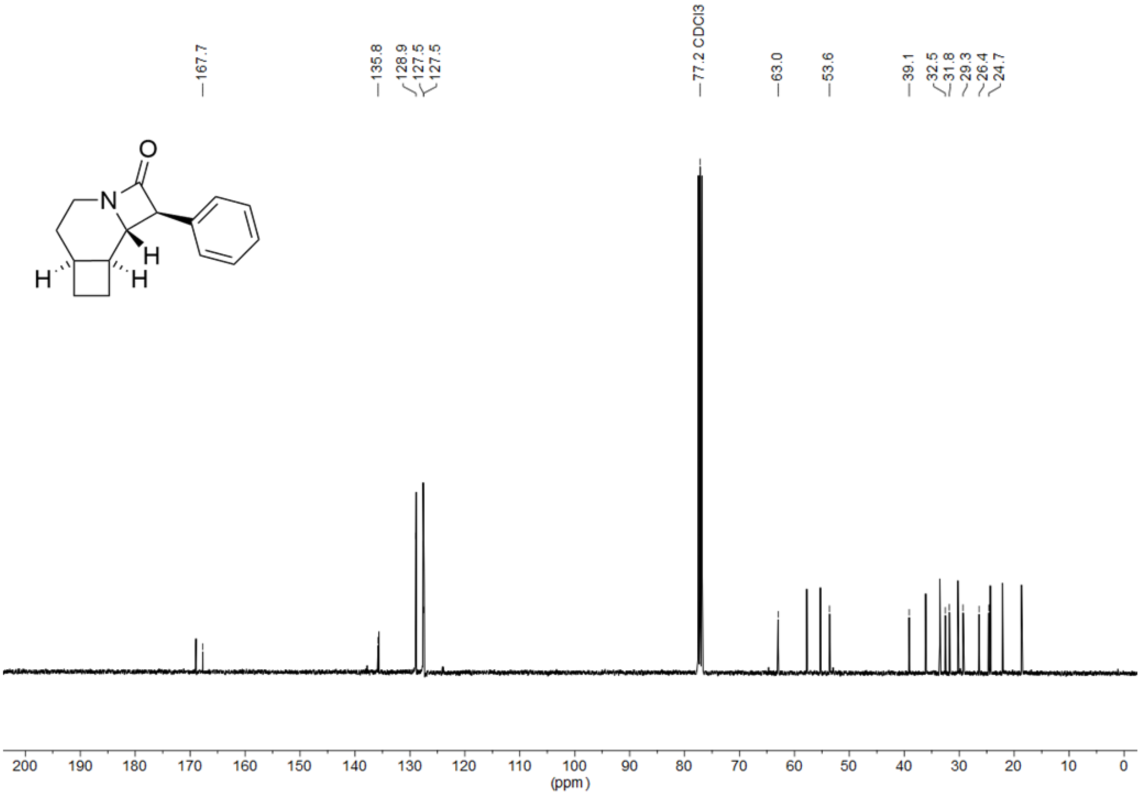

Figure S67: ^{13}C{^{1}H}-NMR of **11b** (101 MHz, $CDCl_3$).

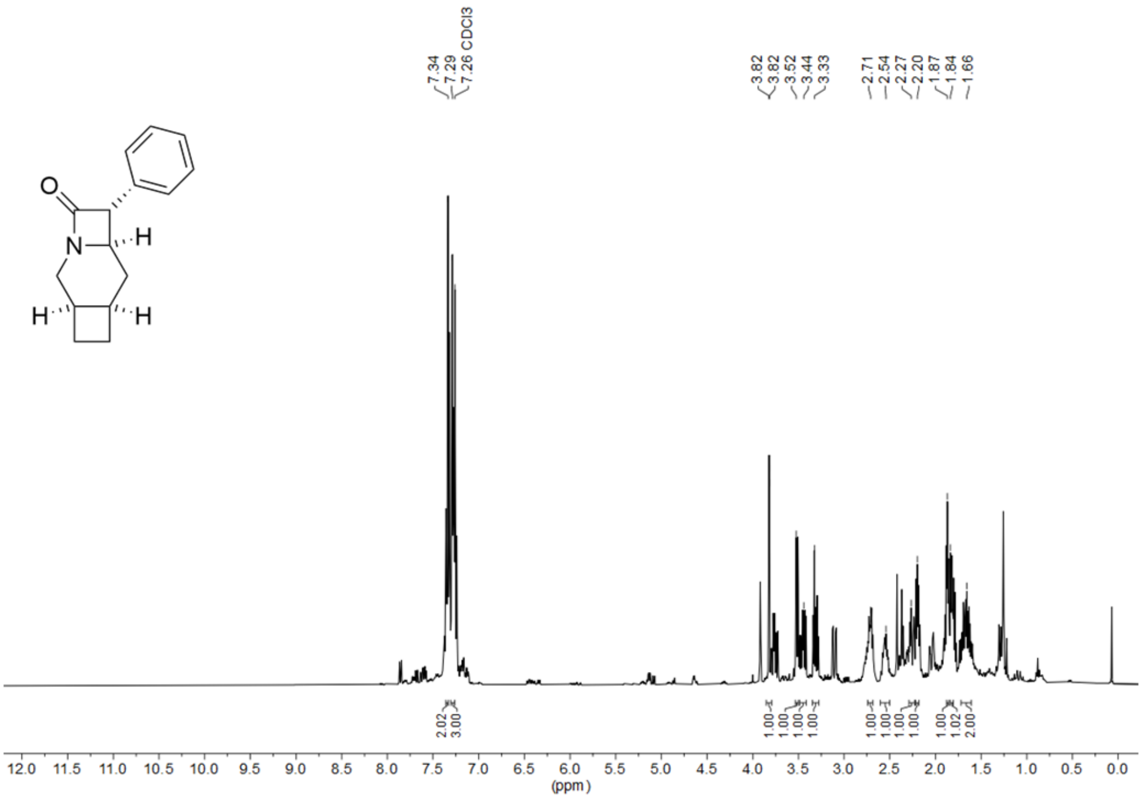

Figure S68: ^{1}H-NMR of **12a** (400 MHz, $CDCl_3$).

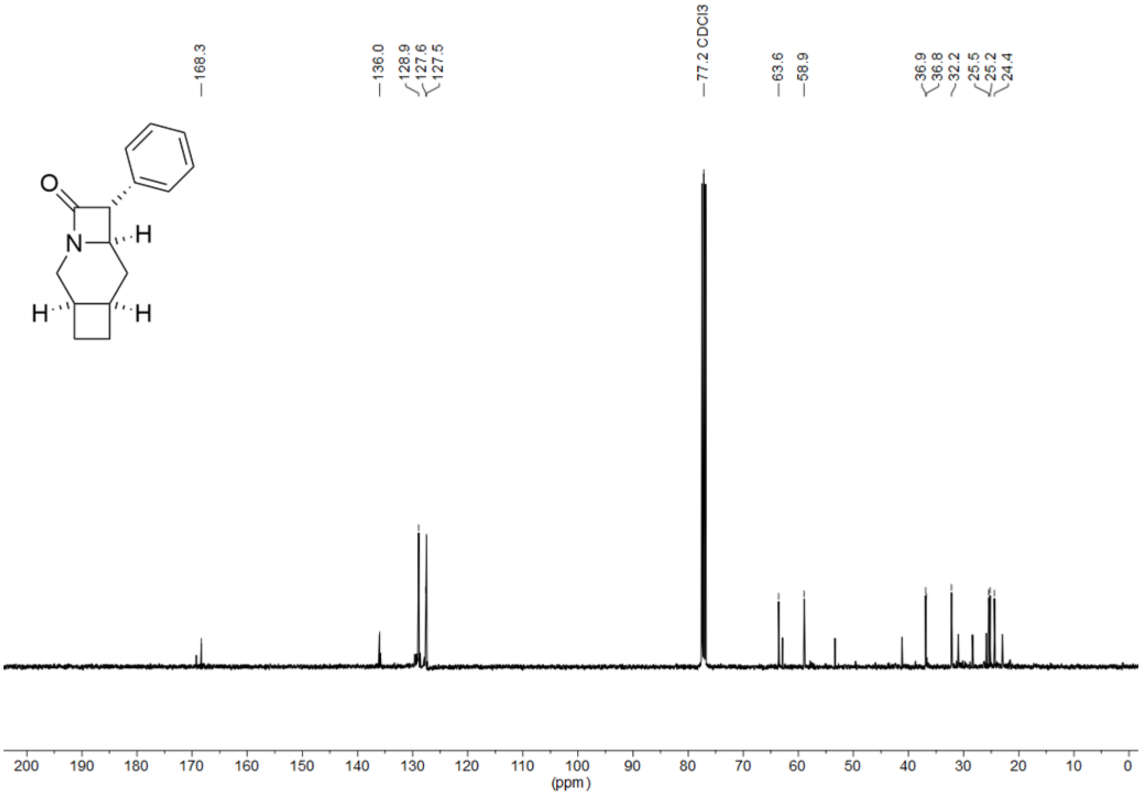

Figure S69: $^{13}C\{^{1}H\}$-NMR of **12a** (101 MHz, $CDCl_3$).

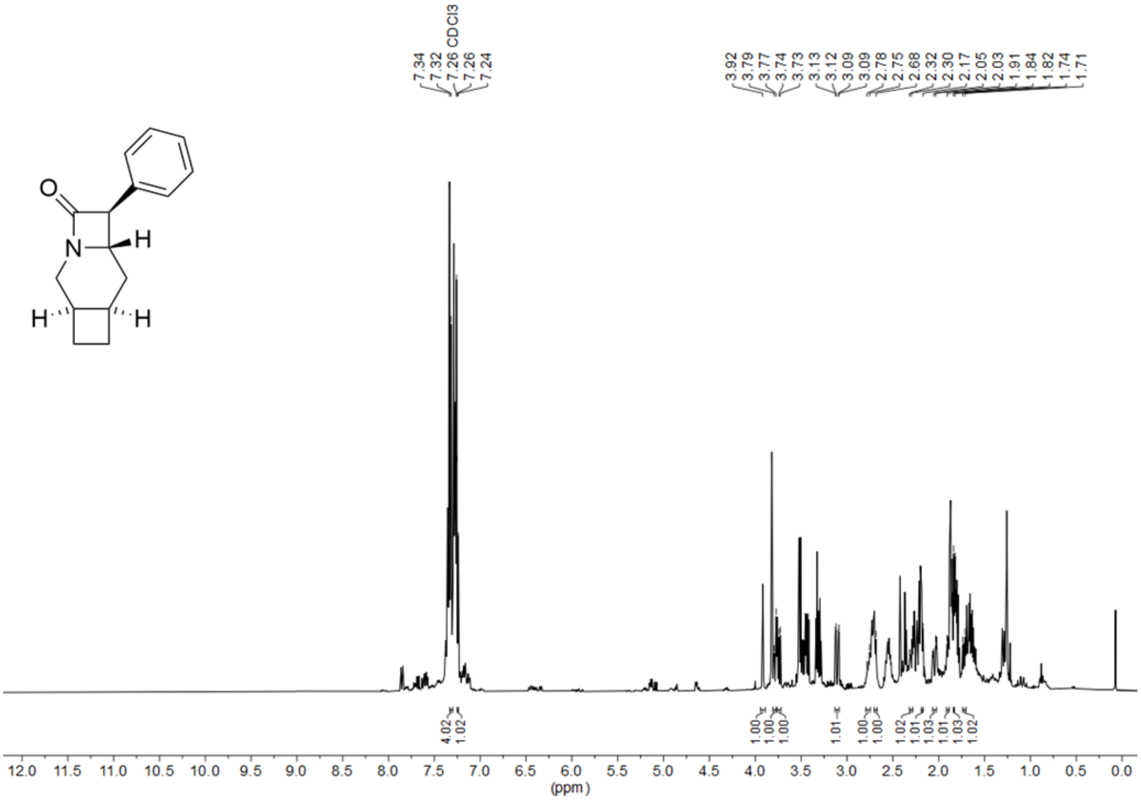

Figure S70: ^{1}H-NMR of **12b** (400 MHz, $CDCl_3$).

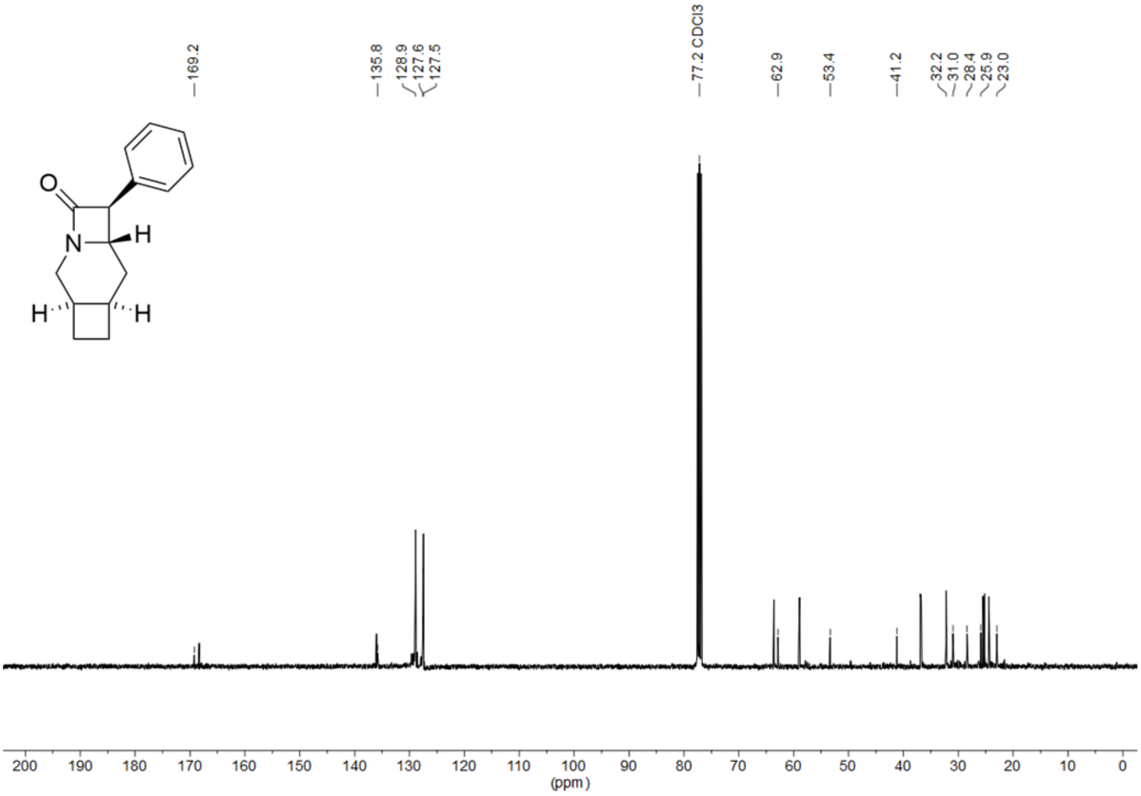

Figure S71: $^{13}C\{^{1}H\}$-NMR of **12b** (101 MHz, $CDCl_3$).

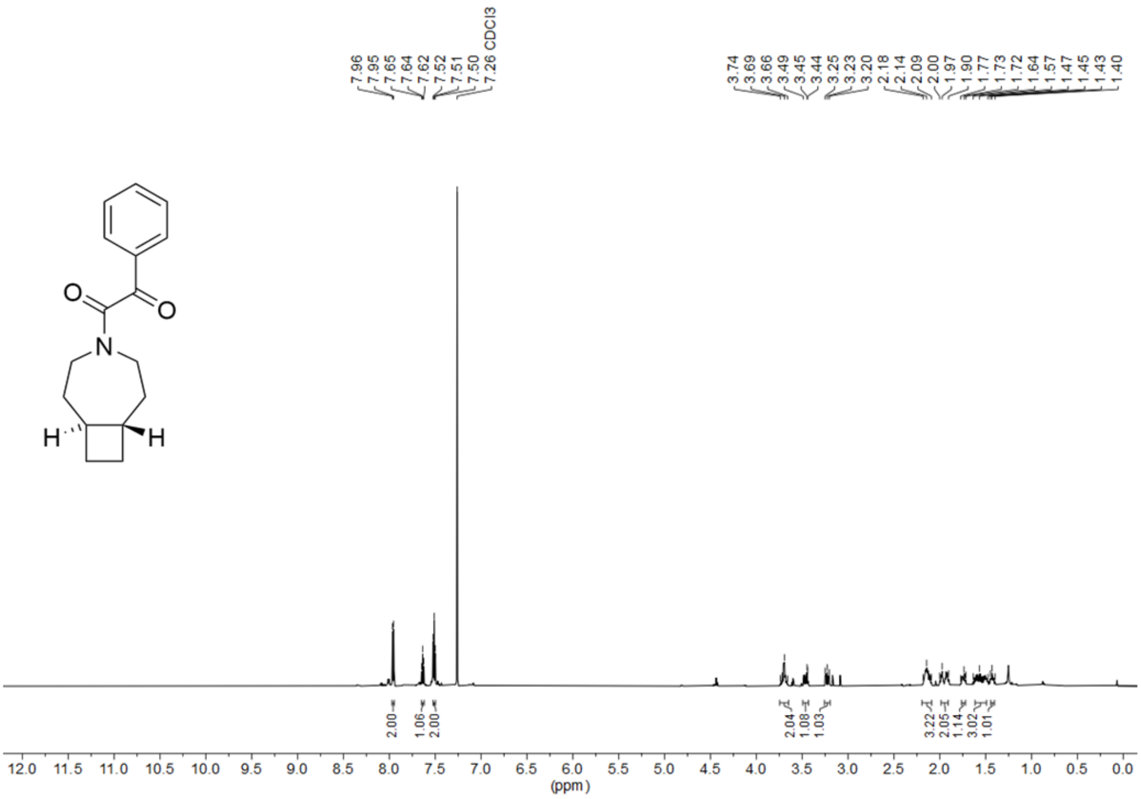

Figure S72: ^{1}H-NMR of **S5** (600 MHz, $CDCl_3$).

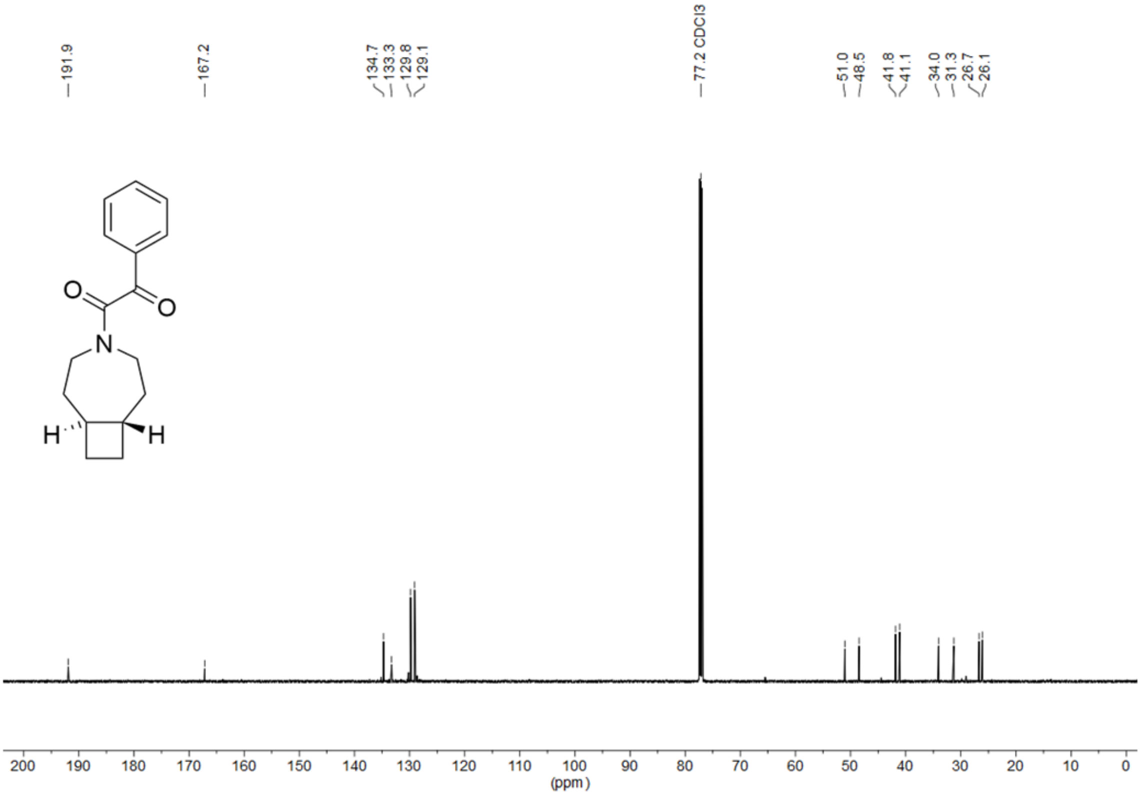

Figure S73: $^{13}C\{^{1}H\}$-NMR of **S5** (151 MHz, $CDCl_3$).

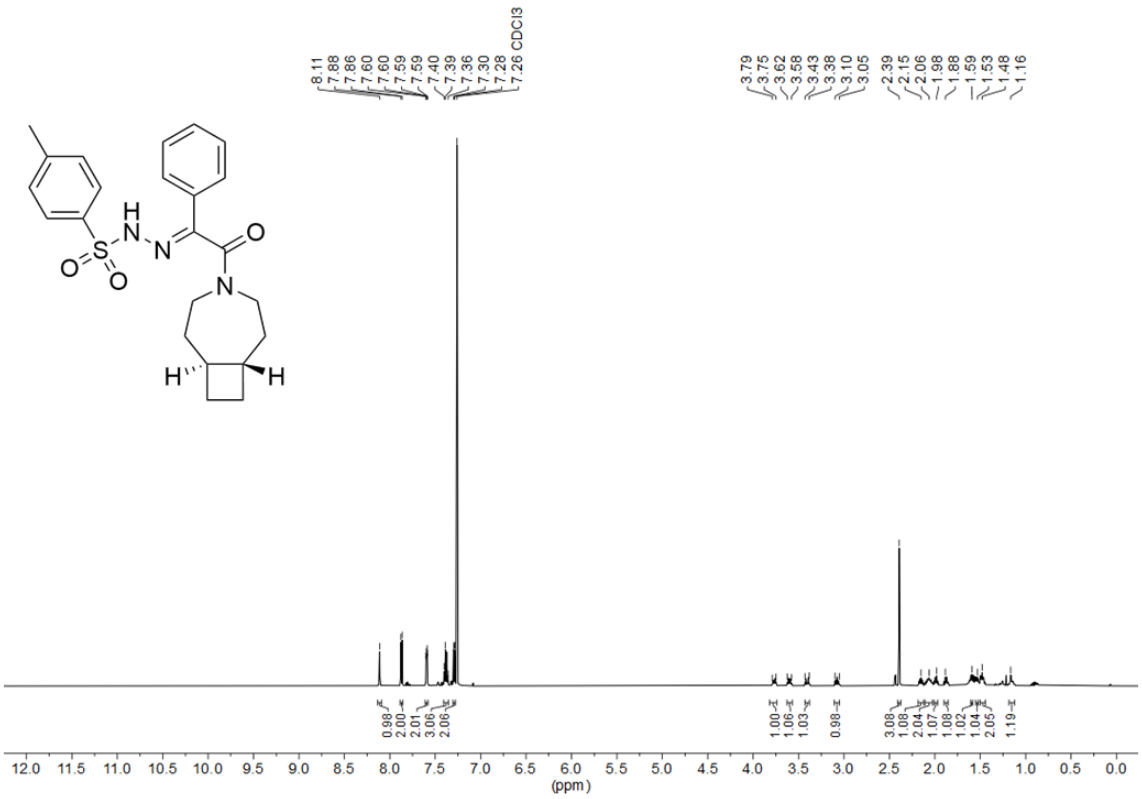

Figure S74: ^{1}H-NMR of **S6** (600 MHz, $CDCl_3$).

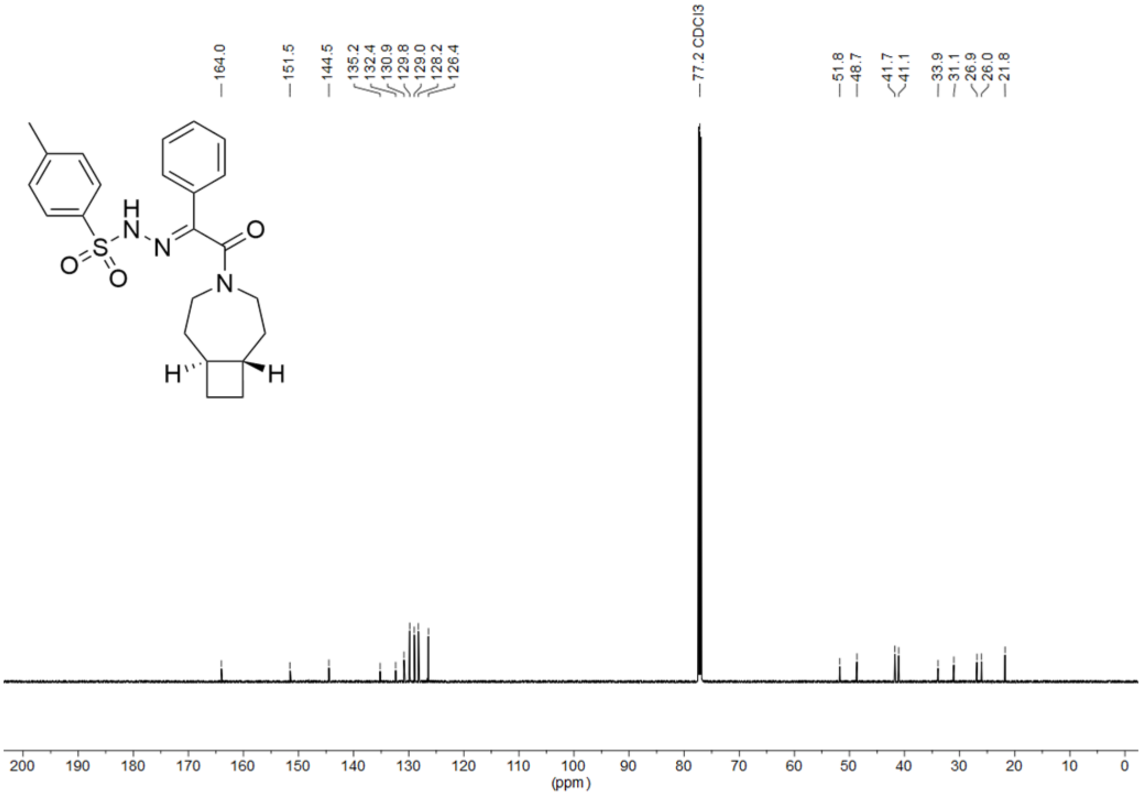

Figure S75: $^{13}C\{^{1}H\}$-NMR of **S6** (151 MHz, $CDCl_3$).

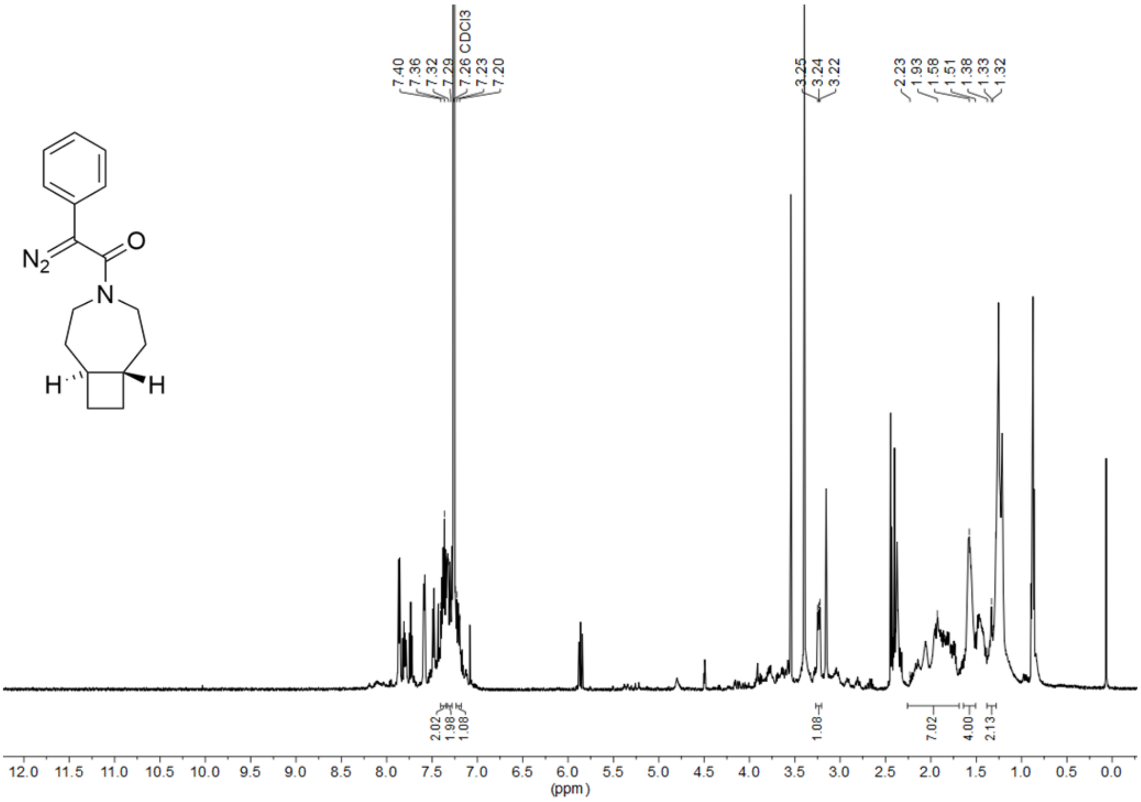

Figure S76: ^{1}H-NMR of **S7** (600 MHz, $CDCl_3$).

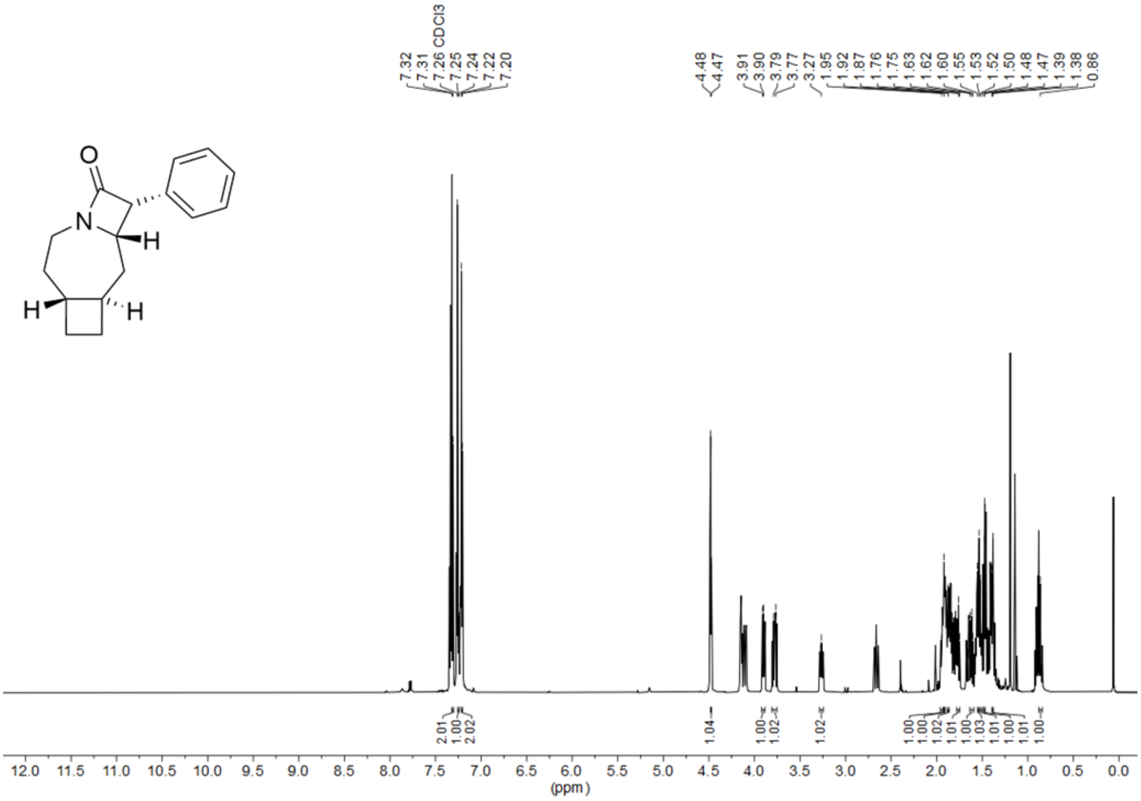

Figure S77: ^{1}H-NMR of **13a** (600 MHz, $CDCl_3$).

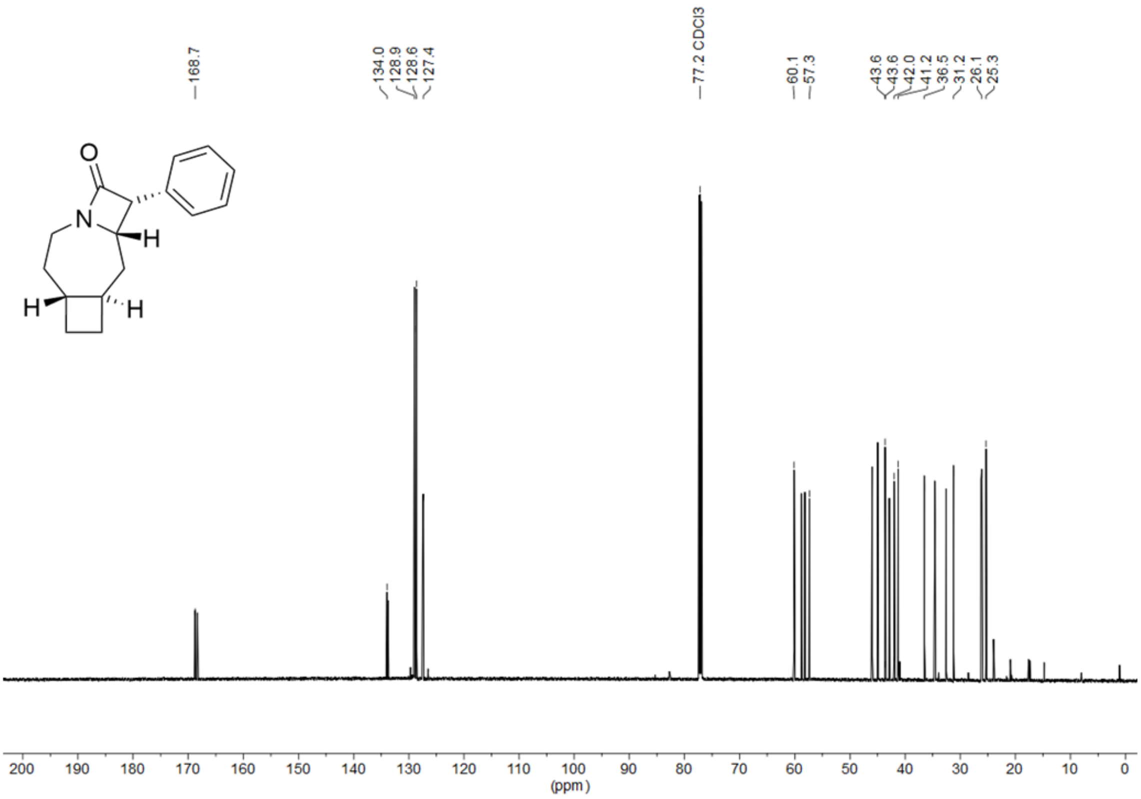

Figure S78: ${}^{13}C\{{}^{1}H\}$-NMR of **13a** (151 MHz, $CDCl_3$).

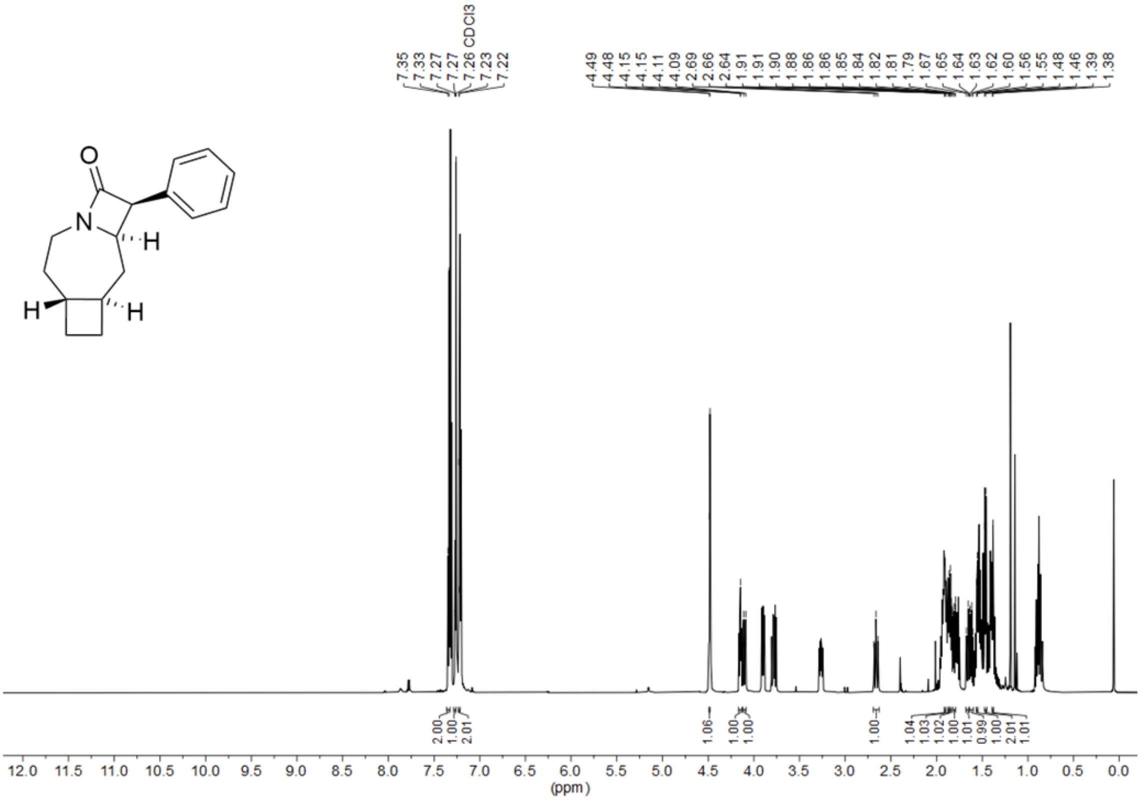

Figure S79: ${}^{1}H$-NMR of **13b** (600 MHz, $CDCl_3$).

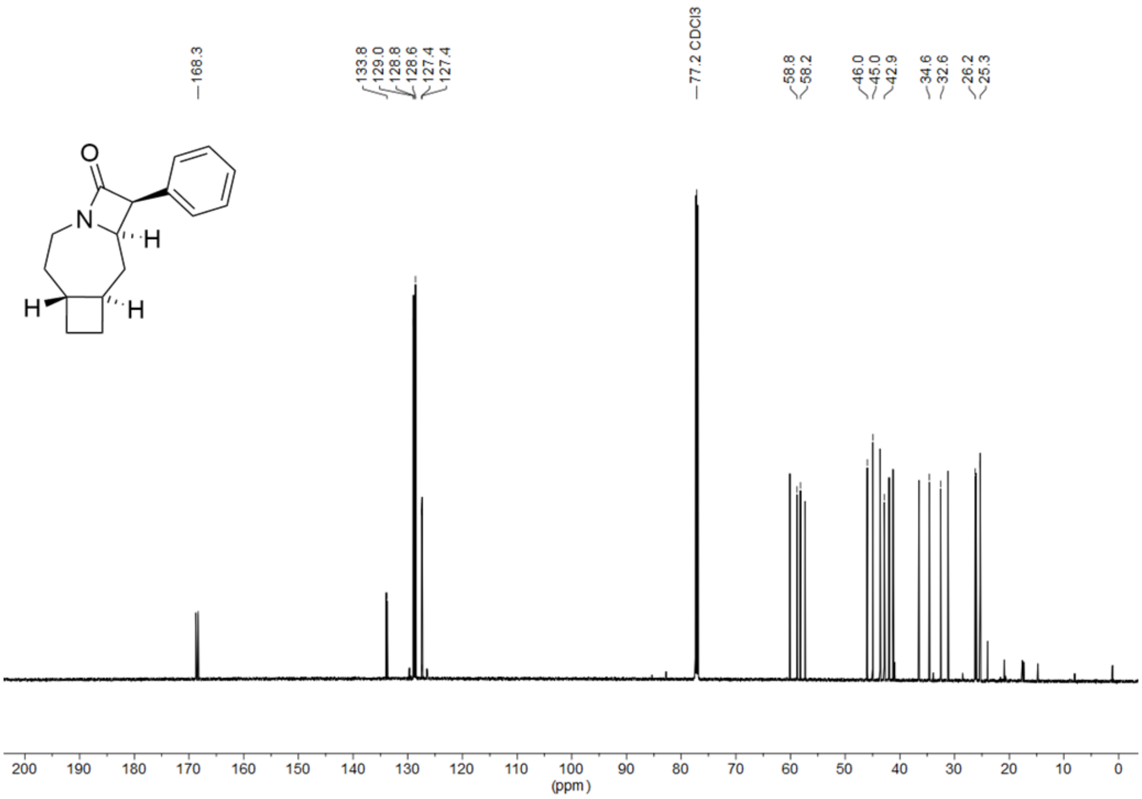

Figure S80: $^{13}C\{^{1}H\}$-NMR of **13b** (151 MHz, $CDCl_3$).

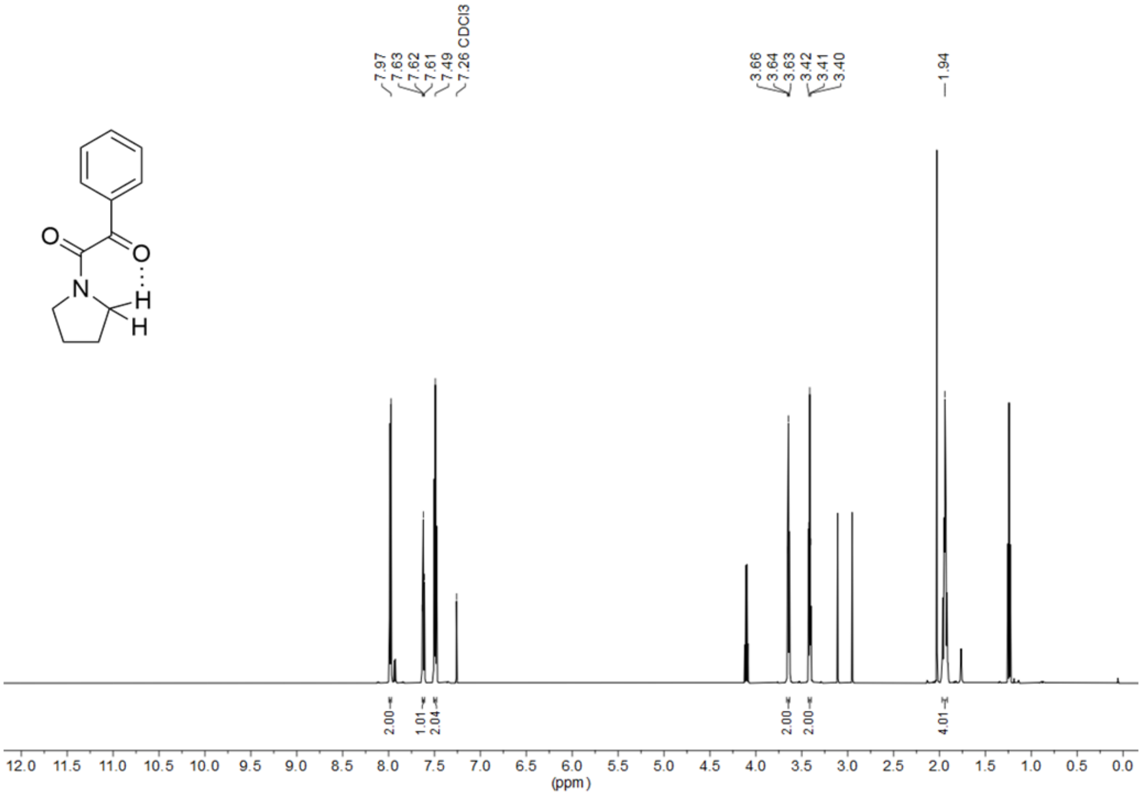

Figure S81: ^{1}H-NMR of **S8** (600 MHz, $CDCl_3$).

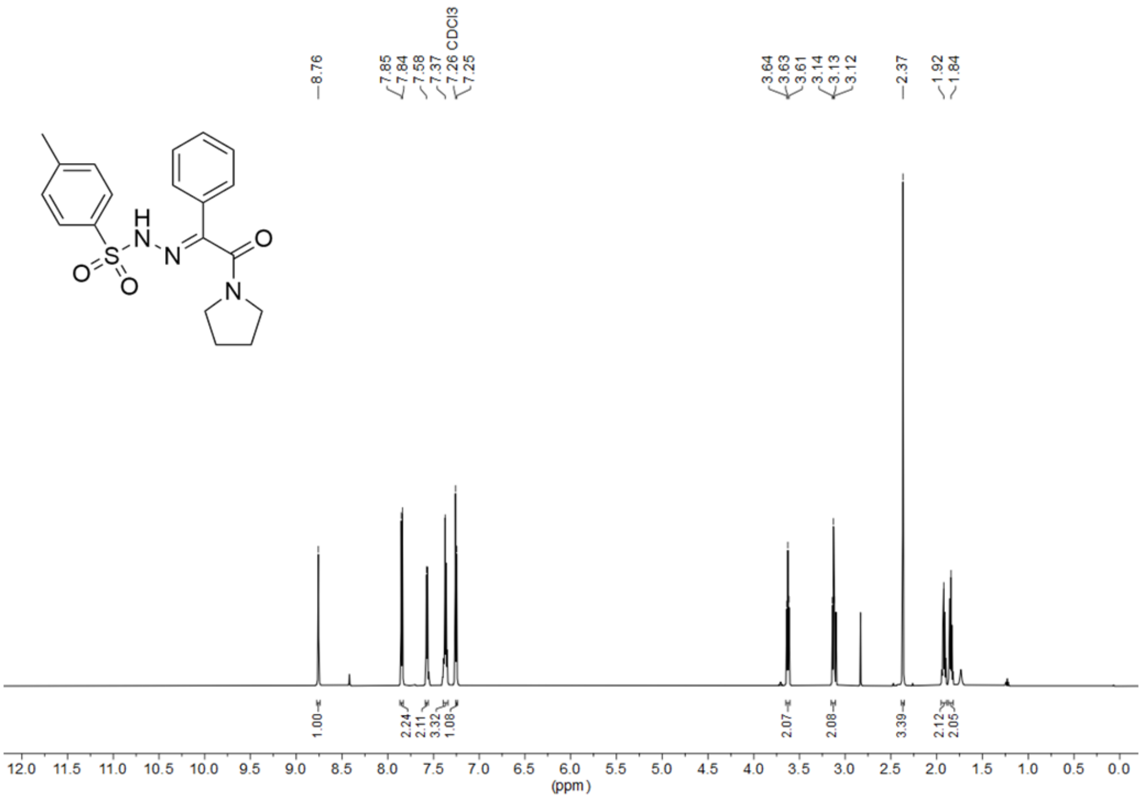

Figure S82: ^{1}H-NMR of **S9** (600 MHz, $CDCl_3$).

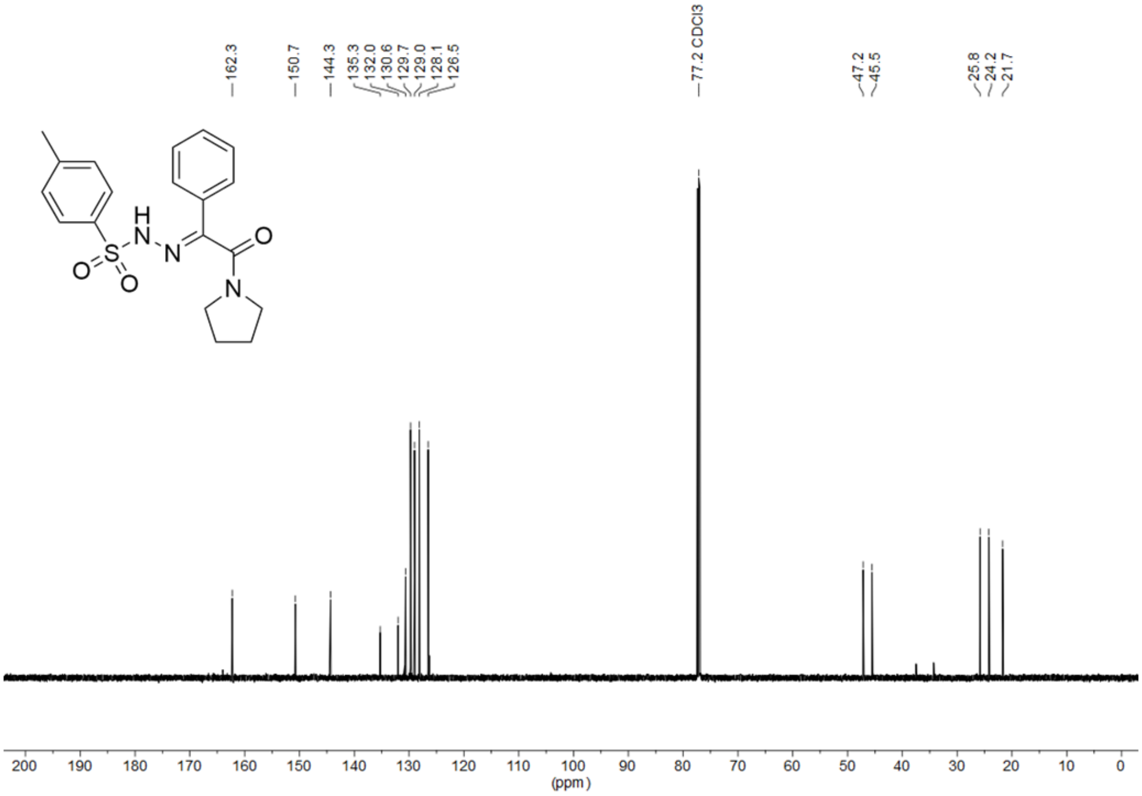

Figure S83: ^{13}C{^{1}H}-NMR of **S9** (151 MHz, $CDCl_3$).

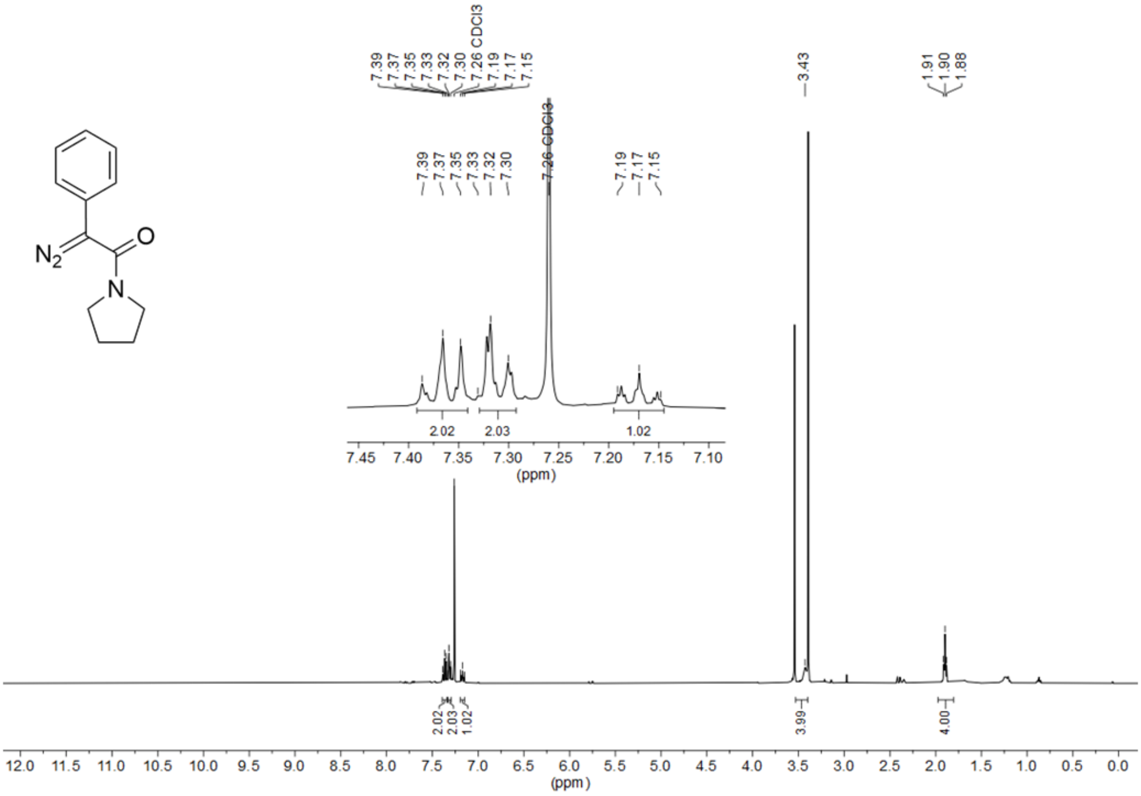

Figure S84: ^{1}H-NMR of **S10** (400 MHz, $CDCl_3$).

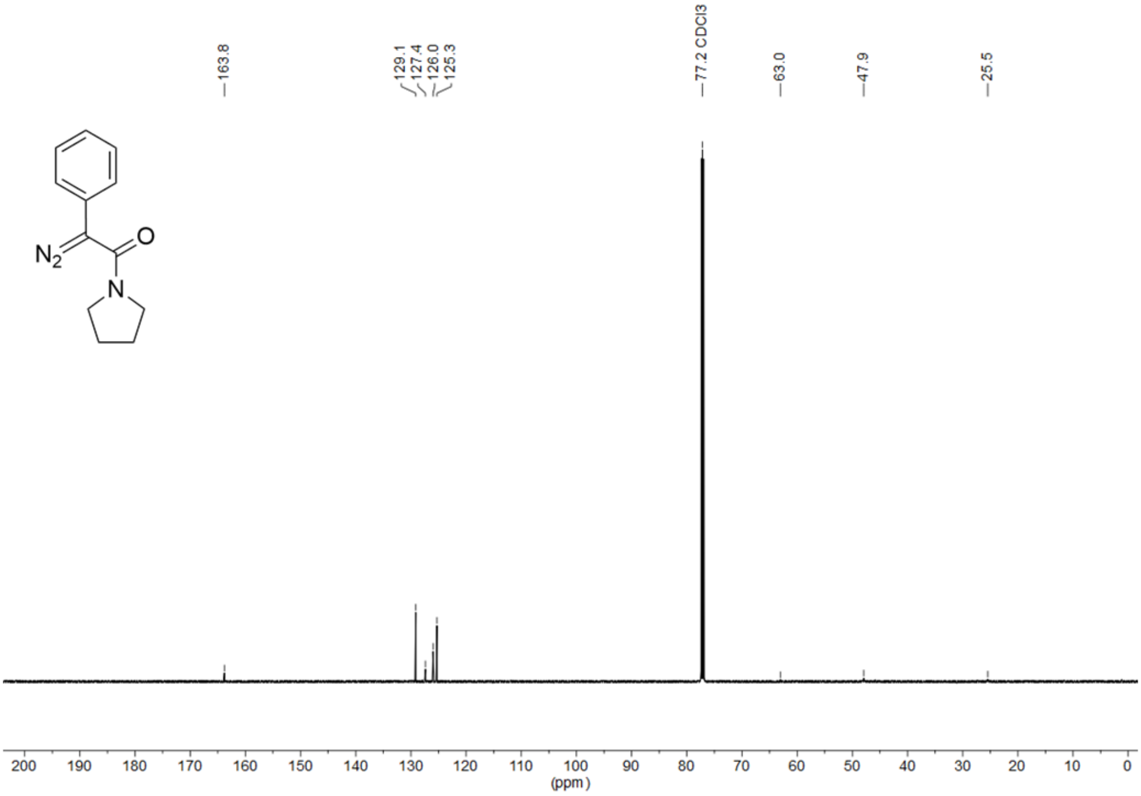

Figure S85: $^{13}C\{^{1}H\}$-NMR of **S10** (151 MHz, $CDCl_3$).

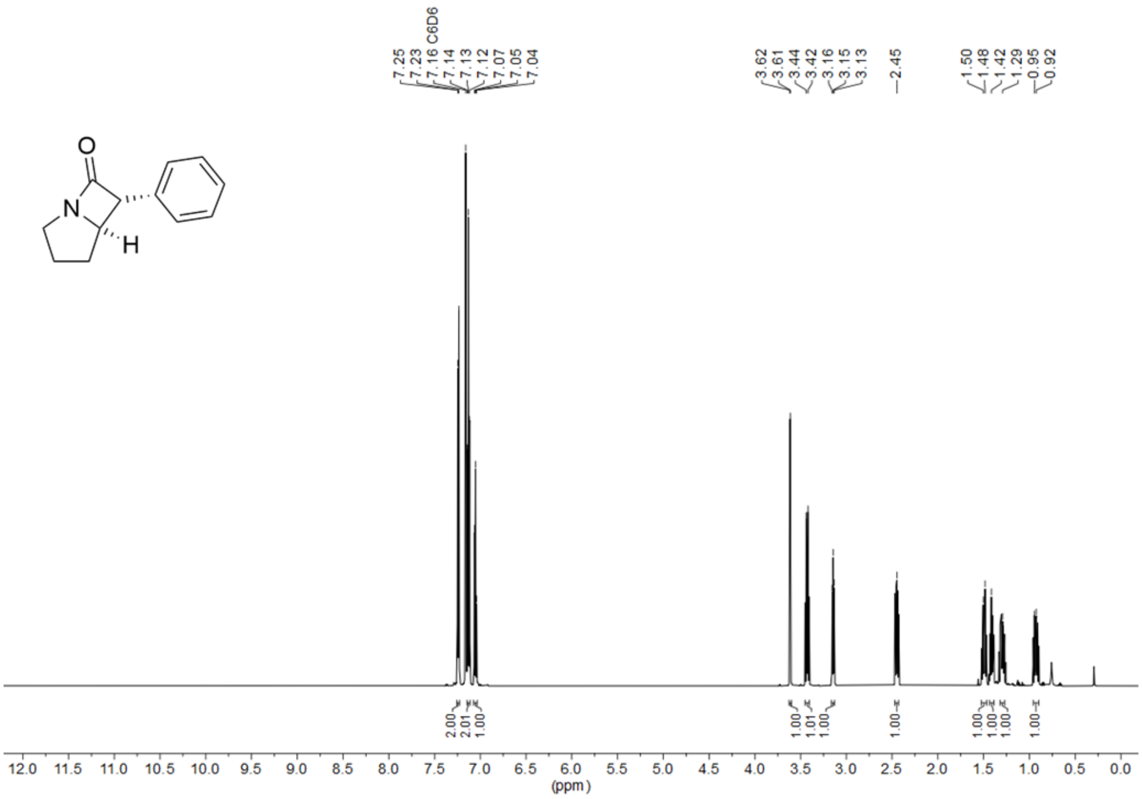

Figure S86: ^{1}H-NMR of **14** (400 MHz, C_6D_6).

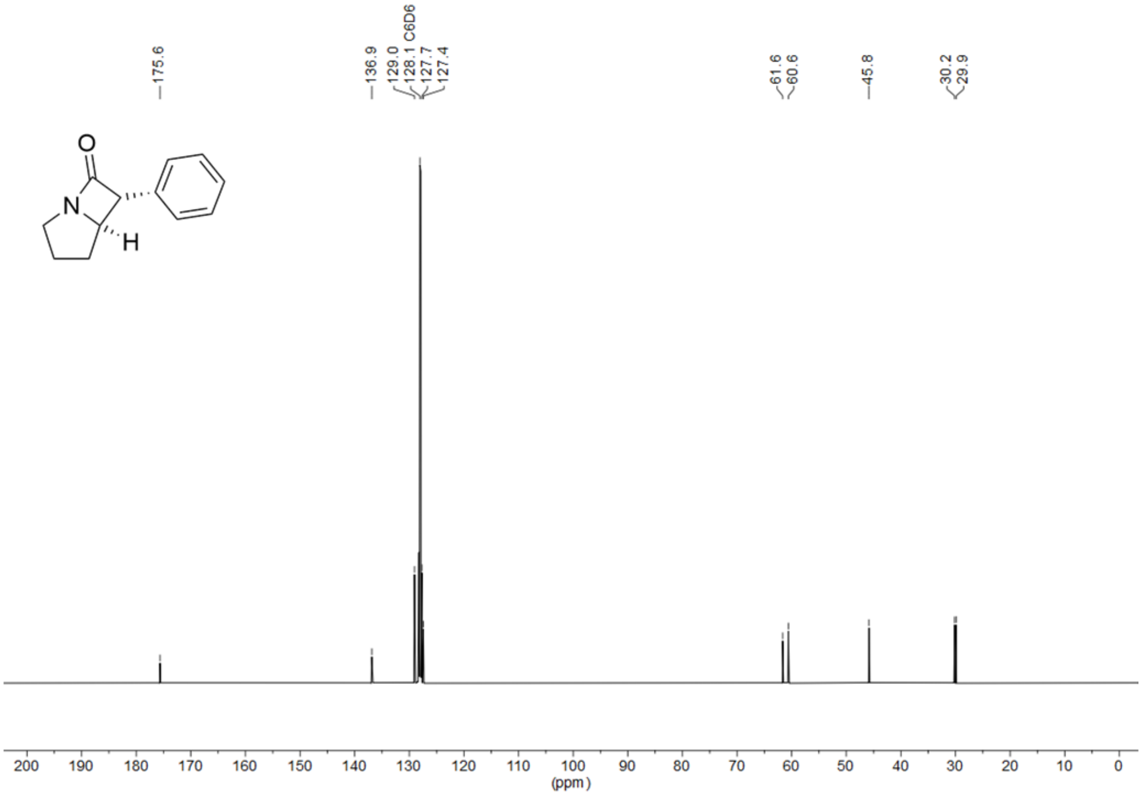

Figure S87: ^{13}C{^{1}H}-NMR of **14** (151 MHz, C_6D_6).

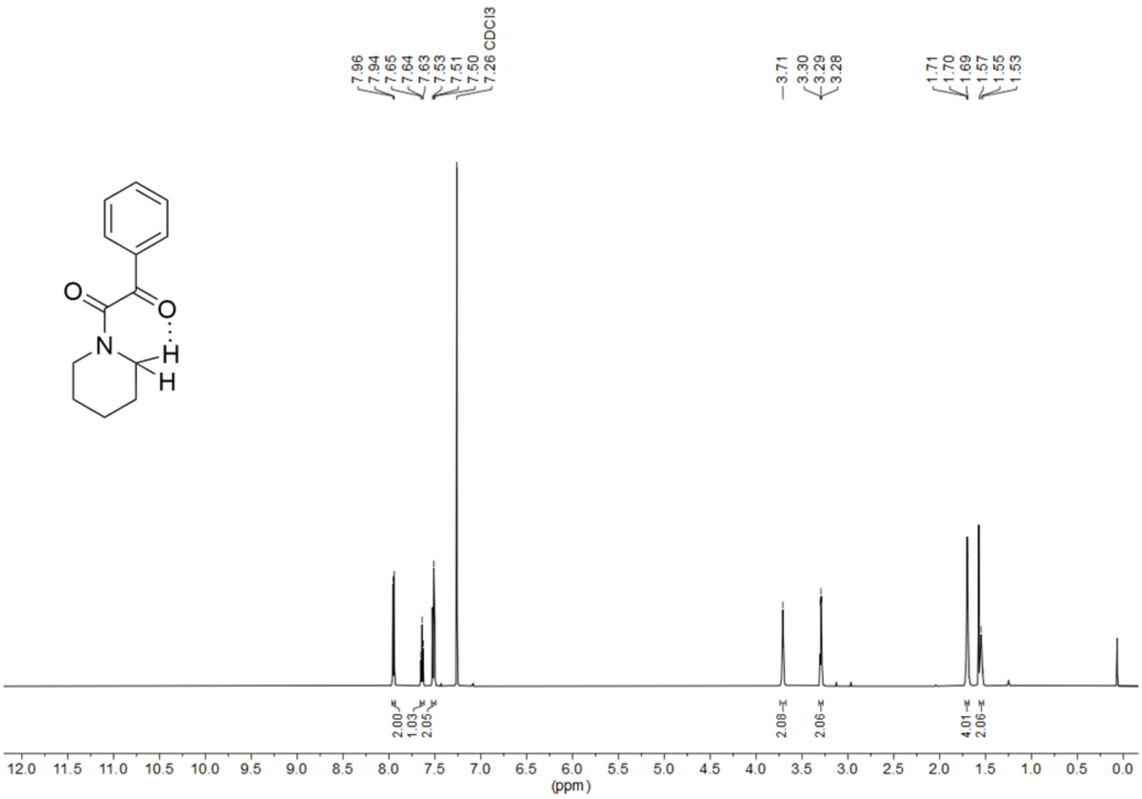

Figure S88: ^{1}H-NMR of **S11** (600 MHz, $CDCl_3$).

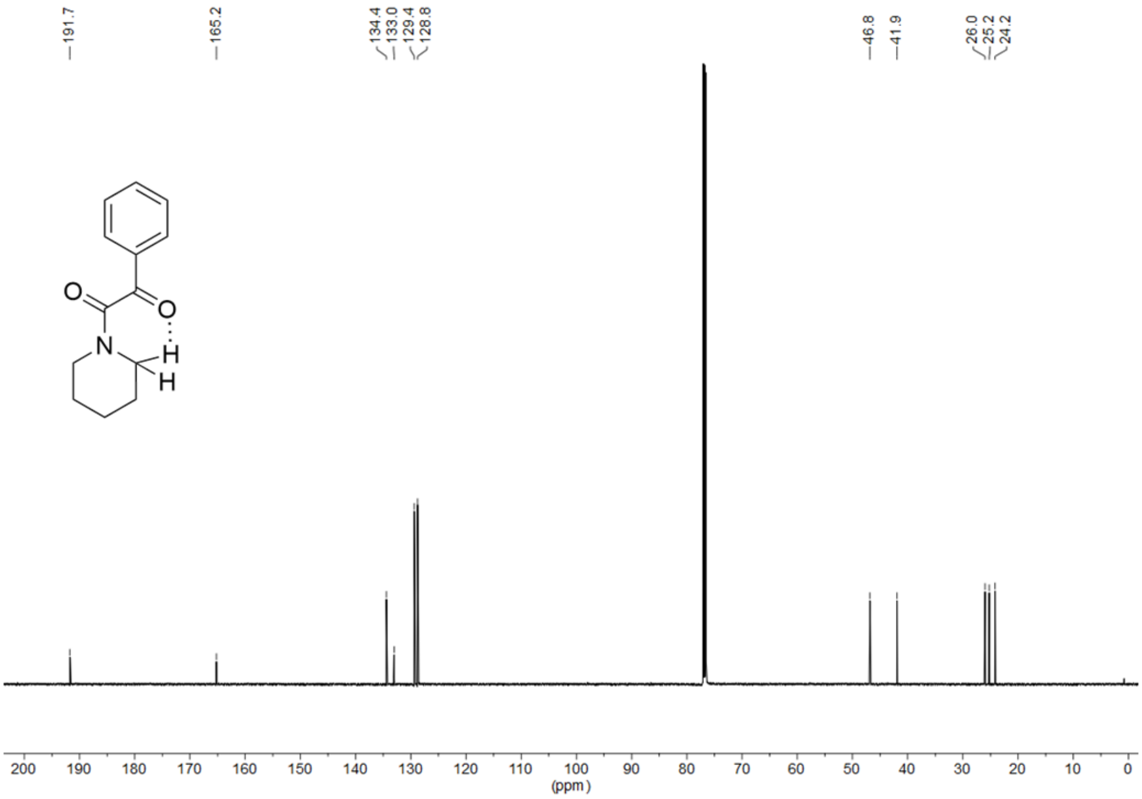

Figure S89: $^{13}C\{^{1}H\}$-NMR of **S11** (151 MHz, $CDCl_3$).

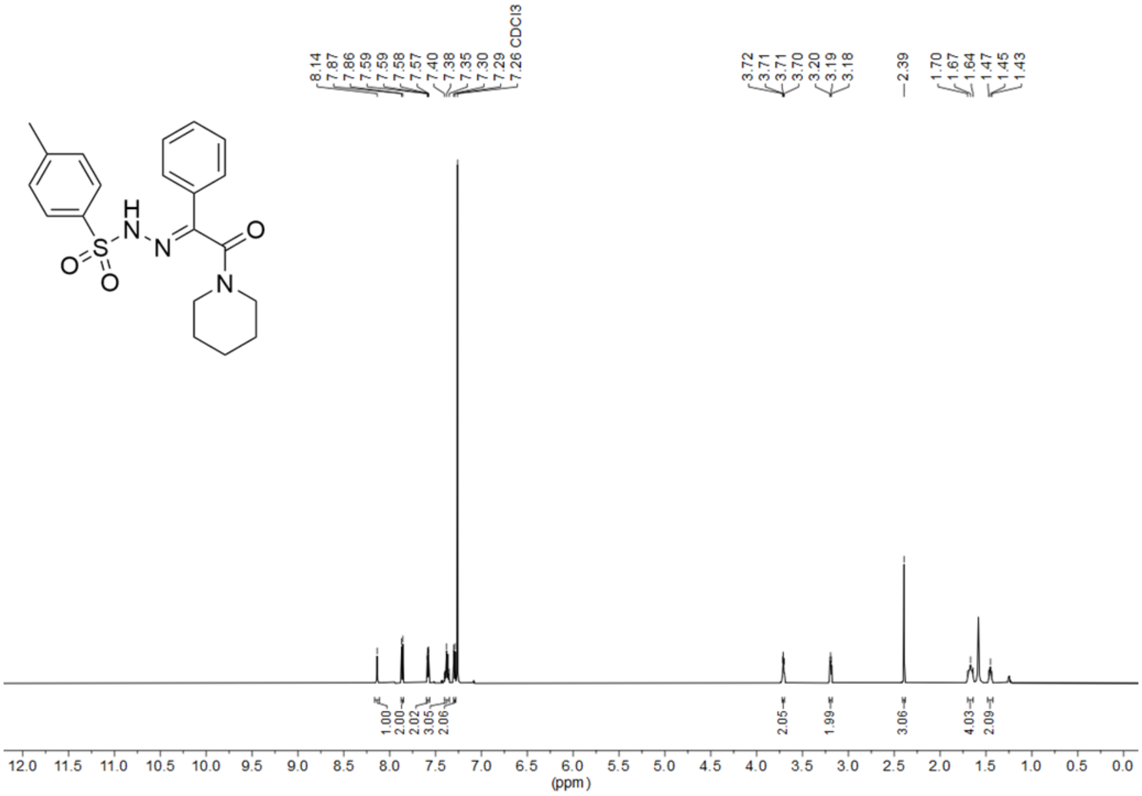

Figure S90: ^{1}H-NMR of **S12** (600 MHz, $CDCl_3$).

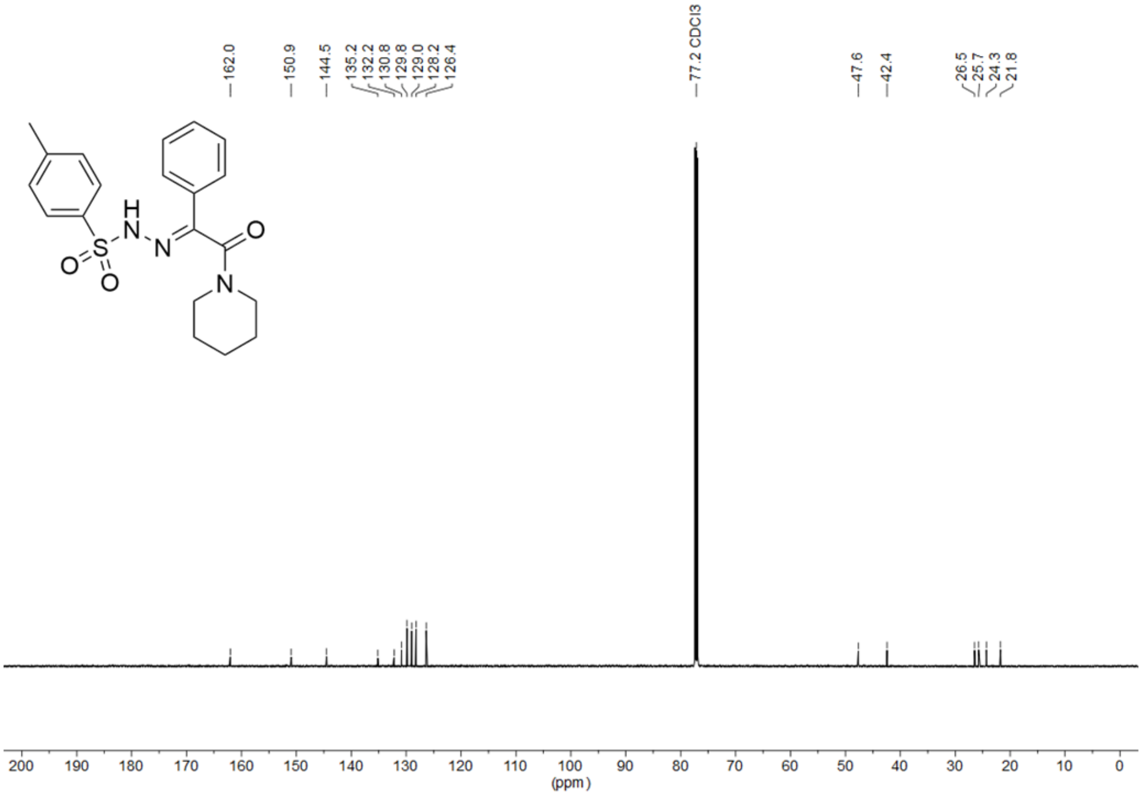

Figure S91: ^{13}C{^{1}H}-NMR of **S12** (151 MHz, $CDCl_3$).

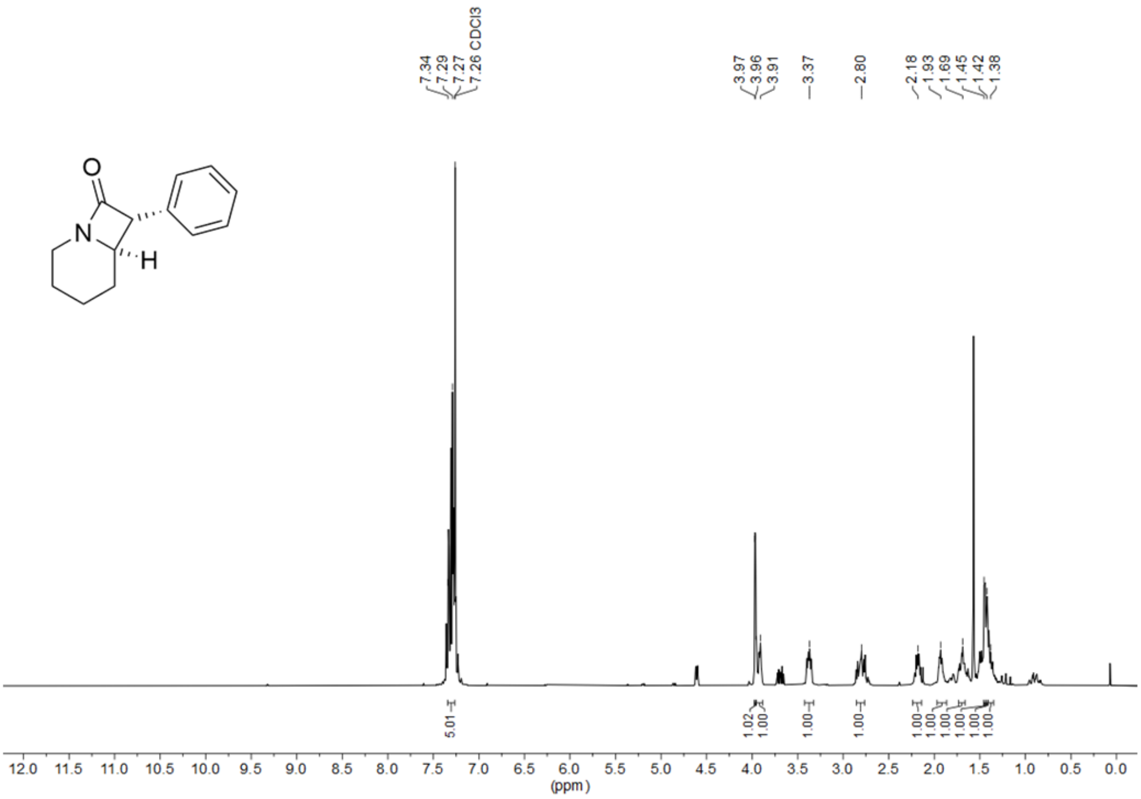

Figure S92: ^{1}H-NMR of **15a** (300 MHz, $CDCl_3$).

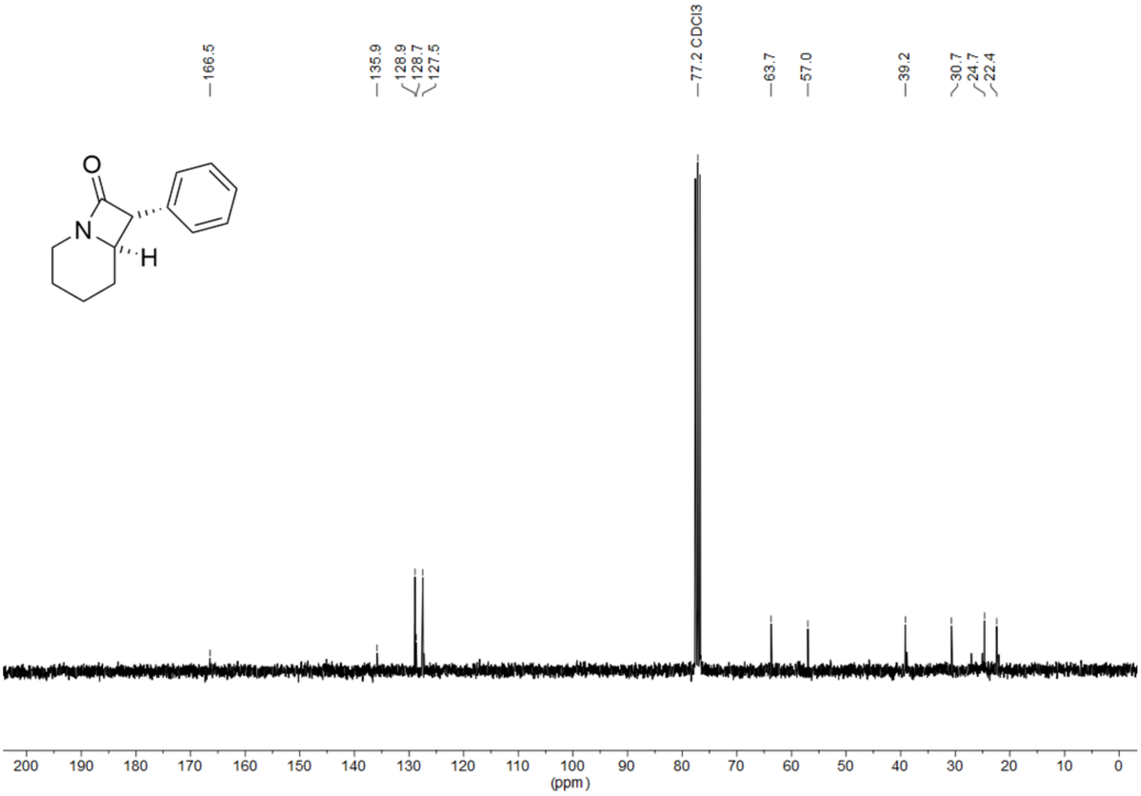

Figure S93: $^{13}C\{^{1}H\}$-NMR of **15a** (75 MHz, $CDCl_3$).

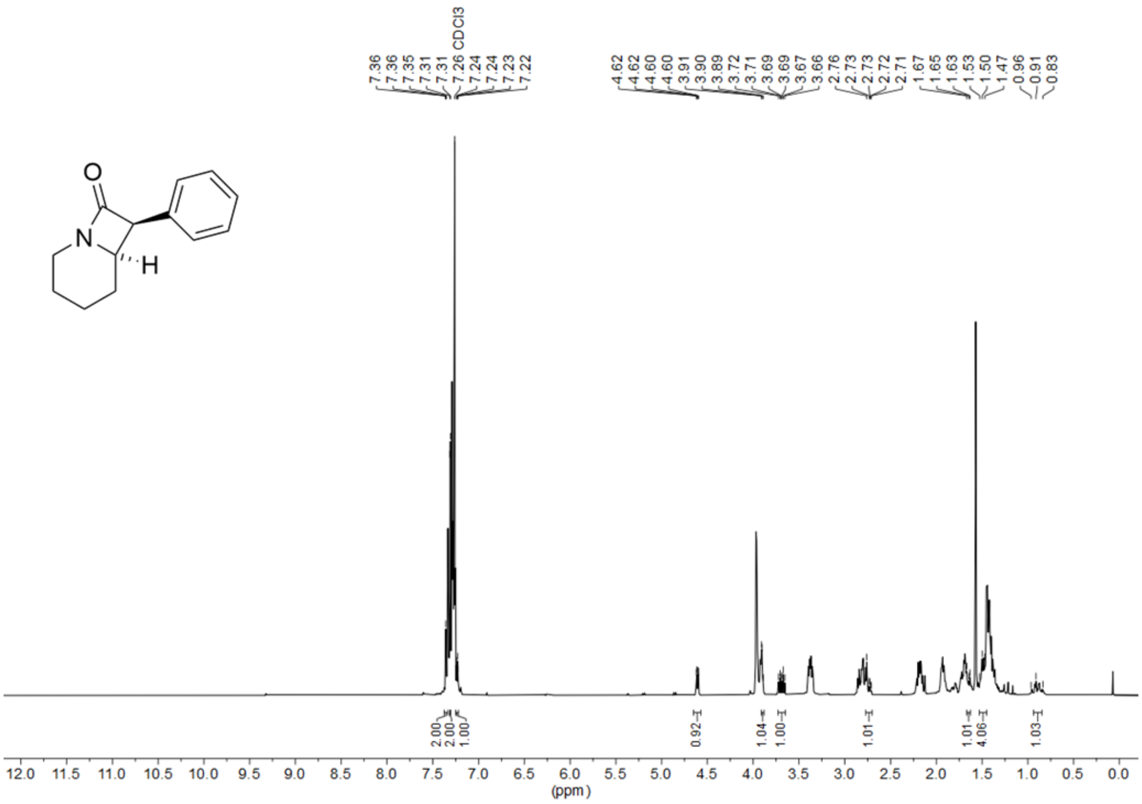

Figure S94: ^{1}H-NMR of **15b** (300 MHz, $CDCl_3$).

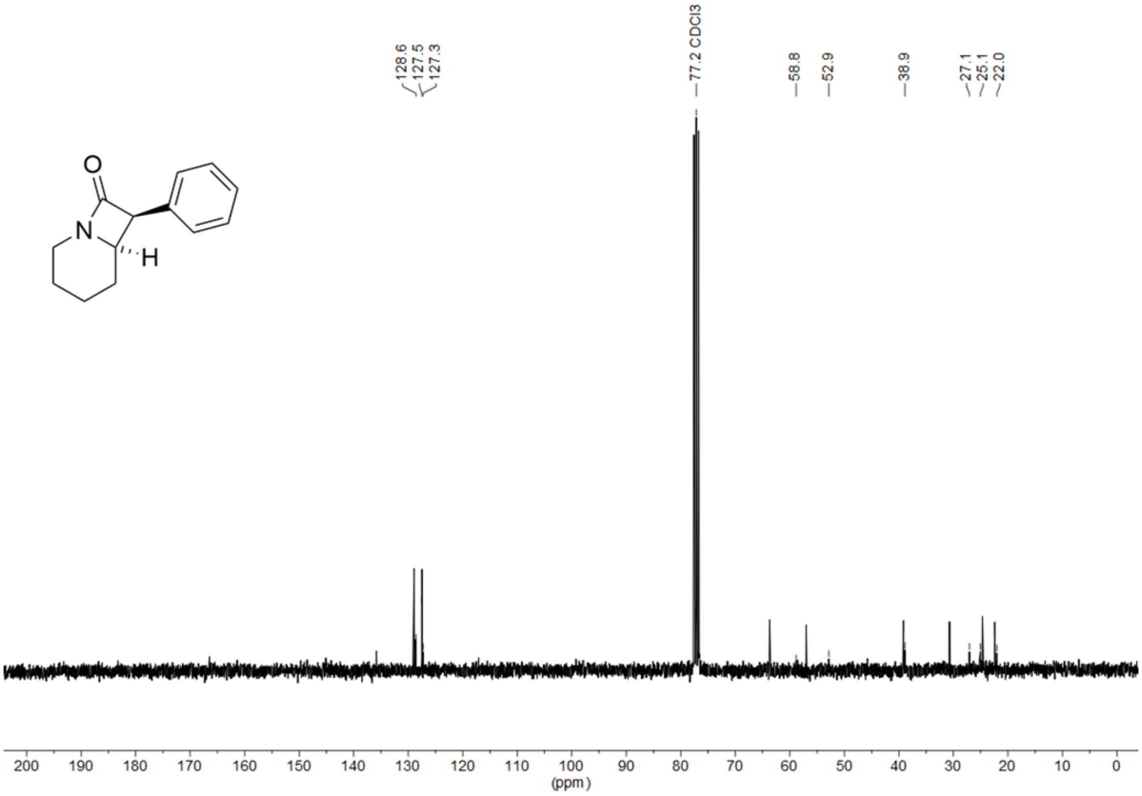

Figure S95: $^{13}C\{^{1}H\}$-NMR of **15b** (75 MHz, $CDCl_3$).

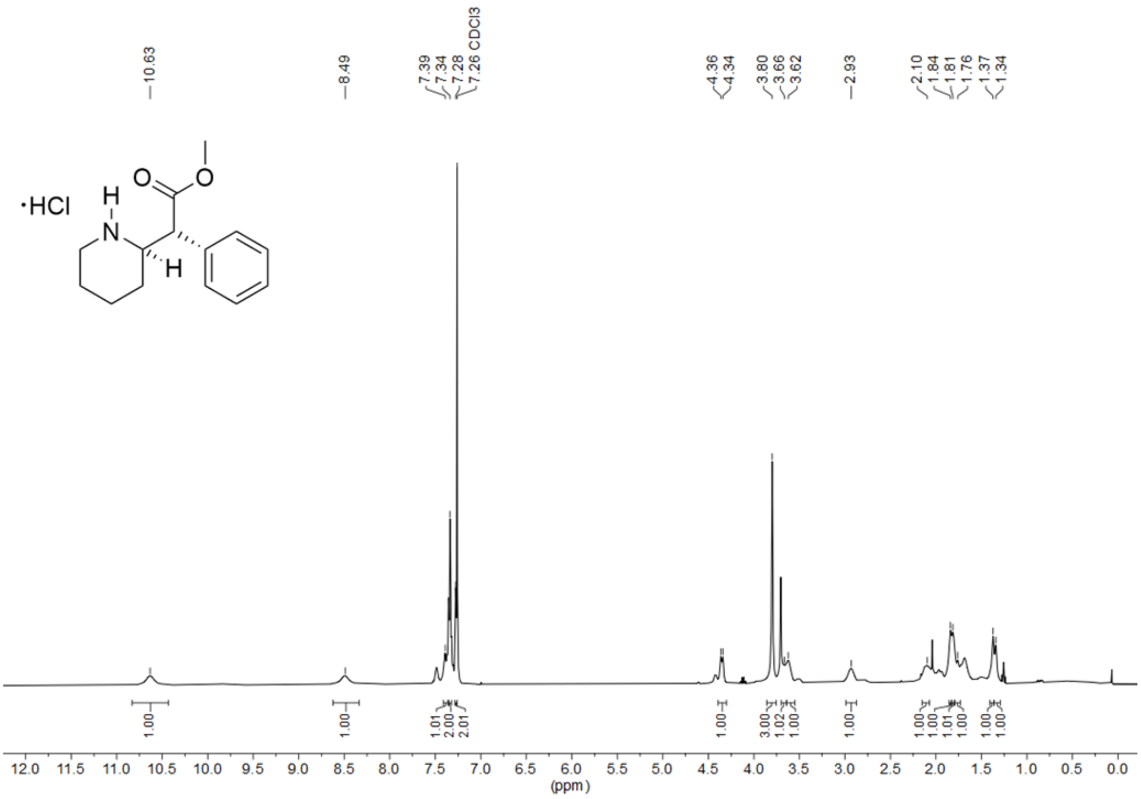

Figure S96: ^{1}H-NMR of **22** (400 MHz, $CDCl_3$).

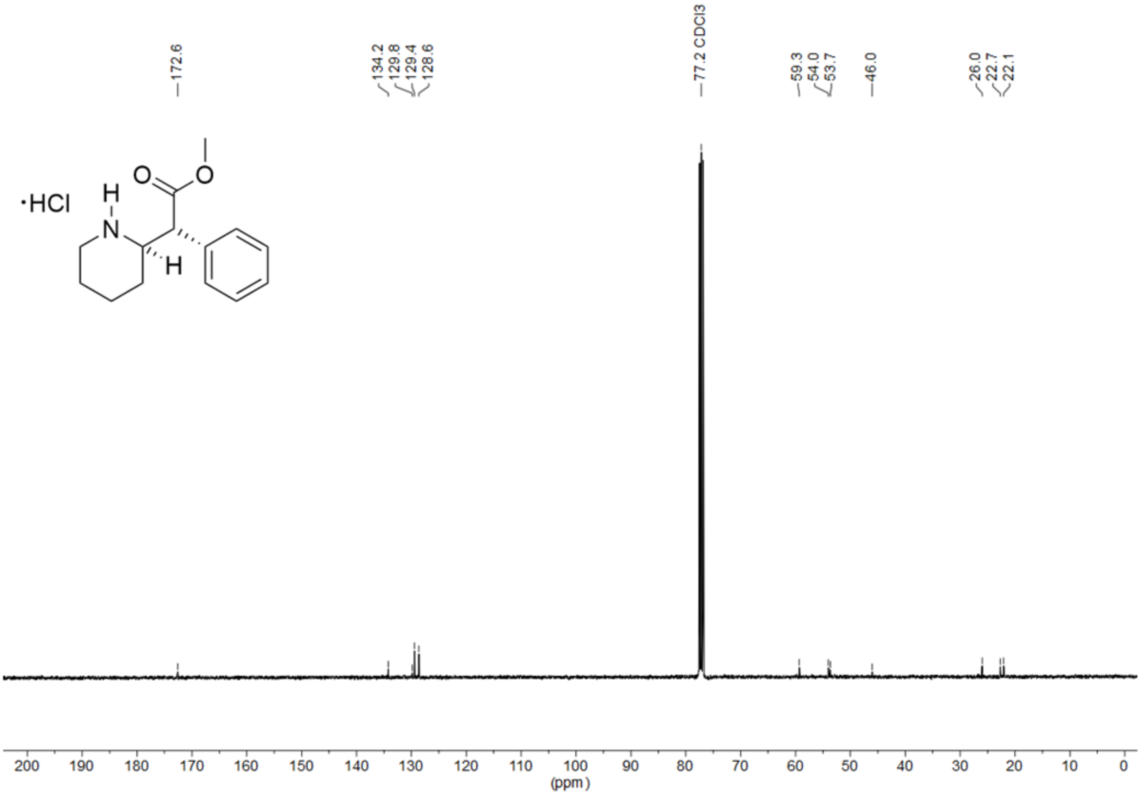

Figure S97: ^{13}C{^{1}H}-NMR of **22** (101 MHz, $CDCl_3$).

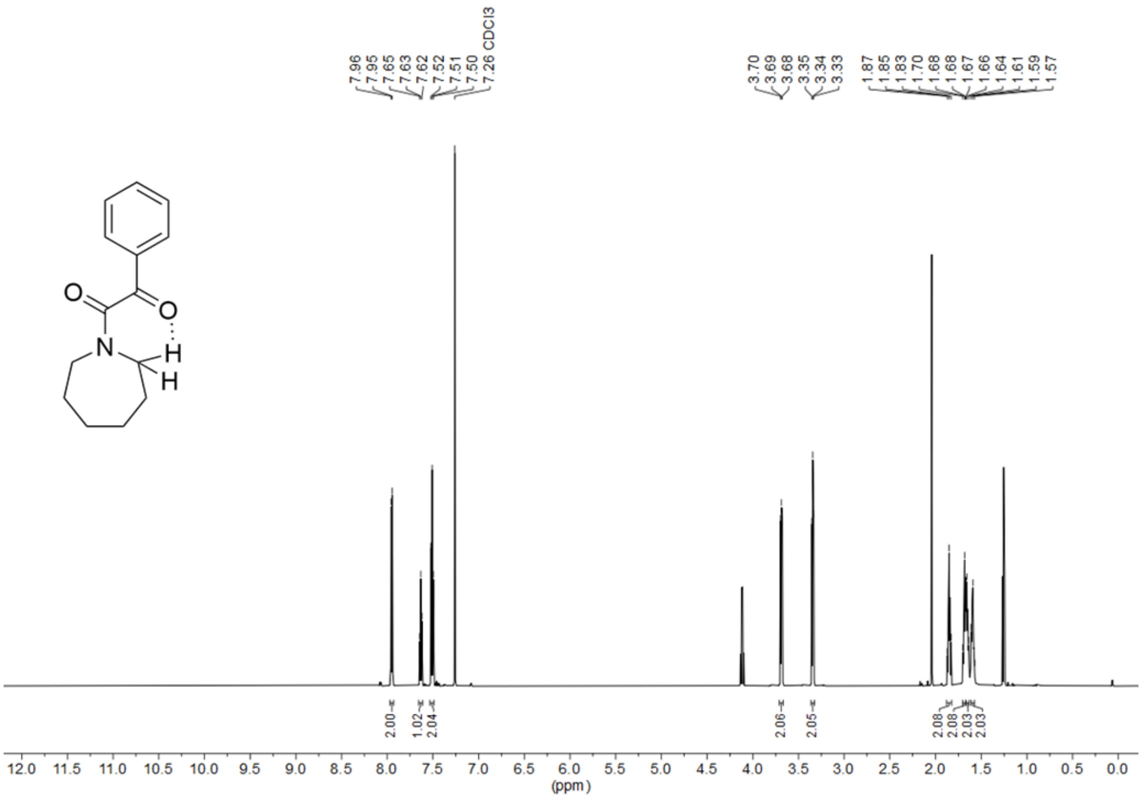

Figure S98: ^{1}H-NMR of **S13** (600 MHz, $CDCl_3$).

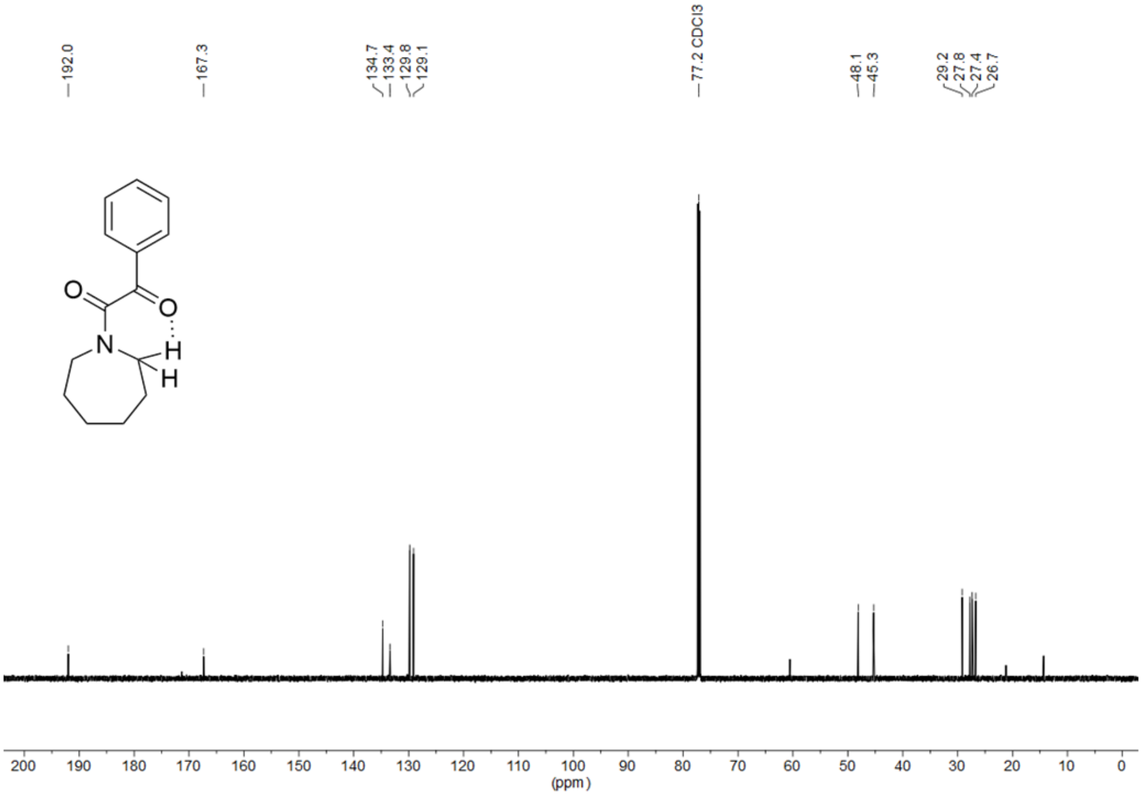

Figure S99: $^{13}C\{^{1}H\}$-NMR of **S13** (151 MHz, $CDCl_3$).

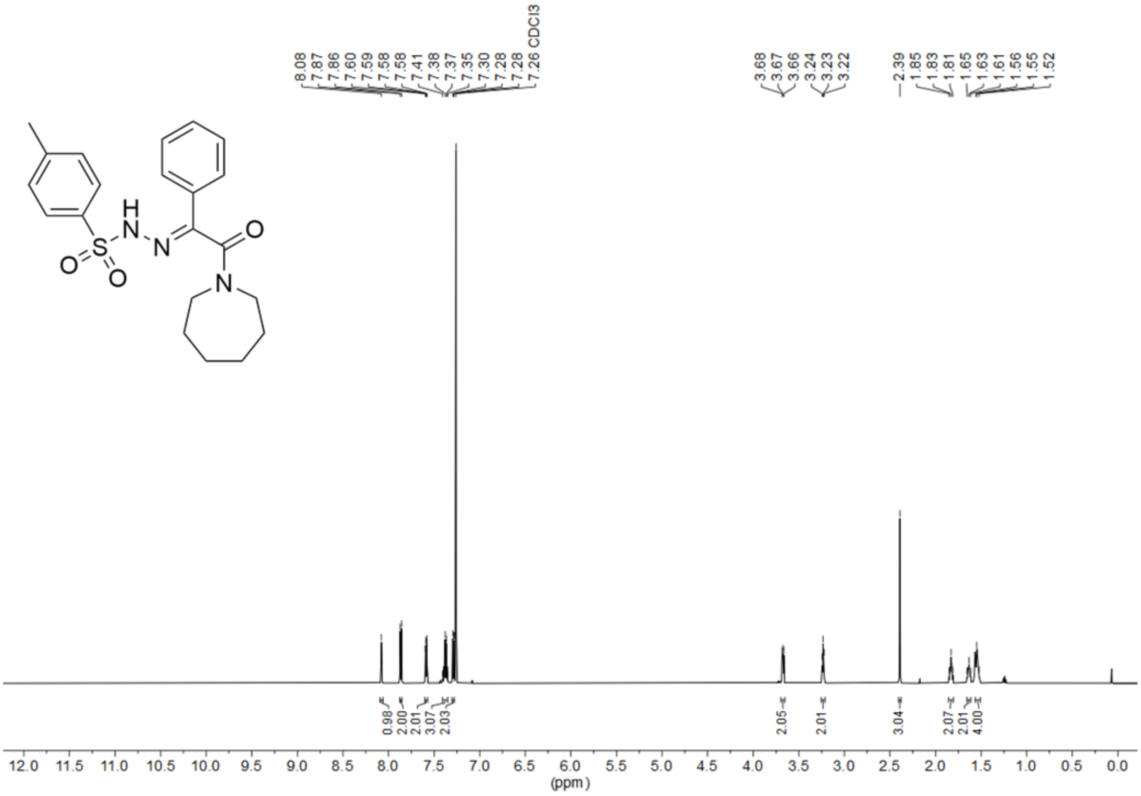

Figure S100: ^{1}H-NMR of **S14** (600 MHz, $CDCl_3$).

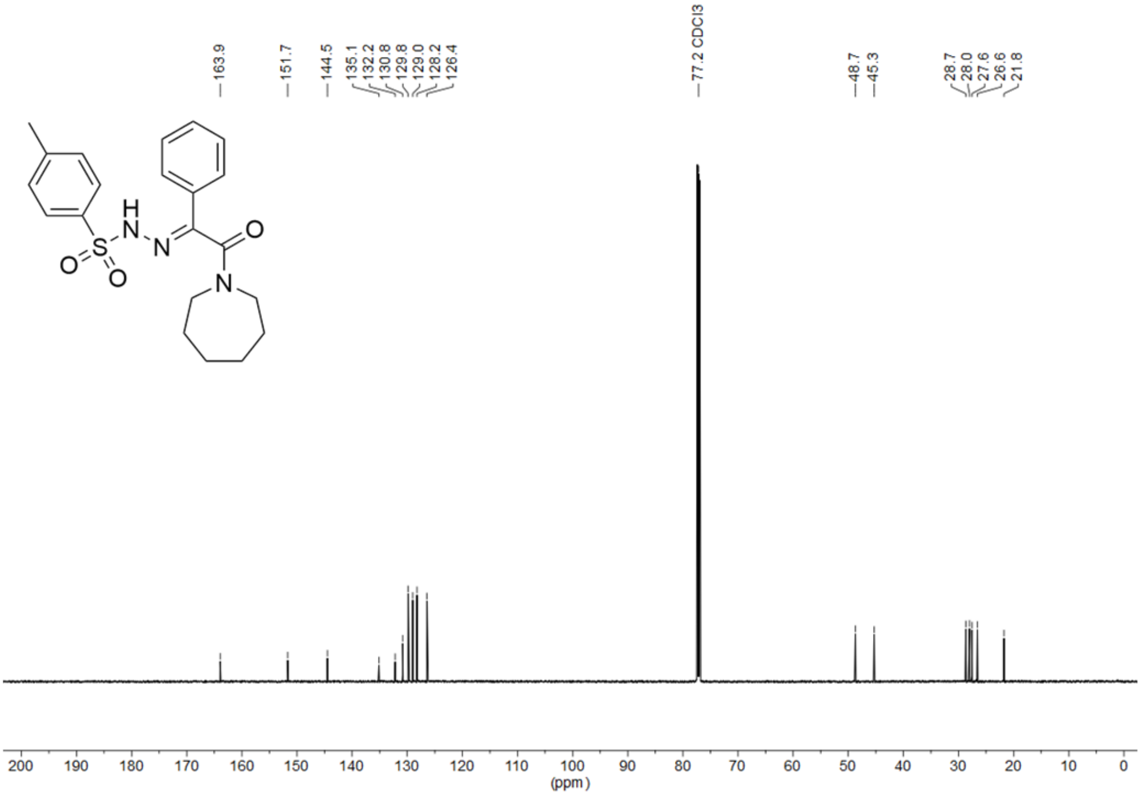

Figure S101: $^{13}C\{^{1}H\}$-NMR of **S14** (151 MHz, $CDCl_3$).

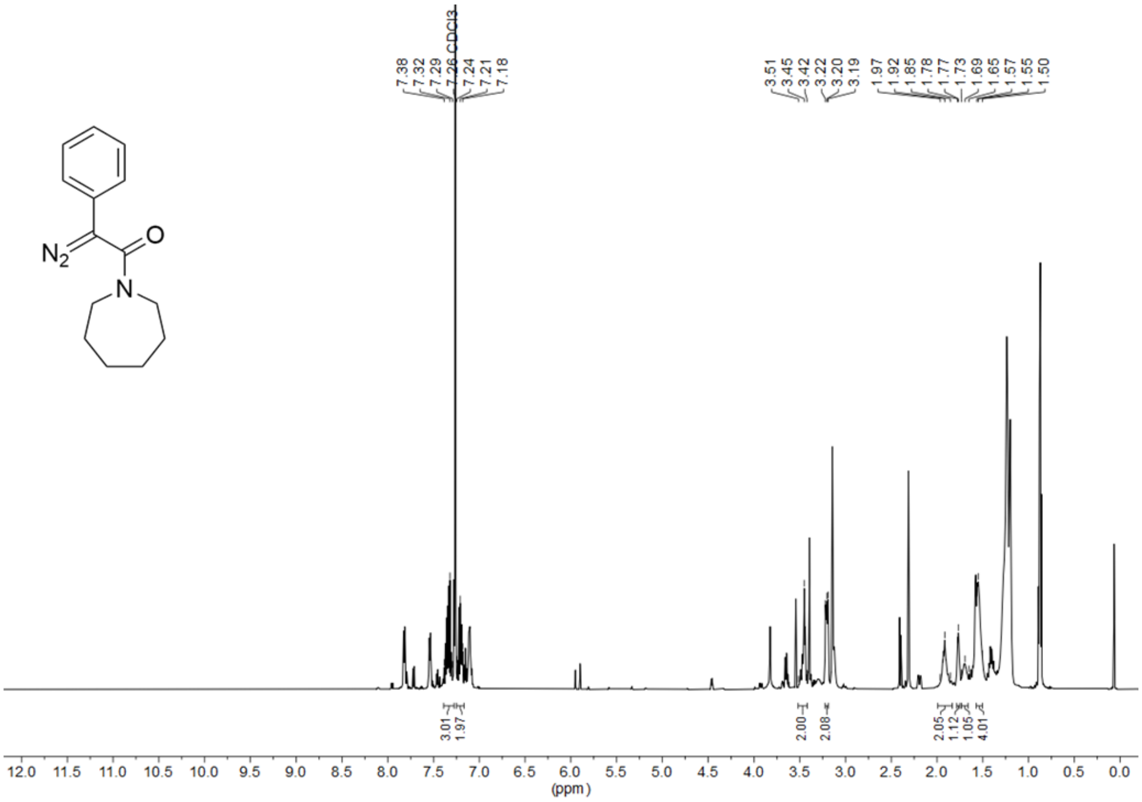

Figure S102: ^{1}H-NMR of **S15** (600 MHz, $CDCl_3$).

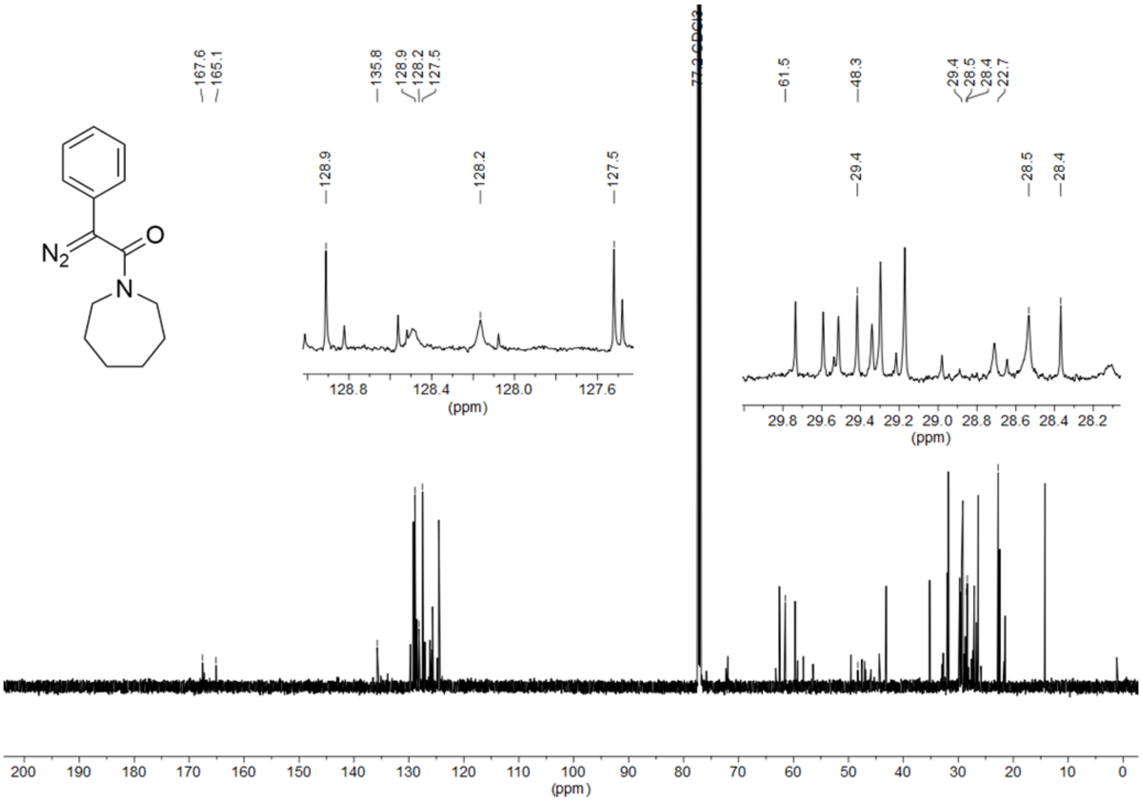

Figure S103: $^{13}C\{^1H\}$-NMR of **S15** (151 MHz, $CDCl_3$).

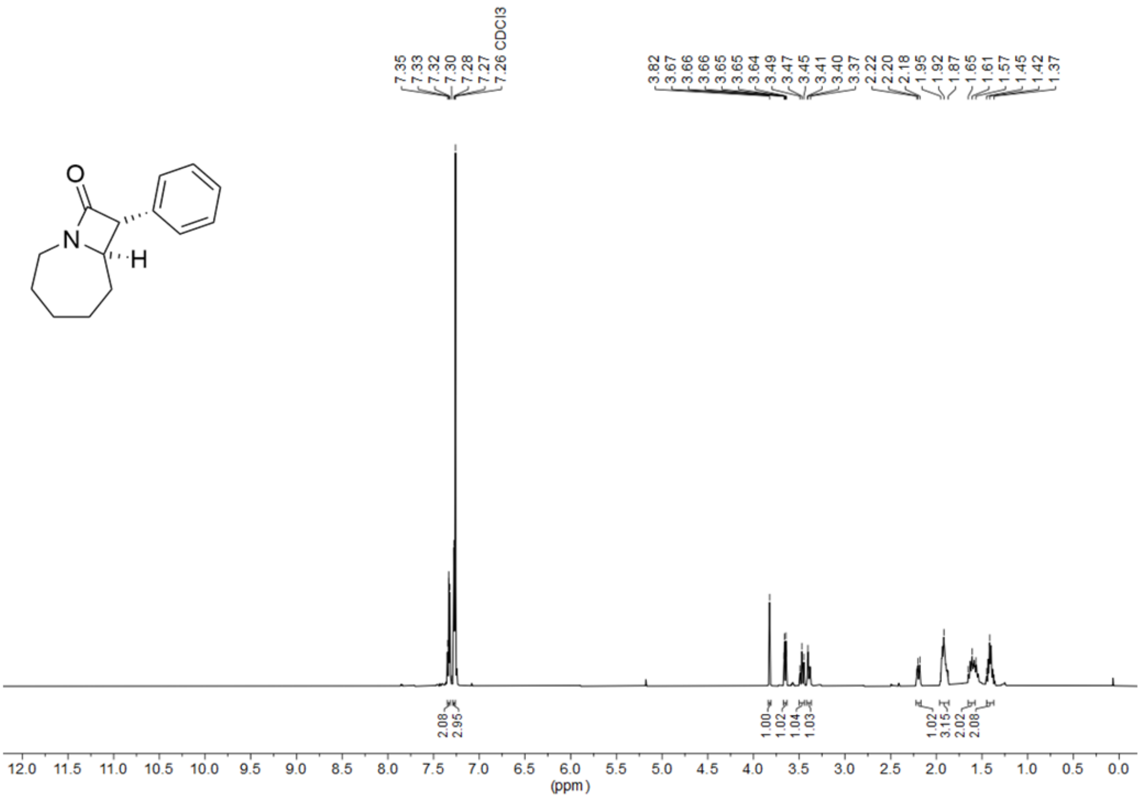

Figure S104: ^{1}H-NMR of **16a** (600 MHz, $CDCl_3$). Since the compound is prone to decomposition, the spectrum contains impurities.

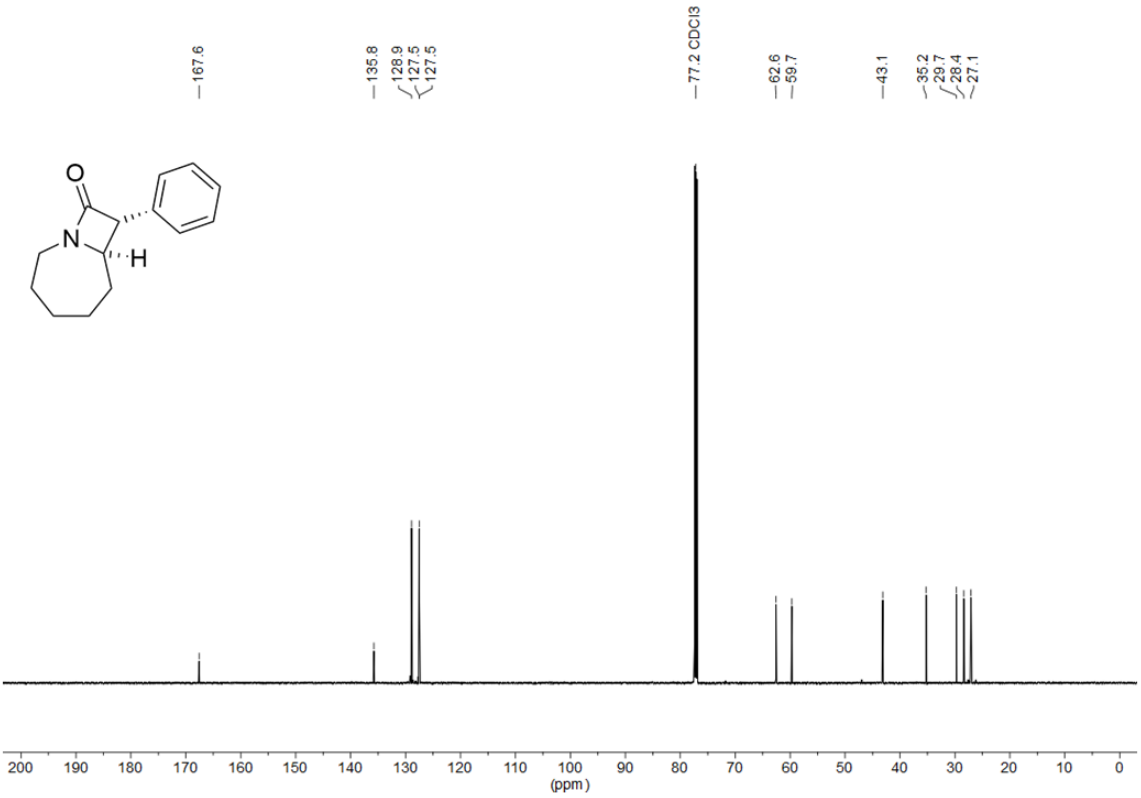

Figure S105: $^{13}C\{^{1}H\}$-NMR of **16a** (151 MHz, $CDCl_3$).

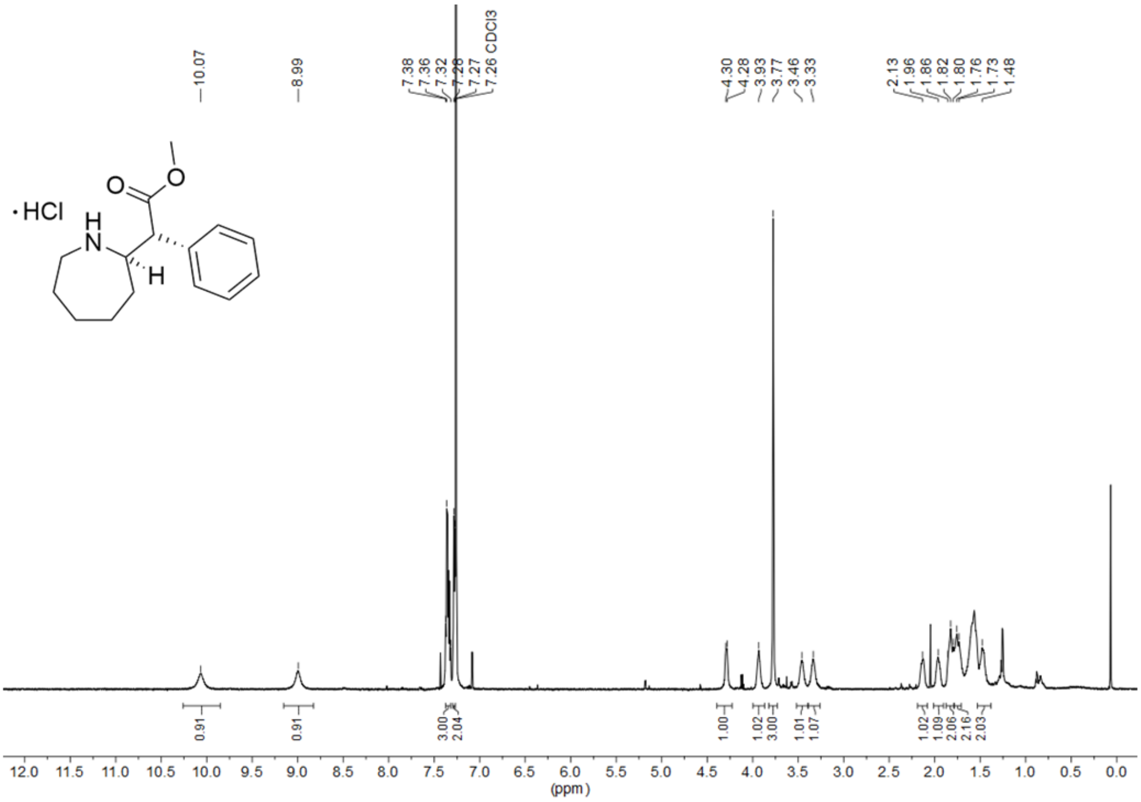

Figure S106: ^{1}H-NMR of **23** (600 MHz, $CDCl_3$).

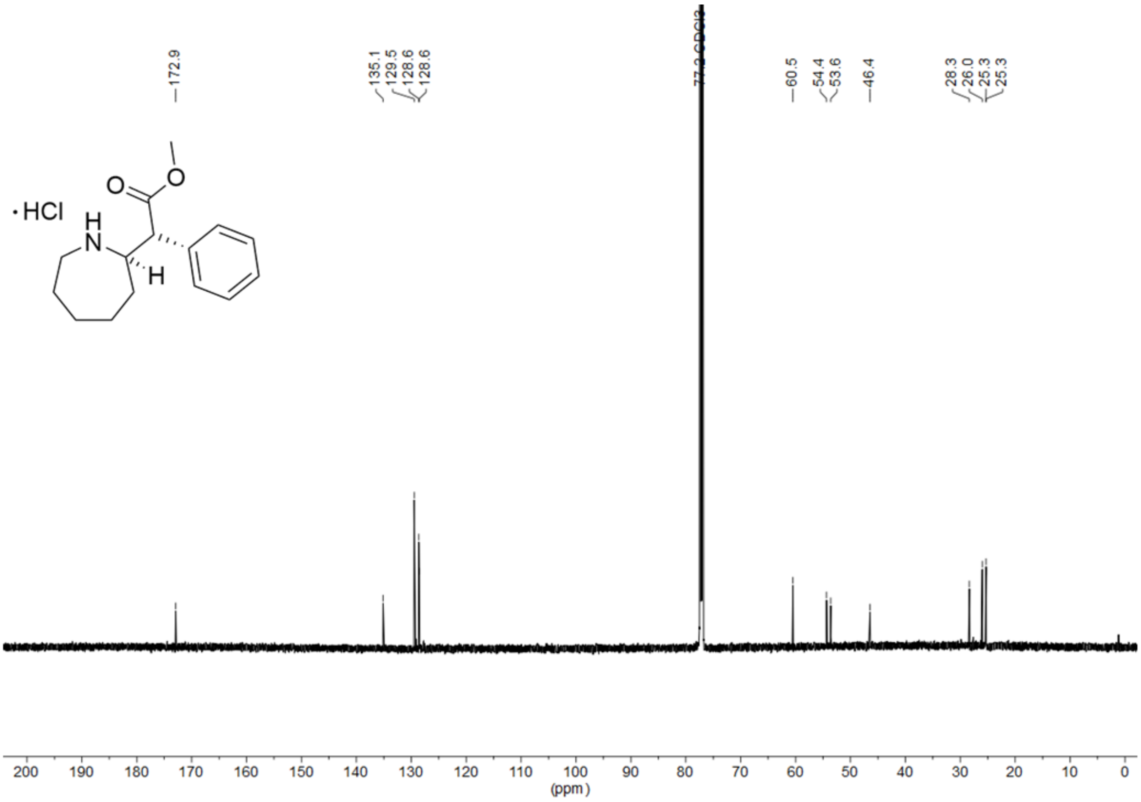

Figure S107: $^{13}C\{^{1}H\}$-NMR of **23** (151 MHz, $CDCl_3$).

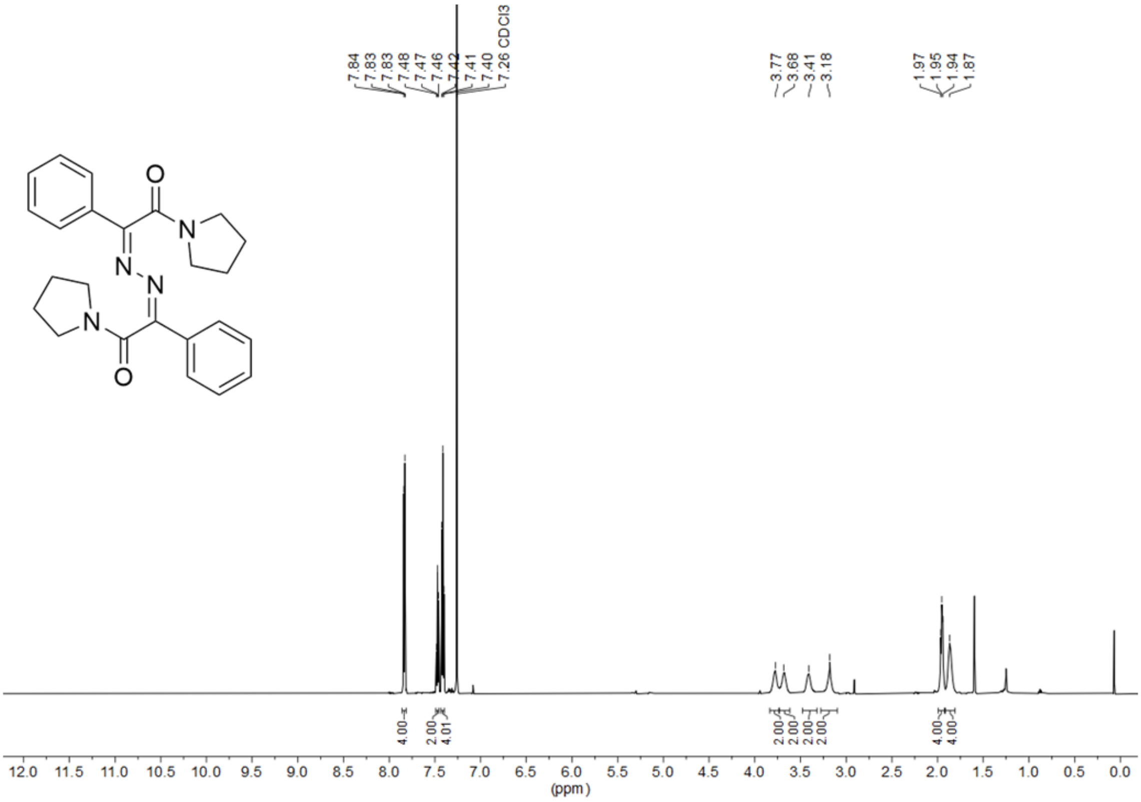

Figure S108: ^{1}H-NMR of **S16** (600 MHz, $CDCl_3$).

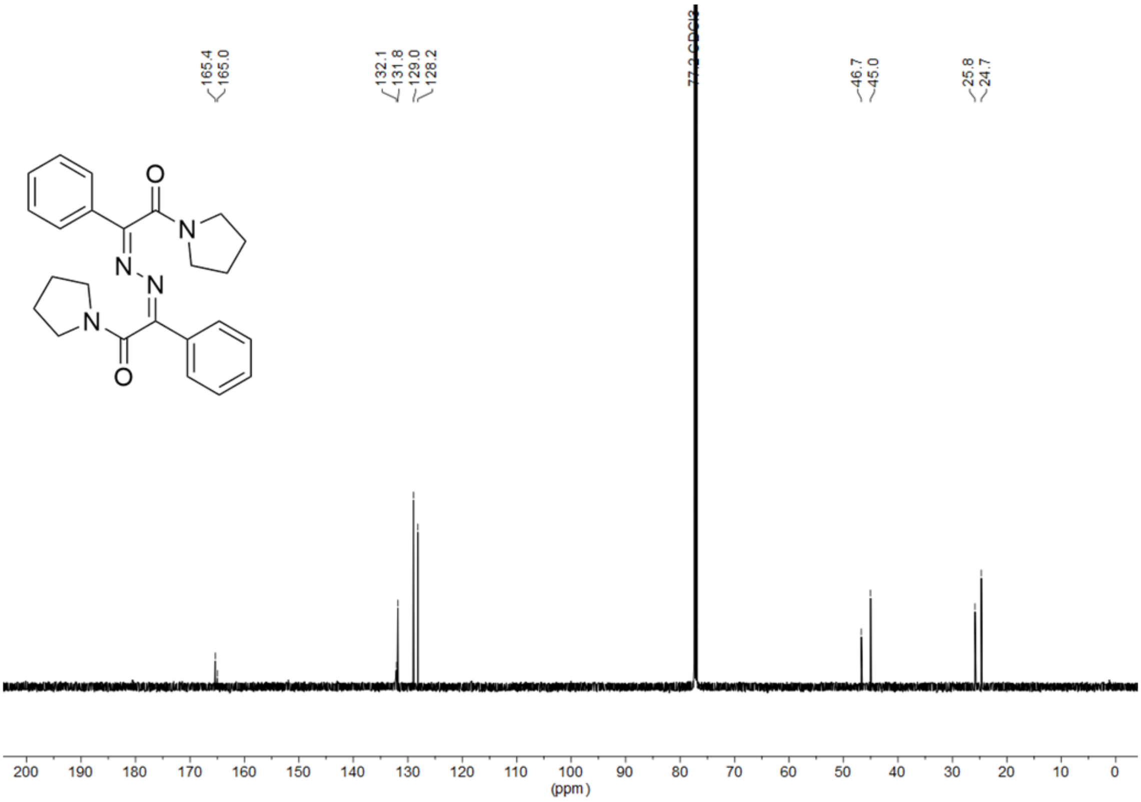

Figure S109: $^{13}C\{^{1}H\}$-NMR of **S16** (151 MHz, $CDCl_3$).

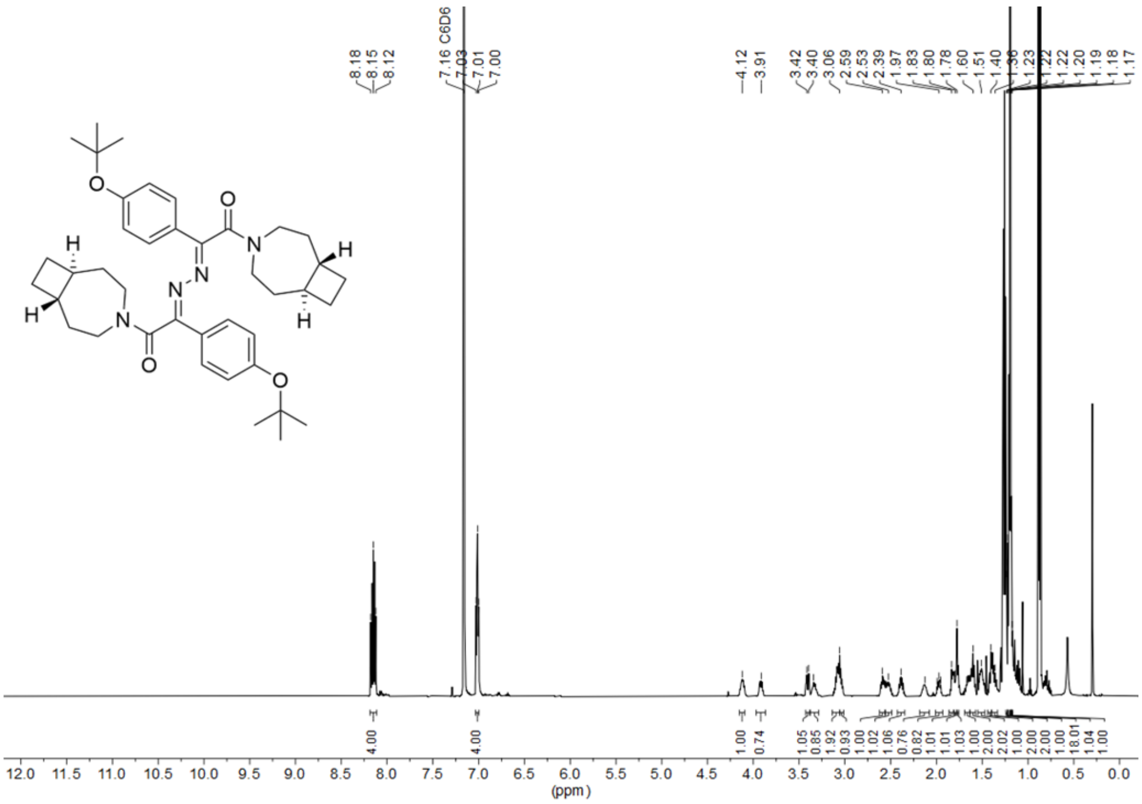

Figure S110: ^{1}H-NMR of **19** (600 MHz, C_6D_6).

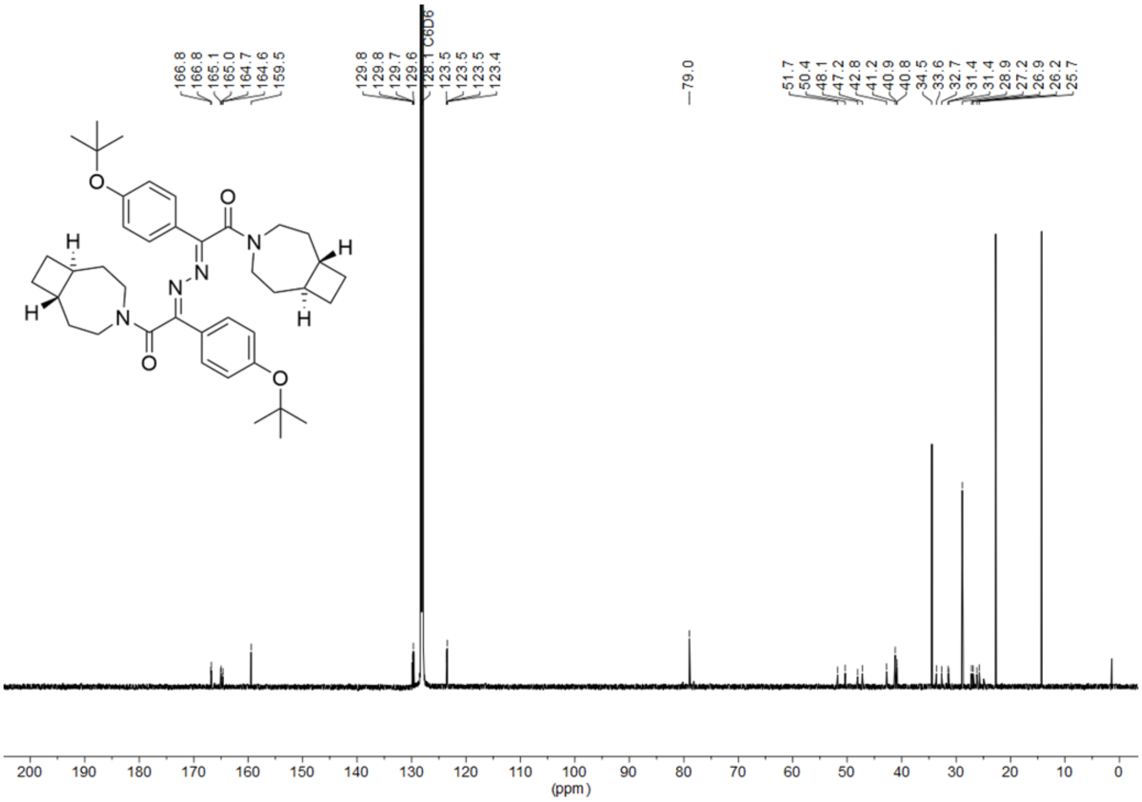

Figure S111: $^{13}C\{^{1}H\}$-NMR of **19** (151 MHz, C_6D_6).

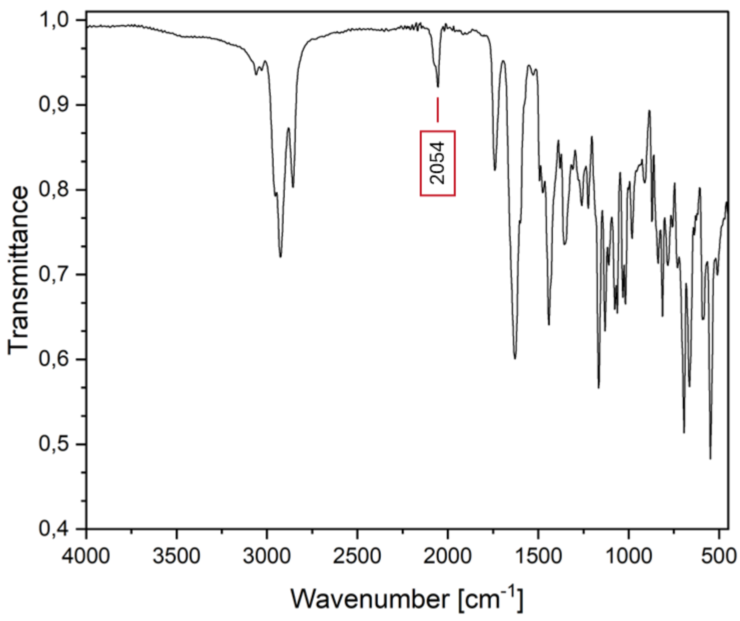

Figure S112: IR spectrum of **S7** with the characteristic $\tilde{\nu}(N_2) = 2054$ cm^{-1} absorption band.

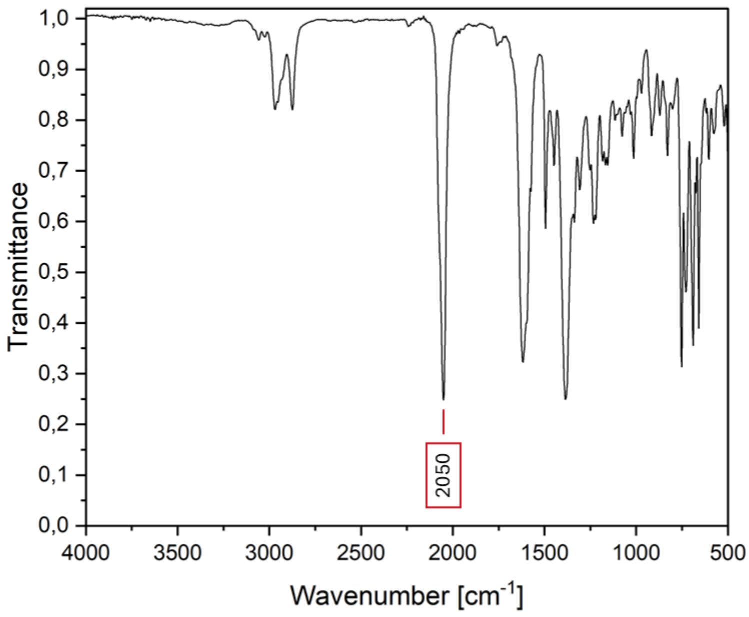

Figure S113: IR spectrum of **S10** with the characteristic $\tilde{\nu}(N_2) = 2050$ cm^{-1} absorption band.

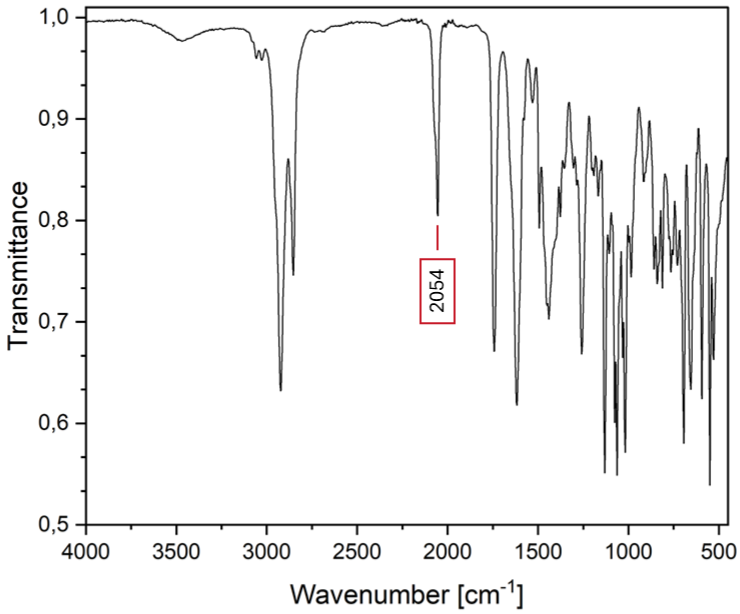

Figure S114: IR spectrum of **S15** with the characteristic $\tilde{\nu}(N_2) = 2054$ cm^{-1} absorption band.

5 References

[1] J. M. Hoyt, V. A. Schmidt, A. M. Tondreau, P. J. Chirik, *Science* **2015**, *349*, 960–963.

[2] L. E. Hertwig, T. Bender, F. J. Becker, P. Jäger, S. Demeshko, S. J. Gross, J. Ballmann, D.-A. Roşca, *ACS Catal.* **2023**, *13*, 6416–6429.

[3] Y. He, B. Sun, X. Lu, Y. Zhou, F.-L. Zhang, *J. Org. Chem.* **2023**, *88*, 4345–4351.

[4] D. J. Lapinsky, R. Velagaleti, N. Yarravarapu, Y. Liu, Y. Huang, C. K. Surratt, J. R. Lever, J. D. Foster, R. Acharya, R. A. Vaughan, H. M. Deutsch, *Bioorg. Med. Chem.* **2011**, *19*, 504–512.

[5] E. J. Corey, A. M. Felix, *J. Am. Chem. Soc.* **1965**, *87*, 2518–2519.

[6] V. George, B. König, *Chem. Commun.* **2023**, *59*, 11835–11838.

[7] C. Zhang, X. Zong, L. Zhang, N. Jiao, *Org. Lett.* **2012**, *14*, 3280–3283.

[8] S. Singh, S. Popuri, Q. M. Junaid, S. Sabiah, J. Kandasamy, *Org. Biomol. Chem.* **2021**, *19*, 7134–7140.

[9] H. F. Kung, J. D. Winkler, *J. Org. Chem.* **1998**, *63*, 9628–9629.

[10] G. Shen, L. Zhao, Y. Wang, T. Zhang, *RSC Adv.* **2016**, *6*, 78307–78310.

[11] K. Kabsch in *International Tables for Crystallography*, *Vol. F*, (Eds.: M. G. Rossmann, E. Arnold), Kluwer Academic Publishers, Dordrecht, The Netherlands, **2001**, Chapter 11.3.

[12] (a) *CrysAlisPro*, Agilent Technologies UK Ltd., Oxford, UK **2011–2014**; (b) Rigaku Oxford Diffraction, Rigaku Polska Sp. z o.o., Wrocław, Poland **2015–2020**.

[13] (a) *SCALE3 ABSPACK*, *CrysAlisPro*, Agilent Technologies UK Ltd., Oxford, UK **2011–2014**; (b) Blessing, R. H., *Acta Cryst.* **1995**, *A51*, 33–38.

[14] W. R. Busing, H. A. Levy, *Acta Cryst.* **1957**, *10*, 180–182.

[15] O. V. Dolomanov, L. J. Bourhis, R. J. Gildea, J. A. K. Howard, H. Puschmann, *J. Appl. Cryst.* **2009**, *42*, 339–341.

[16] (a) Sheldrick, G. M., *SHELXT*, University of Göttingen and Bruker AXS GmbH, Karlsruhe, Germany, **2012–2018**; (b) Ruf, M.; Noll, B. C., Application Note SC-XRD 503, Bruker AXS GmbH, Karlsruhe, Germany **2014**; (c) Sheldrick, G. M., *Acta Cryst.* **2015**, *A71*, 3–8.

[17] (a) Sheldrick, G. M., *SHELXL-20xx*, University of Göttingen and Bruker AXS GmbH, Karlsruhe, Germany **2012–2018**; (b) Sheldrick, G. M., *Acta Cryst.* **2008**, *A64*, 112–122; (c) Sheldrick, G. M., *Acta Cryst.* **2015**, *C71*, 3–8.

[18] (a) Rollett, J. S. in *Crystallographic Computing*, Eds. Ahmed, F. R.; Hall, S. R.; Huber C. P., Munksgaard, Copenhagen, Denmark, p. 167, **1970**; (b) Watkin, D. in *Crystallographic Computing 4*, Eds. Isaaks, N. W.; Taylor, M. R.; Chern, D., IUCr and Oxford University Press, Oxford, UK, **1988**; (c) Müller, P.; Herbst-Irmer, R.; Spek, A. L.; Schneider, T. R.; Sawaya, M. R., in *Crystal Structure Refinement*, Ed. Müller, P., Ch. 5, Oxford University Press, Oxford, UK, **2006**; (d) Watkin, D., *J. Appl. Cryst.* **2008**, *41*, 491–522.

[19] A. Thorn, B. Dittrich, G. M. Sheldrick, *Acta Cryst.* **2012**, *A68*, 448–451.

[20] (a) S. Grimme, C. Bannwarth, P. Shushkov, *J. Chem. Theory Comput.* **2017**, *13*, 1989–2009; (b) C. Bannwarth, S. Ehlert, S. Grimme, *J. Chem Theory Comput* **2019**, *15*, 1652–1671.

[21] S. Grimme, F. Bohle, A. Hansen, P. Pracht, S. Spicher, M. Stahn, *J. Phys. Chem. A* **2021**, *125*, 4039–4054.

[22] C. Bannwarth, E. Caldeweyher, S. Ehlert, A. Hansen, P. Pracht, J. Seibert, S. Spicher, S. Grimme, *WIREs Comput. Mol. Sci.* **2020**, *11*, e1493.

[23] (a) F. Neese, *WIREs Computational Molecular Science* **2011**, *2*, 73–78; (b) F. Neese, *WIREs Computational Molecular Science* **2025**, *15*, e70019.

[24] S. Grimme, C. Bannwarth, S. Dohm, A. Hansen, J. Pisarek, P. Pracht, J. Seibert, F. Neese, *Angew. Chem. Int. Ed.* **2017**, *56*, 14763–14769.

[25] M. W. Lodewyk, M. R. Siebert, D. J. Tantillo, *Chem. Rev.* **2012**, *112*, 1839–1862.

[26] S. G. Smith, J. M. Goodman, *J. Am. Chem. Soc.* **2010**, *132*, 12946–12959.

[27] (a) M. A. Iron, *J. Chem Theory Comput* **2017**, *13*, 5798–5819; (b) M. O. Marcarino, S. Cicetti, M. M. Zanardi, A. M. Sarotti, *Nat. Prod. Rep.* **2022**, *39*, 58–76.

[28] A. Kaupang, T. Bonge-Hansen, *Beilstein J. Org. Chem.* **2013**, *9*, 1407–1413.
